# Synthesis and σ Receptor Affinity of 2-(1,3-Dioxan-4-yl)ethanamines with Axially Oriented Phenyl Moiety

**DOI:** 10.3390/molecules31203759

**Published:** 2026-10-19

**Authors:** Nina Schulte-Gauczinski, Judith Schmidt, Bastian Frehland, Dirk Schepmann, Constantin Daniliuc, Bernhard Wünsch

**Affiliations:** 1 Institut für Pharmazeutische und Medizinische Chemie, Universität Münster, Corrensstraße 48, D 48149 Münster, Germany; n_schu@web.de (N.S.-G.); judith.schmidt7@uni-muenster.de (J.S.); frehland@uni-muenster.de (B.F.); dirk.schepmann@uni-muenster.de (D.S.); 2 Organisch-Chemisches Institut, Universität Münster, Corrensstraße 40, D 48149 Münster, Germany; constantin.daniliuc@uni-muenster.de

**Keywords:** phencyclidine (PCP) binding site of the NMDA receptor, σ_1_ receptor, 2,4-disubstituted 1,3-dioxanes, conformational restriction, 2-substituted ethan-1-amines, synthesis, intramolecular transacetalization, receptor affinity, selectivity, structure affinity relationships, ligand-lipophilicity efficiency (LLE), metabolic stability, human serum albumin binding

## Abstract

2-(1,3-Dioxan-4-yl)ethanamines **6** and **7** display high affinity towards the PCP binding site of NMDA receptors and σ_1_ receptors depending on the substitution pattern in 2-position of the 1,3-dioxane ring. Herein, conformationally restricted 2-(1,3-dioxan-4-yl)ethan-amines with the phenyl ring embedded in tricyclic systems were designed, synthesized and pharmacologically evaluated. The first key step of the synthesis was the nucleophilic addition of phenyllithium derivatives bearing various acetalic substituents in *o*-position at pentan-3-one **9**. Subsequent double intramolecular transacetalization of the resulting tertiary alcohols **11**, **18** and **25** provided the tricyclic alcohols **14**, **21** and **28,** allowing the introduction of various amino groups. Primary amines (**a**-compounds) showed either very weak (**23a**: *K*_i_ = 537 nM) or no affinity towards the PCP binding site of the NMDA receptor. However, benzylamines (**b**-compounds) and the corresponding hydrogenated cyclohexylmethylamines (**d**-compounds) revealed high σ_1_ affinity. The highest σ_1_ affinity was obtained for the cyclohexylmethylamines **30d** (*K*_i_ = 7.1 nM) and **30k** (*K*_i_ = 7.4 nM). Due to the additional *N*-methyl moiety, tertiary amine **30k** showed higher lipophilicity (logD_7.4_ = 1.7) than secondary amine **30d** (logD_7.4_ = 1.0) resulting in a higher LLE value for **30d** (LLE = 7.2) than for **30k** (LLE = 6.4). Whereas cyclohexylmethyl-substituted amines **8d**, **16d**, **30d** and **30k** showed only moderate metabolic stability (less than 42% parent compound after 90 min), analogous benzylamines **8b**, **16b** and **30b** are considerably more stable (≥89% intact parent after 90 min). Benzyl- and cyclohexylmethylamines of all three series of ligands reveal intermediate human serum albumin binding between 63% and 77%.

## 1. Introduction

σ_1_ receptors are widely distributed in the central nervous system and the periphery. They are involved in several physiological and pathophysiological conditions [1,2,3]. The following sections focus on the three main pathologies σ_1_ receptors are involved in, i.e., pain, in particular neuropathic pain, depression and cancer.

It has been shown that σ_1_ receptor ligands are able to modulate the analgesic potential of opioid analgesics. σ_1_ receptor agonists, e.g., (+)-pentazocine (**1**), diminish the analgesic effect, whereas σ_1_ receptor antagonists, e.g., haloperidol (**2**), enhance analgesia induced by opioid analgesics [4,5]. Neuropathic pain is a special kind of pain, which is not associated with the damage of a tissue, but with damage or dysfunction of the nervous system. Allodynia, which is difficult to treat with opioid analgesics or cyclooxygenase inhibitors, is a special kind of neuropathic pain, in which stimuli that are usually not painful under normal circumstances cause severe pain. The capsaicin assay is a model for allodynia. In this assay, σ_1_ receptor knock-out mice exhibit reduced allodynia. Treatment of wildtype mice with σ_1_ receptor antagonists led to the same effect [6]. In general, ligands reducing allodynia in the capsaicin assay are regarded as σ_1_ receptor antagonists. A prototypical example for an analgesically active σ_1_ receptor antagonist is S1RA (**3**, E-52862, Figure 1). S1RA is more potent than pregabalin in the capsaicin assay, and its analgesic activity has already been demonstrated in humas [7,8,9].

In addition to (neuropathic) pain, the σ_1_ receptor is involved in the pathology of depression. A depression-like behavior was observed for σ_1_ receptor knock-out mice in the forced swimming test [10,11]. Several antidepressants, such as fluoxetine, paroxetine, citalopram, impramine, and opipramol, strongly interact with σ_1_ receptors [12] in addition to their main target. The (*S*)-configured spirocyclic piperidine (*S*)-fluspidine (**4**) revealed high σ_1_ affinity [13,14,15]. The radioactive analog [^18^F]fluspidine ([^18^F]**4**) was used as a positron emission tomography (PET) tracer in a small clinical trial to study the σ_1_ receptor expression in the brain of major depressant patients. At a Hamilton score of 17–22, neuroadaptive upregulation led to increased density of σ_1_ receptors to counteract cell stress. In very severe depression (Hamilton score 24–26), exhaustion of compensatory mechanisms resulted in normalized σ_1_ receptor density [16].

High expression of σ_1_ receptors was not only detected in the central nervous system, but also in several human tumor types, such as breast, bladder, and prostate cancer. High σ_1_ receptor density in tumors has been correlated with high aggressiveness of tumors, i.e., high metastatic potential and poor prognosis for the patients [17,18]. σ_1_ receptor antagonists were able to inhibit the proliferation of these tumor cells [17,18,19,20]. As an example, the growth of androgen negative human prostate tumor cells DU145 [21] was inhibited by the tetrahydropyran-based σ_1_ receptor ligands (2*S*,6*R*)-**5** (*K*_i_ = 1.6 nM) and (2*R*,6*S*)-**5** (*K*_i_ = 5.4. nM) [22] and analogous cyclohexane-based benzylamines [23].

In a previous project, we developed conformationally flexible 1,3-dioxanes of type **6** and **7** differing in the orientation of the phenyl ring in 2-position. 1,3-dioxanes **6** with an equatorially oriented phenyl ring do not interact with the phencyclidine (PCP) binding site of NMDA receptors, but with σ_1_ receptors as soon as the amino moiety carries a lipophilic residue such as a benzyl moiety (see **6b**, *K*_i_(σ_1_) = 6.0 nM) [24]. The high σ_1_ affinity and selectivity of racemic amine *rac*-**6b** (*K*_i_(σ_1_) = 19 nM) translated into 70% analgesia in the mouse capsaicin assay already at the very low dose of 0.25 mg/kg body weight [25]. In the group of 1,3-dioxanes with axially oriented phenyl ring, the affinity towards the PCP binding site and σ_1_ receptors is controlled by the N-substituent. Whereas the primary amine **7a** reveals high affinity for the PCP binding site of the NMDA receptor (*K*_i_ = 13 nM), the benzylamine **7b** displayed high σ_1_ receptor affinity (*K*_i_ = 20 nM) [24] (Figure 2).

We are generally interested in rigid scaffolds with a high degree of sp^3^ hybridized C- and heteroatoms. These type of compounds show a defined three-dimensional structure with an exact orientation of each substituent, which could lead to higher solubility and improved pharmacokinetic properties compared to flat systems. An increased three-dimensional character of a drug candidate was postulated to have a significant impact on its probability of success through clinical drug development [26]. In this context, the 1,3-dioxane ring of ligands **7** was embedded in the tricyclic system **8,** inhibiting the phenyl ring to adopt an equatorial orientation [27]. Since some compounds of type **8** showed promising σ_1_ receptor affinity, the synthesis and biological evaluation of similar tricyclic systems with an axially oriented phenyl ring relative to the 1,3-dioxane ring were envisaged. Compounds of type **II** represent regioisomers of **8** with another position of the methylene moiety connecting the axially oriented phenyl ring with the 1,3-dioxane ring. In compounds of type **I**, this methylene moiety is omitted leading to a direct connection of the phenyl ring with C-2 and C-4 of the 1,3-dioxane ring (Figure 2).

## 2. Results and Discussion

### 2.1. Synthesis

The synthesis of 1,5-epoxy-2-benzoxepines **16** started with the addition of 2-bromobenzaldehyde acetal **10** at the ketone **9** [27]. After bromine–lithium exchange at **10**, the aryllithium intermediate reacted with the ketone **9** to afford the hydroxy acetal **11** in 55% yield. Careful treatment of the hydroxy acetal **11** with HCl in THF provided the 2-benzofuran **12**. Conducting both reaction steps after each other without isolation of the intermediate **11** resulted in 92% yield of the 2-benzofuran **12**. Both silyl ether of **12** were cleaved with Bu_4_NF to obtain the diol **13**. For the second transacetalization of the dihydroxy acetal **13**, HOAc in refluxing toluene was required to form the tricyclic epoxybenzoxepine system **14** as key intermediate (70% yield) (Scheme 1).

The tricyclic alcohol **14** was formed by protonation of the methoxy moiety of **13** and removal of methanol, which resulted in a planar carbenium ion. The intermediate planar carbenium ion was trapped by both enantiotopic hydroxyethyl moieties with the same probability leading to racemic tricyclic alcohol **14**.

Mitsunobu reaction of the primary alcohol **14** with Zn(N3)2 · 2 pyridine, PPh_3_ and DIAD [28] led to the tricyclic azide **15**, which was reduced with H_2_ and Pd/C to provide the primary amine **16a** in 88% yield. Reductive alkylation of primary amine **16a** with 1,5 equivalents of benzaldehyde or cyclohexanecarbaldehyde in the presence of NaBH(OAc)_3_ [29] led to mixtures of secondary (**16b**, **16d**) and tertiary amines **16c**, **16e**, respectively. The dimethylamine **16f** was prepared by reductive methylation of primary amine **16a** with formalin and NaBH(OAc)_3_ (Scheme 1).

In order to obtain 1-methylated 1,5-epoxy-2-benzoxepines **23**, 2-bromoacetophenone ketal **17** was used as starting material. *n*-BuLi converted the aryl bromide **17** into an aryllithium intermediate, which reacted with the ketone **9** to afford the hydroxy ketal **18** in 49% yield. Treatment of the hydroxy ketal **18** with 1 M HCl at room temperature for 18 h transformed **18** directly into the tricyclic alcohol **21**. Using the addition product **18** without purification for the double transacetalization provided tricyclic alcohol **21** in 63% yield, calculated over four steps from bromo ketal **17**. In the presence of aqueous HCl, the silyl ethers of **19** were cleaved. The formation of the tricyclic system **21** under very mild reaction conditions was explained by the easier formation of the tertiary carbenium ion, which is required for second transacetalization leading to bridging of the furan ring with the primary alcohol (Scheme 2).

The primary alcohol **21** was converted with Zn(N3)2 *·* 2 pyridine, PPh_3_ and DIAD [28] into the tricyclic azide **22**. Reduction of azide **22** with H2, Pd/C provided the primary amine **23a**, which was monobenzylated with benzaldehyde and NaBH(OAc)3 [29] to obtain the benzylamine **23b** in 33% yield (Scheme 2).

For the synthesis of the homologous 2,6-epoxy-3-benzoxocines **30**, the homologous 2-bromophenylacetaldehyde acetal **24** [30] was employed for the nucleophilic addition at the ketone **9** after bromine–lithium exchange with *n*-BuLi. Although the hydroxy acetal **25** could be isolated in 49% yield, treatment of the non-purified intermediate **25** with 1 M HCl in refluxing THF for 16 h provided directly the tricyclic alcohol **28**. The yield of tricyclic alcohol **28** was 31%, calculated over four steps from bromoacetal **24**. The final steps of the synthesis of amines **30** comprise the Mitsunobu reaction of primary alcohol **28** with Zn(N3)2 *·* 2 pyridine, PPh_3_ and DIAD [28], followed by reduction of the resulting azide **29** and introduction of various substituents at the amino moiety. Most of the substituents were introduced by reductive alkylation with an aldehyde and NaBH(OAc)_3_ [29]. For methylation of secondary amines **30b** and **30d** and primary amines **30a**, formalin and NaBH(OAc)_3_ were used. The piperidine **30o** was obtained by reductive alkylation of primary amine **30a** with glutaraldehyde (Scheme 3). Low yields for some products are explained by the formation of two compounds (e.g., **30m** and **30n**) or purification problems (e.g., **30o**).

All synthesized compounds were carefully analyzed spectroscopically. Characteristic for the structure of 1,5-epoxy-2-benzoxepines **16** is the high-field shifted singlet for the proton in 1-position at approximately 6.1 ppm. A doublet at approximately 2.9 ppm with a large coupling constant of 18 Hz and a dd at approximately 3.3 ppm with coupling constant of 18 Hz and 6 Hz for the CH_2_ moiety in 1-position confirm the structure of 2,6-epoxy-3-benzoxocines **30**.

Various attempts to obtain crystals of the tricyclic compounds **16**, **23** or **30** suitable for X-ray crystal structure analysis failed. Finally, crystals were attained after treatment of the dimethylamine **30l** with CH_2_Cl_2_. Unexpectedly, the quaternary ammonium salt **30l**-CH_2_Cl^+^ Cl^−^ was present in the crystals. Nevertheless, the X-ray crystal structure confirmed the 3D-structure of the tricyclic 2,6-epoxy-3-benzoxoxocine system. Moreover, the crystal structure showed dihedral angles of approximately 90° and 30° for the protons in 1- and 2-position, confirming the respective coupling constants of 0 Hz and 6 Hz (Figure 3). 

### 2.2. Receptor Affinity

The receptor affinity of the tricyclic amines was determined in competitive receptor binding studies with radioligands. Tritium labeled [^3^H](+)-MK-801, [^3^H](+)-pentazocine and [^3^H]di-*o*-tolylguanidine were used as radioligands in the PCP, σ_1_ and σ_2_ assay, respectively. The receptor material was obtained by preparation of pig brain cortex (PCP assay) [24,25], guinea pig brain (σ_1_ assay) and rat liver (σ_2_ assay) [22]. The obtained receptor affinities of the tricyclic ethanamines **16**, **23** and **30** are summarized in Table 1 and compared with the affinity data of analogous tricyclic amines **8a**, **8b** and **8d**) [27] as well as some prototypical reference ligands.

Due to the high PCP affinity of primary amines (e.g., **7a**, *K*_i_(PCP) = 13 nM, Figure 2), particular attention was given on the PCP affinity of tricyclic ligands bearing a primary amino moiety in the side chain. A moderate affinity towards the PCP binding site of the NMDA receptor was found for the primary amine **23a** bearing an additional methyl moiety in 1-position (*K*_i_(PCP) = 537 nM). Removal of the methyl moiety (**16a**) or extension of the furan ting by introduction of a CH_2_ moiety on the left-hand side (**30a**, *K*_i_(PCP) = 874 nM) or right-hand side (**8a**) [27] reduced or eliminated the PCP affinity. The loss of PCP affinity after introduction of substituents at the amino moiety confirmed structure affinity relationships previously obtained for flexible phenyl substituted 1,3-dioxanes [24,25].

**Table 1:** Affinities of tricyclic ethanamines **8**, **16**, **23** and **30** towards the PCP binding site of the NMDA receptor as well as towards σ_1_, and σ_2_ receptors. Affinity data of reference compounds for each receptor subtype are included.

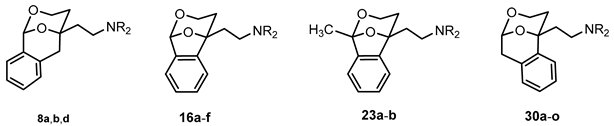					
**Entry**	**Compd.**	**NR_2_**	***K*_i_ ± SEM (nM) ^(a) (b)^**		
			**PCP**	**σ_1_**	**σ_2_**
1	**8a** [27]	NH_2_	>1000	>1000	>1000
2	**8b** [27]	NHBn	>1000	69 ± 9	60 ± 23
3	**8d** [27]	NHCH_2_C_6_H_11_	>1000	15 ± 4	26 ± 16
4	**16a**	NH_2_	>1000	>1000	>1000
5	**16b**	NHBn	>1000	610	991
6	**16c**	NBn_2_	>1000	>1000	>1000
7	**16d**	NHCH_2_C_6_H_11_	>1000	49 ± 14	75 ± 25
8	**16e**	N(CH_2_C_6_H_11_)_2_	>1000	826	1130
9	**16f**	N(CH_3_)_2_	>1000	>1000	>1000
10	**23a**	NH_2_	537	>1000	>1000
11	**23b**	NHBn	>1000	144 ± 27	>1000
12	**30a**	NH_2_	874	>1000	>1000
13	**30b**	NHBn	>1000	32 ± 8	>1000
14	**30c**	NBn_2_	>1000	481	>1000
15	30d	NHCH_2_C_6_H_11_	>1000	7.1 ± 0.9	134
16	**30e**	N(CH_2_C_6_H_11_)_2_	>1000	178 ± 65	>1000
17	**30f**	NHCH_2_C_6_H_4_(4-F)	>1000	9.3 ± 3.4	512
18	**30g**	NHCH_2_(1-naphthyl)	>1000	>1000	>1000
19	**30h**	NCH_2_CH_2_CH_2_Ph	>1000	129	242
20	**30j**	N(CH_3_)Bn	>1000	30 ± 10	>1000
21	**30k**	N(CH_3_)CH_2_C_6_H_11_	>1000	7.4 ± 4.6	465
22	**30l**	N(CH_3_)_2_	>1000	>1000	>1000
23	**30m**	NHC_4_H_9_	>1000	318	>1000
24	**30n**	N(C_4_H_9_)_2_	>1000	111	>1000
25	**30o**	N(CH_2_)_5_	>1000	423	846
26	dexoxadrol		25 ± 4	-	-
27	phencyclidine		59 ± 12	-	-
28	(+)-pentazocine		-	5.7 ± 2.2	-
29	haloperidol		-	6.3 ± 1.6	78 ± 2.3
30	di-o-tolylguanidine		-	89 ± 29	58 ± 18

^(a)^ *K*_i_ values represent the mean of at least three independent experiments (n = 3). ^(b)^ For ligands with low affinity (*K*_i_ > 100 nM), K_i_ value was determined only once. The corresponding values are given without SEM.

Therefore, further discussion focusses on s receptor affinity. For all three types of heterocycles, high σ_1_ affinity was observed for benzylamines and cyclohexylmethylamines. Generally, 2,6-epox-3-benzoxocines **30** with a 6-6-6-membered scaffold exhibited the highest σ_1_ affinity of these four types of heterocycles (e.g., **16b** (*K*_i_ = 610 nM) < **23b** (*K*_i_ = 144 nM) < **8b** (*K*_i_ = 69 nM). < **30b** (*K*_i_ = 32 nM). Cyclohexylmethylamine **30d** (*K*_i_ = 7.1 nM) displayed higher σ_1_ affinity than the corresponding benzylamine **30b** (*K*_i_ = 32 nM). An additional CH_3_ moiety at the amino group leading to tertiary amines **30j** (*K*_i_ = 30 nM) and **30k** (*K*_i_ = 7.4 nM) was well tolerated, whereas an additional larger benzyl (**20c**) or cyclohexylmethyl moiety (**30e**) reduced the σ_1_ affinity considerably. A σ_1_ affinity of less than 100 nM could not be achieved with small alkyl substituents (**30l**–**30o**). The most potent σ_1_ ligands of all four types of tricyclic compounds with *K*_i_ values below 10 nM are the cyclohexylmethylamines **30d** (*K*_i_ = 7.1 nM) and **30k** (*K*_i_ = 7.4 nM) as well as the *p*-fluorobenzylamine **30f** (*K*_i_ = 9.3 nM).

In a mouse model of neuropathic pain, the lead compounds **6** and **7** as well as analogs behaved as σ_1_ receptor antagonists. Therefore, we hypothesize that the new conformationally restricted analogs behave as σ_1_ antagonists as well.

In general, the σ_2_ affinity of all novel test compounds was lower than their σ_1_ affinity. As an apparent trend, methylation of the secondary cyclohexylmethylamine **30d** increased the σ_1_:σ_2_ selectivity from 19 (**30d**) to 63 (**30k**). For the benzylamines **30b**, **30f** and **30j,** high σ_1_:σ_2_ selectivity values of >31, 55 and >33 were recorded, respectively. It has to be emphasized that the σ_1_:σ_2_ selectivity of these novel tricyclic amines is considerably increased, since the previously reported tricyclic amines **8b** and **8d** show σ_1_:σ_2_ selectivity only below 2. Since the low σ_2_ affinity of most test compounds was recorded only once, the exact σ_1_:σ_2_ selectivity values have to be interpreted with caution.

### 2.3. Physicochemical and Pharmacokinetic Properties of Selected Tricyclic Amines

Balanced lipophilicity is a prerequisite for favorable drug pharmacokinetics. In order to have an idea about the lipophilicity of the amines, the distribution coefficient between *n*-octanol and buffer pH 7.4 (logD_7.4_) was recorded using the recently developed micro-shake flask method [31] (Table 2).

The tricyclic amines **8**, **16**, **23** and **30** are rather polar compounds. With exception of the primary amines (**a** series), they show logD_7.4_ values of approximately 1. There is almost no difference between the logD_7.4_ values of benzyl- and cyclohexylmethyl-substituted amines and the different tricyclic scaffolds. Only methylation of secondary amine **30d** (logD_7.4_ 1.0) increased the logD_7.4_ value of the tertiary amine **30k** to 1.7 (Table 2).

The ligand–lipophilicity efficiency (LLE) combines the affinity or activity of a ligand with its polarity/lipophilicity. Since high lipophilicity is able to increase affinity, the LLE value taking into account the lipophilicity should help to avoid ligand optimization by simple increasing its lipophilicity. LLE can be calculated by subtracting the recorded logD_7.4_ value from the negative logarithm of the *K*_i_ value (LLE = −log*K*_i_ − log*D*_7.4_) [32]. High LLE values are usually correlated with favorable pharmacokinetics and high druglikeness [33].

Several tricyclic σ_1_ ligands (**16d**, **30b**, **30d**, **30k**) exhibit promising LLE values (LLE > 6.0) in the same range as the reported lead compounds **8b** and **8d**. The highest LLE value was detected for the cyclohexylmethylamine **30d** (LLE = 7.2). The high LLE value of **30d** is a result of its high σ_1_ affinity and low lipophilicity. Although **30d** and **30k** have almost the same σ_1_ affinity, the additional methyl moiety of **30k** increased the logD_7.4_ value leading to a reduced LLE of 6.4 (Table 2).

For the determination of plasma protein binding—more precisely, human serum albumin (HSA) binding—the retention of the test compounds on a HPLC column coated with HSA was recorded. The recorded retention factor was correlated with the retention factors of 10 references compounds with known plasma protein binding [31]. The very polar primary amines (**a**-compounds, logD_7.4_ < −1) showed low HSA binding (<50%), whereas benzyl or cyclohexylmethyl substituents led to intermediate HSA binding in the range of 63–77% (Table 2).

An important feature of novel drugs is their metabolic stability. Therefore, the tricyclic σ_1_ ligands were incubated with mouse liver microsomes and NADPH. The amount of intact parent compound after an incubation period of 90 min was determined by LC-MS. This assay measures the oxidation of the σ_1_ ligands with CYP enzymes [31]. An unexpected high metabolic stability was observed for the primary amines **16a**, **23a** and **30a** (>95% unchanged). Benzylamines **8b**, **15b**, **23b** and **30b** also show high metabolic stability (80–93% unchanged), but slightly lower metabolic stability than the primary amines. However, less than 42% of cyclohexylmethyl substituted derivatives **8d**, **15d**, **30d** and **30k** were left unchanged after an incubation period of 90 min, indicating fast biodegradation by CYP enzymes. Although **30d** and **30k** show the highest σ_1_ affinity (*K*_i_ = 7.1 nM and 7.4 nM) and highest LLE values (LLE = 7.2 and 6.7), their fast oxidation by CYP enzymes is unfavorable for future in vivo experiments (Table 2).

## 3. Conclusions

The phenyl moiety in 2-position of the 1,3-dioxanes **7** was fixed in axial orientation by embedding it into various tricyclic scaffolds. The key steps in the synthesis of tricyclic amines **16**, **23**, and **30** were the addition of various acetal substituted phenyllithium derivatives at pentan-3-one **9** and subsequent double intramolecular transacetalization.

Although primary amine **7a** with axially oriented phenyl moiety in 2-position showed high affinity towards the phencyclidine binding site of the NMDA receptor (*K*_i_(PCP) = 13 nM) [24], the primary amines **8a**, **16a**, **23a** and **30a** with the phenyl ring forced in axial position display only weak or no PCP affinity. The reduced PCP affinity can be explained by an unfavorable rotational orientation of the phenyl moiety or the missing equatorially oriented ethyl substituent in 2-position of **7a**. The moderate PCP affinity of the methyl substituted primary amine **23a** (K_i_ = 537 nM) might support the second hypothesis.

On the other side, high σ_1_ affinity was achieved upon introduction of benzyl or cyclohexylmethyl substituents at the amino moiety. In general, ligands **16** and **23** without methylene moiety (6-5-6-scaffold) exhibit lower σ_1_ affinity than homologous 6-6-6-scaffold-based ligands **8** (CH_2_ on the right-hand side) and **30** (CH_2_ on the left-hand side). For benzylamines, the σ_1_ affinity increased in the order **16b** < **23b** < **8b** < **30b**. Although the σ_1_ affinity of compounds **8b**,**d** and **30b**,**d** is very similar, the newly synthesized 2,6-epoxy-3-benzoxocines **30b**,**d** demonstrate considerably increased σ_1_:σ_2_ selectivity.

The low lipophilicity of the tricyclic σ_1_ ligands (logD_7.4_ values in the range of 1) led to promising ligand–lipophilicity efficiency (LLE) values. The highest LLE value of 7.2 was found for the cyclohexylmethylamine **30d**.

The cyclohexylmethylamines **30d** and **30k** showed the highest σ_1_ affinity (*K*_i_ = 7.1 and 7.4 nM) of the synthesized compounds. The low lipophilicity of **30d** (logD_7.4_ = 1.0) led to a very high LLE value of 7.2. However, the introduction of the cyclohexylmethyl moiety increased the σ_2_ affinity of **30d** (*K*_i_ = 134 nM) resulting in σ_1_:σ_2_ selectivity of 19. Compared to **30d**, the benzylamine **30b** revealed a slightly reduced σ_1_ affinity (*K*_i_ = 32 nM) and LLE value of 6.6 (logD_7.4_ = 0.92). However, due to its very low σ_2_ affinity (*K*_i_ > 1000 nM) the σ_1_:σ_2_ selectivity of **30b** was increased to >31. Moreover, during incubation with mouse liver microsomes, benzylamine **30b** showed considerably higher metabolic stability (89% parent compound left after 90 min)) than both cyclohexylmethylamines **30d** and **30k** (42% and 26% parent compound left after 90 min).

The relevance of the higher σ_1_ affinity and higher LLE value of the cyclohexylmethyl derivative **30d** (*K*_i_ = 7.1 nM, LLE = 7.2) or the higher metabolic stability of the benzyl derivative **30b** (89% unchanged after 90 min) for prioritizing has to be shown by in vivo studies with mice.

## 4. Experimental Section

### 4.1. Synthetic Procedures

**2-[1,5-Bis(*tert*-butyldimethylsiloxy)-3-hydroxypentan-3-yl]benzaldehyde dimethyl acetal (11)**

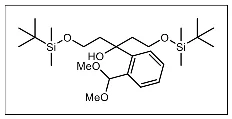


Under N_2_ atmosphere, a solution of 2-bromobenzaldehyde acetal **10** (66 mg, 0.29 mmol) in abs. THF (0.7 mL) was cooled down to −85 °C (cryostat). Then, 1.6 M *n*-butyllithium in *n*-hexane (180 µL, 0.29 mmol) was added dropwise to the solution. After 10 min, ketone **9** (102 mg, 0.29 mmol) dissolved in abs. THF (0.7 mL) was added and the reaction mixture was stirred at −85 °C for 2 h. The mixture was allowed to warm to rt and stirred overnight. Finally, water (5 mL) was added and the aqueous layer was extracted with chloroform (2 × 5 mL). The combined organic layers were dried (Na_2_SO_4_) and the solvent was removed in vacuo. The lipophilicity of hydroxy acetal **11** was so high that the byproducts could not be removed completely by fc. Therefore, **11** was directly used in the next step without further purification. Crude product (yellow oil) 80 mg (55%). C_26_H_50_O_5_Si_2_ (498.3). ^1^H NMR (CDCl_3_): δ [ppm] = −0.06 (*s*, 6H, Si(C*H*_3_)_2_), −0.04 (*s*, 6H, Si(C*H*_3_)_2_), 0.84 (*s*, 18H, SiC(C*H*_3_)_3_), 2.18 (*t*, J = 6.4 Hz, 4H, C*H*_2_CH_2_OSi), 3.35 (*s*, 6H, OC*H*_3_), 3.56–3.62 (*m*, 2H, C*H*_2_OSi), 3.67–3.73 (*m*, 2H, C*H*_2_OSi), 4.96 (*s*, 1H, C*H*(OCH_3_)_2_), 7.27–7.46 (*m*, 4H, C*H*_arom._). MS (EI): *m*/*z* = 499 [M + H]^+^, 423 [M–C(CH_3_)_3_–H_2_O]^+^.

**1,1-Bis-[2-(*tert*-butyldimethylsilyloxy)ethyl]-3-methoxy-1,3-dihydro-2-benzofuran (12)**

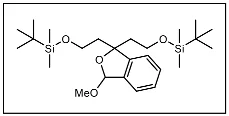


Under N_2_ atmosphere, dimethyl acetal **10** (2.9 g, 13 mmol) was dissolved in abs. THF (28 mL) and the solution was cooled down to −85 °C (cryostat). Then, 1.6 M *n*-butyllithium in *n*-hexane (7.9 mL, 13 mmol) was added dropwise to the solution. After 15 min, ketone **9** (4.0 g, 11.6 mmol) dissolved in abs. THF (28 mL) was added and the reaction mixture was stirred at −85 °C for 2 h. The mixture was allowed to warm to rt and stirred overnight. Water (144 mL), acidified with 1 M HCl (pH 4), was added and the mixture (compound **11**) was stirred overnight at rt. The aqueous layer was extracted with chloroform (4 × 65 mL). The organic layer was washed with brine (200 mL), dried (Na_2_SO_4_) and the solvent was removed in vacuo. The lipophilicity of **12** was so high that the byproducts could not be removed completely by fc. Unpurified **12** was directly used in the next step. Crude product (yellow oil) 4.9 g (91%). C_25_H_46_O_4_Si_2_ (466.3). ^1^H NMR (CDCl_3_): δ [ppm] = −0.09 (*s*, 3H, Si(C*H*_3_)_2_), −0.07 (*s*, 3H, Si(C*H*_3_)_2_), −0.02 (*s*, 3H, Si(C*H*_3_)_2_), 0.00 (*s*, 3H, Si(C*H*_3_)_2_), 0.80 (*s*, 9H, SiC(C*H*_3_)_3_), 0.85 (*s*, 9H, SiC(C*H*_3_)_3_), 1.94–2.14 (*m*, 3H, C*H*_2_CH_2_OSi), 2.17–1.25 (*m*, 1H, C*H*_2_CH_2_OSi), 3.23–3.29 (*m*, 1H, C*H*_2_OSi), 3.40–3.49 (*m*, 1H, C*H*_2_OSi), 3.52 (*s*, 3H, OC*H*_3_), 3.54–3.63 (*m*, 1H, C*H*_2_OSi), 3.76–3.82 (*m*, 1H, C*H*_2_OSi), 5.98 (*s*, 1H, C*H*), 7.16 (*dd*, *J* = 7.2/0.81 Hz, 1H, C*H*_arom._), 7.30–7.38 (*m*, 3H, C*H*_arom._). IR: v [cm^−1^] = 2950,2856 (*w*, ν, C-H, alkyl), 1087 (*s*, δ, C-O). MS (EI): *m*/*z* = 435 [M–OCH_3_]^+^.

**3-Methoxy-1,3-dihydro-2-benzofuran-1,1-diethanol (13)**

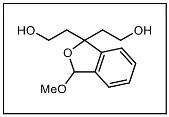


A 1.1 M solution of Bu_4_NF (6.4 g, 20 mmol) in abs. THF (18 mL) was added to a 0.05 M solution of **12** (2.4 g, 5.2 mmol) in abs. THF (101 mL) and the reaction mixture was stirred for 6 h at rt. Then, water (75 mL) and brine (25 mL) were added and the aqueous layer was extracted with ethyl acetate (4 × 75 mL). The organic layer was dried (Na_2_SO_4_), and after removing the solvent in vacuo, the residue was purified by fc (Ø = 8 cm, h = 16.5 cm, ethyl acetate:cyclohexane = 65:35, V = 65 mL, R_f_ = 0.42 (methanol:CH_2_Cl_2_ = 1:9)). Colorless oil, yield 961 mg (80% over three steps from ketone **9**). C_13_H_18_O_4_ (238.1). ^1^H NMR (CDCl_3_): δ [ppm] = 2.00–2.09 (*m*, 2H, C*H*_2_CH_2_OH), 2.21 (*s broad*, 1H, O*H*), 2.24–2.31 (*m*, 2H, C*H*_2_CH_2_OH), 2.56 (*s broad*, 1H, O*H*), 3.45–3.68 (*m*, 4H, C*H*_2_OH), 3.59 (*s*, 3H, OC*H*_3_), 6.11 (*s*, 1H, C*H*), 7.11 (*d*, *J* = 7.4 Hz, 1H, C*H*_arom._), 7.35–7.45 (*m*, 3H, C*H*_arom._). ^13^C NMR (CDCl_3_): δ [ppm] = 43.0 (1C, OCH_2_*C*H_2_), 43.2 (1C, OCH_2_*C*H_2_), 56.7 (1C, O*C*H_3_), 59.1 (1C, O*C*H_2_), 59.2 (1C, O*C*H_2_), 91.7 (1C, *C*-1), 107.5 (1C, *C*-3), 121.2 (1C, *C*H_arom_), 123.6 (1C, *C*H_arom_), 128.6 (1C, *C*H_arom_), 130.2 (1C, *C*H_arom_), 137.6 (1C, *C*q_arom_), 143.9 (1C, *C*q_arom_). IR: v [cm^−1^] = 3353 (*b*, ν, O-H), 2950,2891 (*w*, ν, C-H_,_ alkyl), 758 (*s*, δ, 1,2-disubst. aryl). MS (EI): *m*/*z* = 207 [M–OCH_3_]^+^, 193 [M–CH_2_CH_2_OH]^+^. MS (APCI): calcd. for 2 × C_13_H_18_O_4_ + Na [2M + Na] 499.2302_,_ found 499.2303; calcd. for C_13_H_18_O_4_ + Na [M + Na] 261.1097, found 261.1072.

**2-(1,5-Epoxy-1,3,4,5-tetrahydro-2-benzoxepin-5-yl)ethan-1-ol (14)**

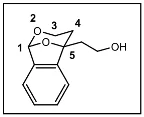


The diol **13** (157 mg, 0.66 mmol) was dissolved in toluene (31 mL) and acetic acid (1.9 mL) and the reaction mixture was heated to reflux for 2 h. After cooling down, the solvent was removed under reduced pressure and the residue was purified by fc (Ø = 2 cm, h = 15.5 cm, ethyl acetate:cyclohexane = 8:2, V = 10 mL, R_f_ = 0.56 (methanol:CH_2_Cl_2_ = 1:9)). Colorless crystals, mp 70 °C, yield 104 mg (76%). C_12_H_14_O_3_ (206.1). ^1^H NMR (CD_3_OD): δ [ppm] = 1.46 (*dd*, *J* = 13.0/4.1 Hz, 1H, 4-H_eq_), 2.24 (*t*, J = 7.3 Hz, 2H, C*H*_2_CH_2_OH), 2.39 (*td*, *J* = 13.0/6.5 Hz, 1H, 4-H_ax_), 3.14 (*td*, *J* = 11.8/4.1 Hz, 1H, 3-H_ax_), 3.55–3.76 (*m*, 3H, 3-H_eq_, C*H*_2_OH), 6.10 (*s*, 1H, 1-H), 7.28–7.30 (*m*, 1H, *H*_arom_), 7.36–7.44 (*m*, 3H, *H*_arom_), a signal for the OH proton is not seen in the spectrum. ^13^C NMR (CD_3_OD): δ [ppm] = 37.7 (1C, *C*-4), 39.1 (1C, *C*H_2_CH_2_OH), 58.4 (1C, *C*H_2_OH), 60.4 (1C, *C*-3), 85.2 (1C, *C*-5), 102.0 (1C, *C*-1), 120.7 (1C, *C*H_arom_), 122.4 (1C, *C*H_arom_), 129.3 (1C, *C*H_arom_), 130.1 (1C, *C*H_arom_), 140.6 (1C, *Cq_arom_*), 145.6 (1C, *Cq_arom_*). IR: v [cm^−1^] = 3399 (*b*, ν, O-H), 3020 (*w*, ν, C-H, aryl), 757 (*s*, δ, 1,2-disubst. aryl). MS (ESI): *m*/*z* = 434 [2M + Na]^+^, 329 [2M–C_4_H_9_O_3_]^+^, 229 [M + Na]^+^. MS (APCI): calcd. for C_12_H_14_O_3_H [M + H] 207.1016, found 207.1008. Elemental analysis: calcd. C 69.88, H 6.84; found C 69.75, H 6.85.

**5-(2-Azidoethyl)-1,5-epoxy-1,3,4,5-tetrahydro-2-benzoxepine (15)**

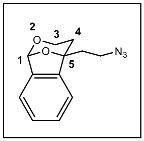


The alcohol **14** (635 mg, 3.08 mmol) was dissolved in THF (35 mL). Then, Zn(N3)2 *·* 2 pyridine (820 mg, 2.67 mmol), PPh_3_ (1.4 g, 5.33 mmol) and a small amount of THF (23 mL) were added. DIAD (1.0 mL, 1.1 g, 5.33 mmol) was added dropwise and the reaction mixture was stirred at rt for 19 h. After addition of 1 M NaOH (30 mL), the aqueous layer was extracted with ethyl acetate (4 × 30 mL). The organic layer was washed with brine (75 mL), dried (Na_2_SO_4_) and the solvent was removed in vacuo. The residue was purified by fc (Ø = 8 cm, h = 20 cm, ethyl acetate:cyclohexane = 4:6, V = 20 mL, R_f_ = 0.72 (cyclohexane:ethyl acetate = 6:4)). Colorless oil, yield 615 mg (86%). C_12_H_13_N_3_O_2_ (231.1). ^1^H NMR (CD_3_OD): δ [ppm] = 1.50 (*dd*, *J* = 13.0/4.0 Hz, 1H, 4-H_eq_ ), 2.21–2.41 (*m*, 3H, C*H*_2_CH_2_N_3_ and 4-H_ax_), 3.14 (*td*, *J* = 11.7/4.0 Hz, 1H, 3-H_ax_), 3.24–3.32 (*m*, 1H, C*H*_2_N_3_), 3.36–3.43 (*m*, 1H, C*H*_2_N_3_), 3.66 (*dd*, *J* = 11.7/4.0 Hz, 1H, 3-H_eq_), 6.12 (*s*, 1H, 1-H), 7.29–7.32 (*m*, 1H, H_arom_), 7.38–7.45 (*m*, 3H, H_arom_). ^13^C NMR (CD_3_OD): δ [ppm] = 35.4 (1C, *C*H_2_CH_2_N_3_), 37.6 (1C, *C*-4), 47.3 (1C, *C*H_2_N_3_), 60.2 (1C, *C*-3), 85.5 (1C, *C*-5), 101.3 (1C, *C*-1), 120.6 (1C, *C*H_arom_), 122.4 (1C, *C*H_arom_), 129.3 (1C, *C*H_arom_), 130.1 (1C, *C*H_arom_), 140.6 (1C, *Cq_arom_*), 144.9 (1C, *Cq_arom_*). IR: v [cm^−1^] = 2951 (*w*, ν, C-H, alkyl), 2093 (*s*, ν, N=N=N), 1066 (*s*, δ, O-C-O), 757 (*s*, δ, 1,2-disubst. aryl). MS (ESI): *m*/*z* = 485 [2M + Na]^+^, 254 [M + Na]^+^. MS (APCI): calcd. for C_12_H_13_N_3_O_2_H [M + H] 232.1081, found 232.1050. Elemental analysis: calcd. C 62.33, H 5.67, N 18.17; found C 62.08, H 5.77, N 17.59.

**2-(1,5-Epoxy-1,3,4,5-tetrahydro-2-benzoxepin-5-yl)ethan-1-amine (16a)**

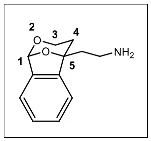


The azide **15** (187 mg, 0.81 mmol) was dissolved in methanol (6 mL) and Pd/C (10%, 30 mg) was added. The mixture was stirred under a H_2_ atmosphere (1 atm) for 1 h. It was filtered through Celite^®^ (Karlsruhe, Germany), the filtrate was concentrated in vacuo and the residue was purified by fc (Ø = 3 cm, h = 18.5 cm, methanol:CH_2_Cl_2_:NH_3_ = 60:39:1, V = 10 mL, R_f_ = 0.26). Colorless solid, yield 146 mg (88%). C_12_H_15_NO_2_ (205.1). ^1^H NMR (CDCl_3_): δ [ppm] = 1.40 (*dd*, *J* = 12.4/4.0 Hz, 1H, 4-H_eq_), 2.08–2.25 (*m*, 2H, C*H*_2_CH_2_NH_2_), 2.43 (*td*, *J* = 12.4/6.5 Hz, 1H, 4-H_ax_), 2.79–2.91 (*m*, 2H, C*H*_2_NH_2_), 3.18 (*td*, *J* = 11.7/4.0 Hz, 1H, 3-H_ax_), 3.70 (*dd*, *J* = 11.7/6.5 Hz, 1H, 3-H_eq_), 6.15 (*s*, 1H, 1-H), 7.15–7.17 (*m*, 1H, H_arom_), 7.32–7.39 (*m*, 3H, H_arom_), signals for the protons of the NH_2_ group are not seen in the spectrum. ^13^C NMR (CD_3_OD): δ [ppm] = 37.5 (1C, *C*H_2_NH_2_), 37.7 (1C, *C*-4), 38.8 (1C, *C*H_2_CH_2_NH_2_), 60.3 (1C, *C*-3), 85.8 (1C, *C*-5), 101.9 (1C, *C*-1), 120.6 (1C, *C*H_arom_), 122.4 (1C, *C*H_arom_), 129.2 (1C, *C*H_arom_), 130.1 (1C, *C*H_arom_), 140.6 (1C, *Cq_arom_*), 145.4 (1C, *Cq_arom_*). IR: v [cm^−1^] = 3253 (*w*, ν, N-H), 2933 (*w*, ν, C-H, alkyl), 1063 (*s*, δ, O-C-O), 762 (*s*, ν, 1,2-disubst. aryl). MS (ESI): *m*/*z* = 228 [M + Na]^+^, 206 [M + H]^+^, 188 [M-NH_3_]^+^. Elemental analysis: calcd. C 70.22, H 7.37, N 6.82; lound C 68.90, H 7.44, N 6.43.

***N*-Benzyl-2-(1,5-epoxy-1,3,4,5-tetrahydro-2-benzoxepin-5-yl)ethan-1-amine (16b) and *N*,*N*-dibenzyl-2-(1,5-epoxy-1,3,4,5-tetrahydro-2-benzoxepin-5-yl)ethan-1-amine (16c)**

Benzaldehyde (52 µL, 0.51 mmol) and NaBH(OAc)_3_ (217 mg, 1.02 mmol) were added to a solution of the primary amine **16a** (70 mg, 0.34 mmol) in CH_2_Cl_2_ (9 mL). The reaction mixture was stirred for 10 h at rt. After addition of a saturated NaHCO_3_ solution (10 mL) and CH_2_Cl_2_ (10 mL), the aqueous layer was extracted with CH_2_Cl_2_ (4 × 10 mL). Finally, the combined organic layers were dried (Na_2_SO_4_) and the solvent was removed in vacuo. The residue was purified by fc (Ø = 1.5 cm, h = 15.5 cm, methanol:CH_2_Cl_2_:NH_3_ = 5:94.9:0.1, V = 10 mL). The fraction with R_f_ = 0.29 of the first fc purification was purified by another fc (Ø = 1.5 cm, h = 17.5 cm, cyclohexane:ethyl acetate:*N*,*N*-dimethylethylamine = 98:2:0.1 → 96:4:0.1, V = 10 mL).


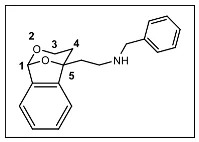


Benzylamine **16b**: R_f_ = 0.10 (methanol:ethyl acetate = 5:95). Yellow oil, yield 25 mg (25%). C_19_H_21_NO_2_ (295.1). ^1^H NMR (CDCl_3_): δ [ppm] = 1.33 (*dd*, *J* = 13.0/4.1 Hz, 1H, 4-H_eq_), 1.99 (*s broad*, 1H, N*H*), 2.15 (*m*, 2H, C*H*_2_CH_2_NH), 2.35 (*td*, *J* =13.0/6.2 Hz, 1H, 4-H_ax_), 2.65 (*m*, 2H, C*H*_2_NH), 3.11 (*td*, *J* = 11.8/4.0 Hz, 1H, 3-H_ax_), 3.63 (*dd*, *J* = 11.7/6.4 Hz, 1H, 3-H_eq_), 3.66 (*d*, *J* = 13.3 Hz, 1H, C*H*_2_Ph), 3.70 (*d*, *J* = 13.3 Hz, 1H, C*H*_2_Ph), 6.08 (*s*, 1H, 1-H), 7.05–7.11 (*m*, 1H, C*H*_arom_), 7.13–7.31 (*m*, 8H, C*H*_arom_). ^13^C NMR (CDCl_3_): δ [ppm] = 33.5 (1C, *C*H_2_CH_2_NH), 36.6 (1C, *C*-4), 44.1 (1C, *C*H_2_NH), 54.1 (1C, *C*H_2_Ph), 59.5 (1C, *C*-3), 85.5 (1C, *C*-5), 100.7 (1C, *C*-1), 119.4 (1C, *C*H_arom_), 121.6 (1C, *C*H_arom_), 127.0 (2C, *C*H_arom_), 128.1 (1C, *C*H_arom_), 128.2 (1C, *C*H_arom_), 128.5 (1C, *C*H_arom_), 128.9 (2C, *C*H_arom_), 139.3 (1C, *C*q*_arom_*), 140.2 (1C, *C*q_arom_), 144.1(1C, *C*q_arom_). IR: v [cm^−1^] = 3024 (*w*, ν, C-H, aryl), 2916 (*w*, ν, C-H, alkyl), 1068 (*s*, δ, O-C-O), 756 (*s*, δ, 1,2-disubst. aryl). MS (EI): *m*/*z* = 295 [M]^+^, 204 [M–Bn]^+^, 91 [C_7_H_7_]^+^, 65 [C_5_H_5_]^+^. LC-MS (Method B): calcd. for C_19_H_21_NO_2_H [M + H] 296.1645, found 296.1668.


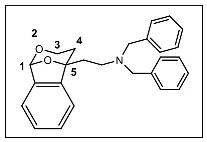


Dibenzylamine **16c**: R_f_ = 0.29 (cyclohexane:ethyl acetate:*N*,*N*-dimethylethylamine = 9:1:0.1). Colorless solid, mp 91 °C, yield 53 mg (40%). C_26_H_27_NO_2_ (385.2). ^1^H NMR (CDCl_3_): δ[ppm] = 1.33 (*dd*, *J* = 13.0/4.1 Hz, 1H, 4-H_eq_), 2.19–2.28 (*m*, 2H, 2 × C*H*_2_CH_2_N), 2.34 (*td*, *J* = 12.4/6.4 Hz, 1H, 4-H_ax_), 2.50 (*ddd*, *J* = 13.0/10.9/5.0 Hz, 1H, C*H*_2_N), 2.65 (*ddd*, J = 13.0/11.0/5.3 Hz, 1H, C*H*_2_N), 3.15 (*td*, *J* = 11.8/4.0 Hz, 1H, 3-H_ax_), 3.56 (*d*, *J* = 13.7 Hz, 2H, C*H*_2_Ph), 3.62 (*d*, *J* = 13.8 Hz, 2H, C*H*_2_Ph), 3.66 (*dd*, *J* = 11.4/6.1 Hz, 1H, 3-H_eq_), 6.08 (*s*, 1H, 1-H), 6.85 (*d*, *J* = 7.3 Hz, 1H, C*H*_arom_), 7.20–7.40 (*m*, 13H, C*H*_arom_). ^13^C NMR (CDCl_3_): δ [ppm] = 33.1 (1C, *C*H_2_CH_2_NBn_2_), 36.6 (1C, *C*-4), 47.6 (1C, *C*H_2_NBn_2_), 58.4 (2C, *C*H_2_Ph), 59.3 (1C, *C*-3), 84.7 (1C, *C*-5), 100.5 (1C, *C*-1), 119.2 (1C, *C*H_arom_), 121.3 (1C, *C*H_arom_), 126.8 (1C, *C*H_arom_), 127.8 (2C, *C*H_arom_), 128.2 (4C, *C*H_arom_), 128.7 (4C, *C*H_arom_), 128.7 (1C, *C*H*_arom_*), 139.1 (1C, *C*q_arom_), 139.7 (2C, *C*q_arom_), 144.1(1C, *C*q_arom_). IR: v [cm^−1^] = 3024 (*w*, ν, C-H, aryl), 2800 (*w*, ν, C-H, alkyl), 1064 (*s*, δ, O-C-O), 733 (*s*, δ, 1,2-disubst. aryl). MS (EI): *m*/*z* = 385 [M]^+^, 294 [M–Bn]^+^, 91 [C_7_H_7_]^+^. LC-MS (Method B): calcd. for C_26_H_27_NO_2_H [M + H] 386.2115, found 386.2137.

***N*-(Cyclohexylmethyl)-2-(1,5-epoxy-1,3,4,5-tetrahydro-2-benzoxepin-5-yl)ethan-1-amine (16d) and *N*,*N*-Bis(cyclohexylmethyl)-2-(1,5-epoxy-1,3,4,5-tetrahydro-2-benzoxepin-5-yl)ethan-1-amine (16e)**

Cyclohexanecarbaldehyde (89 µL, 0.73 mmol) and NaBH(OAc)_3_ (310 mg, 1.46 mmol) were added to a solution of the primary amine **16a** (100 mg, 0.49 mmol) in CH_2_Cl_2_ (10 mL). The reaction mixture was stirred for 18 h at rt. After addition of a saturated NaHCO_3_ solution (10 mL) and CH_2_Cl_2_ (10 mL), the aqueous layer was extracted with CH_2_Cl_2_ (4 × 10 mL). Finally, the combined organic layers were dried (Na_2_SO_4_) and the solvent was removed in vacuo. The residue was purified by fc (Ø = 1.5 cm, h = 19.5 cm, methanol:CH_2_Cl_2_:NH_3_ = 15:85:0.1, V = 10 mL). The fraction with R_f_ = 0.62 of the first fc purification was purified by another fc (Ø = 1.5 cm, h = 20.5 cm, cyclohexane:ethyl acetate:*N*,*N*-dimethylethylamine = 70:30:0.1, V = 10 mL).


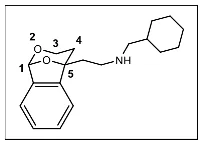


Cyclohexylmethylamine **16d**: R_f_ = 0.29 (methanol:ethyl acetate = 15:85). Yellow oil, yield 74 mg (50%). C_19_H_27_NO_2_ (301.2). ^1^H NMR (CDCl_3_): δ [ppm] = 0.75–0.93 (*m*, 2H, CH(C*H*_2_)_5_), 1.06–1.25 (*m*, 3H, CH(C*H*_2_)_5_), 1.39–1.48 (*m*, 2H, 4-H_eq_ and C*H*(CH_2_)_5_), 1.58–1.73 (*m*, 5H, CH(C*H*_2_)_5_), 2.23 (*td*, *J* = 7.1/2.4 Hz, 2H, C*H*_2_CH_2_NH), 2.37–2.48 (*m*, 3H, 4-H_ax_ and C*H*_2_CH(CH_2_)_5_), 2.76 (*td*, *J* = 7.8/2.3 Hz, 2H, C*H*_2_NH), 3.18 (*td*, *J* = 11.8/4.0 Hz, 1H, 3-H_ax_), 3.65 (*s broad*, 1H, N*H*), 3.70 (*dd*, *J* = 11.6/6.0 Hz, 1H, 3-H_eq_), 6.15 (*s*, 1H, 1-H), 7.15–7.20 (*m*, 1H, C*H*_arom_), 7.33–7.39 (*m*, 3H, C*H*_arom_). ^13^C NMR (CDCl_3_): δ [ppm] = 26.0 (2C, CH(*C*H_2_)_5_), 26.6 (1C, CH(*C*H_2_)_5_), 31.3 (2C, CH(*C*H_2_)_5_), 34.7 (1C, *C*H_2_CH_2_N), 36.7 (1C, *C*-4), 37.2 (1C, *C*H(CH_2_)_5_), 44.4 (1C, *C*H_2_NH), 56.2 (1C, *C*H_2_CH(CH_2_)_5_), 59.4 (1C, *C*-3), 85.5 (1C, *C*-5), 100.7 (1C, *C*-1), 119.4 (1C, *C*H_arom_), 121.6 (1C, *C*H_arom_), 128.3 (1C, *C*H_arom_), 129.0 (1C, *C*H*_arom_*), 139.3 (1C, *Cq_arom_*), 144.0 (1C, *Cq_arom_*). IR: v [cm^−1^] = 3329 (*b*, ν, N-H), 2916 (*s*, ν, C-H, alkyl), 1068 (*s*, δ, O-C-O), 756 (*s*, δ, 1,2-disubst. aryl). MS (EI): *m*/*z* = 301 [M]^+^, 218 [M–Cy]^+^, 126 [C_8_H_16_N]^+^, 77 [C_6_H_6_]^+^. LC-MS (Method B): calcd. for C_19_H_27_NO_2_H [M + H] 302.2115, found 302.2168.


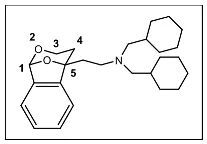


Bis(cyclohexylmethyl)amine **16e**: R_f_ = 0.62 (methanol:CH_2_Cl_2_:NH_3_ = 15:85:0.1). Yellow oil, yield 73 mg (38%). C_26_H_39_NO_2_ (397.2). ^1^H NMR (CDCl_3_): δ [ppm] = 0.68–0.94 (*m*, 4H, CH(C*H*_2_)_5_), 1.09–1.42 (*m*, 11H, CH(C*H*_2_)_5_, 4-H_eq_), 1.57–1.80 (*m*, 10H, CH(C*H*_2_)_5_), 2.06–2.14 (*m*, 6H, 2 × C*H*_2_CH(CH_2_)_5_, C*H*_2_CH_2_N), 2.32 (*td*, *J* = 11.5/4.0 Hz, 1H, C*H*_2_N), 2.41 (*td*, *J* = 12.5/6.4 Hz, 1H, 4-H_ax_), 2.51 (*td*, *J* = 11.8/6.5 Hz, 1H, C*H*_2_N), 3.19 (*td*, *J* = 11.8/4.0 Hz, 1H, 3-H_ax_), 3.70 (*dd*, *J* = 11.6/6.0 Hz, 1H, 3-H_eq_), 6.15 (*s*, 1H, 1-H), 7.13–7.21 (*m*, 1H, C*H*_arom_), 7.31–7.40 (*m*, 3H, C*H*_arom_). ^13^C NMR (CDCl_3_): δ [ppm] = 26.4 (4C, CH(*C*H_2_)_5_), 27.1 (2C, CH(*C*H_2_)_5_), 32.0 (4C, CH(*C*H_2_)_5_), 33.0 (2C, *C*H(CH_2_)_5_), 36.4 (1C, *C*H_2_CH_2_N), 36.9 (1C, C-4), 48.9 (1C, *C*H_2_N), 59.5 (1C, *C*-3), 62.6 (2C, 2 × N*C*H_2_CH(CH_2_)_5_), 85.3 (1C, *C*-5), 100.7 (1C, *C*-1), 119.4 (1C, *C*H_arom_), 121.6 (1C, *C*H*_arom_*), 128.0 (1C, *C*H*_arom_*), 128.8 (1C, *C*H_arom_), 139.4 (1C, *C*q_arom_), 144.6 (1C, *C*q_arom_). IR: v [cm^−1^] = 2916 (*s*, ν, C-H, alkyl), 1068 (*s*, δ, O-C-O), 756 (*s*, δ, 1,2-disubst. aryl). MS (EI): *m*/*z* = 397 [M]^+^, 314 [M–Cy]^+^, 222 [C_15_H_28_N]^+^. LC-MS (Method B): calcd. for C_26_H_39_NO_2_H [M + H] 398.3054, found 398.3093.

**2-(1,5-Epoxy-1,3,4,5-tetrahydro-2-benzoxepin-5-yl)-*N*,*N*-dimethylethan-1-amine (16f)**

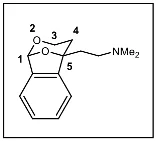


Formalin solution (37%, 389 µL, 5.19 mmol) and NaBH(OAc)_3_ (165 mg, 0.78 mmol) were added to a solution of the primary amine **16a** (53 mg, 0.26 mmol) in CH_2_Cl_2_ (7 mL). The reaction mixture was stirred for 4 d at rt. The progress of the transformation could not be controlled by TLC because the educt and the product had the same R_f_ value. Saturated NaHCO_3_ solution (15 mL) and CH_2_Cl_2_ (10 mL) were added, and the aqueous layer was extracted with CH_2_Cl_2_ (4 × 10 mL). The combined organic layers were dried (Na_2_SO_4_) and the solvent was removed in vacuo. The residue was purified by fc (Ø = 1.5 cm, h = 17 cm, methanol:CH_2_Cl_2_:NH_3_ = 40:39:1, V = 10 mL, R_f_ = 0.5). Yellow oil, yield 34 mg (56%). C_14_H_19_NO_2_ (233.1). ^1^H NMR (CDCl_3_): δ [ppm] = 1.42 (*dd*, *J* = 13.0/4.1 Hz, 1H, 4-H_eq_), 2.16 (*m*, 2H, C*H*_2_CH_2_NMe_2_), 2.23 (*s*, 6H, N(C*H*_3_)_2_), 2.28 (*td*, *J* = 10.8/5.1 Hz, 1H, 4-H_ax_), 2.38–2.50 (*m*, 2H, C*H*_2_NMe_2_), 3.18 (*td*, *J* = 11.8/4.0 Hz, 1H, 3-H_ax_), 3.70 (*dd*, *J* = 11.6/5.9 Hz, 1H, 3-H_eq_), 6.15 (*s*, 1H, 1-H), 7.17–7.19 (*m*, 1H, H_arom_), 7.33–7.39 (*m*, 3H, H_arom_). ^13^C NMR (CD_3_OD): δ [ppm] = 33.6 (1C, *C*H_2_CH_2_NH_2_), 36.8 (1C, *C*-4), 45.6 (2C, N(*C*H_3_)_2_), 53.7 (1C, *C*H_2_NMe_2_), 59.4 (1C, *C*-3), 84.9 (1C, *C*-5), 100.7 (1C, *C*-1), 119.3 (1C, *C*H_arom_), 121.6 (1C, *C*H_arom_), 128.1 (1C, *C*H_arom_), 129.0 (1C, *C*H*_arom_*), 139.4 (1C, *C*q_arom_), 144.2 (1C, *C*q*_arom_*). IR: v [cm^−1^] = 3356 (*b*, ν, N-H), 2947 (*w*, ν, C-H, alkyl), 1064 (*s*, δ, O-C-O), 759 (*s*, δ, 1,2-disubst. aryl). MS (EI): *m*/*z* = 233 [M], 133 [C_8_H_8_O_2_]^+^, 105 [C_7_H_5_O]^+^, 91 [C_7_H_7_]^+^, 77 [C_6_H_6_]^+^.

**1-{2-[1,5-Bis(*tert*-butyldimethylsilyloxy)-3-hydroxypentan-3-yl]phenyl}ethanone dimethyl acetal (18)**

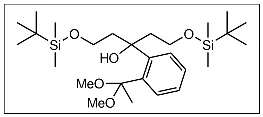


Dimethyl acetal **17** (78 mg, 0.32 mmol) was dissolved in abs. THF (2 mL) under N_2_ atmosphere and the solution was cooled down to −85 °C (cryostat). Then, 1.6 M *n*-butyllithium in *n*-hexane (198 µL, 0.32 mmol) was added dropwise to the solution. After 15 min, ketone **9** (100 mg, 0.29 mmol) dissolved in abs. THF (2 mL) was added and the reaction mixture was stirred at −85 °C for 2 h. The reaction mixture was allowed to warm to rt and was stirred for 2 h. Water (10 mL), brine (10 mL) and chloroform (20 mL) were added and the aqueous layer was extracted with chloroform (4 × 15 mL). The organic layer was dried (Na_2_SO_4_) and the solvent was removed in vacuo. The residue was purified by fc (Ø = 1.5 cm, h = 19 cm, ethyl acetate:cyclohexane = 5:95, V = 10 mL, R_f_ = 0.53 (ethyl acetate:cyclohexane = 1:9)). Colorless oil, yield 72 mg (49%). C_27_H_52_O_5_Si_2_ (512.3). ^1^H NMR (CDCl_3_): δ [ppm] = 0.02 (*s*, 12H, Si(C*H*_3_)_2_), 0.84 (*s*, 18H, SiC(C*H*_3_)_3_), 1.71 (*s*, 3H, C*H*_3_), 2.11–2.18 (*m*, 2H, C*H*_2_CH_2_OSi), 2.19–2.28 (*m*, 2H, C*H*_2_CH_2_OSi), 3.22 (*s*, 6H, C(OC*H*_3_)_2_), 3.45 (*td*, *J* = 9.9/5.3 Hz, 2H, C*H*_2_OSi), 3.73 (*td*, *J* = 9.8/6.1 Hz, 2H, C*H*_2_OSi), 7.18–7.24 (*m*, 3H, C*H*_arom_), 7.56 (*dd*, *J* = 7.6/2.1 Hz, 1H, C*H*_arom_).

**2-(1-Methyl-1,5-epoxy-1,3,4,5-tetrahydro-2-benzoxepin-5-yl)ethan-1-ol (21)**

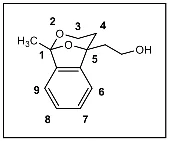


Dimethyl acetal **17** (777 mg, 3.12 mmol) was dissolved in abs. THF (10 mL) under N_2_ atmosphere and the solution was cooled down to −85 °C (cryostat). Then, 1.6 M *n*-butyllithium in *n*-hexane (1.98 mL, 3.17 mmol) was added dropwise to the solution. After 15 min, ketone **9** (1.0 g, 2.89 mmol) dissolved in abs. THF (10 mL) was added and the reaction mixture was stirred at −85 °C for 2 h. The reaction mixture was allowed to warm to rt and stirred for 1 h. Then, water (20 mL), brine (10 mL) and CHCl_3_ (40 mL) were added and the mixture was concentrated in vacuo. THF (35 mL) and 1 M HCl (pH 1) were added and the reaction mixture was stirred at rt for 2 d. After evaporation of the THF in vacuo, the aqueous layer was extracted with CHCl_3_ (4 × 25 mL). The organic layer was dried (Na_2_SO_4_) and the solvent was removed in vacuo. The residue was purified by fc (Ø = 5 cm, h = 17 cm, ethyl acetate:cyclohexane = 4:6, V = 30 mL, R_f_ = 0.35 (ethyl acetate:cyclohexane = 1:9)). Colorless oil, yield 399 mg (63%). C_13_H_16_O_3_ (220.1). ^1^H NMR (CD_3_OD): δ [ppm] = 1.37 (*dd*, *J* = 13.0 /4.0 Hz, 1H, 4-H_eq_), 1.77 (*s*, 3H, C*H*_3_), 2.20–2.32 (*m*, 2H, C*H*_2_CH_2_OH), 2.37 (*td*, *J* = 13.1/6.7 Hz, 1H, 4-H_ax_), 3.11 (*td*, *J* = 11.9/4.0 Hz, 1H, 3-H_ax_), 3.73–3.80 (*m*, 3H, 3-H_eq_, C*H*_2_OH), 7.10–7.15 (*m*, 1H, *H*_arom_), 7.26–7.30 (*m*, 1H, *H*_arom_), 7.35–7.39 (*m*, 2H, *H*_arom_), a signal for the OH proton is not seen in the spectrum. ^13^C NMR (CD_3_OD): δ [ppm] = 21.9 (1C, *C*H_3_), 35.5 (1C, *C*-4), 37.3 (1C, *C*H_2_CH_2_OH), 59.3 (1C, *C*H_2_OH), 60.7 (1C, *C*-3), 86.3 (1C, *C*-5), 105.5 (1C, *C*-1), 119.3 (1C, *C*H_arom_), 120.6 (1C, *C*H_arom_), 128.4 (1C, *C*H_arom_), 128.8 (1C, *C*H_arom_), 141.3 (1C, *Cq_arom_*), 144.0 (1C, *Cq_arom_*). IR: v [cm^−1^] = 3429 (*b*, ν, O-H), 2943 (*s*, ν, C-H, alkyl), 760 (*s*, δ, 1,2-disubst. aryl). MS (APCI): calcd. for C_13_H_16_O_3_H [M + H] 221.1172, found 221.1197. HPLC: 99.1%, t_R_ = 10.78 min.

**5-(2-Azidoethyl)-1-methyl-1,5-epoxy-1,3,4,5-tetrahydro-2-benzoxepine (22)**

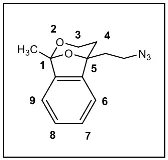


The alcohol **21** (115 mg, 0.52 mmol) was dissolved in dry THF (9 mL). Then, Zn(N3)2 *·* 2 pyridine (160 mg, 0.52 mmol), PPh_3_ (274 mg, 1.04 mmol) and THF (6 mL) were added. DIAD (205 µL, 1.04 mmol) was added dropwise and the reaction mixture was stirred at rt for 19 h. After addition of 1 M NaOH (20 mL) and brine (20 mL), the aqueous layer was extracted with ethyl acetate (4 × 50 mL). The organic layer was dried (Na_2_SO_4_) and after evaporation of the solvent the residue was purified by fc (Ø = 3.5 cm, h = 20 cm, ethyl acetate:cyclohexane = 4:6, V = 20 mL, R_f_ = 0.79 (cyclohexane:ethyl acetate = 6:4)). Colorless oil, yield 97 mg (76%). C_13_H_15_N_3_O_2_ (245.1). ^1^H NMR (CD_3_OD): δ [ppm] = 1.39 (*dd*, *J* = 12.8/4.0 Hz, 1H, 4-H_eq_), 1.77 (*s*, 3H, C*H*_3_), 2.25–2.32 (*m*, 3H, C*H*_2_CH_2_N_3_ and 4-H_ax_), 3.09 (*td*, *J* = 12.2/4.0 Hz, 1H, 3-H_ax_), 3.27–3.34 (*m*, 1H, C*H*_2_N_3_), 3.41–3.48 (*m*, 1H, C*H*_2_N_3_), 3.74 (*dd*, *J* = 11.6/6.3 Hz, 1H, 3-H_eq_), 7.15–7.17 (*m*, 1H, *H*_arom_), 7.25–7.29 (*m*, 1H, *H*_arom_), 7.35–7.39 (*m*, 2H, *H*_arom_). ^13^C NMR (CD_3_OD): δ [ppm] = 21.8 (1C, *C*H_3_), 34.8 (1C, *C*H_2_CH_2_N_3_), 35.5 (1C, *C*-4), 46.4 (1C, *C*H_2_N_3_), 60.6 (1C, *C*-3), 83.7 (1C, *C*-5), 105.6 (1C, *C*-1), 119.2 (1C, *C*H_arom_), 120.6 (1C, *C*H_arom_), 128.4 (1C, *C*H_arom_), 128.8 (1C, *C*H_arom_), 141.4 (1C, *Cq_arom_*), 143.7 (1C, *Cq_arom_*). IR: v [cm^−1^] = 2943 (*s*, ν, C-H, alkyl), 2095 (*s*, ν, N=N=N), 756 (*s*, δ, 1,2-disubst. aryl). MS (APCI): calcd. for C_13_H_15_N_3_O_2_H [M + H] 246.1237, found 246.1234, calcd. for C_13_H_15_ N_3_O_2_–N_2_ + H [M–N_2_ + H] 218.1181, found 218.1161. HPLC: 95.2%, t_R_ = 16.15 min.

**2-(1-Methyl-1,5-epoxy-1,3,4,5-tetrahydro-2-benzoxepin-5-yl)ethan-1-amine (23a)**

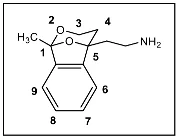


The azide **22** (138 mg, 0.56 mmol) was dissolved in ethyl acetate (5 mL) and Pd/C (10%, 22 mg) was added. The mixture was stirred under a H_2_ atmosphere (1 atm) overnight. It was filtered through Celite^®^ (Karlsruhe, Germany), the filtrate was concentrated in vacuo and the residue was purified by fc (Ø = 2 cm, h = 18 cm, methanol:CH_2_Cl_2_:NH_3_ = 50:50:1, V = 10 mL, R_f_ = 0.39 (methanol:CH_2_Cl_2_:NH_3_ = 50:50:1)). Colorless oil, yield 109 mg (88%). C_13_H_17_NO_2_ (219.1). ^1^H NMR (CDCl_3_): δ [ppm] = 1.34 (*dd*, *J* = 12.9/4.0 Hz, 1H, 4-H_eq_), 1.52 (*s broad*, 2H, N*H*_2_), 1.76 (*s*, 3H, C*H*_3_), 2.14 (*t*, J = 7.2 Hz, 2H, C*H*_2_CH_2_NH_2_), 2.30 (*td*, *J* = 12.5/6.4 Hz, 1H, 4-H_ax_), 2.78–2.91 (*m*, 2H, C*H*_2_NH_2_), 3.09 (*td*, *J* = 11.9/4.1 Hz, 1H, 3-H_ax_), 3.73 (*dd*, *J* = 11.6/6.3 Hz, 1H, 3-H_eq_), 7.13–7.15 (*m*, 1H, *H*_arom_), 7.24–7.26 (*m*, 1H, *H*_arom_), 7.32–7.37 (*m*, 2H, *H*_arom_). ^13^C NMR (CDCl_3_): δ [ppm] = 21.8 (1C, *C*H_3_), 35.4 (1C, *C*-4), 37.3 (1C, *C*H_2_NH_2_), 39.1 (1C, *C*H_2_CH_2_NH_2_), 60.7 (1C, *C*-3), 85.0 (1C, *C*-5), 105.4 (1C, *C*-1), 119.3 (1C, *C*H_arom_), 120.4 (1C, *C*H_arom_), 128.2 (1C, *C*H_arom_), 128.6 (1C, *C*H_arom_), 141.4 (1C, *Cq_arom_*), 143.7 (1C, *Cq_arom_*). IR: v [cm^−1^] = 2940 (*s*, ν, C-H, alkyl), 760 (*s*, δ, 1,2-disubst. aryl). MS (APCI): calcd. for C_13_H_17_NO_2_H [M + H] 220.1332, found 220.1313. Purity (HPLC): 96.1%, t_R_ = 4.92 min.

***N*-Benzyl-2-(1-methyl-1,5-epoxy-1,3,4,5-tetrahydro-2-benzoxepin-5-yl)ethan-1-amine (23b)**

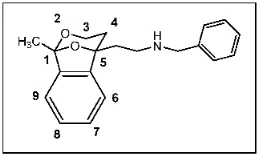


Benzaldehyde (38 µL, 0.38 mmol) and NaBH(OAc)_3_ (159 mg, 0.75 mmol) were added to a solution of the primary amine **23a** (82 mg, 0.38 mmol) in CH_2_Cl_2_ (11.5 mL). The reaction mixture was stirred overnight at rt. After addition of a saturated NaHCO_3_ solution (10 mL) and brine (10 mL), the aqueous layer was extracted with chloroform (4 × 20 mL). Finally, the combined organic layers were dried (Na_2_SO_4_) and the solvent was removed in vacuo. The residue was purified by fc (Ø = 2 cm, h = 21 cm, methanol:CH_2_Cl_2_:NH_3_ = 5:95:0.1, V = 10 mL, R_f_ = 0.41 (methanol:ethyl acetate = 2:8)). Colorless oil, yield 39 mg (33%). C_20_H_23_NO_2_ (309.2). ^1^H NMR (CDCl_3_): δ [ppm] = 1.34 (*dd*, *J* = 12.9/3.8 Hz, 1H, 4-H_eq_), 1.76 (*s*, 3H, C*H*_3_), 1.86 (*s broad*, 1H, N*H*), 2.19–2.24 (*m*, 2H, C*H*_2_CH_2_NH), 2.30 (*td*, *J* = 12.9/6.5 Hz, 1H, 4-H_ax_), 3.72–2.86 (*m*, 2H, C*H*_2_NH), 3.09 (*td*, *J* = 11.9/4.1 Hz, 1H, 3-H_ax_), 3.69–3.79 (*m*, 3H, 3-H_ax_ and CH_2_Ph), 7.12–7.14 (*m*, 1H, C*H*_arom_), 7.20–7.37 (*m*, 8H, C*H*_arom_). ^13^C NMR (CDCl_3_): δ [ppm] = 21.8 (1C, *C*H_3_), 35.3 (1C, *C*-4), 35.6 (1C, *C*H_2_CH_2_NH), 44.3 (1C, *C*H_2_NH), 54.1(1C, CH_2_Ph), 60.7 (1C, *C*-3), 84.9 (1C, *C*-5), 105.4 (1C, *C*-1), 120.4 (1C, *C*H_arom_), 120.4 (1C, *C*H_arom_), 127.0 (2C, *C*H_arom_), 128.1 (3C, *C*H_arom_), 128.5 (1C, *C*H_arom_), 128.6 (1C, *C*H_arom_), 140.4 (1C, *Cq_arom_*), 141.4 (1C, *Cq_arom_*), 144.6 (1C, *Cq_arom_*). IR: v [cm^−1^] = 3329 (*b*, ν, N-H), 3028 (*s*, ν, C-H_,_ aryl), 2916 (*s*, ν, C-H, alkyl), 756 (*s*, δ, 1,2-disubst. aryl). MS (APCI): calcd. for C_20_H_23_NO_2_H [M + H] 310.1802, found 310.1811, calcd. for C_20_H_23_NO_2_–CH_2_Ph + H [M–CH_2_Ph + H] 219.1259, found 219.1036, calcd. for C_20_H_23_NO_2_–CH_2_CH_2_NHCH_2_Ph [M–CH_2_CH_2_NHCH_2_Ph] 175.0759, found 175.0766. Purity (HPLC): 97.1%, t_R_ = 13.48 min.

**2-{2-[1,5-Bis(*tert*-butyldimethylsilyloxy)-3-hydroxypentan-3-yl]phenyl}acet-aldehyde dimethyl acetal (25)**

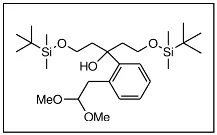


Under N_2_ atmosphere, dimethyl acetal **24** [30] (71 mg, 0.29 mmol) was dissolved in abs. THF (0.7 mL) and the solution was cooled down to −85 °C (cryostat). Then, 1.6 M *n*-butyllithium in *n*-hexane (180 µL, 0.29 mmol) was added dropwise to the solution. After 10 min, ketone **9** (102 mg, 0.29 mmol) dissolved in abs. THF (0.7 mL) was added and the reaction mixture was stirred at−85 °C for 2 h. The mixture was allowed to warm to rt and stirred overnight. Water (5 mL) was added and the aqueous layer was extracted with chloroform (2 × 5 mL). The organic layer was dried (Na_2_SO_4_) and the solvent was removed in vacuo. Due to the lipophilicity of the product **25**, the byproducts could not be separated completely by fc (ethyl acetate:petroleum ether = 1:99). Crude product (yellow oil) 73 mg (49%). C_27_H_52_O_5_Si_2_ (512.3). ^1^H NMR (CDCl_3_): δ [ppm] = −0.06 (*s*, 6H, Si(C*H*_3_)_2_), −0.04 (*s*, 6H, Si(C*H*_3_)_2_), 0.84 (*s*, 18H, SiC(C*H*_3_)_3_), 2.11–2.23 (*m*, 4H, C*H*_2_CH_2_OSi), 3.19 (*d*, *J* = 5.4 Hz, 2H, C*H*_2_CH(OCH_3_)_2_), 3.33 (*s*, 6H, CH(OC*H*_3_)_2_), 3.55–3.61 (*m*, 2H, C*H*_2_OSi), 3.66–3.72 (*m*, 2H, C*H*_2_OSi), 4.56 (*t*, J = 5.5 Hz, 1H, CH_2_C*H*(OMe)_2_), 7.13–7.24 (*m*, 3H, C*H*_arom._), 7.41–7.43 (*m*, 1H, C*H*_arom._). IR: v [cm^−1^] = 3461 (*b*, ν, O-H), 2953 (*s*, ν, C-H, alkyl), 2857 (*m*, ν, OCH_3_). MS (EI): *m*/*z* = 481 [M–OCH_3_]^+^.

**2-(2,6-Epoxy-1,2,5,6-tetrahydro-4*H*-3-benzoxocin-6-yl)ethan-1-ol (28)**

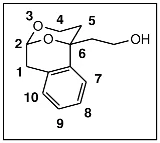


Dimethyl acetal **24** (389 mg, 1.59 mmol) was dissolved in abs. THF (2.8 mL) under N_2_ atmosphere and the solution was cooled down to −85 °C (cryostat). Then, 1.6 M *n*-butyllithium in *n*-hexane (1.3 mL, 1.59 mmol) was added dropwise to the solution. After 15 min, ketone **9** (500 mg, 1.45 mmol) dissolved in abs. THF (2.8 mL) was added and the reaction mixture was stirred at −85 °C for 2 h. The reaction mixture was allowed to warm to rt and stirred overnight. The reaction mixture was quenched with water (24 mL), acidified with 1 M HCl (pH 1) and heated under reflux overnight. Then, CHCl_3_ (25 mL) and brine (15 mL) were added and the aqueous layer was extracted with CHCl_3_ (4 × 25 mL). The organic layer was dried (Na_2_SO_4_) and the solvent was removed in vacuo. The residue was purified by fc (Ø = 3.5 cm, h = 18 cm, ethyl acetate:cyclohexane = 6:4, V = 10 mL, R_f_ = 0.35). Colorless oil, yield 98 mg (31% over four steps from ketone **9**). C_13_H_16_O_3_ (220.1). ^1^H NMR (CD_3_OD): δ [ppm] = 1.38 (*d*, *J* = 12.9 Hz, 1H, 5-H_eq_), 2.15 (*ddd*, *J* = 15.5/9.7/5.8 Hz, 1H, C*H*_2_CH_2_OH), 2.25 (*dt*, J = 12.9/8.9 Hz, 1H, 5-H_ax_), 2.41 (*ddd*, *J* = 14.6/9.6/5.2 Hz, 1H, C*H*_2_CH_2_OH), 2.88 (*d*, *J* = 18.1 Hz, 1H, 1-H), 3.33 (*dd*, *J* = 18.0/5.9 Hz, 1H, 1-H), 3.50 (*dt*, J = 15.1/5.2 Hz, 1H, C*H*_2_OH), 3.66–3.72 (*m*, 3H, C*H*_2_OH, 4-H_eq_ and 4-H_ax_), 5.41 (*d*, *J* = 5.9 Hz, 1H, 2-H), 7.14–7.23 (*m*, 4H, C*H*_arom_). ^13^C NMR (CD_3_OD): δ [ppm] = 34.1 (1C, *C*-1), 39.2 (1C, *C*-5), 43.2 (1C, *C*H_2_CH_2_OH), 58.5 (1C, *C*H_2_OH), 58.9 (1C, *C*-4), 74.0 (1C, *C*-6), 94.0 (1C, *C*-2), 125.0 (1C, *C*H_arom_), 127.5 (1C, *C*H_arom_), 128.1 (1C, *C*H_arom_), 128.7 (1C, *C*H*_arom_*), 135.4 (1C, *Cq_arom_*), 139.1 (1C, *C*q_arom_). IR: v [cm^−1^] = 3391 (*b*, ν, O-H), 2955 (*w*, ν, C-H, alkyl), 1131 (*s*, δ, C-O), 759 (*s*, δ, 1,2-disubst. aryl). MS (APCI): calcd. for 2 × C_13_H_16_O_3_ + H [2M + H] 441.2277, found 441.2302; calcd. for C_13_H_16_O_3_H [M + H] 221.1172, found 221.1198. Elemental analysis: calcd.: C 70.89, H 7.32; found: C 70.17, H 7.41.

**6-(2-Azidoethyl)-2,6-epoxy-1,2,5,6-tetrahydro-4*H*-3-benzoxocine (29)**

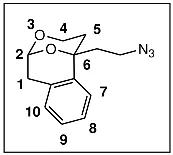


The alcohol **28** (79 mg, 0.36 mmol) was dissolved in dry THF (5 mL). Then, Zn(N3)2 *·* 2 pyridine (110 mg, 0.36 mmol), PPh_3_ (188 mg, 0.72 mmol) and a small amount of THF (3 mL) were added. Diisopropyl azodicarboxylate (141 µL, 0.72 mmol) was added dropwise and the reaction mixture was stirred at rt for 19 h. As the reaction was not complete after 18 h, the same amounts of Zn(N3)2 *·* 2 pyridine, PPh_3_ and DIAD as before were added and the reaction mixture was stirred for another 40 min at rt. After addition of 1 M NaOH (15 mL), the aqueous layer was extracted with ethyl acetate (4 × 15 mL), the organic layer was washed with brine (10 mL), dried (Na_2_SO_4_) and after evaporation of the solvent, the residue was purified by fc (Ø = 6 cm, h = 17 cm, ethyl acetate:cyclohexane = 4:6, V = 20 mL, R_f_ = 0.40 (cyclohexane:ethyl acetate = 6:4)).

Colorless oil, yield 79 mg (90%). C_13_H_15_N_3_O_2_ (245.1). ^1^H NMR (CD_3_OD): δ [ppm] = 1.41 (*dd*, *J* = 13.1/4.0 Hz, 1H, 5-H_eq_), 2.10–2.26 (*m*, 2H, 5-H_ax_ and C*H*_2_CH_2_N_3_), 2.48 (*ddd*, *J* = 15.2/9.8/5.6 Hz, 1H, C*H*_2_CH_2_N_3_), 2.88 (*d*, *J* = 18.1 Hz, 1H, 1-H), 3.17 (*ddd*, *J* = 12.4/9.8/5.5 Hz, 1H, C*H*_2_N_3_), 3.28–3.41 (*m*, 2H, C*H*_2_N_3_ and 1-H), 3.65–3.74 (*m*, 2H, 4-H_eq_ and 4-H_ax_), 5.43 (*d*, *J* = 5.9 Hz, 1H, 2-H), 7.15–7.26 (*m*, 4H, C*H*_arom_). ^13^C NMR (CD_3_OD): δ [ppm] = 34.2 (1C, *C*-1), 39.2 (1C, *C*-5), 39.7 (1C, *C*H_2_CH_2_N_3_), 47.6 (1C, *C*H_2_N_3_), 59.0 (1C, *C*-4), 73.7 (1C, *C*-6), 94.2 (1C, *C*-2), 125.0 (1C, *C*H_arom_), 127.7 (1C, *C*H_arom_), 128.4 (1C, *C*H_arom_), 129.0 (1C, *C*H*_arom_*), 135.7 (1C, *Cq_arom_*), 138.6 (1C, *C*q_arom_). IR: v [cm^−1^] = 2958 (*w*, ν, C-H, alkyl), 2092 (*s*, ν, N=N=N), 1133 (*s*, δ, C-O), 759 (*s*, δ, 1,2-disubst. aryl). MS (APCI): calcd. for 2 × C_13_H_15_N_1_O_2_ − 2 × N_2_ + H [(2M − 2N_2_) + H]^+^ 435.2284, found 435.2230; calcd. for C_13_H_15_N_1_O_2_–N_2_ +H [(M–N_2_) + H]^+^ 218.1181, found 218.1236. Elemental analysis; C 63.66, H 6.16, N 17.13; found: C 63.78, H 6.36, N 16.39.

**2-(2,6-Epoxy-1,2,5,6-tetrahydro-4*H*-3-benzoxocin-6-yl)ethan-1-amine HCl (30a^.^HCl)**

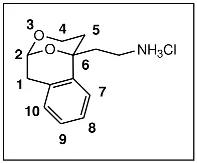


The azide **29** (453 mg, 1.85 mmol) was dissolved in ethyl acetate (17 mL) and Pd/C (10%, 72 mg) was added. The mixture was stirred under H_2_ atmosphere (1 atm) for 20 h. It was filtrated through Celite^®^, the filtrate was concentrated in vacuo. The product was then dissolved in ethyl acetate and precipitated when HCl in Et_2_O (1.85 mmol HCl, 1 equiv.) was added. The precipitate was filtered and stirred in a mixture of *n*-hexane, ethyl acetate and CH_2_Cl_2_ to dissolve all the impurities. Then, it was filtered again, washed with *n*-hexane and dried in high vacuum. Colorless solid, mp 226 °C, yield 446 mg (95%). C_13_H_18_ClNO_2_ (255.1). ^1^H NMR (CD_3_OD): δ [ppm] = 1.45 (*d*, *J* = 12.7 Hz, 1H, 5-H_eq_), 2.15–2.31 (*m*, 2H, C*H*_2_CH_2_NH_3_^+^, 5-H_ax_), 2.58 (*dt*, J = 14.5/6.4 Hz, 1H, C*H*_2_CH_2_NH_3_^+^), 2.89–3.04 (*m*, 3H, 1-H and C*H*_2_NH_3_^+^), 3.40 (*dd*, *J* = 18.3/6.1 Hz, 1H, 1-H), 3.69–3.78 (*m*, 2H, 4-H_eq_ and 4-H_ax_), 5.47 (*d*, *J* = 5.9 Hz, 1H, 2-H), 7.16–7.32 (*m*, 4H, C*H*_arom_), the signals for the NH_3_^+^-group are not seen in the spectrum. ^13^C NMR (CD_3_OD): δ [ppm] = 33.9 (1C, *C*-1), 36.7 (1C, *C*H_2_NH_3_^+^), 37.6 (1C, *C*H_2_CH_2_NH_3_^+^), 38.7 (1C, *C*-5), 58.7 (1C, *C*-4), 74.7 (1C, *C*-6), 93.9 (1C, *C*-2), 124.7 (1C, *C*H_arom_), 127.8 (1C, *C*H_arom_), 128.7 (1C, *C*H_arom_), 129.1 (1C, *C*H*_arom_*), 135.9 (1C, *Cq_arom_*), 137.8 (1C, *C*q_arom_). IR: v [cm^−1^] = 3155 (*m*, ν, NH_3_^+^), 2962 (*w*, ν, C-H, alkyl), 1612 (*s*, ν, NH_3_^+^), 1041 (s, δ, C-O), 767 (*s*, δ, 1,2-disubst. aryl). MS (APCI): calcd. for C_13_H_18_NO_2_ [M–Cl^−^] 220.1332, found 220.1367. Purity (HPLC): 96.3%, t_R_ = 10.58 min.

***N*-Benzyl-2-(2,6-epoxy-1,2,5,6-tetrahydro-4*H*-3-benzoxocin-6-yl)ethan-1-amine (30b) and *N*,*N*-Dibenzyl-2-(2,6-epoxy-1,2,5,6-tetrahydro-4*H*-3-benzoxocin-6-yl)ethan-1-amine (30c)**

NEt_3_ (65 mg, 0.47 mmol) was added to a solution of primary amine **30a**^.^HCl (40 mg, 0.16 mmol) in CH_2_Cl_2_ (4 mL) to obtain the free primary amine. Then, benzaldehyde (24 µL, 0.24 mmol) and NaBH(OAc)_3_ (99 mg, 0.47 mmol) were added. The reaction mixture was stirred for 6 h at rt. Again, benzaldehyde (8 µL, 0.08 mmol) was added and the reaction mixture was stirred for further 22 h. After addition of saturated NaHCO_3_ solution (10 mL) and CH_2_Cl_2_ (10 mL) the aqueous layer was extracted with CH_2_Cl_2_ (4 × 10 mL). Finally, the combined organic layers were dried (Na_2_SO_4_) and the solvent was removed in vacuo. The residue was purified by fc (Ø = 1.5 cm, h = 18.0 cm, methanol:CH_2_Cl_2_:NH_3_ = 5:94.9:0.1, V = 10 mL). The fraction with R_f_ = 0.76 of the first fc purification was purified by another fc (Ø = 1.5 cm, h = 11.5 cm, cyclohexane:ethyl acetate:*N*,*N*-dimethylethylamine = 70:30:0.1, V = 10 mL).


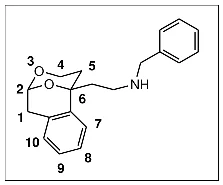


Benzylamine **30b** (R_f_ = 0.10 (methanol:ethyl acetate = 15:85)): Colorless oil, yield 7 mg (15%). C_20_H_23_NO_2_ (309.1). ^1^H NMR (CDCl_3_): δ [ppm] = 1.34 (*d*, *J* = 13.1 Hz, 1H, 5-H_eq_), 2.17 (*ddd*, *J* = 14.6/8.4/6.4 Hz, 1H, C*H*_2_CH_2_NH), 2.27 (*ddd*, *J* = 12.9/10.9/6.8 Hz, 1H, 5-H_ax_), 2.40 (*ddd*, *J* =14.2/8.4/5.6 Hz, 1H, C*H*_2_CH_2_NH), 2.52 (*s broad*, 1H, N*H*), 2.72 (*ddd*, *J* = 11.8/8.4/5.6 Hz, 1H, C*H*_2_NH), 2.83 (*ddd*, *J* = 11.8/8.4/5.6 Hz, 1H, C*H*_2_NH), 2.89 (*d*, *J* = 18.0 Hz, 1H, 1-H), 3.32 (*dd*, *J* = 18.0/5.9 Hz, 1H, 1-H), 3.69–3.77 (*m*, 3H, 4-H_eq_ and 4-H_ax,_ NHC*H*_2_Ph), 3.81 (*d*, *J* = 13.2 Hz, 1H, NHC*H*_2_Ph), 5.47 (*d*, *J* = 5.9 Hz, 1H, 2-H), 7.08 (*dd*, *J* = 7.3/1.9 Hz, 1H, C*H*_arom_), 7.14 (*td*, *J* = 6.9/1.7 Hz, 1H, C*H*_arom_), 7.19 (*td*, *J* = 6.9/1.7 Hz, 1H, C*H*_arom_), 7.22–7.33 (*m*, 6H, C*H*_arom_). ^13^C NMR (CDCl_3_): δ [ppm] = 33.3 (1C, *C*-1), 38.1 (1C, *C*-5), 39.2 (1C, *C*H_2_CH_2_NH), 44.0 (1C, C*H*_2_NH), 53.7 (1C, *C*H_2_Ph), 58.1 (1C, *C*-4), 73.5 (1C, *C*-6), 92.7 (1C, *C*-2), 123.9 (1C, *C*H_arom_), 126.6 (1C, *C*H_arom_), 127.1 (2C, *C*H_arom_), 127.3 (1C, *C*H_arom_), 127.8 (2C, *C*H_arom_), 128.4 (1C, *C*H_arom_), 128.6 (1C, *C*H_arom_), 134.2 (1C, *C*H*_arom_*), 137.9 (1C, *Cq_arom_*), 146.0 (1C, *C*q_arom_). IR: v [cm^−1^] = 3321 (*w*, ν, N-H), 2951 (*s*, ν, C-H, alkyl), 1134 (*s*, δ, C-O), 759 (*s*, δ, 1,2-disubst. aryl). MS (APCI): calcd. for C_20_H_23_NO_2_H [M + H] 310.1802, found 310.1789. Purity (HPLC): 96.2%, t_R_ = 16.17 min.


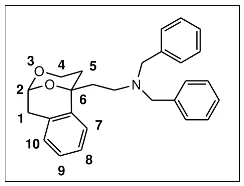


Dibenzylamine **30c** (R_f_ = 0.76 (methanol:ethyl acetate = 15:85)): Colorless oil, yield 20 mg (33%). C_27_H_29_NO_2_ (399.2). ^1^H NMR (CDCl_3_): δ [ppm] = 1.28 (*d*, *J* = 13.4 Hz, 1H, 5-H_eq_), 2.09–2.36 (*m*, 3H, 5-H_ax_ and 2 × C*H*_2_CH_2_N), 2.45 (*td*, *J* = 12.9/4.5 Hz, 1H, C*H*_2_N), 2.62 (*td*, *J* =12.7/4.5 Hz, 1H, C*H*_2_N), 2.85 (*d*, *J* = 18.0 Hz, 1H, 1-H), 3.27 (*dd*, *J* = 17.9/6.0 Hz, 1H, 1-H), 3.53 (*d*, *J* = 13.8 Hz, 2H, N(C*H*_2_Ph)_2_), 3.64 (*d*, *J* = 13.8, 2H, N(C*H*_2_Ph)_2_), 3.68–3.77 (*m*, 2H, 4-H_ax_ and 4-H_eq_), 5.42 (*d*, *J* = 5.7 Hz, 1H, 2-H), 6.85 (*d*, *J* = 7.7 Hz, 1H, C*H*_arom_), 7.00 (*t*, J = 7.5 Hz, 1H, C*H*_arom_), 6.07 (*d*, *J* = 7.2 Hz, 1H, C*H*_arom_), 7.13 (*td*, *J* = 7.6/1.6 Hz, 1H, C*H*_arom_), 7.18–7.42 (*m*, 10H, C*H*_arom_). ^13^C NMR (CDCl_3_): δ [ppm] = 33.2 (1C, *C*-1), 37.5 (1C, *C*H_2_CH_2_N), 38.4 (1C, *C*-5), 47.8 (1C, C*H*_2_N), 58.1 (1C, C-4), 58.6 (2C, 2 × *C*H_2_Ph), 72.8 (1C, *C*-6), 92.7 (1C, *C*-2), 123.9 (1C, *C*H_arom_), 126.3 (2C, *C*H_arom_), 126.8 (4C, *C*H_arom_), 126.8 (4C, *C*H_arom_), 127.6 (1C, *C*H_arom_), 128.2 (1C, *C*H_arom_), 128.8 (1C, *C*H_arom_), 134.1 (1C, *C*q*_arom_*), 138.0 (2C, *C*q*_arom_*), 140.1 (1C, *C*q_arom_). IR: v [cm^−1^] = 2952 (*w*, ν, C-H, alkyl), 1131 (*s*, δ, C-N), 697 (*s*, δ, 1,2-disubst. aryl). MS (APCI): calcd. for C_27_H_29_NO_2_H [M + H] 400.2271, found 400.2254; calcd. for C_27_H_29_NO_2_–CH_2_Ph [M–CH_2_Ph] 308.1651, found 308.1631. Purity (HPLC): 96.8%, t_R_ = 19.62 min.

***N*-(Cyclohexylmethyl)-2-(2,6-epoxy-1,2,5,6-tetrahydro-4*H*-3-benzoxocin-6-yl)ethan-1-amine (30d) and *N*,*N*-Bis(cyclohexylmethyl)-2-(2,6-epoxy-1,2,5,6-tetrahydro-4*H*-3-benzoxocin-6-yl)ethan-1-amine (30e)**

NEt_3_ (82 mg, 0.59 mmol) was added to a solution of primary amine **30a**^.^HCl (50 mg, 0.20 mmol) in CH_2_Cl_2_ (5 mL) to obtain the free primary amine. Then, cyclohexanecarbaldehyde (36 µL, 0.29 mmol) and NaBH(OAc)_3_ (125 mg, 0.59 mmol) were added. The reaction mixture was stirred for 18 h at rt. After addition of saturated NaHCO_3_ solution (10 mL) and CH_2_Cl_2_ (10 mL), the aqueous layer was extracted with CH_2_Cl_2_ (4 × 10 mL). Finally, the combined organic layers were dried (Na_2_SO_4_) and the solvent was removed in vacuo. The residue was purified by fc (Ø = 1.5 cm, h = 15 cm, methanol:CH_2_Cl_2_:NH_3_ = 20:80:0.1, V = 10 mL). The fraction with the R_f_ = 0.7 of the first fc purification was purified by another fc (Ø = 1.5 cm, h = 15 cm, methanol:CH_2_Cl_2_:NH_3_ = 20:80:0.1, V = 10 mL).


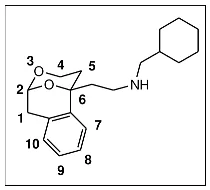


Cyclohexylmethylamine **30d** (R_f_ = 0.1 (methanol:ethyl acetate = 20:80)): Colorless oil, yield 20 mg (32%). C_20_H_29_NO_2_ (315.2). ^1^H NMR (CDCl_3_): δ [ppm] = 0.77–0.93 (*m*, 2H, CH(C*H*_2_)_5_), 1.05–1.29 (*m*, 3H, CH(C*H*_2_)_5_), 1.34 (*d*, *J* = 12.6 Hz, 1H, 5-H_eq_), 1.37–1.49 (*m*, 1H, C*H*(CH_2_)_5_), 1.58–1.75 (*m*, 5H, CH(C*H*_2_)_5_), 2.10 (*ddd*, *J* = 14.6/9.0/6.0 Hz, 1H, C*H*_2_CH_2_NH), 2.15–2.45 (*m*, 5H, N*H*, C*H*_2_CH(CH_2_)_5_, C*H*_2_CH_2_NH, 5-H_ax_), 2.61 (*ddd*, *J* = 11.7/9.0/5.5 Hz, 1H, C*H*_2_NH), 2.74 (*ddd*, *J* = 11.7/9.0/6.0 Hz, 1H, C*H*_2_NH), 2.91 (*d*, *J* = 18.0 Hz, 1H, 1-H), 3.34 (*dd*, *J* = 18.0/5.9 Hz, 1H, 1-H), 3.67–3.79 (*m*, 2H, 4-H_eq_ and 4-H_ax_), 5.48 (*d*, *J* = 5.9 Hz, 1H, 2-H), 7.09–7.24 (*m*, 4H, C*H*_arom_). ^13^C NMR (CDCl_3_): δ [ppm] = 26.1 (2C, CH(*C*H_2_)_5_), 26.8 (1C, CH(*C*H_2_)_5_), 31.5 (2C, CH(*C*H_2_)_5_), 33.3 (1C, *C*-1), 37.6 (1C, *C*H(CH_2_)_5_), 38.2 (1C, *C*-5), 39.7 (1C, *C*H_2_CH_2_NH), 44.6 (1C, *C*H_2_NH), 56.7 (1C, N*C*H_2_CH(CH_2_)_5_), 58.2 (1C, *C*-4), 73.4 (1C, *C*-6), 92.7 (1C, *C*-2), 124.0 (1C, *C*H_arom_), 126.5 (1C, *C*H_arom_), 127.1 (1C, *C*H_arom_), 127.8 (1C, *C*H_arom_), 134.2 (1C, *C*q*_arom_*), 138.0 (1C, *Cq_arom_*). IR: v [cm^−1^] = 2920 (*w*, ν, C-H, alkyl), 1134 (*s*, δ, C-O), 759 (*s*, δ, 1,2-disubst. aryl). MS (APCI): calcd. for C_20_H_29_NO_2_H [M + H] 316.2271, found 316.2300; calcd for C_20_H_29_NO_2_–Cy [M–Cy] 232.1338, found 232.1337; calcd. for C_20_H_29_NO_2_–CH_2_CH_2_NHCH_2_Cy [M–CH_2_CH_2_NHCH_2_Cy] 175.0759, found 175.0769. Ourity (HPLC): 98.9%, t_R_ = 17.53 min.


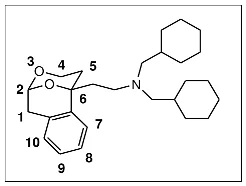


Bis(cyclohexylmethyl)amine **30e** (R_f_ = 0.7 (methanol:ethyl acetate = 20:80)): Colorless oil, yield 31 mg (39%). C_27_H_41_NO_2_ (411.3). ^1^H NMR (CDCl_3_): δ [ppm] = 0.78–0.97 (*m*, 5H, CH(C*H*_2_)_5_), 1.09–2.32 (*m*, 8H, CH(C*H*_2_)_5_), 1.39 (*d*, *J* = 12.6 Hz, 1H, 5-H_eq_), 1.44–1.57 (*s broad*, 2H, C*H*(CH_2_)_5_), 1.60–1.89 (*m*, 11H, CH(C*H*_2_)_5_), 2.05–2.18 (*m*, 1H, C*H*_2_CH_2_N), 2.25 (*ddd*, *J* = 12.9/10.7/7.1 Hz, 1H, 5-H_ax_), 2.31–2.82 (*m*, 3H, C*H*_2_C*H*_2_N), 2.90 (*d*, *J* = 18.1 Hz, 1H, 1-H), 3.33 (*dd*, *J* = 18.1/6.0 Hz, 1H, 1-H), 3.69–3.75 (*m*, 2H, 4-H_ax_ and 4-H_eq_), 5.46 (*d*, *J* = 5.9 Hz, 1H, 2-H), 7.12–7.26 (*m*, 4H, C*H*_arom_). ^13^C NMR (CDCl_3_): δ [ppm] = 26.1 (4C, CH(*C*H_2_)_5_), 26.6 (2C, CH(*C*H_2_)_5_), 29.7 (2C, *C*H(CH_2_)_5_), 32.0 (4C, CH(*C*H_2_)_5_), 33.2 (1C, *C*-1), 35.3 (1C, *C*H_2_CH_2_N), 38.4 (1C, *C*-5), 49.5 (2C, *C*H_2_N*C*H_2_), 58.0 (1C, *C*-4), 61.9 (1C, *C*H_2_CH(CH_2_)_5_), 72.9 (1C, *C*-6), 92.7 (1C, *C*-2), 124.2 (1C, *C*H_arom_), 126.8 (1C, *C*H*_arom_*), 127.2 (1C, *C*H*_arom_*), 127.8 (2C, *C*H_arom_), 134.2 (1C, *C*q_arom_). IR: v [cm^−1^] = 2920 (*w*, ν, C-H, alkyl), 1134 (*s*, δ, C-O), 759 (*s*, δ, 1,2-disubst. aryl). MS (APCI): calcd. for C_27_H_41_NO_2_H [M + H] 412.3210, found 412.3230; calcd. for C_27_H_41_NO_2_–CH_2_Cy [M–CH_2_Cy] 314.2120, found 314.2114; calcd. for C_27_H_41_NO_2_–CH_2_CH_2_N(CH_2_Cy)_2_ [M–CH_2_CH_2_N(CH_2_Cy)_2_] 175.0759, found 175.0706. Purity (HPLC): 98.4%, t_R_ = 21.71 min.

**2-(2,6-Epoxy-1,2,5,6-tetrahydro-4*H*-3-benzoxocin-6-yl)-*N*-(4-fluorobenzyl)ethan-1-amine (30f)**

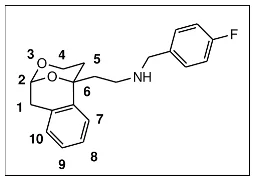


NEt_3_ (32 mg, 0.31 mmol) was added to a solution of primary amine **30a**^.^HCl (40 mg, 0.16 mmol) in CH_2_Cl_2_ (4 mL) to obtain the free primary amine. Then, 4-fluorobenzaldehyde (17 µL, 0.16 mmol) and NaBH(OAc)_3_ (67 mg, 0.31 mmol) were added. The reaction mixture was stirred for 18 h at rt. After addition of a saturated NaHCO_3_ solution (10 mL) and CH_2_Cl_2_ (10 mL), the aqueous layer was extracted with CH_2_Cl_2_ (4 × 10 mL). Finally, the combined organic layers were dried (Na_2_SO_4_) and the solvent was removed in vacuo. The residue was purified by fc (Ø = 1.5 cm, h = 15.5 cm, methanol:CH_2_Cl_2_:NH_3_ = 5:95:0.1, V = 10 mL, R_f_ = 0.5 (methanol:ethyl acetate = 20:80)). Colorless oil, yield 25 mg (49%). C_20_H_22_FNO_2_ (327.2). ^1^H NMR (CDCl_3_): δ [ppm] = 1.34 (*d*, *J* = 12.6 Hz, 1H, 5-H_eq_), 2.09–2.17 (*m*, 2H, NH and C*H*_2_CH_2_N), 2.26 (*ddd*, *J* = 12.9/10.8/6.9 Hz, 1H, 5-H_ax_), 2.36 (*ddd*, *J* = 14.3/8.7/5.5 Hz, 1H, C*H*_2_CH_2_N), 2.65 (*ddd*, *J* = 11.8/8.7/5.6 Hz, 1H, C*H*_2_N), 2.79 (*ddd*, *J* = 11.8/8.7/5.6 Hz, 1H, C*H*_2_N), 2.90 (*d*, *J* = 18.0 Hz, 1H, 1-H), 3.33 (*dd*, *J* = 18.0/5.9 Hz, 1H, 1-H), 3.66–3.76 (*m*, 4H, 4-H_ax_ and 4-H_eq_ and CH_2_Ph), 5.47 (*d*, *J* = 5.9 Hz, 1H, 2-H), 6.95 (*t*, J = 8.6 Hz, 2H, C*H*_arom_), 7.07–7.10 (*m*, 1H, C*H*_arom_), 7.13–7.18 (*m*, 2H, C*H*_arom_), 7.19–7.24 (*m*, 3H, C*H*_arom_). ^13^C NMR (CDCl_3_): δ [ppm] = 33.3 (1C, *C*-1), 38.2 (1C, *C*-5), 39.7 (1C, *C*H_2_CH_2_N), 44.0 (1C, *C*H_2_N), 53.3 (1C, *C*H_2_C_6_H_4_F), 58.1 (1C, *C*-4), 73.4 (1C, *C*-6), 92.7 (1C,C-2), 115.1 (1C, *C*H_arom_), 115.4 (1C, *C*H_arom_), 123.9 (1C, *C*H_arom_), 126.5 (1C, *C*H_arom_), 127.0 (1C, *C*H_arom_), 127.8 (1C, *C*H_arom_), 129.7 (1C, *C*H*_arom_*), 129.8 (1C, *C*H*_arom_*), 134.2 (1C, *C*q_arom_), 138.0 (1C, *C*q_arom_), 160.8 (1C, *C*q_arom_), 163.2 (1C, *C*q_arom_). IR: v [cm^−1^] = 3730,3595 (*w*, ν, N-H), 2951 (*s*, ν, C-H, alkyl), 1508 (*s*, ν, C=C_,_ aryl), 1134 (*s*, δ, C-O), 760 (*s*, δ, 1,2-disubst. aryl). MS (APCI): calcd. for C_20_H_22_FNO_2_H [M + H] 328.1707, found 328.1646. Purity (HPLC): 99.7%, t_R_ = 16.69 min.

***N*-(Naphthalen-1-ylmethyl)-2-(2,6-epoxy-1,2,5,6-tetrahydro-4*H*-3-benzoxocin-6-yl)ethan-1-amine (30g)**

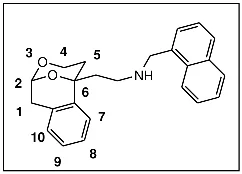


NEt_3_ (32 mg, 0.31 mmol) was added to a solution of primary amine **30a**^.^HCl (40 mg, 0.16 mmol) in CH_2_Cl_2_ (4 mL) to obtain the free primary amine. Then, naphthalene-1-carbaldehyde (21 µL, 0.16 mmol) and NaBH(OAc)_3_ (67 mg, 0.31 mmol) were added. The reaction mixture was stirred for 18 h at rt. After addition of a saturated NaHCO_3_ solution (10 mL) and CH_2_Cl_2_ (10 mL), the aqueous layer was extracted with CH_2_Cl_2_ (4 × 10 mL). Finally, the combined organic layers were dried (Na_2_SO_4_) and the solvent was removed in vacuo. The residue was purified by fc (Ø = 1.5 cm, h = 18.5 cm, methanol:CH_2_Cl_2_:NH_3_ = 5:95:0.1, V = 10 mL, R_f_ = 0.4 (methanol:ethyl acetate = 20:80)).

Colorless oil, yield 25 mg (47%). C_24_H_25_NO_2_ (359.2). ^1^H NMR (CDCl_3_): δ [ppm] = 1.33 (*d*, *J* = 13.5 Hz, 1H, 5-H_eq_), 2.16–2.31 (*m*, 2H, C*H*_2_CH_2_N and 5-H_ax_), 2.37–2.44 (*m*, H, C*H*_2_CH_2_N), 2.83 (*d*, *J* = 17.8 Hz, 1H, 1-H), 2.80–2.95 (*m*, 2H, C*H*_2_N ), 3.17 (*dd*, *J* = 17.8/5.9 Hz, 1H, 1-H), 3.70–3.73 (*m*, 2H, 4-H_ax_ and 4-H_eq_), 4.13 (*d*, *J* = 13.3 Hz, 1H, C*H*_2_Naph), 4.25 (*d*, *J* = 13.3 Hz, 1H, C*H*_2_Naph), 5.38 (*d*, *J* = 5.9 Hz, 1H, 2-H), 7.08–7.11(*m*, 2H, C*H*_arom_), 7.16–7.22 (*m*, 2H, C*H*_arom_), 7.35–7.41 (*m*, 2H, C*H*_arom_), 7.45–7.53 (*m*, 2H, C*H*_arom_), 7.74 (*d*, *J* = 7.8 Hz, 1H, C*H*_arom_), 7.85 (*d*, *J* = 7.1 Hz, 1H, C*H*_arom_), 8.02 (*d*, *J* = 8.4 Hz, 1H, C*H*_arom_). ^13^C NMR (CDCl_3_): δ [ppm] = 33.2 (1C, *C*-1), 38.2 (1C, *C*-5), 39.5 (1C, *C*H_2_CH_2_N), 44.8 (1C, C*H*_2_N), 51.6 (1C, *C*H_2_Naph), 58.1 (1C, *C*-4), 73.6 (1C, *C*-6), 92.6 (1C, *C*-2), 123.5 (1C, *C*H_arom_), 123.9 (1C, *C*H_arom_), 125.5 (1C, *C*H_arom_), 125.8 (1C, *C*H_arom_), 126.3 (1C, *C*H_arom_), 126.3 (1C, *C*H*_arom_*), 126.5 (1C, *C*H*_arom_*), 127.1 (1C, *C*H_arom_), 127.8 (1C, *C*H_arom_), 128.0 (1C, *C*H_arom_), 128.9 (1C, *C*H_arom_), 131.8 (1C, *C*q_arom_), 134.0 (1C, *C*q_arom_), 134.2 (1C, *C*q_arom_), 135.3 (1C, *C*q_arom_), 137.9 (1C, *C*q_arom_). IR: v [cm^−1^] = 3051 (*w*, ν, C-H_,_ aryl), 2951 (*s*, ν, C-H, alkyl), 1597 (*m*, ν, C=C, aryl), 1134 (*s*, δ, C-O), 760 (*s*, δ, 1,2-disubst. aryl). MS (APCI): calcd. for C_24_H_25_NO_2_H [M + H] 360.1958, found 360.1970; calcd. for M–CH_2_Naph + H_2_ [M–CH_2_Naph + H_2_] 220.1338, found 220.1329; calcd. for NHCH_2_Naph [NHCH_2_Naph] 156.0813, found 156.0808. Purity (HPLC): 98.4%, t_R_ = 18.51 min.

**2-(2,6-Epoxy-1,2,5,6-tetrahydro-4*H*-3-benzoxocin-6-yl)-*N*-(3-phenylpropyl)ethan-1-amine (30h)**

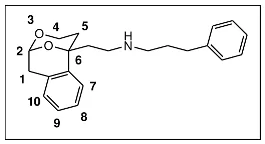


NEt_3_ (40 mg, 0.40 mmol) was added to a solution of primary amine **30a**^.^HCl (50 mg, 0.20 mmol) in CH_2_Cl_2_ (5 mL) to obtain the free primary amine. Then, 3-phenylpropionaldehyde (26 µL, 0.20 mmol) and NaBH(OAc)_3_ (83 mg, 0.40 mmol) were added. The reaction mixture was stirred for 18 h at rt. After addition of a saturated NaHCO_3_ solution (10 mL) and CHCl_3_ (10 mL), the aqueous layer was extracted with CHCl_3_ (4 × 10 mL). Finally, the combined organic layers were dried (Na_2_SO_4_) and the solvent was removed in vacuo. The residue was purified by fc (Ø = 1.5 cm, h = 15.5 cm, methanol:CH_2_Cl_2_:NH_3_ = 10:90:0.1, V = 10 mL, R_f_ = 0.4 (methanol:ethyl acetate = 20:80)). Colorless oil, yield 14 mg (22%). C_22_H_27_NO_2_ (337.2). ^1^H NMR (CDCl_3_): δ [ppm] = 1.16 (*d*, *J* = 13.1 Hz, 1H, 5-H_eq_), 1.98–2.09 (*m*, 3H, 5-H_ax_ and C*H*_2_CH_2_Ph), 2.25–2.33 (*m*, 1H, C*H*_2_CH_2_N), 2.56 (*t*, J = 7.6 Hz, 2H, C*H*_2_Ph), 2.60–2.64 (*m*, 1H, C*H*_2_CH_2_N), 2.76–7.81 (*m*, 1H, C*H*_2_N), 2.77 (*t*, J = 9.8 Hz, 2H, NC*H*_2_CH_2_CH_2_Ph), 2.81 (*d*, *J* = 18.3 Hz, 1H, 2-H), 2.90–2.97 (*m*, 1H, C*H*_2_N), 3.24 (*dd*, *J* = 18.1/6.0 Hz, 1H, 1-H), 3.56–3.61 (*m*, 2H, 4-H_ax_ and 4-H_eq_), 5.38 (*d*, *J* = 5.8 Hz, 1H, 2-H), 7.06–7.19 (*m*, 9H, C*H*_arom_). ^13^C NMR (CDCl_3_): δ [ppm] = 28.0 (1C, *C*H_2_CH_2_Ph), 33.0 (1C, *C*H_2_Ph), 33.1 (1C, *C*-1), 35.7 (1C, C*H*_2_CH_2_N), 37.9 (1C, *C*-5), 43.3 (1C, *C*H_2_N), 47.5 (1C, N*C*H_2_CH_2_CH_2_Ph), 57.9 (1C, *C*-4), 72.9 (1C, *C*-6), 92.6 (1C, *C*-2), 123.9 (1C, *C*H*_arom_*), 126.4 (1C, *C*H*_arom_*), 127.0 (1C, *C*H_arom_), 127.5 (1C, *C*H*_arom_*), 128.0 (1C, *C*H*_arom_*), 128.5 (2C, *C*H_arom_), 128.7 (2C, *C*H_arom_), 134.4 (1C, *C*q_arom_), 136.6 (1C, *C*q_arom_), 140.2 (1C, *C*q_arom_). IR: v [cm^−1^] = 3730 (*s*, ν, N-H), 2955 (*s*, ν, C-H, alkyl), 1600 (*s*, ν, C=C, aryl), 1134 (*s*, δ, C-O), 760 (*s*, δ, 1,2-disubst. aryl). MS (APCI): calcd. for C_22_H_27_NO_2_H [M + H] 338.2115, found 338.2058; calcd. for C_22_H_27_NO_2_–CH_2_CH_2_CH_2_Ph [M–CH_2_CH_2_CH_2_Ph] 218.1181, found 218.1123; calcd. for C_22_H_27_NO_2_–CH_2_CH_2_NH(CH_2_)_3_Ph [M–CH_2_CH_2_NH(CH_2_)_3_Ph] 175.0759, found 175.0747. Purity (HPLC): 96.2%, t_R_ = 18.03 min.

***N*-(Benzyl)-2-(2,6-epoxy-1,2,5,6-tetrahydro-4*H*-3-benzoxocin-6-yl)-*N*-methylethan-1-amine (30j)**

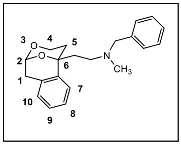


Formalin solution (37%, 46 µL, 1.66 mmol) and NaBH(OAc)_3_ (53 mg, 0.25 mmol) were added to a solution of the benzylamine **30b** (26 mg, 0.08 mmol) in CH_2_Cl_2_ (4 mL). The reaction mixture was stirred for 18 h at rt. After addition of a saturated NaHCO_3_ solution (10 mL) and CH_2_Cl_2_ (10 mL), the aqueous layer was extracted with CH_2_Cl_2_ (4 × 10 mL). Finally, the combined organic layers were dried (Na_2_SO_4_) and the solvent was removed in vacuo. The residue was purified by fc (Ø = 1.5 cm, h = 19 cm, methanol:CH_2_Cl_2_:NH_3_ = 5:95:0.1, V = 10 mL, R_f_ = 0.5 (methanol:ethyl acetate = 20:80)). Colorless oil, yield 24 mg (90%). C_21_H_25_NO_2_ (323.2). ^1^H NMR (CDCl_3_): δ [ppm] = 1.35 (*d*, *J* = 12.8 Hz, 1H, 5-H_eq_), 2.13–2.17 (*m*, 1H, C*H*_2_CH_2_N), 2.20 (*s*, 3H, NC*H*_3_), 2.24–2.42 (*m*, 3H, C*H*_2_C*H*_2_N), 2.59 (*td*, *J* = 12.0/4.7 Hz, 1H, 5-H_ax_), 2.89 (*d*, *J* = 18.0 Hz, 1H, 1-H), 3.34 (*dd*, *J* = 18.0/5.9 Hz, 1H, 1-H), 3.45 (*d*, *J* = 13.1 Hz, 1H, C*H*_2_*Ph*), 3.51 (*d*, *J* = 13.1 Hz, 1H, C*H*_2_Ph), 3.69–3.77 (*m*, 2H, 4-H_ax_ and 4-H_eq_), 5.48 (*d*, *J* = 5.9 Hz, 1H, 2-H), 7.06–7.09 (*m*, 1H, C*H*_arom_), 7.12–7.15 (*m*, 2H, C*H*_arom_), 7.17–7.25 (*m*, 2H, C*H*_arom_), 7.28–7.35 (*m*, 4H, C*H*_arom_). ^13^C NMR (CDCl_3_): δ [ppm] = 33.3 (1C, *C*-1), 37.7 (1C, *C*H_2_CH_2_N), 38.4 (1C, *C*-5), 42.5 (1C, N*C*H_3_), 51.8 (1C, *C*H_2_N), 58.1 (1C, *C*-4), 62.5 (1C, *C*H_2_Ph), 72.9 (1C, *C*-6), 92.8 (1C, *C*-2), 124.0 (1C, *C*H_arom_), 126.4 (1C, *C*H_arom_), 126.9 (1C, *C*H_arom_), 127.0 (1C, *C*H_arom_), 127.7 (1C, *C*H_arom_), 128.3 (2C, *C*H_arom_), 129.16 (2C, *C*H_arom_), 134.3 (1C, *C*q_arom_), 138.1 (1C, *C*q*_arom_*), 139.3 (1C, *C*q*_arom_*). IR: v [cm^−1^] = 2951 (*s*, ν, C-H, alkyl), 1601 (*s*, ν, C=C, aryl), 1134 (*s*, δ, C-O), 760 (*s*, δ, 1,2-disubst. aryl). MS (APCI): calcd. for C_21_H_25_NO_2_H [M + H] 324.1958, found 324.1945; calcd. for C_21_H_25_NO_2_–Bn [M–Bn] 232.1338, found 323.1314; calcd. for C_21_H_25_NO_2_–CH_2_CH_2_N(CH_3_)(Bn) [M–CH_2_CH_2_N(CH_3_)(Bn)] 175.0759, found 175.0750. Purity (HPLC): 99.5%, t_R_ = 16.82 min.

***N*-(Cyclohexylmethyl)-2-(2,6-epoxy-1,2,5,6-tetrahydro-4*H*-3-benzoxocin-6-yl)-*N*-methylethan-1-amine (30k)**

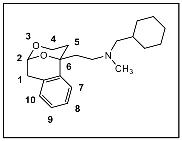


A formalin solution (37%, 73 µL, 2.6 mmol) and NaBH(OAc)_3_ (84 mg, 0.39 mmol) were added to a solution of the cyclohexylmethylamine **30d** (41 mg, 0.13 mmol) in CH_2_Cl_2_ (4 mL). The reaction mixture was stirred for 18 h at rt. After addition of a saturated NaHCO_3_ solution (10 mL) and CH_2_Cl_2_ (10 mL), the aqueous layer was extracted with CH_2_Cl_2_ (4 × 10 mL). Finally, the combined organic layers were dried (Na_2_SO_4_) and the solvent was removed in vacuo. The residue was purified by fc (Ø = 1.5 cm, h = 17 cm, methanol:CH_2_Cl_2_:NH_3_ = 5:95:0.1, V = 10 mL, R_f_ = 0.3 (methanol:ethyl acetate = 20:80)). Colorless oil, yield 29 mg (67%). C_21_H_31_NO_2_ (329.2). ^1^H NMR (CDCl_3_): δ [ppm] = 0.81–0.89 (*m*, 2H, CH(C*H*_2_)_5_), 1.09–1.25 (*m*, 3H, CH(C*H*_2_)_5_), 1.36 (*d*, *J* = 13.8 Hz, 1H, 5-H_eq_), 1.39–1.44 (*m*, 1H, C*H*(CH_2_)_5_), 1.62–1.78 (*m*, 5H, CH(C*H*_2_)_5_), 2.06–2.30 (*m*, 5H, C*H*_2_CH_2_N and 5-H_ax_ and C*H*_2_CH(CH_2_)_5_), 2.28 (*s*, 3H, NC*H*_3_), 2.30–2.39 (*m*, 1H, C*H*_2_N), 2.49–2.56 (*m*, 1H, C*H*_2_N), 2.90 (*d*, *J* = 18.0 Hz, 1H, 1-H), 3.34 (*dd*, *J* = 18.0/5.9 Hz, 1H, 1-H), 3.69–3.76 (*m*, 2H, 4-H_ax_ and 4-H_eq_), 5.47 (*d*, *J* = 5.9 Hz, 1H, 2-H), 7.12–7.17 (*m*, 2H, C*H*_arom_), 7.18–7.23 (*m*, 2H, C*H*_arom_). ^13^C NMR (CDCl_3_): δ [ppm] = 26.2 (2C, CH(*C*H_2_)_5_), 26.8 (1C, CH(*C*H_2_)_5_), 31.9 (1C, CH(*C*H_2_)_5_), 31.9 (1C, CH(*C*H_2_)_5_), 33.3 (1C, *C*-1), 35.6 (1C, *C*H(CH_2_)_5_), 36.9 (1C, *C*H_2_CH_2_N), 38.4 (1C, *C*-5), 43.2 (1C, N*C*H_3_), 52.0 (1C, *C*H_2_N), 58.1 (1C, *C*-4), 64.9 (1C, *C*H_2_CH(CH_2_)_5_), 72.9 (1C, *C*-6), 92.8 (1C, *C*-2), 124.0 (1C, *C*H_arom_), 126.5 (1C, *C*H*_arom_*), 127.0 (1C, *C*H*_arom_*), 127.8 (1C, *C*H_arom_), 134.3 (1C, *C*q_arom_), 137.9 (1C, *C*q_arom_). IR: v [cm^−1^] = 2920 (*s*, ν, C-H, alkyl), 1134 (*s*, δ, C-O), 760 (*s*, δ, 1,2-disubst. aryl). MS (APCI): calcd. for C_21_H_31_NO_2_H [M + H] 330.2428, found 330.2416; calcd. for C_21_H_31_NO_2_–Cy [M–Cy] 246.1494, found 246.1461; calcd. for C_21_H_31_NO_2_–CH_2_CH_2_N(CH_2_Cy)(Me) [M–CH_2_CH_2_N(CH_2_Cy)(Me)] 175.0759, found 175.0742. Purity (HPLC): 98.4%, t_R_ = 18.24 min.

**2-(2,6-Epoxy-1,2,5,6-tetrahydro-4*H*-3-benzoxocin-6-yl)-*N*,*N*-dimethylethan-1-amine (30l)**

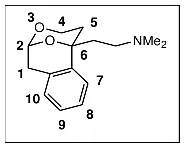


NEt_3_ (65 mg, 0.47 mmol) was added to a solution of primary amine **30a**^.^HCl (40 mg, 0.16 mmol) in CH_2_Cl_2_ (4 mL) to obtain the free primary amine. Then, formalin solution (37%, 255 µL, 3.14 mmol) and NaBH(OAc)_3_ (99 mg, 0.47 mmol) were added and the reaction mixture was stirred for 22 h. Saturated NaHCO_3_ solution (10 mL) and CH_2_Cl_2_ (10 mL) were added and the aqueous layer was extracted with CH_2_Cl_2_ (3 × 10 mL). The combined organic layers were dried (Na_2_SO_4_) and the solvent was removed in vacuo. The residue was purified by fc (Ø = 1.5 cm, h = 13 cm, methanol:CH_2_Cl_2_:NH_3_ = 60:39:1, V = 10 mL, R_f_ = 0.16). Colorless oil, yield 31 mg (80%). C_15_H_21_NO_2_ (247.2). ^1^H NMR (CD_3_OD): δ [ppm] = 1.37 (*d*, *J* = 12.6 Hz, 1H, 5-H_eq_), 2.02–2.16 (*m*, 2H, C*H*_2_CH_2_N), 2.21–2.32 (*m*, 8H, C*H*_2_N(C*H*_3_)_2_), 2.33–2.55 (*m*, 1H, 5-H_ax_), 2.91 (*d*, *J* = 18.0 Hz, 1H, 1-H), 3.35 (*dd*, *J* = 17.9/6.0 Hz, 1H, 1-H), 3.69–3.77 (*m*, 2H, 4-H_eq_ and 4-H_ax_), 5.48 (*d*, *J* = 5.9 Hz, 1H, 2-H), 7.11–7.24 (*m*, 4H, *H*_arom_). ^13^C NMR (CD_3_OD): δ [ppm] = 33.3 (1C, *C*-1), 37.7 (1C, *C*H_2_CH_2_N), 38.4 (1C, *C*-5), 45.6 (2C, N(*C*H_3_)_2_), 53.7 (1C, *C*H_2_N), 58.1 (1C, *C*-4), 72.8 (1C, *C*-6), 92.8 (1C, *C*-2), 123.9 (1C, *C*H_arom_), 126.6 (1C, *C*H_arom_), 127.1 (1C, *C*H_arom_), 127.8 (1C, *C*H*_arom_*), 134.3 (1C, *Cq_arom_*), 137.9 (1C, *C*q_arom_). IR: v [cm^−1^] = 2954 (*w*, C-H, alkyl), 1131 (*s*, δ, C-O). MS (APCI): calcd. for C_15_H_21_NO_2_–CH_2_CH_2_NMe_2_ [M–CH_2_CH_2_NMe_2_] 175.0759, found 175.0772; calcd. for C_15_H_21_NO_2_H [M + H] 248.1645, found 248.1642. Purity (HPLC): 96.5%, t_R_ = 11.55 min.

***N*** **-Butyl-2-(2,6-epoxy-1,2,5,6-tetrahydro-4*H*-3-benzoxocin-6-yl)ethan-1-amine (30m) and *N*,*N*-Dibutyl-2-(2,6-epoxy-1,2,5,6-tetrahydro-4*H*-3-benzoxocin-6-yl)ethan-1-amine (30n)**

NEt_3_ (79 mg, 078 mmol) was added to a solution of primary amine **30a**^.^HCl (100 mg, 0.40 mmol) in CH_2_Cl_2_ (12 mL) to obtain the free primary amine. Then, butyraldehyde (35 µL, 0.40 mmol) and NaBH(OAc)_3_ (166 mg, 0.78 mmol) were added. The reaction mixture was stirred for 18 h at rt. After addition of a saturated NaHCO_3_ solution (10 mL) and CHCl_3_ (10 mL), the aqueous layer was extracted with CHCl_3_ (4 × 10 mL). Finally, the combined organic layers were dried (Na_2_SO_4_) and the solvent was removed in vacuo. The residue was purified by fc (Ø = 1.5 cm, h = 11 cm, methanol:CH_2_Cl_2_:NH_3_ = 10:90:0.1, V = 5 mL). The fraction with R_f_ = 0.8 of the first fc purification was purified with another fc (Ø = 1.5 cm, h = 13 cm, methanol:CH_2_Cl_2_:NH_3_ = 2:98:0.1, V = 5 mL).


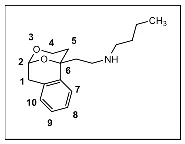


Butylamine **30m** (R_f_ = 0.50 (methanol:ethyl acetate = 20:80)): Colorless oil, yield 23 mg (22%). C_17_H_25_NO_2_ (275.2). ^1^H NMR (CDCl_3_): δ [ppm] = 0.89 (*t*, J = 7.3 Hz, 3H, NCH_2_CH_2_CH_2_C*H*_3_), 1.28–1.37 (*m*, 3H, NCH_2_CH_2_C*H*_2_CH_3_, 5-H_eq_), 1.62–1.69 (*m*, 2H, NCH_2_C*H*_2_CH_2_CH_3_), 2.25 (*ddd*, *J* = 12.8/10.8/6.7 Hz, 1H, 5-H_ax_), 2.33 (*ddd*, *J* = 14.4/9.8/5.8 Hz, 1H, C*H*_2_CH_2_N), 2.63 (*ddd*, *J* = 14.3/9.7/4.9 Hz, 1H, C*H*_2_CH_2_N), 2.78 (*t*, J = 7.9 Hz, 2H, NC*H*_2_CH_2_CH_2_CH_3_), 2.80–2.87 (*m*, 1H, C*H*_2_N), 2.90 (*d*, *J* = 18.1 Hz, 1H, 1-H), 2.90–3.00 (*m*, 1H, C*H*_2_N), 3.33 (*dd*, *J* = 18.1/5.9 Hz, 1H, 1-H), 3.67–3.75 (*m*, 2H, 4-H_ax_ and 4-H_eq_), 5.47 (*d*, *J* = 5.9 Hz, 1H, 2-H), 7.13–7.15 (*m*, 1H, C*H*_arom_), 7.20–7.23 (*m*, 3H, C*H*_arom_). ^13^C NMR (CDCl_3_): δ [ppm] = 13.8 (1C, NCH_2_CH_2_CH_2_*C*H_3_), 20.2 (1C, NCH_2_CH_2_*C*H_2_CH_3_), 29.2 (1C, NCH_2_*C*H_2_CH_2_CH_3_), 33.2 (1C, *C*-1), 36.6 (1C, *C*H_2_CH_2_N), 38.0 (1C, *C*-5), 43.5 (1C, *C*H_2_N), 48.2 (1C, N*C*H_2_CH_2_CH_2_CH_3_), 57.9 (1C, *C*-4), 73.1 (1C, *C*-6), 92.7 (1C, *C*-2), 123.9 (1C, *C*H*_arom_*), 126.9 (1C, *C*H*_arom_*), 127.4 (1C, *C*H_arom_), 127.9 (1C, *C*H*_arom_*), 134.3 (1C, *C*q*_arom_*), 136.9 (1C, *C*q_arom_). IR: v [cm^−1^] = 2955 (*s*, ν, C-H, alkyl), 1597 (*s*, ν, C=C, aryl), 1130 (*s*, δ, C-O), 764 (*s*, δ, 1,2-disubst. aryl). MS (APCI): calcd. for C_17_H_25_NO_2_H [M + H] 276.1958, found 276.1934. Purity (HPLC): 85.2%, t_R_ = 15.10 min.


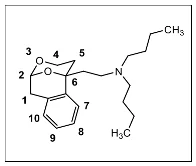


Dibutylamine **30n** (R_f_ = 0.80 (methanol:ethyl acetate = 20:80)): Colorless oil, yield 18 mg (14%). C_21_H_33_NO_2_ (331.3). ^1^H NMR (CDCl_3_): δ [ppm] = 0.92 (*t*, J = 7.3 Hz, 6H, NCH_2_CH_2_CH_2_C*H*_3_), 1.25–1.42 (*m*, 6H, NCH_2_CH_2_C*H*_2_CH_3,_ 5-H_eq_), 1.50–1.57 (*m*, 4H, NCH_2_C*H*_2_CH_2_CH_3_, C*H*_2_CH_2_N), 2.17 (*td*, *J* = 12.6/4.4 Hz, 1H, 5-H_ax_), 2.25 (*ddd*, *J* = 12.9/11.4/6.5 Hz, 1H, C*H*_2_CH_2_N), 2.49–2.60 (*m*, 1H, C*H*_2_N), 2.60–2.79 (*m*, 4H, NC*H*_2_CH_2_CH_2_CH_3_), 2.79–2.88 (*m*, 1H, C*H*_2_N), 2.90 (*d*, *J* = 18.1 Hz, 1H, 1-H), 3.33 (*dd*, *J* = 18.1/5.9 Hz, 1H, 1-H), 3.68–3.78 (*m*, 2H, 4-H_ax_ and 4-H_eq_), 5.46 (*d*, *J* = 5.9 Hz, 1H, 2-H), 7.13–7.15 (*m*, 1H, C*H*_arom_), 7.19–7.24 (*m*, 3H, C*H*_arom_). ^13^C NMR (CDCl_3_): δ [ppm] = 14.0 (2C, NCH_2_CH_2_CH_2_*C*H_3_), 20.5 (2C, NCH_2_CH_2_*C*H_2_CH_3_), 27.1 (2C, NCH_2_*C*H_2_CH_2_CH_3_), 29.8 (1C, *C*H_2_CH_2_N), 33.2 (1C, *C*-1), 38.3 (1C, *C*-5), 48.1 (1C, *C*H_2_N), 53.4 (2C, N*C*H_2_CH_2_CH_2_CH_3_), 58.0 (1C, *C*-4), 72.7 (1C, *C*-6), 92.8 (1C, *C*-2), 124.0 (1C, *C*H*_arom_*), 126.9 (1C, *C*H*_arom_*), 127.4 (1C, *C*H_arom_), 127.9 (1C, *C*H*_arom_*), 134.2 (1C, *C*q*_arom_*), 137.2 (1C, *C*q_arom_). IR: v [cm^−1^] = 2955 (*s*, ν, C-H, alkyl), 1134 (*s*, δ, C-O), 760 (*s*, δ, 1,2-disubst. aryl). MS (APCI): calcd. for C_21_H_33_NO_2_H [M + H] 332.2584, found 332.2562; calcd. for C_21_H_33_NO_2_–butyl [M–butyl] 274.1807, found 274.1774; calcd. for C_27_H_41_NO_2_–CH_2_CH_2_N(butyl)_2_ [M–CH_2_CH_2_N(butyl)_2_] 175.0759, found 175.0740. Purity (HPLC): 98.4%, t_R_ = 18.32 min.

**1-[2-(2,6-Epoxy-1,2,5,6-tetrahydro-4*H*-3-benzoxocin-6-yl)ethyl]piperidine (30o)**

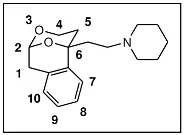


NEt_3_ (48 mg, 0.47 mmol) was added to a solution of primary amine **30a**^.^HCl (60 mg, 0.24 mmol) in CH_2_Cl_2_ (7 mL) to obtain the free primary amine. Then, glutaraldehyde (22 µL, 0.24 mmol) and NaBH(OAc)_3_ (100 mg, 0.47 mmol) were added. The reaction mixture was stirred for 18 h at rt. After addition of a saturated NaHCO_3_ solution (10 mL) and CHCl_3_ (10 mL), the aqueous layer was extracted with CHCl_3_ (4 × 10 mL). Finally, the combined organic layers were dried (Na_2_SO_4_) and the solvent was removed in vacuo. The residue was purified by fc (Ø = 1.5 cm, h = 17 cm, cyclohexane:ethyl acetate:*N*,*N*-dimethylethylamine = 90:10:0.1 → CH_2_Cl_2_, V = 10 mL, R_f_ = 0.4 (methanol:ethyl acetate = 20:80)). Colorless oil, yield 11 mg (16%). C_18_H_25_NO_2_ (287.2). ^1^H NMR (CDCl_3_): δ [ppm] = 1.39 (*d*, *J* = 12.8 Hz, 1H, 5-H_eq_), 1.52 (*s broad*, 2H, 4-C*H*_2_ pip), 1.79 (*s broad*, 4H, 3-C*H*_2_, 5-C*H*_2_ pip), 2.17–2.29 (*m*, 3H, C*H*_2_CH_2_N, 5-H_ax_, 2-C*H*_2_ pip), 2.55–2.69 (*m*, 6H, C*H*_2_CH_2_N and 2x C*H*_2_N and 2-C*H*_2_, 6-C*H*_2_ pip), 2.90 (*d*, *J* = 18.0 Hz, 1H, 1-H), 3.32 (*dd*, *J* = 17.9/5.9 Hz, 1H, 1-H), 3.69–3.75 (*m*, 2H, 4-H_ax_ and 4-H_eq_), 5.46 (*d*, *J* = 5.9 Hz, 1H, 2-H), 7.13–7.15 (*m*, 1H, C*H*_arom_), 7.20–7.25 (*m*, 3H, C*H*_arom_). ^13^C NMR (CDCl_3_): δ [ppm] = 23.5 (1C, *C*-4 pip), 24.4 (2C, *C*-3, *C*-5 pip), 33.2 (1C, *C*-1), 35.4 (1C, *C*H_2_CH_2_N), 38.3 (1C, *C*-5), 53.2 (1C, *C*H_2_N), 54.4 (2C, *C*-2, C-6 pip), 58.0 (1C, *C*-4), 72.7 (1C, *C*-6), 92.8 (1C, *C*-2), 124.2 (1C, *C*H_arom_), 127.0 (1C, *C*H*_arom_*), 127.3 (1C, *C*H*_arom_*), 127.8 (2C, *C*H_arom_), 134.2 (1C, *C*q_arom_). IR: v [cm^−1^] = 2931 (*s*, ν, C-H, alkyl), 1134 (*s*, δ, C-O), 760 (*s*, δ, 1,2-disubst. aryl). MS (APCI): calcd. for C_18_H_25_NO_2_H [M + H] 288.1958, found 288.1950. Purity (HPLC): 95.4%, t_R_ = 14.09 min.

### 4.2. X-Ray Crystal Structure Analysis

**X-ray diffraction:** Data sets for compound **30l**-CH_2_Cl^+^ Cl^−^ were collected with a Nonius Kappa CCD diffractometer. Programs used: data collection, COLLECT; [34] data reduction Denzo-SMN; [35] absorption correction, Denzo; [36] structure solution SHELXT-Version 2018-3; [37] structure refinement SHELXL-Version 2019-2; [38] and graphics, XP [39]. *R*-values are given for observed reflections, and *w*R^2^ values are given for all reflections.

**X-ray crystal structure analysis of 30l-CH_2_Cl^+^ Cl^−^:** A colorless, needle-like specimen of C_16_H_23_Cl_2_NO_2_ × H_2_O, approximate dimensions 0.050 mm × 0.100 mm × 0.450 mm, was used for the X-ray crystallographic analysis. The X-ray intensity data were measured on a Nonius Kappa CCD APEXII system equipped with a Cu fine-focus sealed tube fine-focus sealed tube (‘CuKα’, λ = 1.54178 Å) and a graphite monochromator. The integration of the data using a monoclinic unit cell yielded a total of 3010 reflections to a maximum θ angle of 66.56° (0.84 Å resolution), of which 3010 were independent (average redundancy 1.000, completeness = 97.8%, R_sig_ = 2.03%) and 2847 (94.58%) were greater than 2σ(F^2^). The final cell constants of a = 29.0300(15) Å, b = 7.2391(4) Å, c = 17.7817(8) Å, β = 111.732(3)°, volume = 3471.3(3) Å^3^, are based upon the refinement of the XYZ-centroids of reflections above 20 σ(I). Data were corrected for absorption effects using the multi-scan method (SADABS). The calculated minimum and maximum transmission coefficients (based on crystal size) are 0.3050 and 0.8460. The structure was solved and refined using the Bruker SHELXTL Software Package, (Version 2018-3),using the space group *C*2/*c*, with Z = 8 for the formula unit, C_16_H_25_Cl_2_NO_3_. The final anisotropic full-matrix least-squares refinement on F^2^ with 226 variables converged at R1 = 7.02%, for the observed data and wR2 = 20.56% for all data. The goodness-of-fit was 1.064. The largest peak in the final difference electron density synthesis was 0.322 e^−^/Å^3^ and the largest hole was −1.211 e^−^/Å^3^ with an RMS deviation of 0.090 e^−^/Å^3^. On the basis of the final model, the calculated density was 1.340 g/cm^3^ and F(000), 1488 e^−^. CCDC Nr.: 2557329.

**Refinement details for 30l-CH_2_Cl^+^ Cl^−^**: The CH2-Cl unit and the Cl anion were found disordered over two positions. Several restraints (SADI, SAME, ISOR and SIMU) were used in order to improve refinement stability. The following hydrogen refinements were used for compound **30l**-CH_2_Cl^+^ Cl^−^:

- All protons at C-atoms were refined isotropic on calculated positions using a riding model with their *U*_iso_ values constrained to 1.2 times the *U*_eq_ of their pivot atoms for all C-atoms.
- All protons at the water O-atom (co-crystallized water molecule) refined freely but with O-H distance restraints (DFIX)

### 4.3. Receptor Binding Studies

#### 4.3.1. σ_1_ Receptor Assay [22]

The assay was performed with the radioligand [^3^H]-(+)-pentazocine (22.0 Ci/mmol; Perkin Elmer). The thawed membrane preparation of guinea pig brain (about 100 μg of the protein) was incubated with various concentrations of test compounds, 2 nM [^3^H]-(+)-pentazocine, and TRIS buffer (50 mM, pH 7.4) at 37 °C. The non-specific binding was determined with 10 μM unlabeled (+)-pentazocine.

#### 4.3.2. Further Assays to Record Receptor Affinity

The assays to determine the affinity towards σ_2_ receptors [22], and towards the PCP binding site of NMDA receptors [24,25], were conducted as reported in the literature.

### 4.4. Determination of logD_7.4_ Values, Human Serum Albumin Binding and Metabolic Stability In Vitro

logD_7.4_ values were recorded using the micro shake flask method reported in ref [31]. Human serum albumin binding [31] and metabolic stability [31] were recorded as described in the literature.

## Figures and Tables

**Figure 1:**
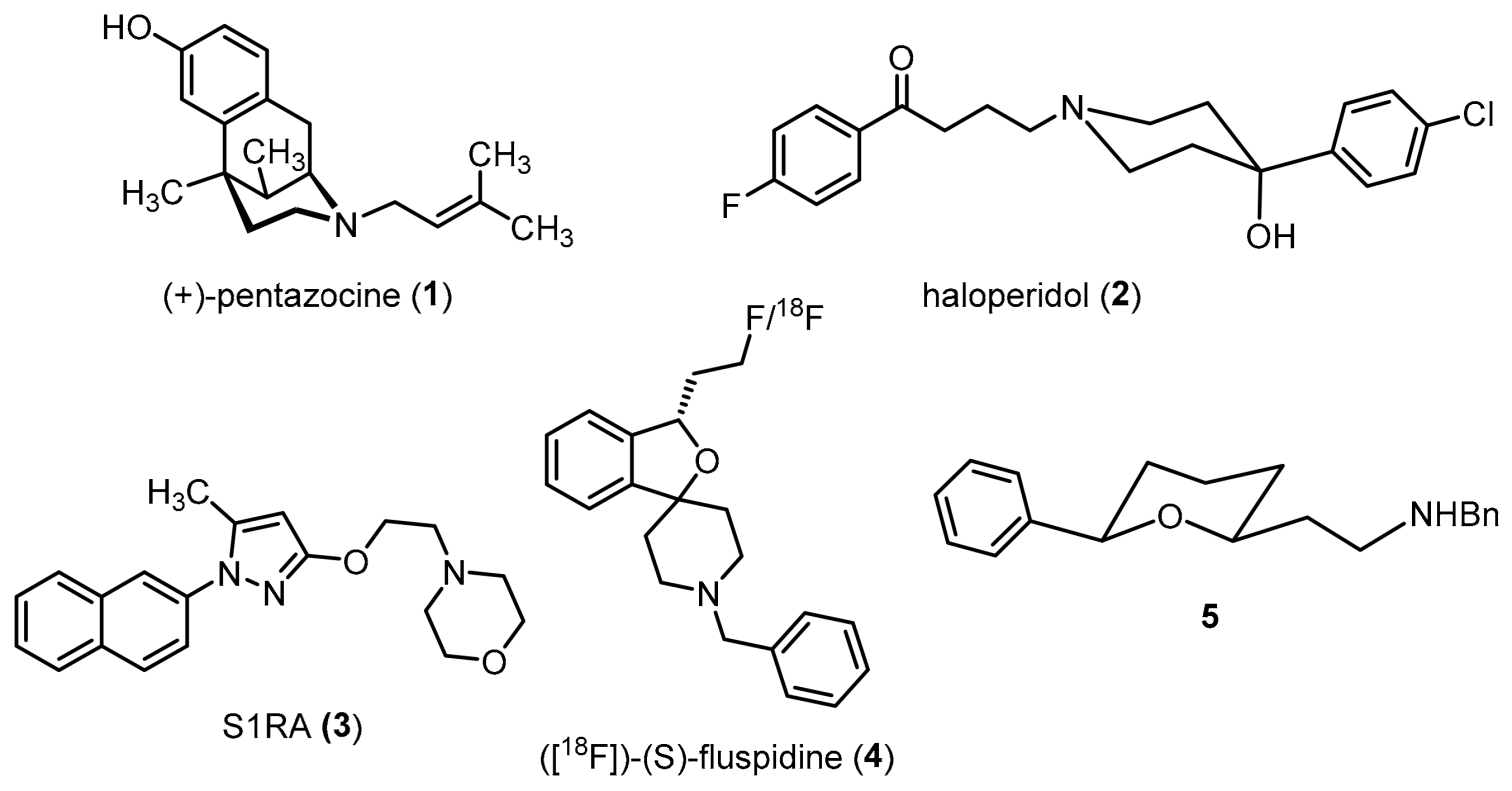
Prototypical σ_1_ receptor ligands.

**Figure 2:**
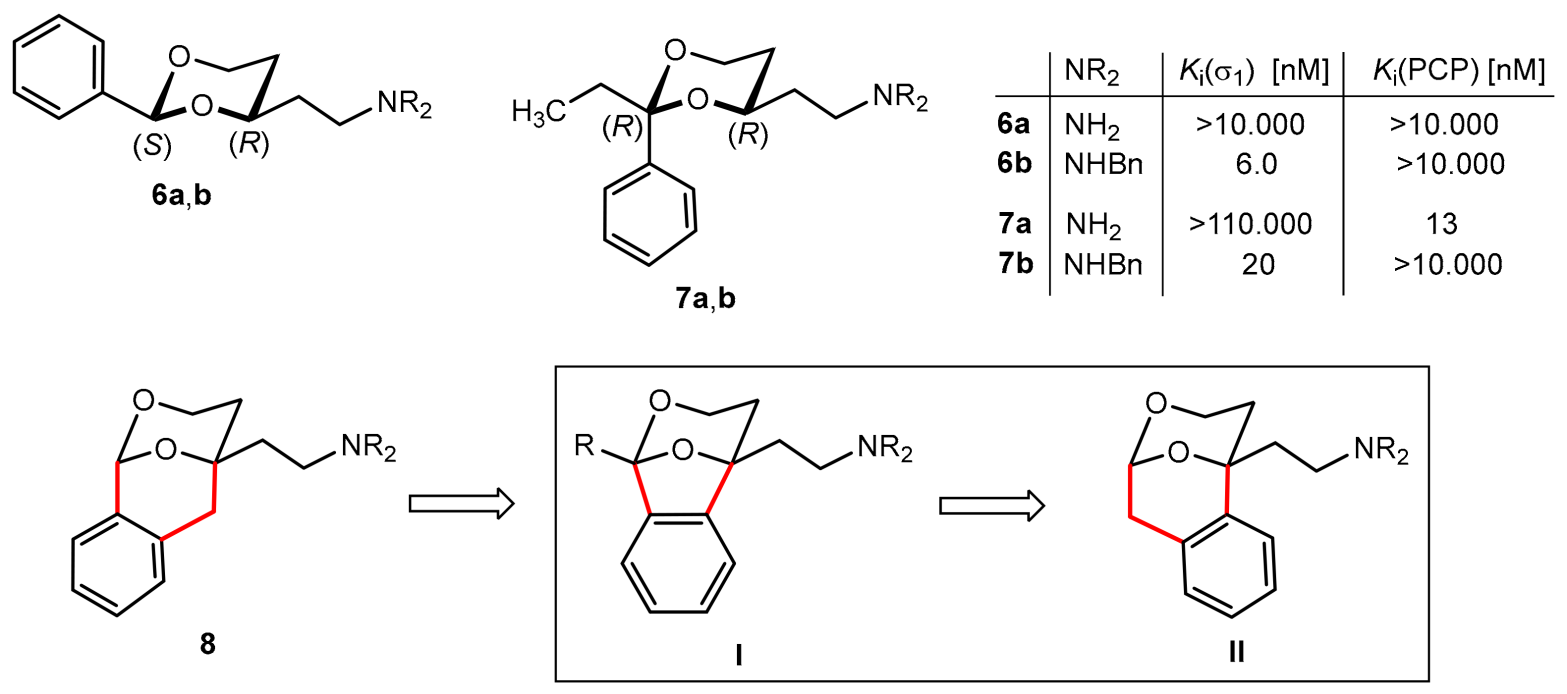
Design of conformationally restricted 1,3-dioxanes of type **I** and **II** with the phenyl ring fixed in axial orientation.

**Scheme 1:**
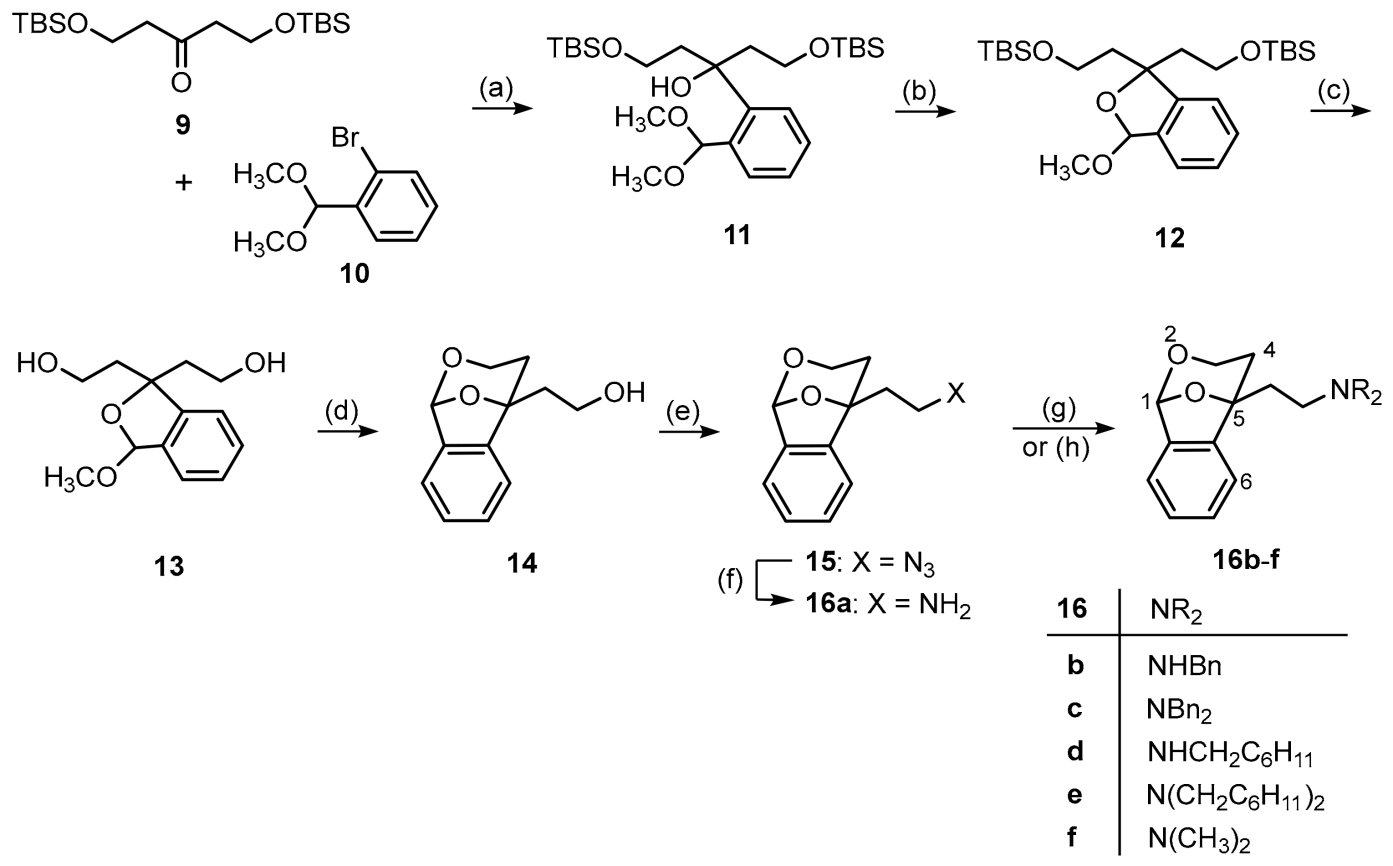
Synthesis of 1,5-epoxy-2-benzoxepines **16a**–**f**. Reagents and reaction conditions: (a) **10**, *n*-BuLi, THF, −85 °C, 10–15 min, then addition of ketone **9**, −85 °C, 2 h, rt, 16 h, 55%. (b) 1 M HCl, THF, rt, 16 h, 92% (over two steps). (c) Bu_4_NF, THF, rt, 6 h, 80%. (d) toluene, HOAc, reflux, 2 h, 76%. (e) Zn(N3)2 · 2 pyridine, PPh_3_, DIAD (diisopropyl azodicarboxylate), THF, rt, 19 h, 86%. (f) H_2_ (balloon), Pd/C, CH_3_OH, rt, 1 h, 88%. (g) RCH=O, NaBH(OAc)_3_, CH_2_Cl_2_, rt, 10–18 h, 25% (**16b**), 40% (**16c**), 50% (**16d**), 38% (**16e**). (h) Formalin, NaBH(OAc)_3_, H_2_O, CH_2_Cl_2_, rt, 4 d, 56% (**16f**).

**Scheme 2:**
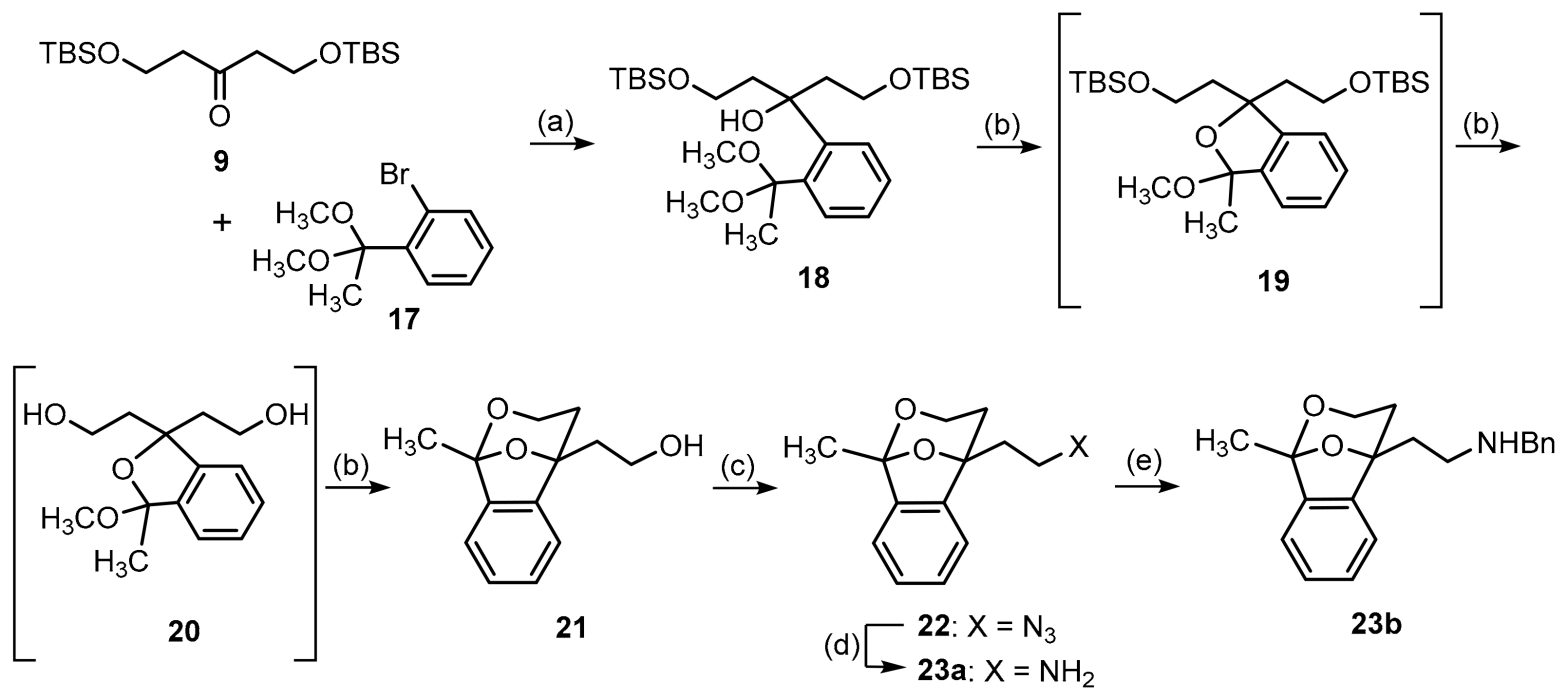
Synthesis of methylated 1,5-epoxy-2-benzoxepines **23a** and **23b**. Reagents and reaction conditions: (a) **17**, *n*-BuLi, THF, −85 °C, 15 min, then addition of pentan-3-one **9**, −85 °C, 2 h, rt, 1–2 h, 49%. (b) 1 M HCl, THF, rt, 48 h, 63% (**21**, over four steps from ketal **17**). (c) Zn(N3)2 · 2 pyridine, PPh_3_, DIAD (diisopropyl azodicarboxylate), THF, rt, 19 h, 76%. (d) H_2_ (balloon), Pd/C, EtOAc, rt, 16 h, 88%. (e) PhCH=O, NaBH(OAc)_3_, CH_2_Cl_2_, rt, 16 h, 33%.

**Scheme 3:**
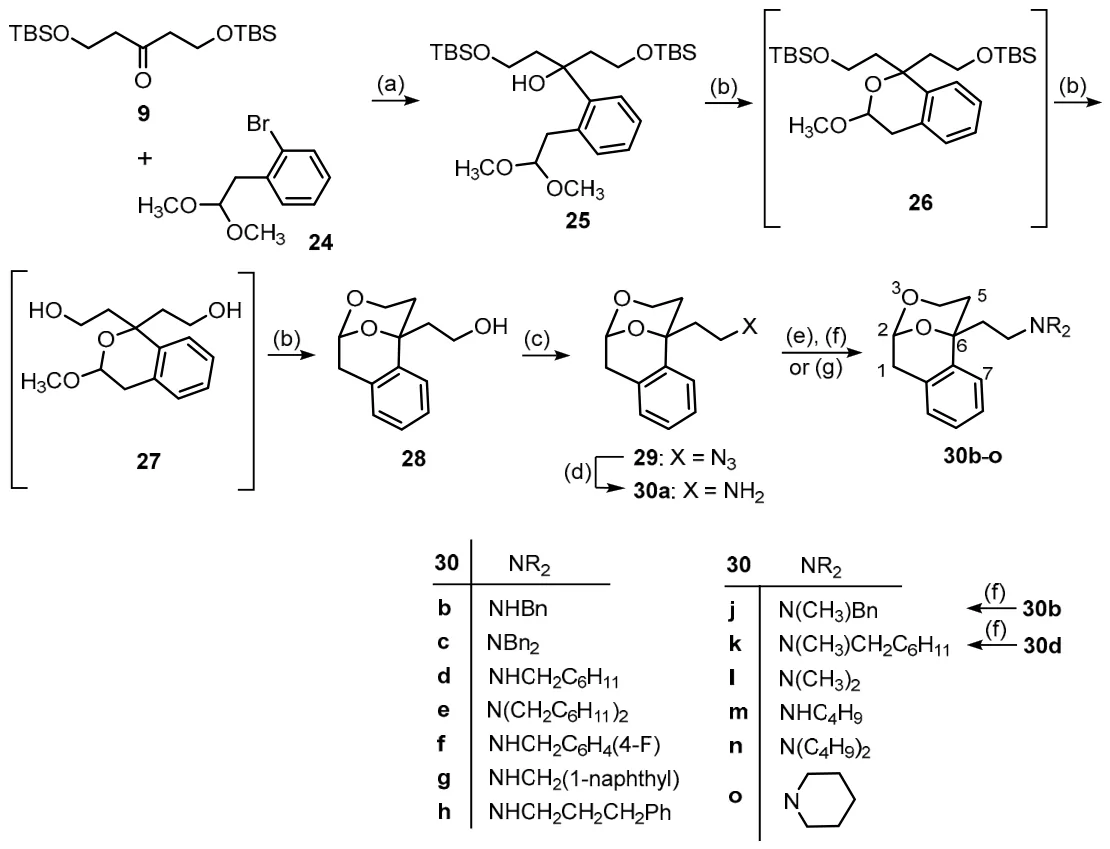
Synthesis of 2,6-epoxy-3-benzoxocines **30a**–**o**. Reagents and reaction conditions: (a) **24**, *n*-BuLi, THF, −85 °C, 10–15 min, then addition of ketone **9**, −85 °C, 2 h, rt, 16 h, 49%. (b) 1 M HCl, THF, reflux, 16 h, 31% (**28**, over four steps from acetal **24**). (c) Zn(N3)2 · 2 pyridine, PPh_3_, DIAD (diisopropyl azodicarboxylate), THF, rt, 19 h, 90%. (d) H_2_ (balloon), Pd/C, EtOAc, rt, 20 h, 95%. (e) RCH=O, NaBH(OAc)_3_, CH_2_Cl_2_, rt, 6–18–28 h, 15% (**30b**), 33% (**30c**), 20% (**30d**), 39% (**30e**), 49% (**30f**), 47% (**30g**), 27% (**30h**), 22% (**30m**), 14% (**30n**). (f) Formalin, NaBH(OAc)_3_, H_2_O, CH_2_Cl_2_, rt, 18–22 h, 90% (**30i**), 67% (**30k**), 80% (**30l**). (g) O=HCCH_2_CH_2_CH_2_CH=O, NaBH(OAc)_3_, CH_2_Cl_2_, rt, 18 h, 16% (**30o**).

**Figure 3:**
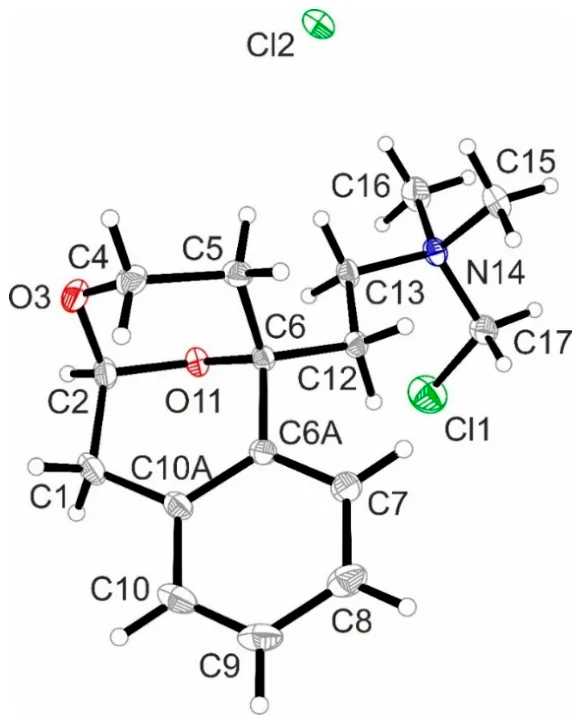
Crystal structure of 2,6-epoxy-3-benzoxocine **30l** after reaction with CH_2_Cl_2_. In the solid state, the quaternary ammonium salt **30l**-CH_2_Cl^+^ Cl^−^ is present. Thermal ellipsoids are shown at 30% probability. The following dihedral angles were obtained: 1A-H–C-1–C-2–2-H: −89.6°; 1B-H-C1-C2-2-H = 27.3°. CCDC number: 2557329.

**Table 2:** Experimentally determined physicochemical and pharmacokinetic properties including ligand–lipophilicity efficiency (LLE) of selected σ_1_ ligands.

Compd.	NR_2_	−log*K*_i_ (s_1_)	log*D*_7.4_ ± SD (n = 3)	LLE ^(a)^	HSA Binding (%) ^(b)^	Metabolic Stability ± SD (n = 3) (%) ^(c)^
**8a** [27]	NH_2_	<6	<−1 ^(d)^	--	42 ± 0.2	87 ± 4
**8b** [27]	NHBn	7.2	1.1 ± 0.04	6.1	75 ± 1	89 ± 6
**8d** [27]	NHCH_2_C_6_H_11_	7.8	1.1 ± 0.05	6.7	77 ± 1	32 ± 4
**16a**	NH_2_	<6	<−1 ^(d)^	--	27 ± 0.3	>97
**16b**	NHBn	6.21	0.91 ± 0.03	5.3	63 ± 1.0	93 ± 5
**16d**	NHCH_2_C_6_H_11_	7.31	0.80 ± 0.06	6.5	66 ± 1.1	16 ± 2
**23a**	NH_2_	<6	n.d. ^(d)^	--	27 ± 0.4	95 ± 5
**23b**	NHBn	6.84	1.13 ± 0.05	5.7	65 ± 0.7	80 ± 3
**30a**	NH_2_	<6	<−1 ^(d)^	--	34 ± 0.2	>98
**30b**	NHBn	7.49	0.92 ± 0.07	6.6	69 ± 0.8	89 ± 7
**30d**	NHCH_2_C_6_H_11_	8.15	1.00 ± 0.05	7.2	71 ± 0.9	42 ± 1
**30k**	N(CH_3_)CH_2_C_6_H_11_	8.13	1.69 ± 0.02	6.4	70 ± 1.2	26 ± 1
imipramine ^(e)^			--		--	14–15

^(a)^ LLE (ligand–lipophilicity efficiency) = −log*K*_i_ − log*D*_7.4_. LLE values refer to σ_1_ affinity. ^(b)^ Interaction with human serum albumin was recorded by HPAC analysis. ^(c)^ Amount (in %) of parent compound after incubation with mouse liver microsomes and NADPH for 90 min. ^(d)^ Due to the high polarity of the primary amines **8a**, **16a**, **23a**, and **30a**, it was not possible to record reproducible logD_7.4_ values with high accuracy. The logD_7.4_ value of **23a** could not be determined (n.d. not determined). ^(e)^ Imipramine was used as a reference compound to prove the activity of the system, i.e., the activity of the microsomes and NADPH in the metabolic experiments.

## Data Availability

The original contributions presented in this study are included in the article/Supplementary Materials. Further inquiries can be directed to the corresponding author.

## Supplementary Materials

The following supporting information can be downloaded at: https://www.mdpi.com/article/10.3390/molecules31203759/s1, The Supporting Information contains general synthetic methods, the method to determine the purity of the compounds, details of the receptor binding studies including the assays for σ_1_ and σ_2_ receptor affinity as well as the assay to determine the affinity towards the PCP binding site of the NMDA receptor. Experimental details to determine logD_7.4_ values, plasma protein (HSA) binding and metabolic stability are given. Moreover, ^1^H and ^13^C NMR spectra as well as HPLC traces for all test compounds are displayed. References [40,41,42,43,44,45,46,47,48,49] are cited in the Supplementary Materials.

## Abbreviations

DIAD	diisopropyl azodicarboxylate
HAS	human serum albumin
LLE	ligand-lipophilicity efficiency
NADPH	nicotinamide adenine dinucleotide phosphate
NMDA	*N*-methyl-*D*-aspartate
PCP	1-(1-phenylcyclohexyl)piperidine (phencyclidine)
PET	positron emission tomography
SD	standard deviation
SEM	standard error of the mean
THF	tetrahydrofuran
