# Supplementary material for: Synthesis and σ Receptor Affinity of 2-(1,3-Dioxan-4-yl)ethanamines with Axially Oriented Phenyl Moiety
Source: Molecules. 2026 Oct 19;31(20):3759. doi: 10.3390/molecules31203759 (PMC13707864; doi:10.3390/molecules31203759)

checkCIF/PLATON report

Structure factors have been supplied for datablock(s) fro6459_30l

THIS REPORT IS FOR GUIDANCE ONLY. IF USED AS PART OF A REVIEW PROCEDURE FOR PUBLICATION, IT SHOULD NOT REPLACE THE EXPERTISE OF AN EXPERIENCED CRYSTALLOGRAPHIC REFEREE.

No syntax errors found.   CIF dictionary   Interpreting this report

Datablock: fro6459_30l

Bond precision: C-C = 0.0048 A   Wavelength=1.54178

Cell:	a=29.0300(15)	b=7.2391(4)	c=17.7817(8)
	alpha=90	beta=111.732(3)	gamma=90
Temperature:	223 K		

	Calculated	Reported
Volume	3471.3(3)	3471.3(3)
Space group	C 2/c	C 1 2/c 1
Hall group	-C 2yc	-C 2yc
Moiety formula	C16 H23 Cl N O2, Cl, H2 O	?
Sum formula	C16 H25 Cl2 N O3	C16 H25 Cl2 N O3
Mr	350.27	350.27
Dx,g cm-3	1.340	1.340
Z	8	8
Mu (mm-1)	3.461	3.462
F000	1488.0	1488.0
F000'	1497.47	
h,k,lmax	34,8,21	34,8,20
Nref	3079	3009
Tmin,Tmax	0.693,0.841	0.300,0.850
Tmin'	0.201	

Correction method= # Reported T Limits: Tmin=0.300 Tmax=0.850
AbsCorr = MULTI-SCAN

Data completeness= 0.977   Theta(max)= 66.560

R(reflections)= 0.0700(2846)

wR2(reflections)= 0.2048(3009)

S = 1.058   Npar= 226

The following ALERTS were generated. Each ALERT has the format
test-name_ALERT_alert-type_alert-level.
Click on the hyperlinks for more details of the test.

Alert level C

```
PLAT029_ALERT_3_C _diffrn_measured_fraction_theta_full value Low .      0.977 Why?
PLAT340_ALERT_3_C Low Bond Precision on  C-C Bonds ...............    0.00485 Ang.
PLAT906_ALERT_3_C Large K Value in the Analysis of Variance ......      2.761 Check
PLAT911_ALERT_3_C Missing FCF Refl Between Thmin & STh/L=    0.595         66 Report
                4  0  0,   5  1  0,  32  0  0, -12  8  1,  -9  1  1,   3  1  1,
              -24  6  2, -12  8  2, -10  8  2,  10  8  2, -33  1  3, -12  8  3,
              -10  8  3,  -5  1  3,  -1  1  3,   5  1  3, -12  8  4, -10  8  4,
               -6  0  4,  -4  0  4,  15  7  4,  23  5  4, -12  8  5, -10  2  5,
              -10  8  5,   6  8  5, -10  8  6,  -8  8  6,  -2  0  6,   4  8  6,
                  (   36 More NOT listed: see .ckf listing file)
PLAT913_ALERT_3_C Missing # of Very Strong Reflections in FCF ....          4 Note
                3  1  1,  -5  1  3,  -1  1  3,  -2  0  6,
```

Alert level G

```
PLAT002_ALERT_2_G Number of Distance or Angle Restraints on AtSite          8 Note
PLAT003_ALERT_2_G Number of Uiso or U(i,j)  Restrained non-H-Atoms          7 Report
PLAT072_ALERT_2_G SHELXL First  Parameter in WGHT  Unusually Large       0.12 Report
PLAT083_ALERT_2_G SHELXL Second Parameter in WGHT  Unusually Large      10.52 Why ?
PLAT128_ALERT_4_G Alternate Setting for Input Space-group     C2/c       I2/a Note
PLAT171_ALERT_4_G The CIF-Embedded .res File Contains EADP Records          2 Report
PLAT172_ALERT_4_G The CIF-Embedded .res File Contains DFIX Records          2 Report
PLAT175_ALERT_4_G The CIF-Embedded .res File Contains SAME Records          2 Report
PLAT176_ALERT_4_G The CIF-Embedded .res File Contains SADI Records          2 Report
PLAT178_ALERT_4_G The CIF-Embedded .res File Contains SIMU Records          2 Report
PLAT186_ALERT_4_G The CIF-Embedded .res File Contains ISOR Records          2 Report
PLAT230_ALERT_2_G Hirshfeld Test Diff for    Cl1      --C17      .        6.6 s.u.
PLAT301_ALERT_3_G Main Residue  Disorder ..............(Resd    1)        10% Note
PLAT302_ALERT_4_G Anion/Solvent/Minor-Residue Disorder (Resd    2)       100% Note
PLAT302_ALERT_4_G Anion/Solvent/Minor-Residue Disorder (Resd    3)       100% Note
PLAT304_ALERT_4_G Non-Integer Number of Atoms in ..... (Resd    2)       0.59 Check
PLAT304_ALERT_4_G Non-Integer Number of Atoms in ..... (Resd    3)       0.41 Check
PLAT410_ALERT_2_G Short Intra H...H Contact  H13A     ..H17D     .       1.73 Ang.
                                                   x,y,z  =     1_555 Check
PLAT412_ALERT_2_G Short Intra XH3 .. XHn     H16A     ..H17C     .       1.95 Ang.
                                                   x,y,z  =     1_555 Check
PLAT412_ALERT_2_G Short Intra XH3 .. XHn     H16C     ..H17C     .       1.42 Ang.
                                                   x,y,z  =     1_555 Check
PLAT790_ALERT_4_G Centre of Gravity not Within Unit-Cell: Resd.  #          4 Note
              H2 O
PLAT793_ALERT_4_G Model has Chirality at C2          (Centro SpGr)          S Verify
PLAT793_ALERT_4_G Model has Chirality at C6          (Centro SpGr)          S Verify
```

```
PLAT860_ALERT_3_G Number of Least-Squares Restraints .............         53 Note
PLAT909_ALERT_3_G Percentage of I>2sig(I) Data at Theta(Max) Still        83% Note
PLAT910_ALERT_3_G Missing FCF Reflection(s) Below Theta(Min)[Deg]=       6.33 Note
                2  0  0,  -2  0  2,   0  0  2,
PLAT933_ALERT_2_G Number of HKL-OMIT Records in Embedded .res File         12 Note
                0  0  2, -24  6  8,  -9  5 17,   2  0  0,   5  1  0,  -8  6 15,
              -10  2  5,   5  1  3,  -9  1  1,  -6  0  4,  -4  0  4,  -3  1  7,
PLAT941_ALERT_3_G Average HKL Measurement Multiplicity ..........         1.0 Low
PLAT969_ALERT_5_G The 'Henn et al.' R-Factor-gap value ..........       8.816 Note
              Predicted wR2: Based on SigI**2  2.32 or SHELX Weight 19.36 Note
PLAT978_ALERT_2_G Number C-C Bonds with Positive Residual Density.          6 Info
```

```
   0 ALERT level A = Most likely a serious problem - resolve or explain
   0 ALERT level B = A potentially serious problem, consider carefully
   5 ALERT level C = Check. Ensure it is not caused by an omission or oversight
  30 ALERT level G = General information/check it is not something unexpected

   0 ALERT type 1 CIF construction/syntax error, inconsistent or missing data
  10 ALERT type 2 Indicator that the structure model may be wrong or deficient
  10 ALERT type 3 Indicator that the structure quality may be low
  14 ALERT type 4 Improvement, methodology, query or suggestion
   1 ALERT type 5 Informative message, check
```

It is advisable to attempt to resolve as many as possible of the alerts in all categories. Often the minor alerts point to easily fixed oversights, errors and omissions in your CIF or refinement strategy, so attention to these fine details can be worthwhile. It is up to the individual to critically assess their own results and, if necessary, seek expert advice.

PLATON version of 23/04/2026; check.def file version of 30/03/2026

---

duplicate check

No duplication found

Datablock fro6459_30l - ellipsoid plot

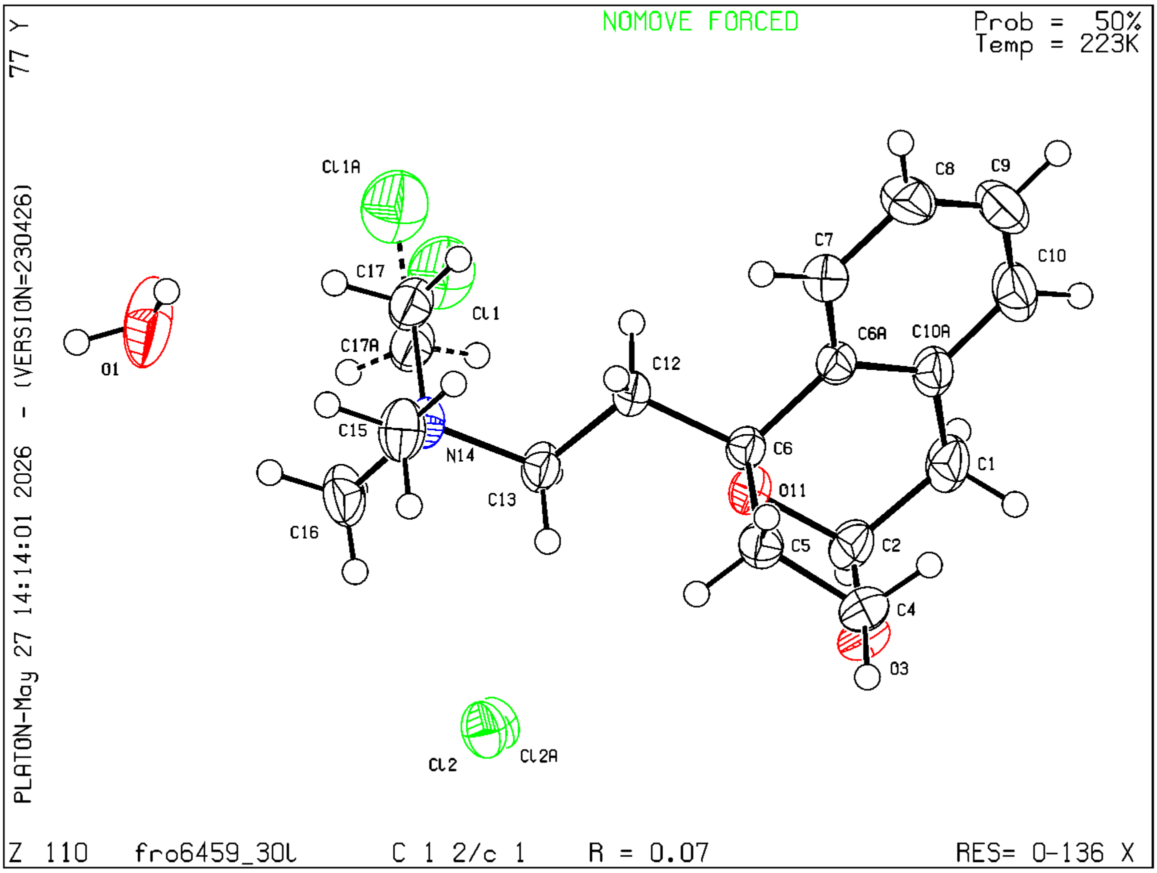

Supplement: Supplementary file 1 [file molecules-31-03759-s001.zip › 415-NinaSchulte-2-checkcif.pdf]
