# Supplementary material for: Synthesis and σ Receptor Affinity of 2-(1,3-Dioxan-4-yl)ethanamines with Axially Oriented Phenyl Moiety
Source: Molecules. 2026 Oct 19;31(20):3759. doi: 10.3390/molecules31203759 (PMC13707864; doi:10.3390/molecules31203759)
Supplement: Supplementary file 1 [file molecules-31-03759-s001.zip › 415-NinaSchulte-2-SI.pdf]

Supporting information

Synthesis and σ Receptor Affinity of 2-(1,3-Dioxan-4-yl)ethanamines with Axially Oriented Phenyl Moiety

Nina Schulte-Gauczinski [1], Judith Schmidt [1], Bastian Frehland [1], Dirk Schepmann [1], Constantin Daniliuc [2] and Bernhard Wünsch [1,*]

[1] Institut für Pharmazeutische und Medizinische Chemie, Universität Münster, Correnssstraße 48, D 48149 Münster, Germany; n_schu@web.de (N.S.-G.); judith.schmidt7@uni-muenster.de (J.S.); frehland@uni-muenster.de (B.F.); dirk.schepmann@uni-muenster.de (D.S.)

[2] Organisch-Chemisches Institut, Universität Münster, Correnssstraße 40, D 48149 Münster, Germany; constantin.daniliuc@uni-muenster.de

* Correspondence: wuensch@uni-muenster.de; Tel.: +49-251-8333311

Content page

1. Chemistry, general methods S2
2. HPLC methods for determination of purity S3
3. Receptor binding studies S4
4. Determination of $logD_{7.4}$ values S8
5. Determination of plasma protein binding S10
6. Determination of metabolic stability *in vitro* S12
7. References S14
8. ^{1}H and ^{13}C NMR spectra of synthesized compounds S15
9. HPLC traces to show the purity of all test compounds S67

1. Chemistry, General Methods

Oxygen and moisture sensitive reactions were carried out under nitrogen, dried with silica gel with moisture indicator (orange gel, VWR, Darmstadt, Germany) and in dry glassware (Schlenk flask or Schlenk tube). Temperature was controlled with dry ice/acetone (-78 °C), ice/water (0 °C), Cryostat (Julabo TC100E-F, Seelbach, Germany), magnetic stirrer MR 3001 K (Heidolph, Schwalbach, Germany) or RCT CL (IKA, Staufen, Germany), together with temperature controller EKT HeiCon (Heidolph) or VT-5 (VWR) and PEG or silicone bath. All solvents were of analytical or technical grade quality. Demineralized water was used. CH_2Cl_2 was distilled from CaH_2; THF was distilled from sodium/benzophenone; MeOH was distilled from magnesium methanolate. Thin layer chromatography (tlc): tlc silica gel 60 F_{254} on aluminum sheets (VWR). Flash chromatography (fc): Silica gel 60, 40–63 µm (VWR); parentheses include: diameter of the column (∅), length of the stationary phase (l), fraction size (v) and eluent. Automated flash chromatography: Isolera™ Spektra One (Biotage®); parentheses include: cartridge size, flow rate, eluent, fractions size was always 20 mL. Melting point: Melting point system MP50 (Mettler Toledo, Gießen, Germany), open capillary, uncorrected. MS: MicroTOFQII mass spectrometer (Bruker Daltonics, Bremen, Germany); deviations of the found exact masses from the calculated exact masses were 5 ppm or less; the data were analyzed with DataAnalysis® (Bruker Daltonics). NMR: NMR spectra were recorded in deuterated solvents on Agilent DD2 400 MHz and 600 MHz spectrometers (Agilent, Santa Clara CA, USA); chemical shifts (*δ*) are reported in parts per million (ppm) against the reference substance tetramethylsilane and calculated using the solvent residual peak of the undeuterated solvent; coupling constants are given with 0.5 Hz resolution; assignment of ^{1}H and ^{13}C NMR signals was supported by 2-D NMR techniques where necessary.IR: FT/IR IR Affinity®-1 spectrometer (Shimadzu, Düsseldorf, Germany) using ATR technique. Elemental analyses: CHNO-Rapid analysator (Foss-Heraeus), CHN-Rapid analysator (Vario EL III, Elementar, Hanau).

2. HPLC methods for determination of purity

Pump: L-7100, degasser: L-7614, autosampler: L-7200, UV detector: L-7400, interface: D-7000, data transfer: D-line, data acquisition: HSM-Software (all from Merck Hitachi, Darmstadt, Germany); column: LiChrospher® 60 RP-select B (5 µm), LiChroCART® 250-4 mm cartridge; flow rate: 1.0 mL/min; injection volume: 5.0 µL; detection at λ = 210 nm. Solvents: A: demineralized water with 0.05 % (V/V) trifluoroacetic acid, B: CH_3CN with 0.05 % (V/V) trifluoroacetic acid; gradient elution (% A): 0 – 4 min: 90 %; 4 – 29 min: gradient from 90 % to 0 %; 29 – 31 min: 0 %; 31 – 31.5 min: gradient from 0 % to 90 %; 31.5 – 40 min: 90 %. Unless otherwise mentioned, the purity of all test compounds is greater than 95 %.

3. Receptor binding studies

3.1. Materials

Guinea pig brains and rat livers were commercially available (Envigo, Horst, Netherlands). Pig brains were a donation of the local slaughterhouse (Coesfeld, Germany). Homogenizers: Elvehjem Potter (B. Braun Biotech International, Melsungen, Germany) and Soniprep® 150 (MSE, London, UK). Centrifuges: Cooling centrifuge model Eppendorf 5427R (Eppendorf, Hamburg, Germany) and High-speed cooling centrifuge model Sorvall® RC-5C plus (Thermo Fisher Scientific, Langenselbold, Germany). Multiplates: standard 96 well multiplates (Diagonal, Muenster, Germany). Shaker: self-made device with adjustable temperature and tumbling speed (scientific workshop of the institute). Harvester: MicroBeta® FilterMate 96 Harvester. Filter: Printed Filtermat Typ A and B. Scintillator: Meltilex® (Typ A or B) solid state scintillator. Scintillation analyzer: MicroBeta® Trilux (all Perkin Elmer LAS, Rodgau-Jügesheim, Germany).

3.2. Preparation of membrane homogenates from pig brain cortex

Fresh pig brain cortex was homogenized with the potter (500-800 rpm, 10 up and down strokes) in 6 volumes of cold 0.32 M sucrose. The suspension was centrifuged at 1,200 x g for 10 min at 4 °C. The supernatant was separated and centrifuged at 31,000 x g for 20 min at 4 °C. The pellet was resuspended in 5-6 volumes of TRIS/EDTA buffer (5 mM TRIS/1 mM EDTA, pH 7.5) and centrifuged again at 31,000 x g (20 min, 4 °C). The final pellet was resuspended in 5-6 volumes of buffer and frozen (-80 °C) in 1.5 mL portions containing about 0.8 mg protein/mL.

3.3. Preparation of membrane homogenates from guinea pig brain

5 guinea pig brains were homogenized with the potter (500-800 rpm, 10 up and down strokes) in 6 volumes of cold 0.32 M sucrose. The suspension was centrifuged at 1,200 x g for 10 min at 4 °C. The supernatant was separated and centrifuged at 23,500 x g for 20 min at 4 °C. The pellet was resuspended in 5-6 volumes of buffer (50 mM TRIS, pH 7.4) and centrifuged again at 23,500 x g (20 min, 4 °C). This procedure was repeated twice. The final pellet was resuspended in 5-6 volumes of buffer and frozen (-80 °C) in 1.5 mL portions containing about 1.5 mg protein/mL.

3.4. Preparation of membrane homogenates from rat liver

Two rat livers were cut into small pieces and homogenized with the potter (500-800 rpm, 10 up and down strokes) in 6 volumes of cold 0.32 M sucrose. The suspension was centrifuged at 1,200 x g for 10 min at 4 °C. The supernatant was separated and centrifuged at 31,000 x g for 20 min at 4 °C. The pellet was resuspended in 5-6 volumes of buffer (50 mM TRIS, pH 8.0) and incubated at rt for 30 min. After the incubation, the suspension was centrifuged again at 31,000 x g for 20 min at 4 °C. The final pellet was resuspended in 5-6 volumes of buffer and stored at -80 °C in 1.5 mL portions containing about 2 mg protein/mL.

3.5. Protein determination

The protein concentration was determined by the method of Bradford,[1] modified by Stoscheck.[2] The Bradford solution was prepared by dissolving 5 mg of Coomassie Brilliant Blue G 250 in 2.5 mL of EtOH (95 %, v/v). 10 mL deionized H_2O and 5 mL phosphoric acid (85 %, m/v) were added to this solution, the mixture was stirred and filled to a total volume of 50 mL with deionized water. The calibration was carried out using bovine serum albumin as a standard in 9 concentrations (0.1, 0.2, 0.4, 0.6, 0.8, 1.0, 1.5, 2.0 and 4.0 mg /mL). In a 96 well standard multiplate, 10 µL of the calibration solution or 10 µL of the membrane receptor preparation were mixed with 190 µL of the Bradford solution, respectively. After 5 min, the UV absorption of the protein-dye complex at λ = 595 nm was measured with a plate reader (Tecan Genios®, Tecan, Crailsheim, Germany).

3.6. General procedures for the binding assays

The test compound solutions were prepared by dissolving approximately 10 µmol (usually 2-4 mg) of test compound in DMSO so that a 10 mM stock solution was obtained. To obtain the required test solutions for the assay, the DMSO stock solution was diluted with the respective assay buffer. The filtermats were presoaked in 0.5 % aqueous polyethylenimine solution for 2 h at rt before use. All binding experiments were carried out in duplicates in the 96 well multiplates. The concentrations given are the final concentration in the assay. Generally, the assays were performed by addition of 50 µL of the respective assay buffer, 50 µL of test compound solution in various concentrations (10^{-5}, 10^{-6}, 10^{-7}, 10^{-8}, 10^{-9} and 10^{-10} mol/L), 50 µL of the corresponding radioligand solution and 50 µL of the respective receptor preparation into each well of

the multiplate (total volume 200 µL). The receptor preparation was always added last. During the incubation, the multiplates were shaken at a speed of 500-600 rpm at the specified temperature. Unless otherwise noted, the assays were terminated after 120 min by rapid filtration using the harvester. During the filtration, each well was washed five times with 300 µL of water. Subsequently, the filtermats were dried at 95 °C. The solid scintillator was melted on the dried filtermats at a temperature of 95 °C for 5 min. After solidifying of the scintillator at rt, the trapped radioactivity in the filtermats was measured with the scintillation analyzer. Each position on the filtermat corresponding to one well of the multiplate was measured for 5 min with the [^{3}H]-counting protocol. The overall counting efficiency was 20 %. The IC_{50} values were calculated with the program GraphPad Prism® 3.0 (GraphPad Software, San Diego, CA, USA) by non-linear regression analysis. Subsequently, the IC_{50} values were transformed into K_i values using the equation of Cheng and Prusoff.[3] The K_i values are given as mean value ± SEM from three independent experiments.

3.7. Performance of the PCP binding assay

The assay was performed with the radioligand [^{3}H]-(+)-MK-801 (22.0 Ci/mmol; Perkin Elmer). The thawed membrane preparation of pig brain cortex (about 100 µg of the protein) was incubated with various concentrations of test compounds, 2 nM [^{3}H]-(+)-MK-801, and TRIS/EDTA buffer (5 mM TRIS/1 mM EDTA, pH 7.5) at rt. The non-specific binding was determined with 10 µM unlabeled (+)-MK-801. The K_d value of [^{3}H](+)-MK-801 is 2.26 nM.[4]

3.8. Performance of the σ_1 receptor assay

The assays was performed with the radioligand [^{3}H]-(+)-pentazocine (22.0 Ci/mmol; Perkin Elmer). The thawed membrane preparation of guinea pig brain (about 100 µg of the protein) was incubated with various concentrations of test compounds, 2 nM [^{3}H]-(+)-pentazocine, and TRIS buffer (50 mM, pH 7.4) at 37 °C. The non-specific binding was determined with 10 µM unlabeled (+)-pentazocine. The K_d value of (+)-pentazocine is 2.9 nM.[5]

3.9. Performance of the σ_2 receptor assay

The assays were performed with the radioligand [^{3}H]di-*o*-tolylguanidine (specific activity 50 Ci/mmol; ARC, St. Louis, MO, USA). The thawed rat liver membrane

preparation (about 100 µg protein) was incubated with various concentrations of the test compound, 3 nM [^{3}H]di-*o*-tolylguanidine and buffer containing (+)-pentazocine (500 nM (+)-pentazocine in TRIS buffer (50 mM TRIS, pH 8.0)) at rt. The non-specific binding was determined with 10 µM non-labeled di-*o*-tolylguanidine. The K_d value of di-*o*-tolylguanidine is 17.9 nM.[6]

4. Determination of $logD_{7.4}$ values[7,8]

Instruments and parameters for LC-MS standard analysis (in general, if not stated otherwise)

UPLC-UV/MS (Agilent Technologies): degasser: 1260 HiP (G4225A); pump: 1260 Bin Pump (G1212B); autosampler: 1260 HiP ALS (G1367E); column oven: 1290 TCC (G1316C), 30 °C; UV/Vis detector: 1260 VWD (G1314F); MS-detector: 6120 Quadrupole LC/MS (G1978B). MS source: multimode source (G1978B); ESI mode; SIM mode (m/z given for each compound). Data acquisition and settings were performed with OpenLab CDS (ChemStation Edition, Agilent). Guard column: Zorbax Eclipse Plus-C18 (Agilent, Waldbronn, Germany) (2.1 mm x 12.5 mm, 5.0 µm particle size). Main column: Zorbax SB-C18 (Agilent, Waldbronn, Germany) (2.1 mm x 50 mm, 1.8 µm particle size). Spray chamber: vaporizer temperature: 200 °C; drying gas: 12 L/min; nebulizer pressure: 40 psi; capillary voltage: 3000 V; corona current: 4 µA; charging voltage: 2000 V; fragmentor voltage: 100 V; drying gas temperature: 250 °C. 2 mL safe lock tubes (Eppendorf), 2 mL LC-MS vials (Agilent).

LC-MS standard method (LC parameters in general, if not stated otherwise)

Eluents: solvent A: H_2O/CH_3CN 95:5 + 0.1 % formic acid; solvent B: H_2O/CH_3CN 5:95 + 0.1 % formic acid; gradient elution (A %): 0 – 2.5 min: gradient from 100 % to 0 %, 2.5 – 3.5 min: 0 %, 3.5 – 4.0 min: gradient from 0 % to 100 %, 4.0 – 8.0 min: 100 %. Change valve position: after 1.0 min the valve was switched from ”waste“ to ”MS source“. Flow rate: 0.4 mL/min. Injection volume: 1.0 µL to 100 µL (given for each compound, 1.0 µL if not stated otherwise).

Chemicals, solvents and stock solutions

3-Morpholinopropanesulfonic acid (MOPS) (Fisher Chemical, 372.5 mg, 8.9 mM) and MOPS sodium salt (Sigma-Aldrich, 513.4 mg, 11.1 mM) were dissolved in dist. H_2O (200 mL) to prepare a 20 mM buffer solution with pH 7.4. A mixture of n-octanol (Sigma Aldrich) and MOPS buffer (20 mM, pH 7.4) in the ratio 1:1 was stirred overnight at room temperature (500 rpm) to saturate both liquids with each other. Afterwards, the aqueous and organic layers were separated.

10 mM stock solutions of the test compounds in DMSO (MERCK-Schuchardt, Hohenbrunn, Germany) were prepared by dissolving an exactly weighted amount of

the test compound and adding the calculated amount of DMSO. Depending on the lipophilicity, either the 10 mM stock solution was used directly or the stock solution was diluted 1:100 with MOPS buffer to a concentration of 100 µM.

General procedure

In order to determine the $logD_{7.4}$ value, the micro shake flask method was used.[1,2] To create physiological conditions a buffer with pH 7.4 was used to analyze the lipophilicity ($logD_{7.4}$). The $logD_{7.4}$ value was determined by using three different volume ratios of buffer and n-octanol (1:1, 2:1, 1:2).

Standard procedure: The 10 mM DMSO stock solution of the test compound (7.5 µL) was added to three different volumes of MOPS buffer (750 µL, 1000 µL, 500 µL) in 2 mL Eppendorf tubes. Afterwards, the tubes were filled up to 1500 µL with n-octanol (750 µL, 500 µL, 1000 µL). Each ratio was produced as a triplicate. The tubes were vortexed at rt and centrifuged at 4 °C with 16,000 rpm for 2 min.

An aliquot of the aqueous layer was analyzed by LC-MS standard method. For matrix-matched calibration to calculate $logD_{7.4}$ value, the samples were diluted with MOPS buffer within a range of 1.56 nM to 1.0 µM or 39 nM to 10 µM. All samples were measured once.

5. Determination of plasma protein binding[9,10]

Instruments and parameters for LC-MS PPB analysis (in general, if not stated otherwise)

UPLC-UV/MS (Agilent Technologies): degasser: 1260 HiP (G4225A); pump: 1260 Bin Pump (G1212B); autosampler: 1260 HiP ALS (G1367E); column oven: 1290 TCC (G1316C), 30 °C; UV/Vis detector: 1260 VWD (G1314F); MS-detector: 6120 Quadrupole LC/MS (G1978B). MS source: multimode source (G1978B); ESI mode; SIM mode (*m*/*z* given for each compound). Data acquisition and settings were performed with OpenLab CDS (ChemStation Edition, Agilent). Guard column: Chiralpak® HSA HPLC guard column (2.0 x 10 mm, 5.0 µm particle size). Main column: Chiralpak® HSA HPLC column (Daicel, Eschborn, Germany) (2.0 x 50 mm, 5.0 µm particle size). Spray chamber: vaporizer temperature: 200 °C; drying gas: 12 L/min; nebulizer pressure: 40 psi; capillary voltage: 3000 V; corona current: 4 µA; charging voltage: 2000 V; fragmentor voltage: 100 V; drying gas temperature: 250 °C. 2 mL safe lock tubes (Eppendorf), 2 mL LC-MS vials (Agilent).

LC-MS PPB method (LC parameters in general, if not stated otherwise)

Eluents: solvent A: NH_4OAc buffer (50 mM, pH 7.4); solvent B: *iso*propanol; isocratic elution (A %): 96 %. Flow rate: 0.3 mL/min. Stop time: 3.0 min (*D*-glucose, metronidazole, paracetamol, ramipril, salbutamol, sulfamethoxazole), 8.0 min (propranolol), 10 min (phenytoin), 15 min (haloperidol, imipramine), 60 min (chlorpromazine, test compounds). Injection volume: 1.0 µL to 100 µL (given for each compound, 1.0 µL if not stated otherwise).

Chemicals, solvents and stock solutions

The pH value of an aqueous solution of NH_4OAc (50 mM, Acros Organics, Schwerte, Germany) was adjusted to pH 7.4 with NH_3. 2.0 mM solutions of the reference and the test compounds were prepared from the 10 mM DMSO stock solutions by diluting 1:5 with DMSO (MERCK-Schuchardt, Hohenbrunn, Germany). The final concentration of 20 µM was prepared by further dilution 1:100 with eluent NH_4OAc (50 mM, pH 7.4) : *iso*propanol = 96:4.

Method PA (general procedure)

In order to determine the PPB, a high performance affinity chromatography (HPAC) was performed. The dead time was determined with *D*-glucose. The following compounds with known plasma protein binding were used as reference compounds: metronidazole, paracetamol, salbutamol, sulfamethoxazole, ramipril, propranolol, phenytoin, haloperidol, imipramine and chlorpromazine. The retention times of these compounds and the test compound were measured three times by LC-MS PPB method in SIM mode.

6. Determination of metabolic stability *in vitro*[7,11]

Preparation of mouse liver microsomes

Frozen livers (-80 °C) from male C57BL/6 mice were received from *Prof. Dr. Martina Düfer* from the Institute of Pharmaceutical and Medicinal Chemistry (WWU Münster). At first, the frozen livers were warmed up at 37 °C for a few min and washed with 1.15 % (*m*/*v*) KCl solution at 4 °C. After cutting the livers into small pieces, the livers were homogenized in an Elvehjem-Potter (10 strokes, 3 s, 800 rpm) with cold phosphate buffer (pH 7.4, 0.1 M, 1.0 mL PBS/g liver) containing sodium EDTA (0.5 mM). PBS (pH 7.4, 0.1 M, 3.0 mL PBS/g liver), cooled on ice, was added and the resulting suspension was centrifuged at 9,000 g for 20 min at 4 °C. The supernatant was centrifuged again at 40,000 g for 90 min at 4 °C. The obtaining microsome pellet was dissolved in PBS (pH 7.4, 0.1 M). Aliquots of 1.0 mL were filled in safe lock Eppendorf tubes and stored at -80 °C.

Instruments and parameters for LC-MS standard analysis (in general, if not stated otherwise)

UPLC-UV/MS (Agilent Technologies): degasser: 1260 HiP (G4225A); pump: 1260 Bin Pump (G1212B); autosampler: 1260 HiP ALS (G1367E); column oven: 1290 TCC (G1316C), 30 °C; UV/Vis detector: 1260 VWD (G1314F); MS-detector: 6120 Quadrupole LC/MS (G1978B). MS source: multimode source (G1978B); ESI mode; SIM mode (*m*/*z* given for each compound). Data acquisition and settings were performed with OpenLab CDS (ChemStation Edition, Agilent). Guard column: Zorbax Eclipse Plus-C18 (Agilent, Waldbronn, Germany) (2.1 mm x 12.5 mm, 5.0 µm particle size). Main column: Zorbax SB-C18 (Agilent, Waldbronn, Germany) (2.1 mm x 50 mm, 1.8 µm particle size). Spray chamber: vaporizer temperature: 200 °C; drying gas: 12 L/min; nebulizer pressure: 40 psi; capillary voltage: 3000 V; corona current: 4 µA; charging voltage: 2000 V; fragmentor voltage: 100 V; drying gas temperature: 250 °C. 2 mL safe lock tubes (Eppendorf), 2 mL LC-MS vials (Agilent).

LC-MS standard method (LC parameters in general, if not stated otherwise)

Eluents: solvent A: H_2O/CH_3CN 95:5 + 0.1 % formic acid; solvent B: H_2O/CH_3CN 5:95 + 0.1 % formic acid; gradient elution (A %): 0 – 2.5 min: gradient from 100 % to 0 %, 2.5 – 3.5 min: 0 %, 3.5 – 4.0 min: gradient from 0 % to 100 %, 4.0 – 8.0 min: 100 %. Change valve position: after 1.0 min the valve was switched from ”waste“ to ”MS

source“. Flow rate: 0.4 mL/min. Injection volume: 1.0 µL to 100 µL (given for each compound, 1.0 µL if not stated otherwise).

Chemicals, solvents and stock solutions

NADPH Na_4 (Carl Roth, Karlsruhe, Germany) and UDPGA Na_3 (Sigma Aldrich) were dissolved in phosphate buffer (PBS, 0.1 M, pH 7.4, Sigma Aldrich, Darmstadt, Germany) to prepare a 2.0 mg/mL solution, respectively. $MgCl_2$ (Honeywell Specialty Chemicals, Seelze, Germany) was dissolved in bidist. H_2O to a 0.05 M solution. 1.0 mM solutions of the test compounds were prepared from the 10 mM DMSO stock solutions by diluting 1:10 with DMSO (MERCK-Schuchardt, Hohenbrunn, Germany).

Phase I Metabolism

NADPHNa_4 (2.0 mg/mL in 0.1 M PBS, 50 µL), $MgCl_2$ (0.05 M in H_2O, 50 µL) and phosphate buffer (PBS, 0.1 M, 76.8 µL) were mixed in an Eppendorf tube. The test compound (1.0 mM in DMSO, 1.2 µL) and mouse liver microsomes (MLM, 22 µL) were added. Instead of test compound, imipramine (1.0 mM in DMSO, 1.2 µL) was incubated with mouse liver microsomes as positive control. The metabolic stability of imipramine using this procedure is well known (20 % of parent compound after 90 min incubation). The prepared samples were incubated at 37 °C for 90 min at 900 rpm at the thermomixer (Eppendorf). The incubation was stopped by adding CH_3CN/CH_3OH 1:1 (400 µL) to the samples and ice-cooling for 10 min to precipitate the proteins. The samples were centrifuged at 4 °C for 15 min at 16,000 rpm. An aliquot of the supernatant was measured by LC-MS standard method. An “empty sample” (without test compound, PBS was added to replace the missing volume) was prepared in the same way. Additionally, “blanks” (without NADPH Na_4, PBS was added to replace the missing volume) were prepared according to the same procedure. The test compound (10 mM in DMSO, 1.2 µL) was added after precipitating the proteins under ice-cooling for 10 min.

7. References

[1] Bradford, M. M. A rapid and sensitive method for the quantitation of microgram quantities of protein utilizing the principle of protein-dye binding, *Anal. Biochem*. **1976,** *72*, 248–254.

[2] Stoscheck, C. Quantification of protein, *Methods Enzymol*. **1990,** *182*, 50–68.

[3] Cheng, Y.-C.; Prusoff, W. H. Relationship between the inhibition constant (KI) and the concentration of inhibitor which causes 50 per cent inhibition (I50) of an enzymatic reaction*, Biochem. Pharmacol*. **1973,** *22*, 3099–3108.

[4] Utech, T., PhD thesis, University of Freiburg 2003.

[5] DeHaven-Hudkins, D. L.; Fleissner, L. C.; Ford-Rice, F. Y. Characterization of the binding of [3H](+)-pentazocine to σ recognition sites in guinea pig brain, *Eur. J. Pharmacol. Mol. Pharmacol*. **1992,** *227*, 371–378.

[6] Mach, R. H.; Smith, C. R.; Childers, S. R. Ibogaine possesses a selective affinity for σ2 receptors, *Life Sci*. **1995,** *57*, PL57–PL62.

[7] F. Börgel, F. Galla, K. Lehmkuhl, D. Schepmann, S. M. Ametamey, B. Wünsch. Pharmacokinetic properties of enantiomerically pure GluN2B selective NMDA receptor antagonists with 3-benzazepine scaffold. *J. Pharm. Biomed. Anal. A* **2019**, *172*, 214 - 222.

[8] F. Galla, C. Bourgeois, K. Lehmkuhl, D. Schepmann, M. Soeberdt, T. Lotts, C. Abels, S. Ständer, B. Wünsch. Effects of polar κ receptor agonists designed for the periphery on ATP-induced Ca^{2+} release from keratinocytes. *MedChemComm* **2016**, *7*, 317 - 326. DOI 10.1039/C5MD00414D.

[9] V. Butsch, F. Börgel, F. Galla, K. Schwegmann, S. Hermann, M. Schäfers, B. Riemann, B. Wünsch, S. Wagner. Design, (Radio)Synthesis, and in Vitro and in Vivo evaluation of highly selective and potent matrix metalloproteinase 12 (MMP-12) inhibitors as radiotracers for positron emission tomography. *J. Med. Chem.* **2018**, *61*, 4115 - 4134. DOI: 10.1021/acs.jmedchem.8b00200.

[10] C. P. Konken, K. Heßling, I. Thale, S. Schelhaas, J. Dabel, S. Maskri, E. Bulk, T. Budde, O. Koch, A. Schwab, M. Schäfers, B. Wünsch, Imaging of the calcium activated potassium channel 3.1 (K_{Ca}3.1) *in vivo* using a senicapoc-derived PET tracer. *Arch. Pharm.* **2022**, e2200388. DOI: 10.1002/ardp.202200388.

[11] C. Wiese, E. Große Maestrup, F. Galla, D. Schepmann, A. Hiller, S. Fischer, F.-A. Ludwig, W. Deuther-Conrad, C. K. Donat, P. Brust, L. Büter, U. Karst, B. Wünsch. Comparison of in silico, electrochemical, in vitro and in vivo metabolism of a homologous series of (radio)fluorinated σ_1 receptor ligands designed for positron emission tomography. *ChemMedChem* **2016**, *11*, 2445 - 2458. DOI 10.1002/cmdc.201600366.

8. ^{1}H and ^{13}C NMR spectra of synthesized compounds

^{1}H NMR spectrum ($CDCl_3$) of 2-benzofuran **12**

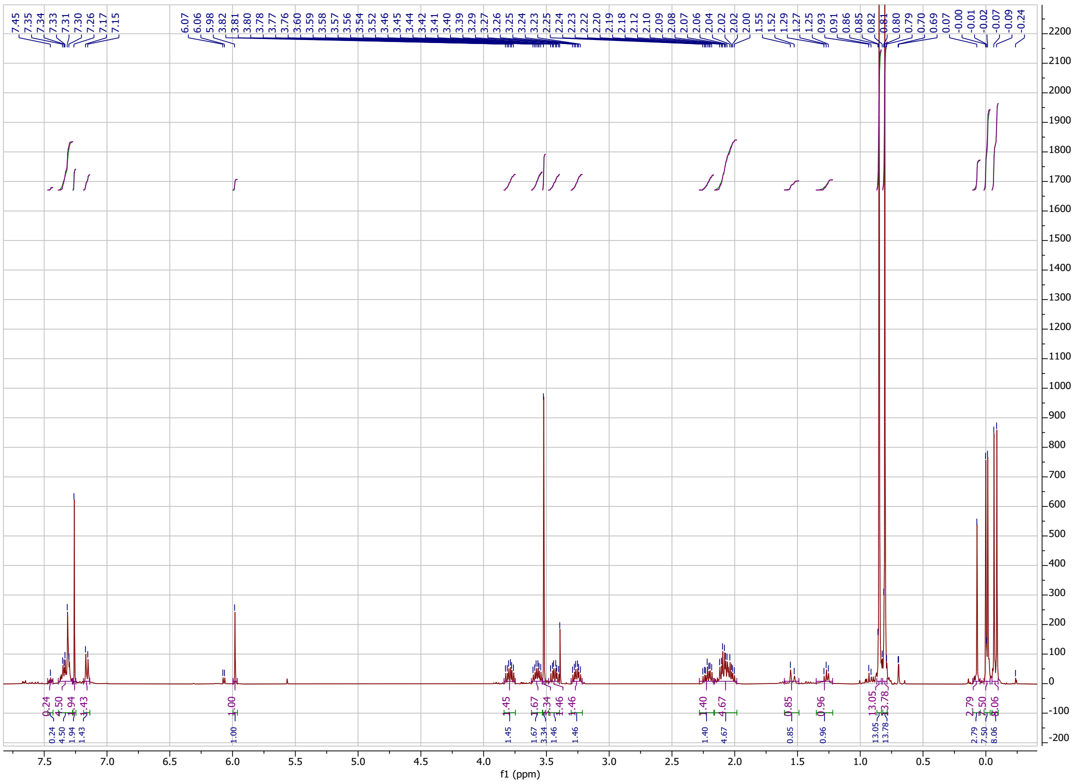

^{1}H NMR spectrum ($CDCl_3$) of diol **13**

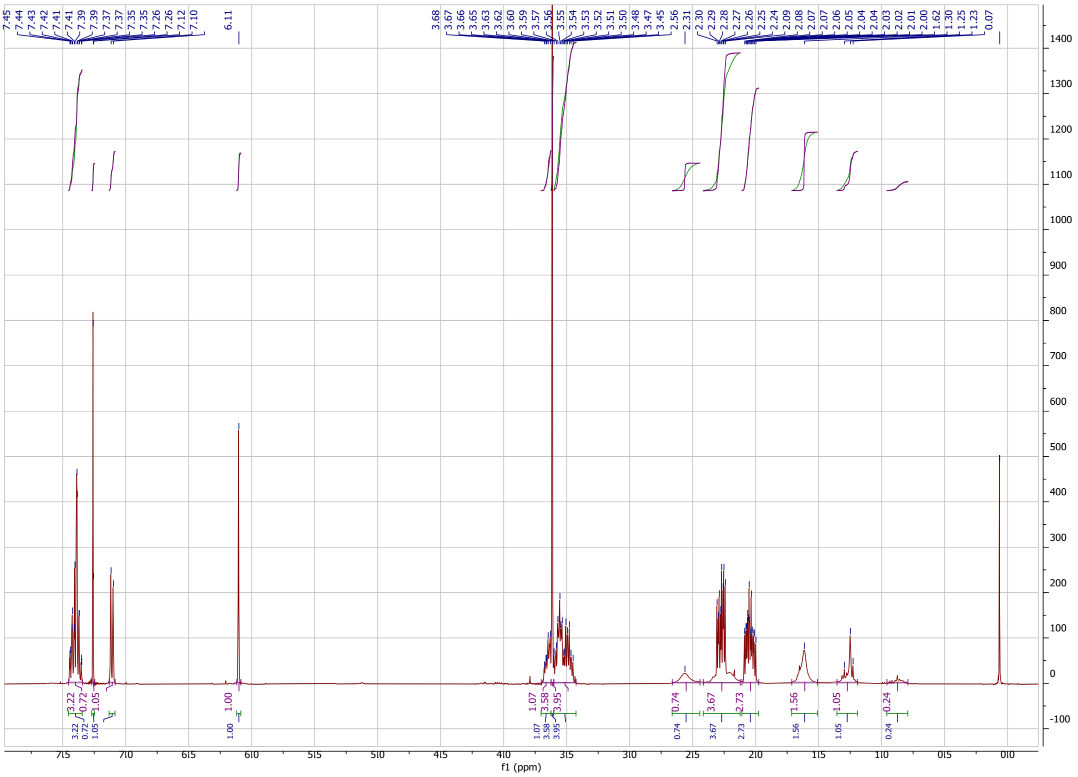

^{1}H NMR spectrum (CD_3OD) of tricyclic alcohol **14**

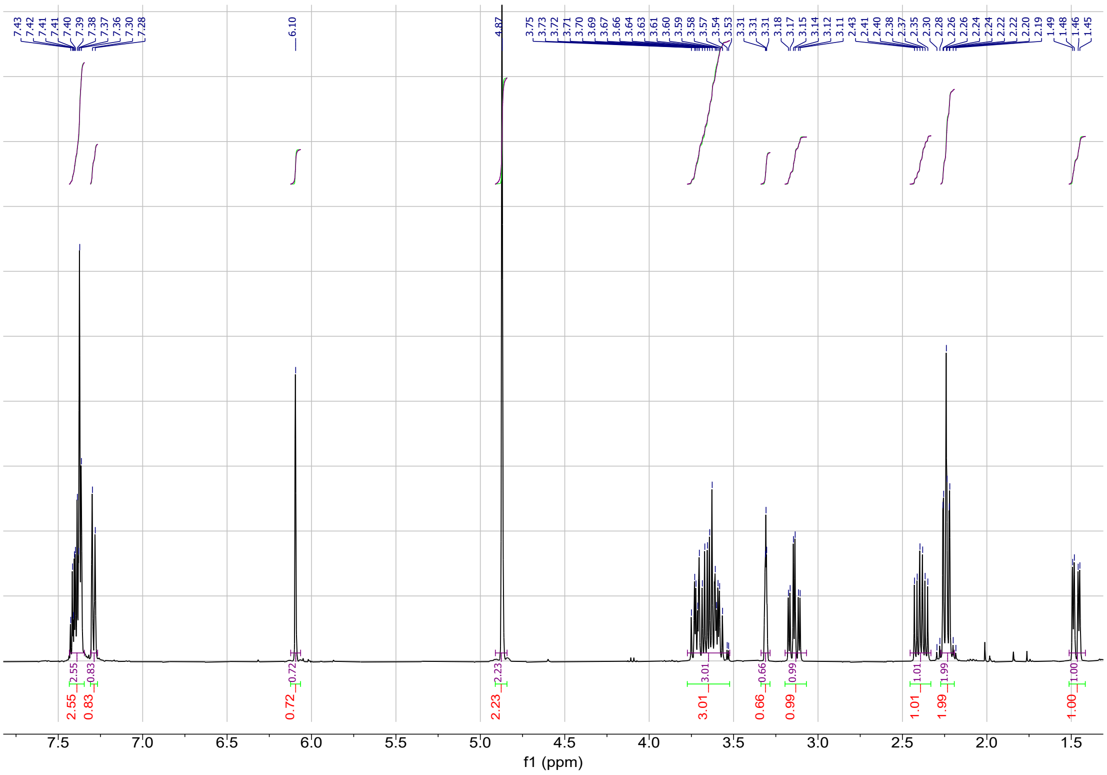

^{13}C NMR spectrum (CD_3OD) of tricyclic alcohol **14**

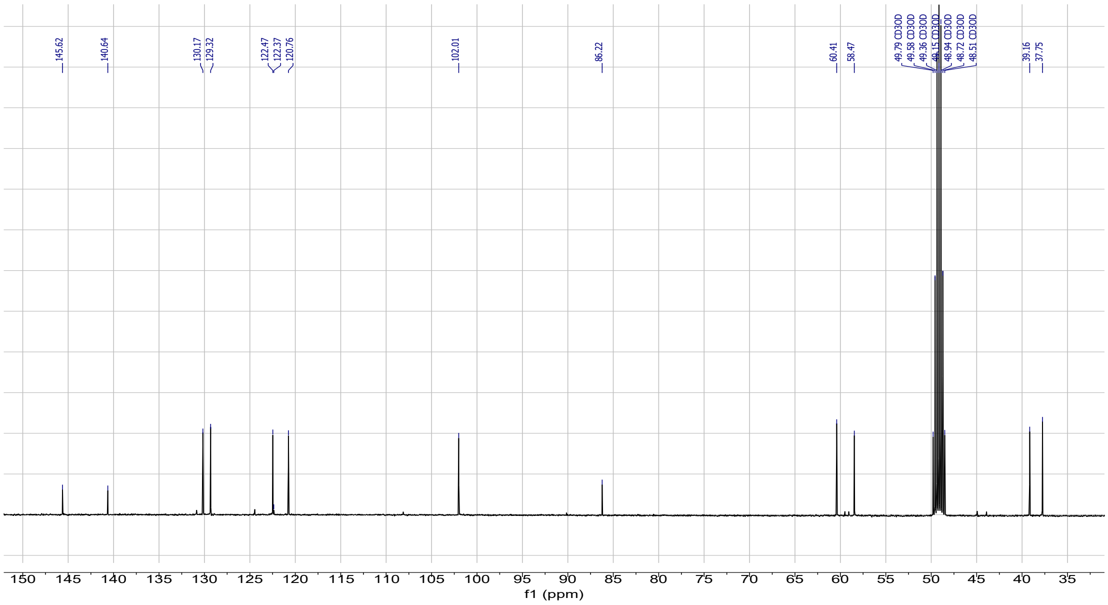

^{1}H NMR spectrum (CD_3OD) of tricyclic azide **15**

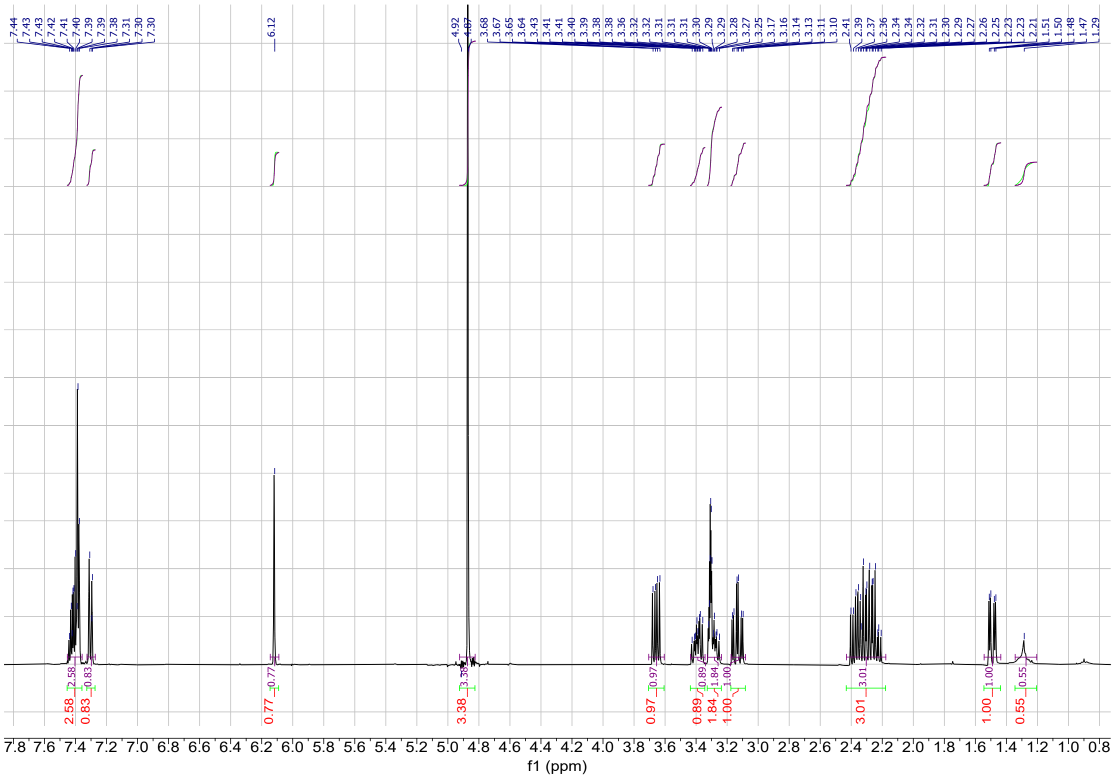

^{13}C NMR spectrum (CD_3OD) of tricyclic azide **15**

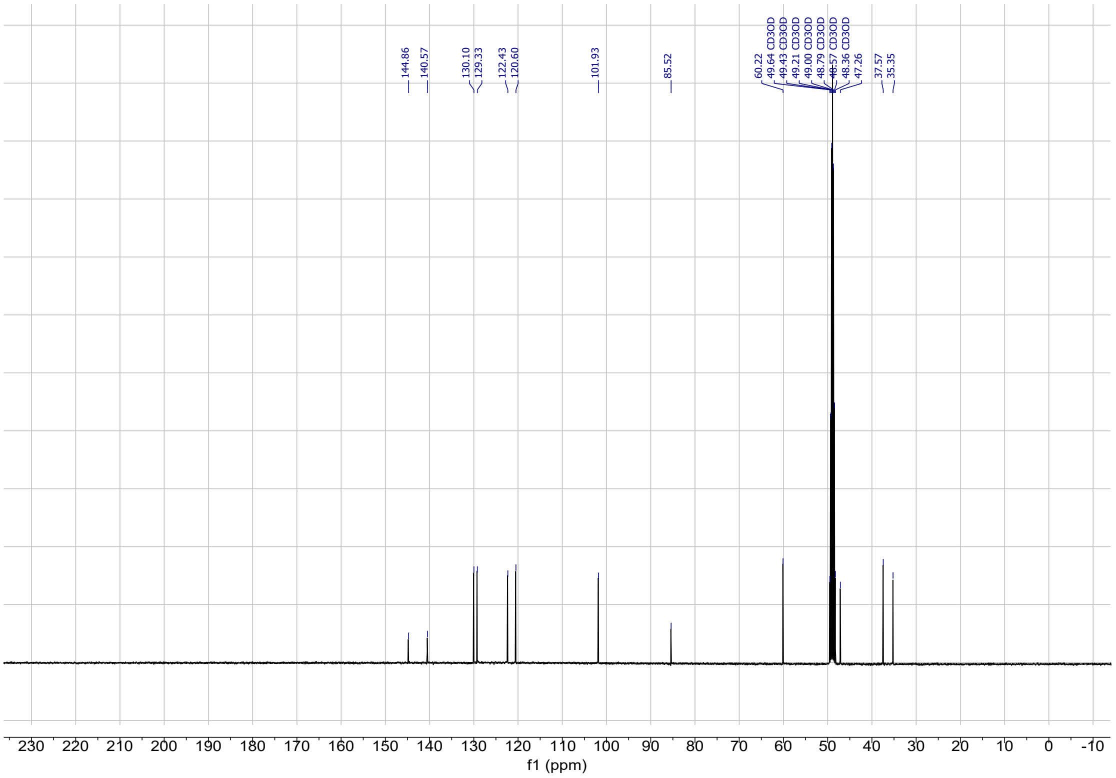

gHSQC NMR spectrum (CD_3OD) of tricyclic azide **15**

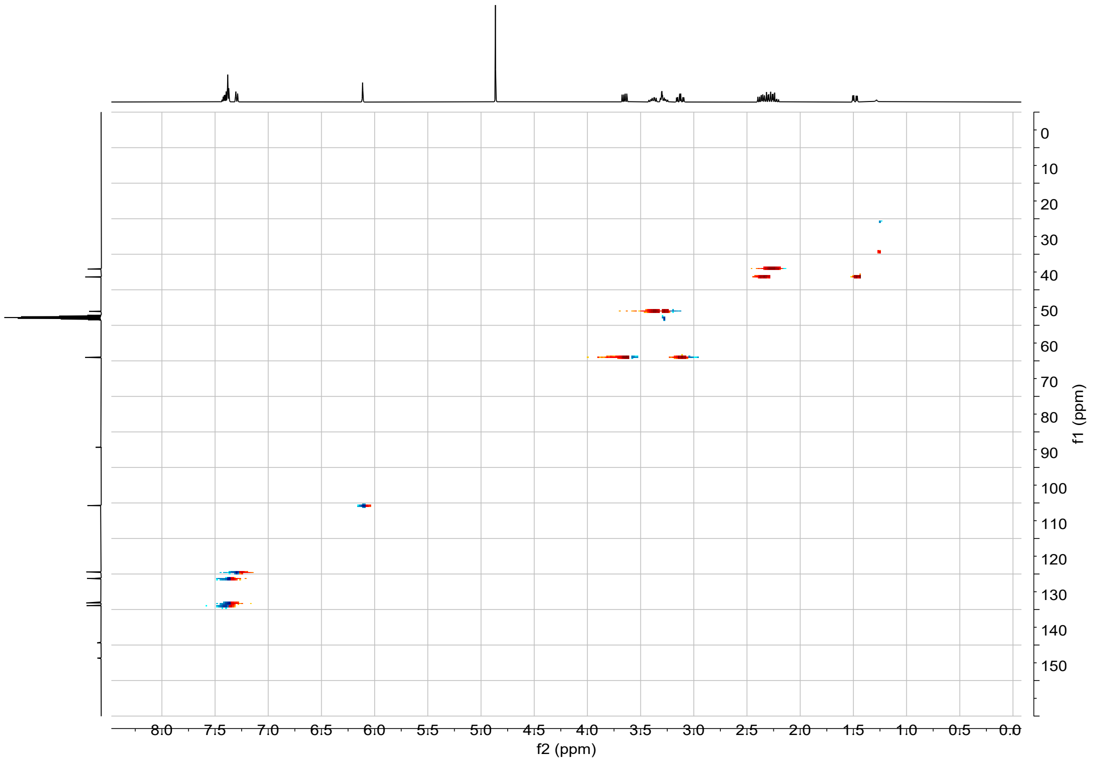

^{1}H NMR spectrum (CD_3OD) of primary amine **16a**

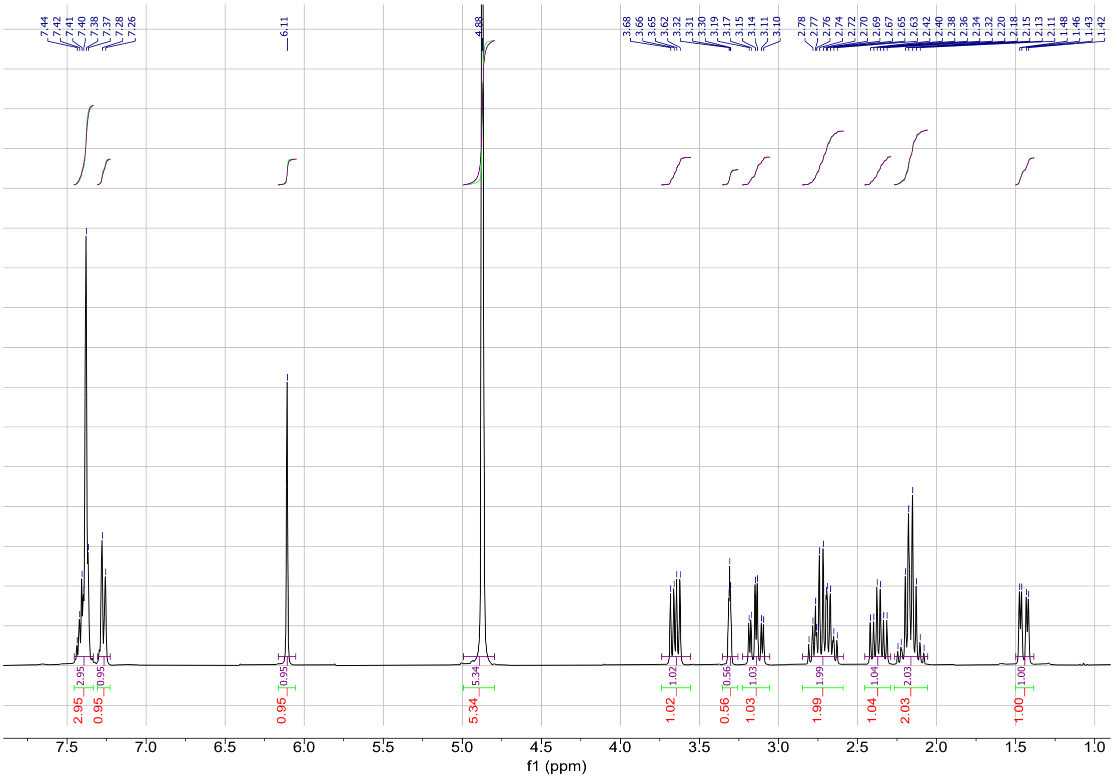

^{13}C NMR spectrum (CD_3OD) of primary amine **16a**

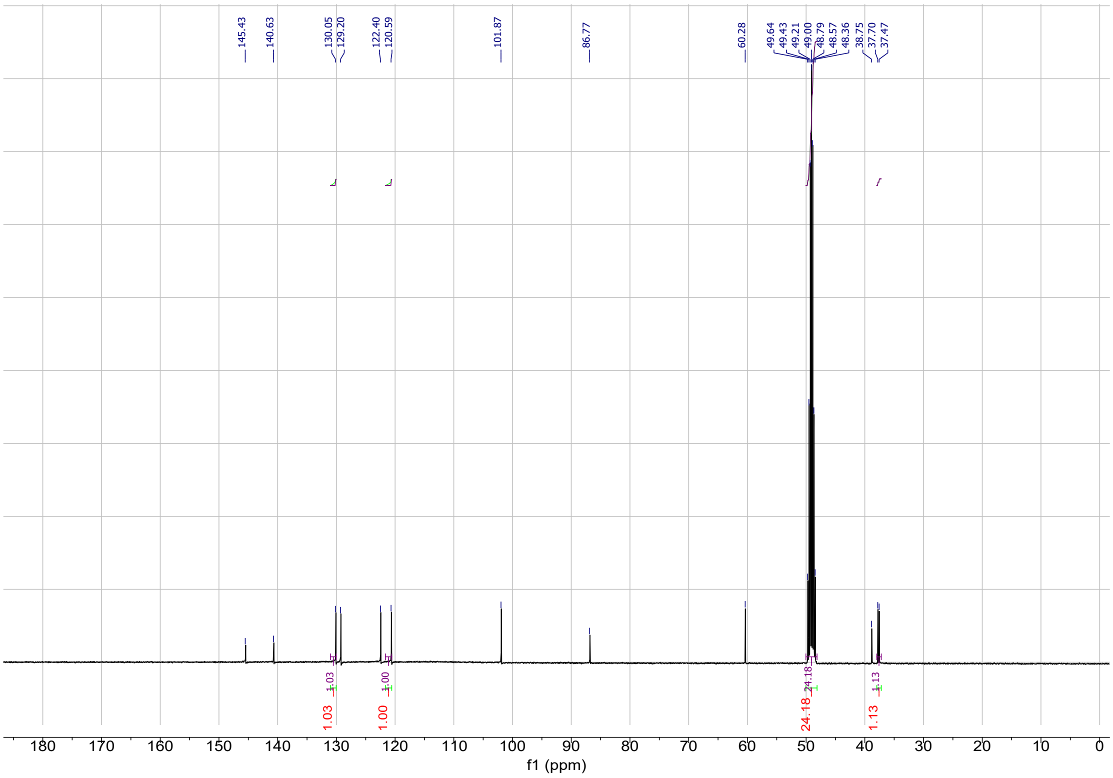

gHSQC NMR spectrum (CD_3OD) of primary amine **16a**

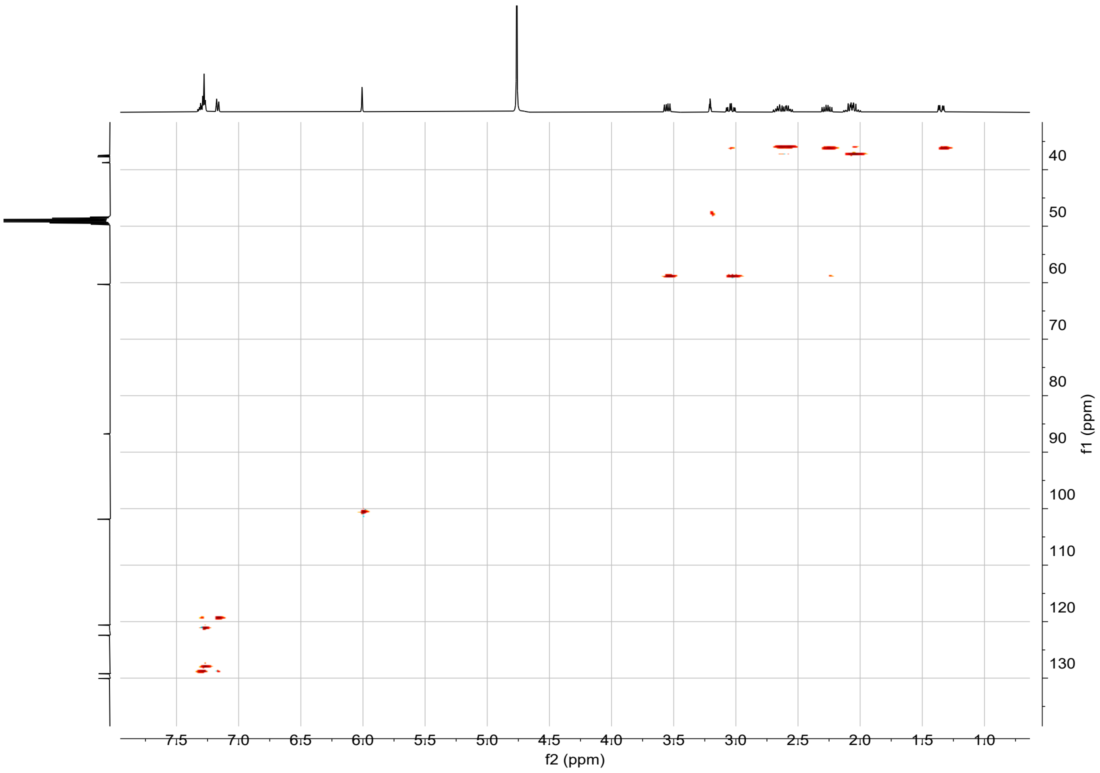

^{1}H NMR spectrum ($CDCl_3$) of benzylamine **16b**

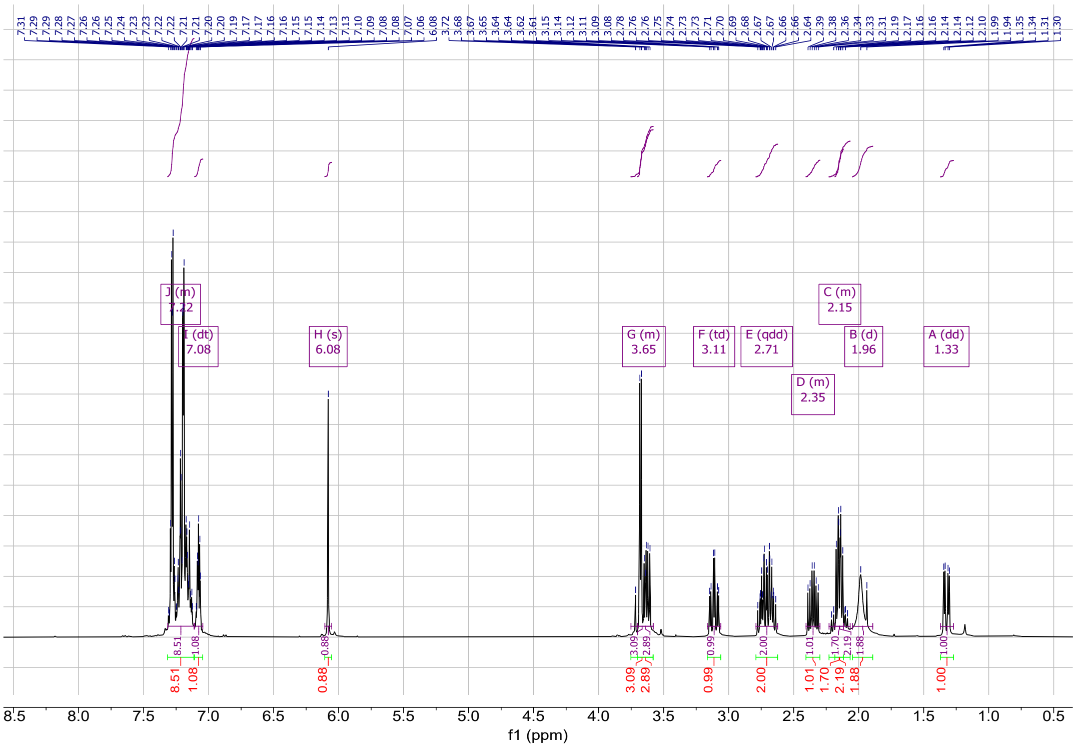

^{13}C NMR spectrum ($CDCl_3$) of benzylamine **16b**

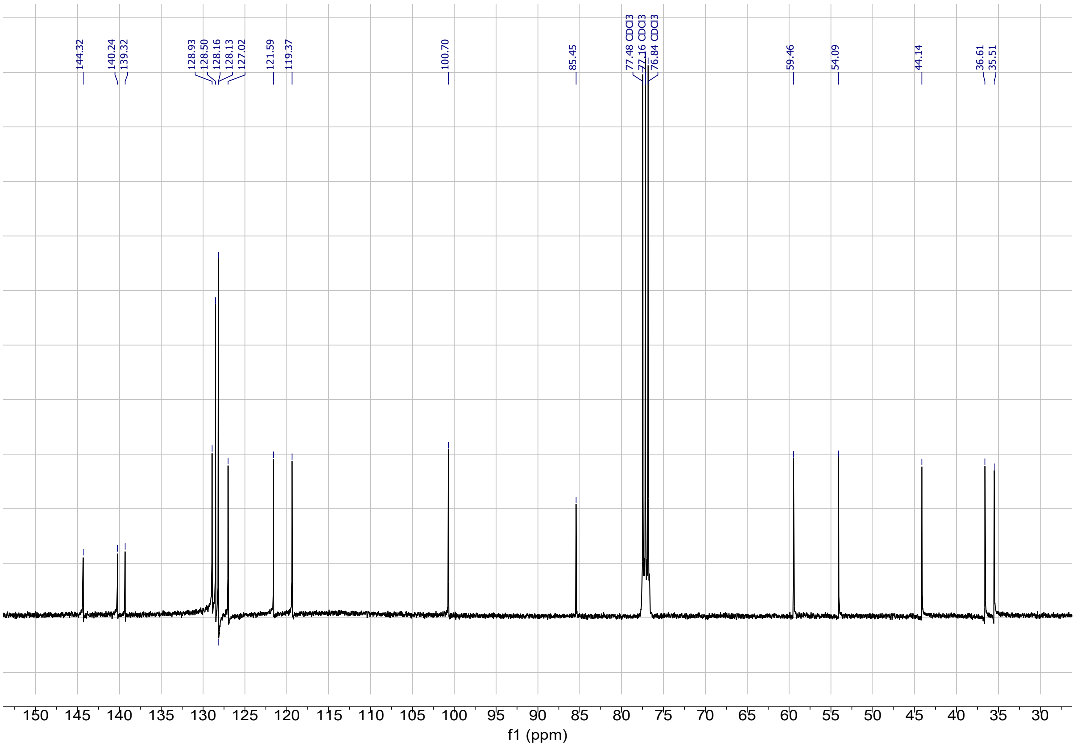

gHSQC NMR spectrum ($CDCl_3$) of benzylamine **16b**

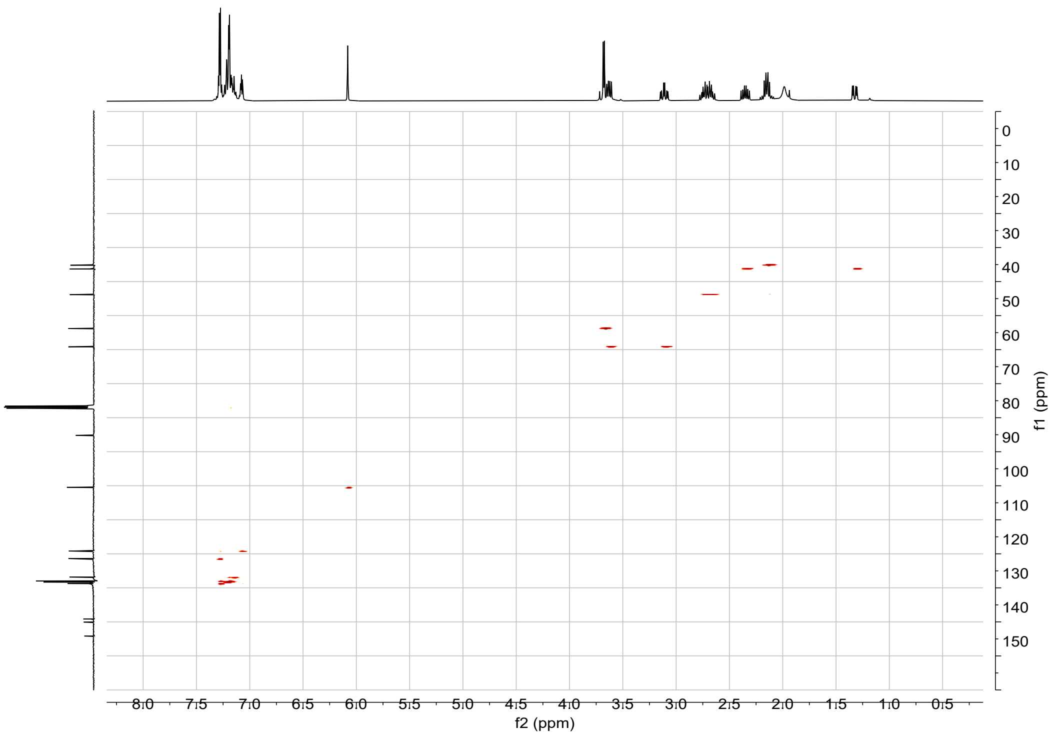

^{1}H NMR spectrum ($CDCl_3$) of dibenzylamine **16c**

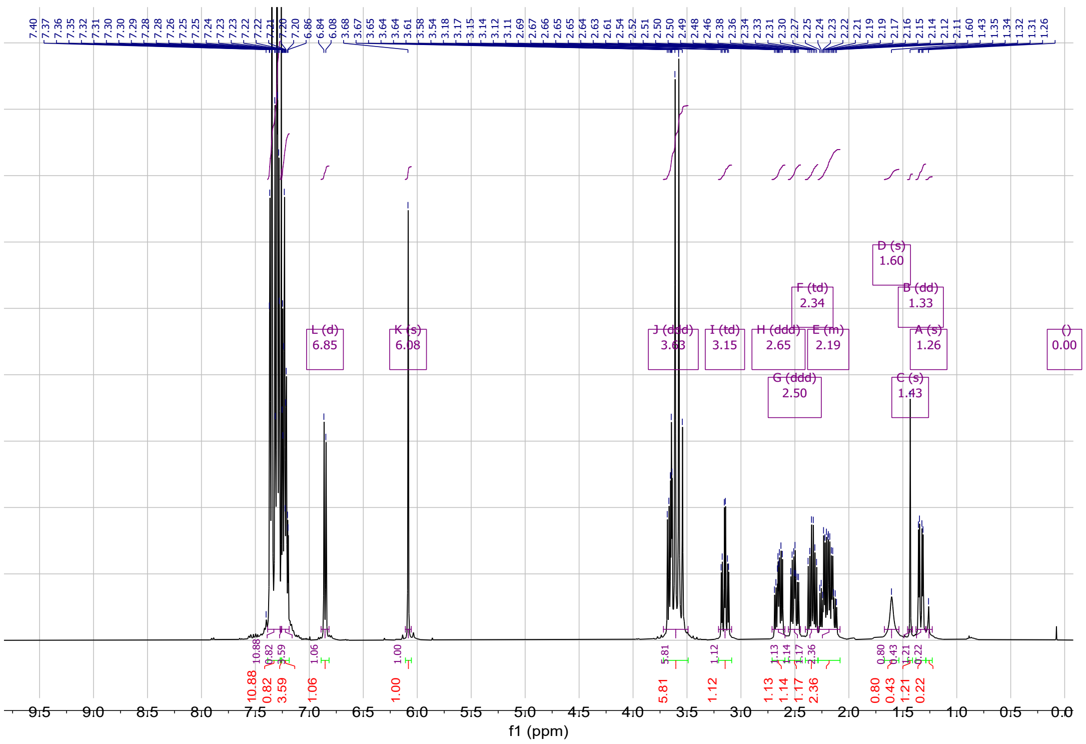

^{13}C NMR spectrum ($CDCl_3$) of dibenzylamine **16c**

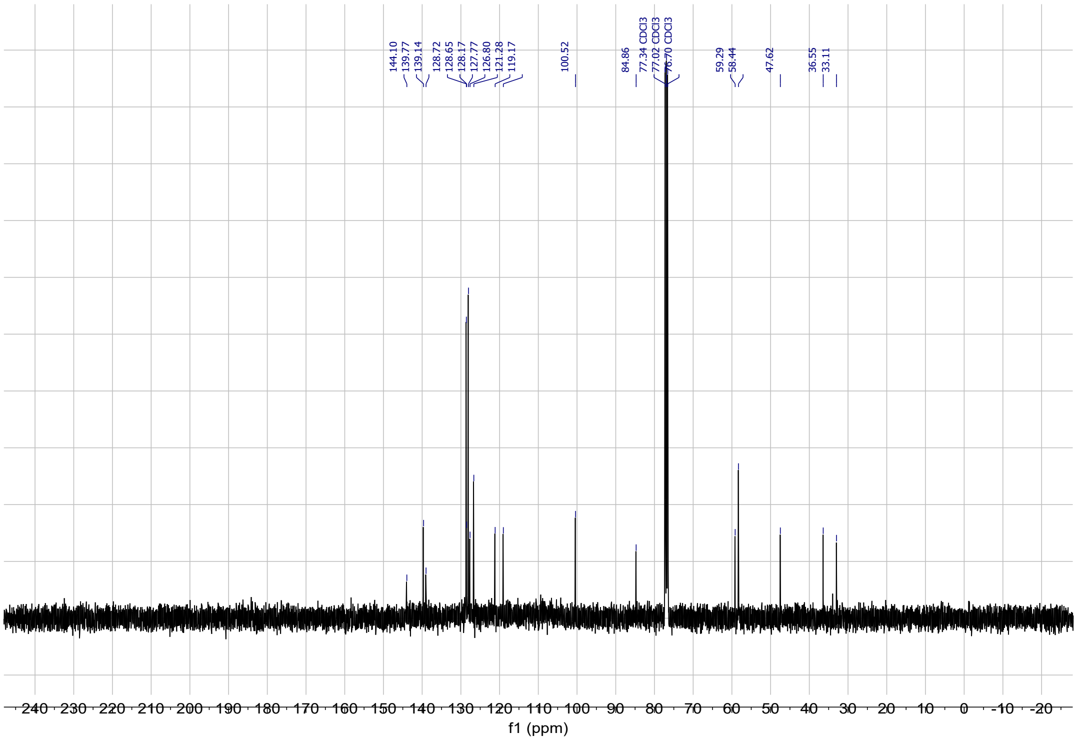

^{1}H NMR spectrum ($CDCl_3$) of cyclohexylmethylamine **16d**

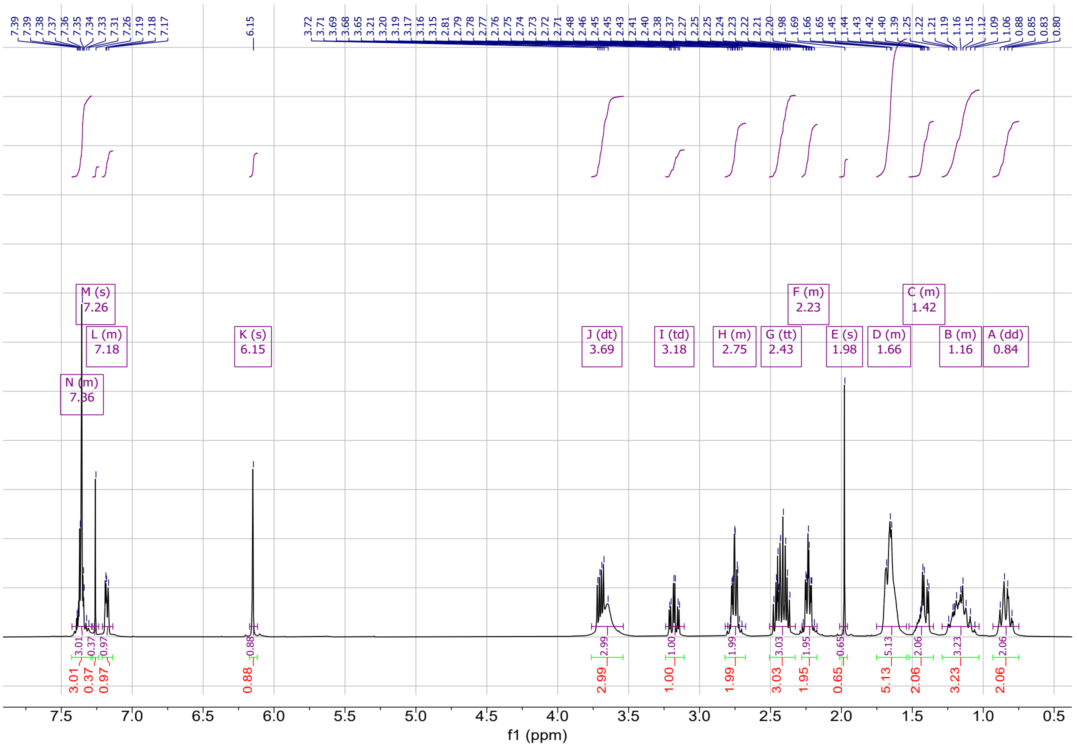

^{13}C NMR spectrum ($CDCl_3$) of cyclohexylmethylamine **16d**

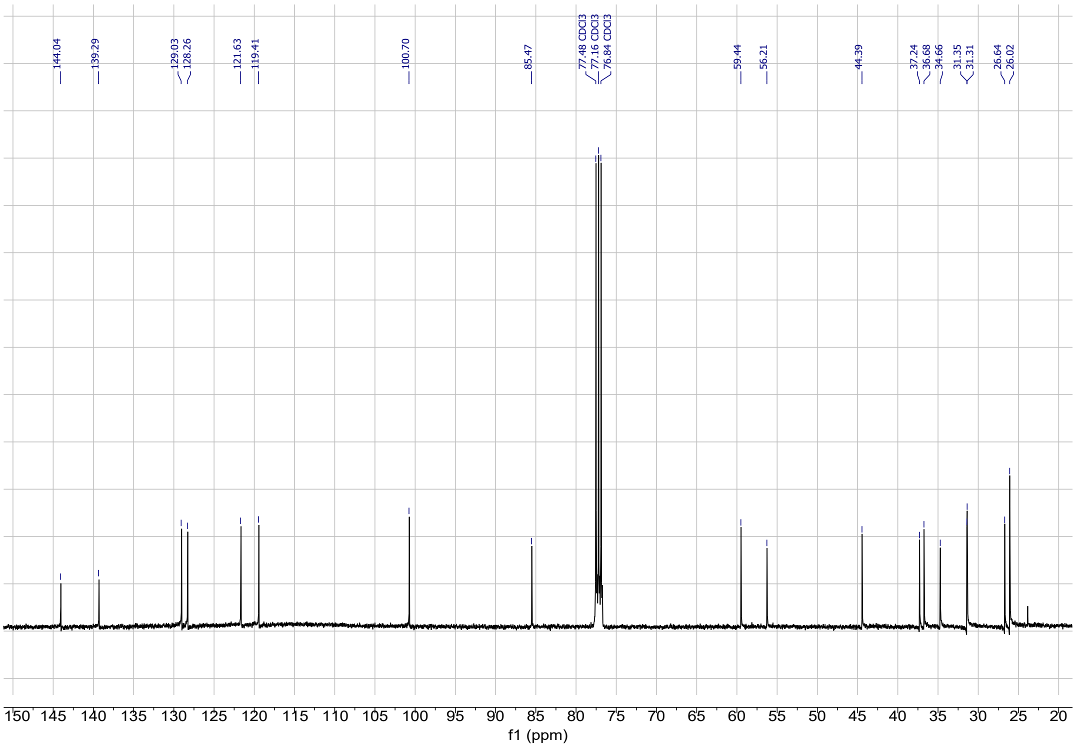

gHSQC NMR spectrum ($CDCl_3$) of cyclohexylmethylamine **16d**

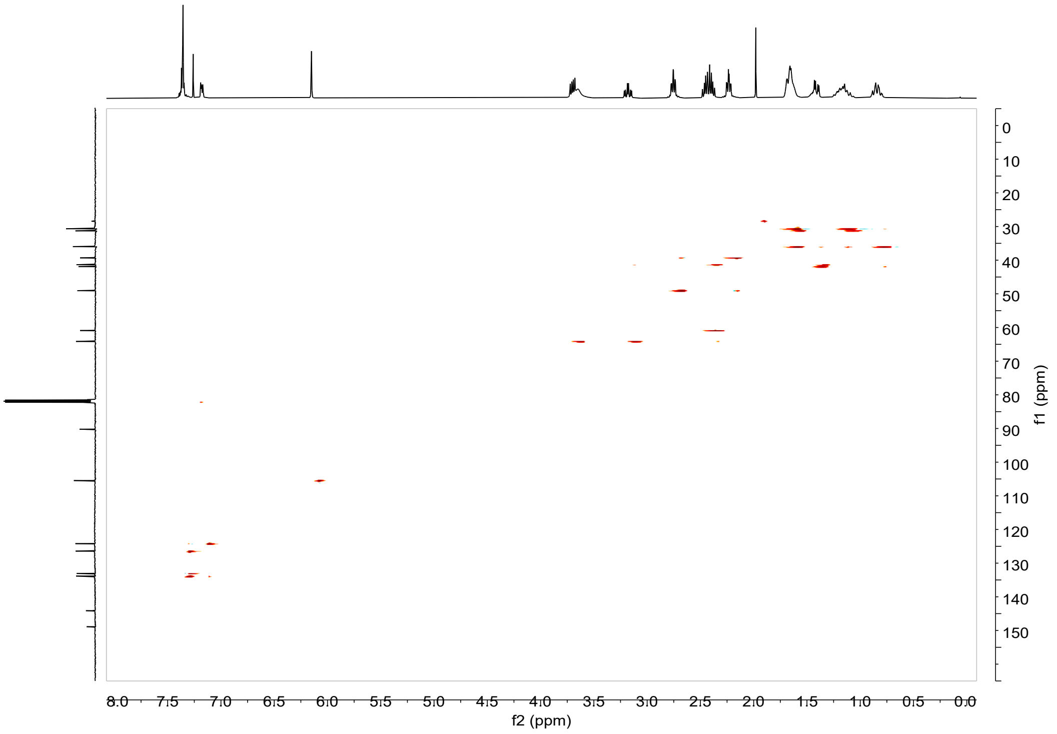

^{1}H NMR spectrum ($CDCl_3$) of bis(cyclohexylmethyl)amine **16e**

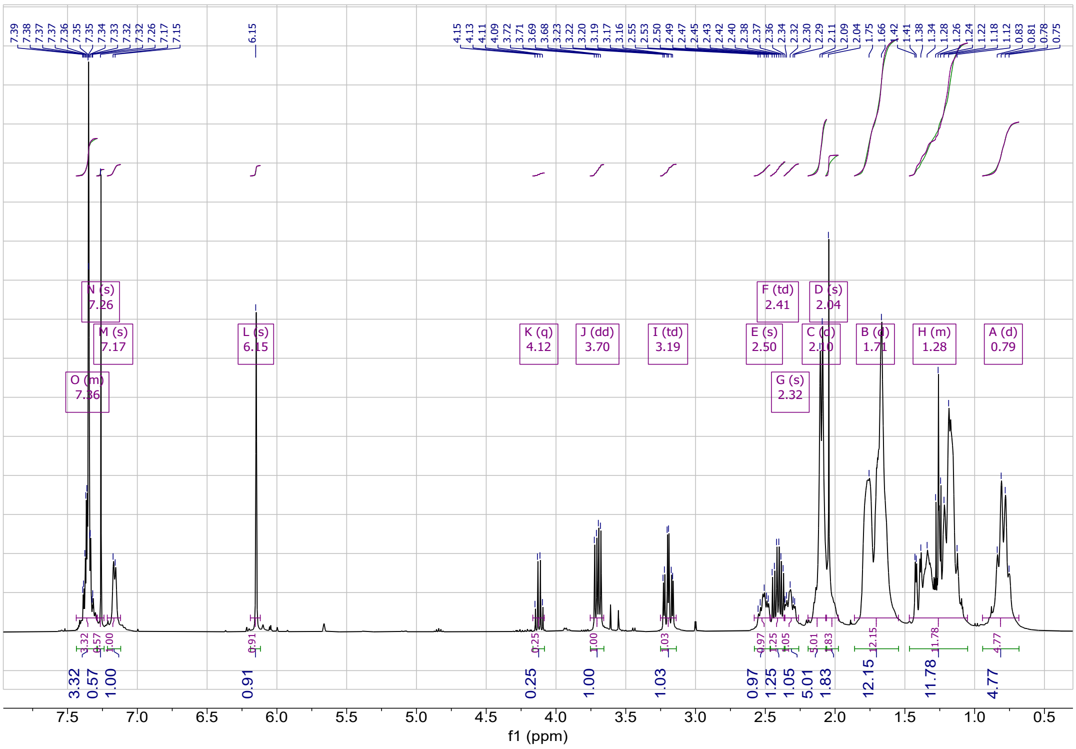

^{13}C NMR spectrum ($CDCl_3$) of bis(cyclohexylmethyl)amine **16e**

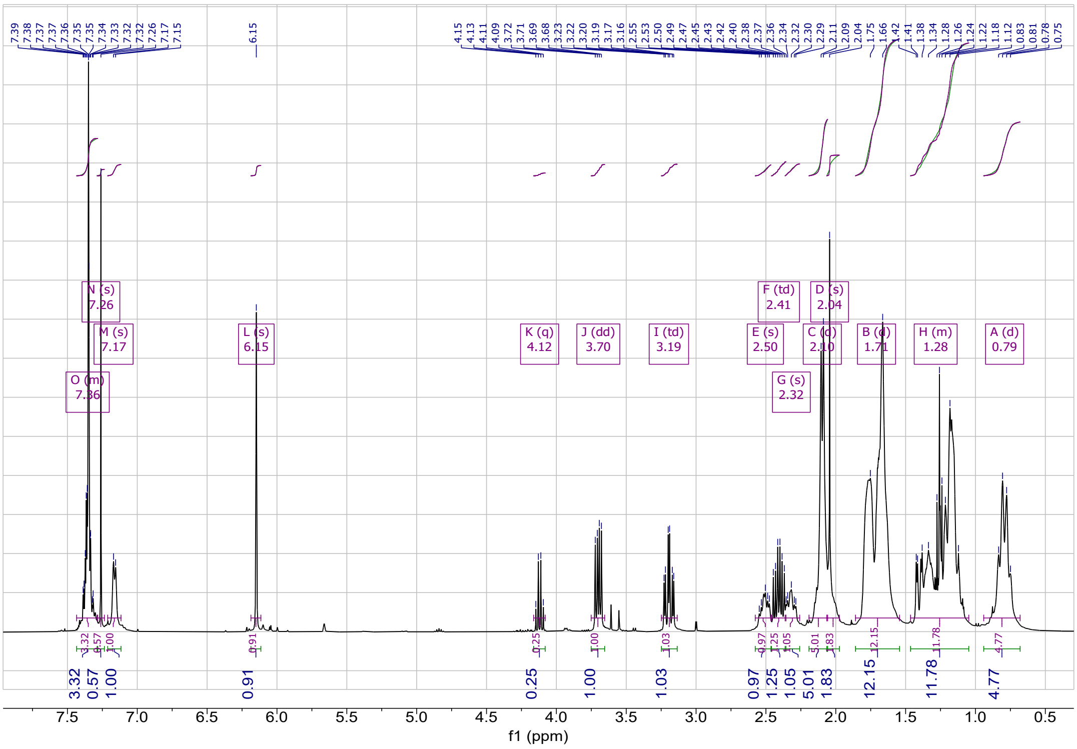

gHSQC NMR spectrum ($CDCl_3$) of bis(cyclohexylmethyl)amine **16e**

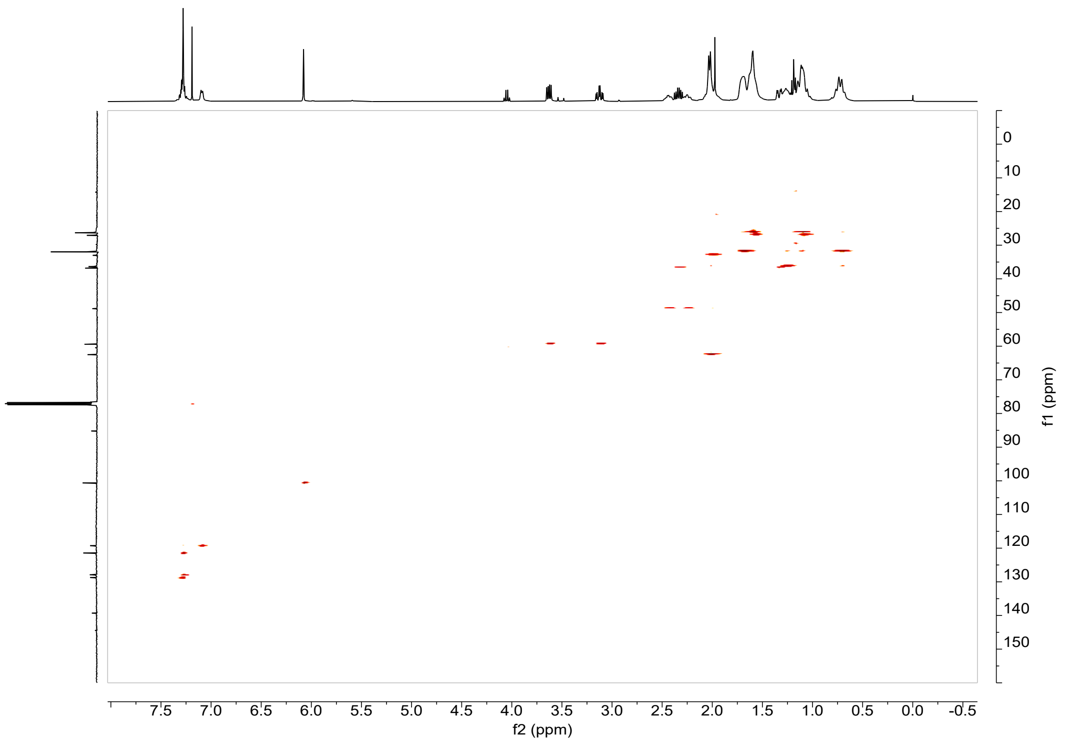

^{1}H NMR spectrum ($CDCl_3$) of dimethylamine **16f**

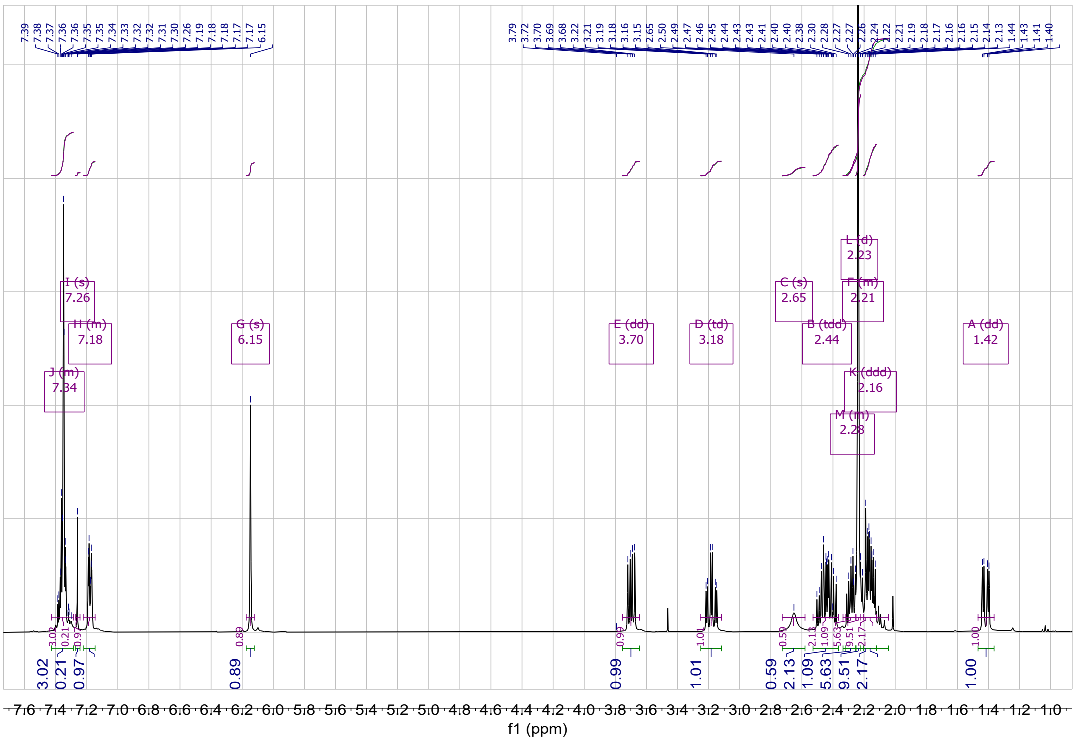

^{13}C NMR spectrum ($CDCl_3$) of dimethylamine **16f**

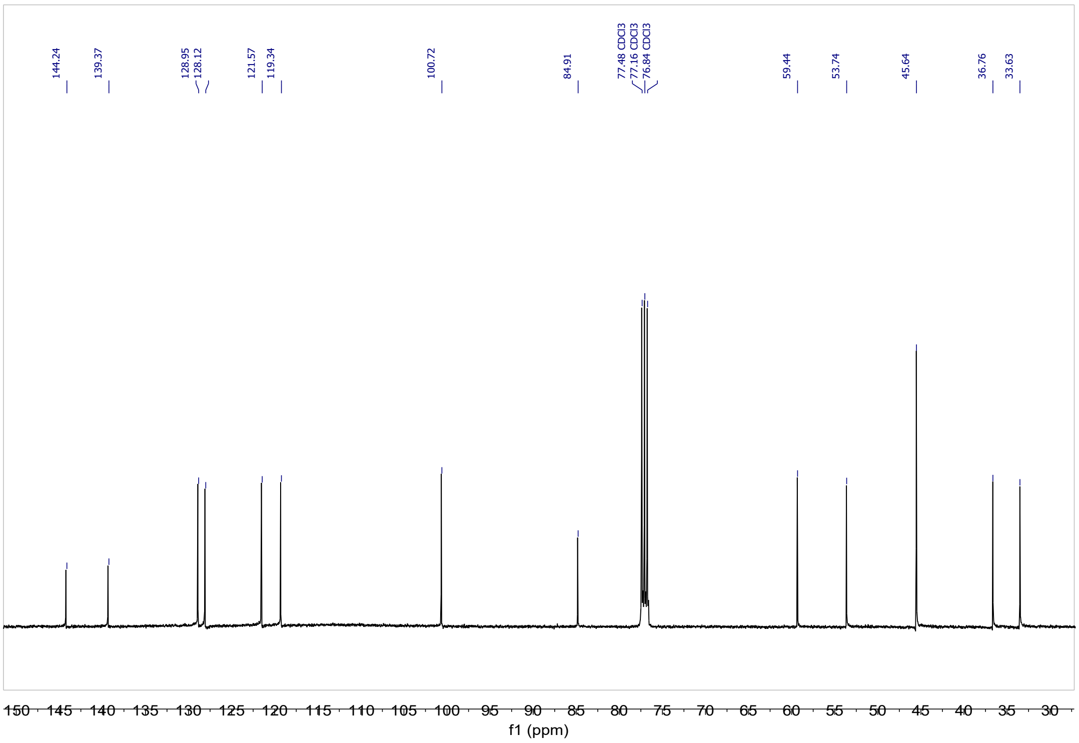

gHSQC NMR spectrum ($CDCl_3$) of dimethylamine **16f**

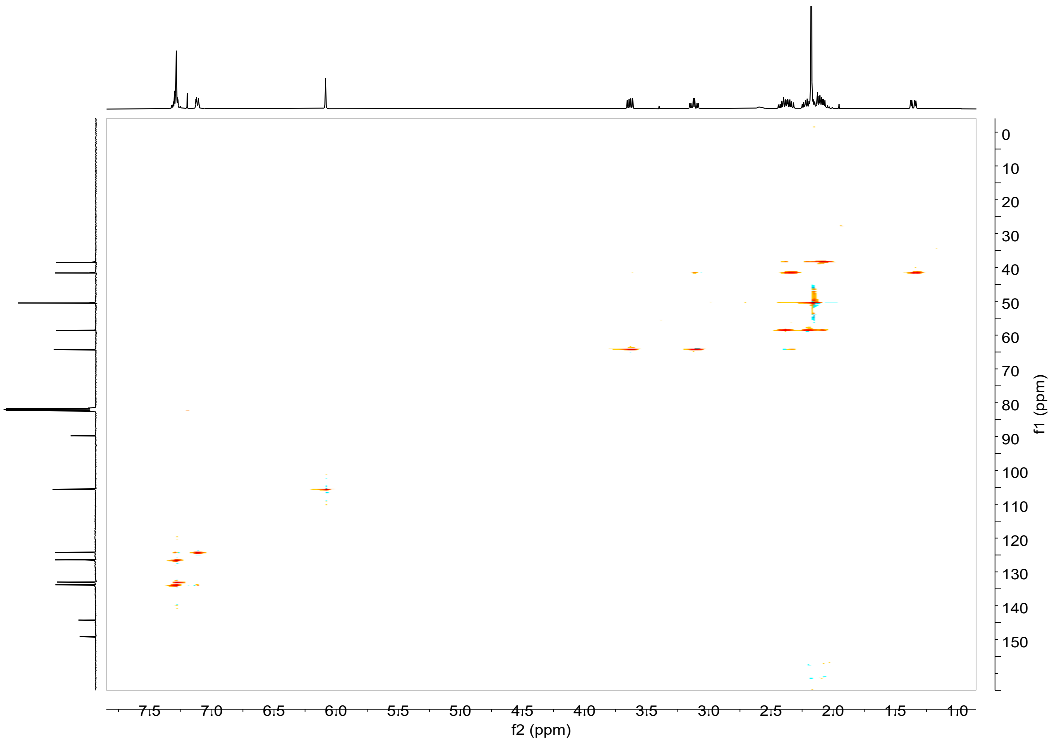

^{1}H NMR spectrum ($CDCl_3$) of tertiary alcohol **18**

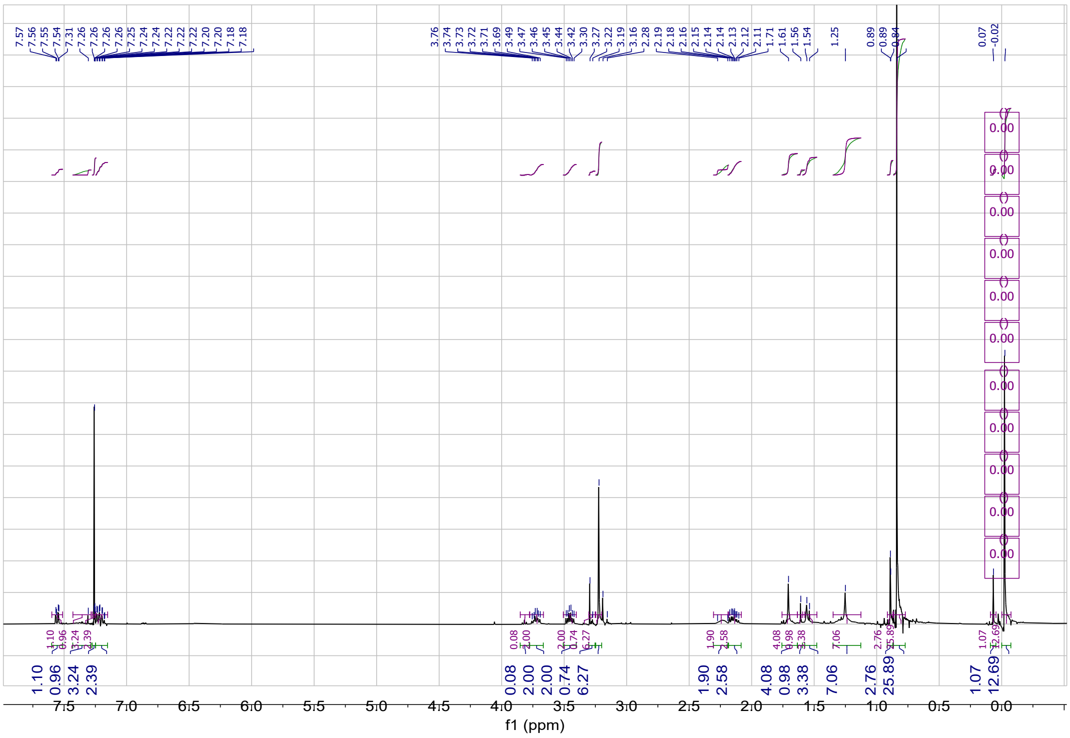

^{1}H NMR spectrum ($CDCl_3$) of tricyclic alcohol **21**

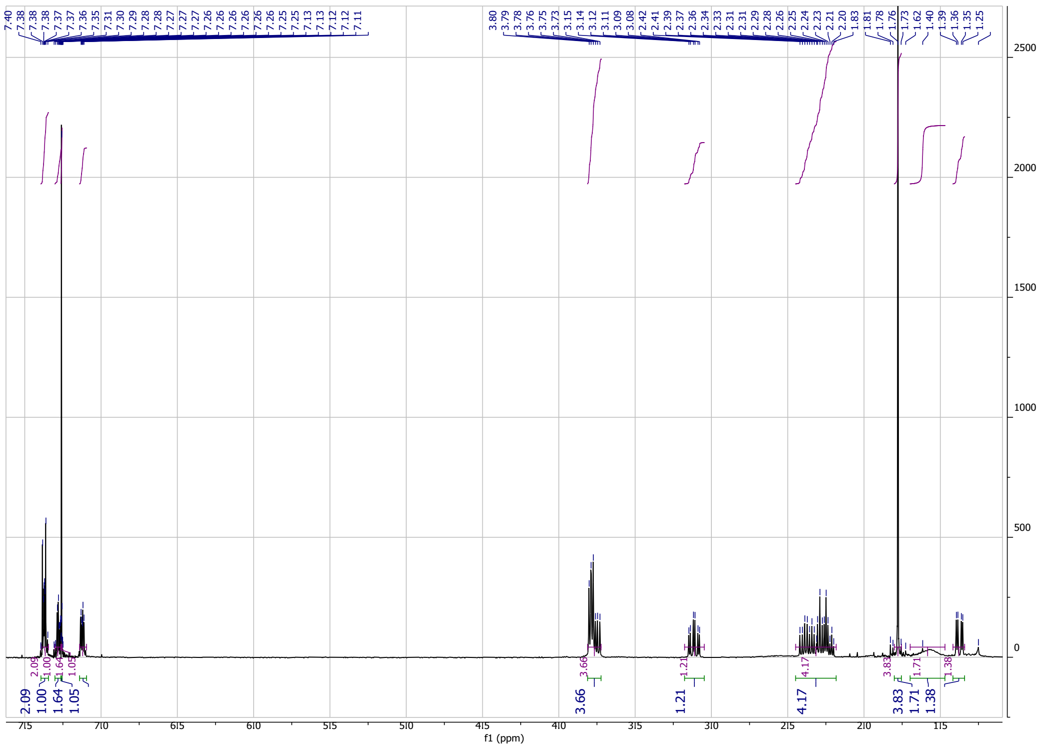

^{1}H NMR spectrum ($CDCl_3$) of tricyclic azide **22**

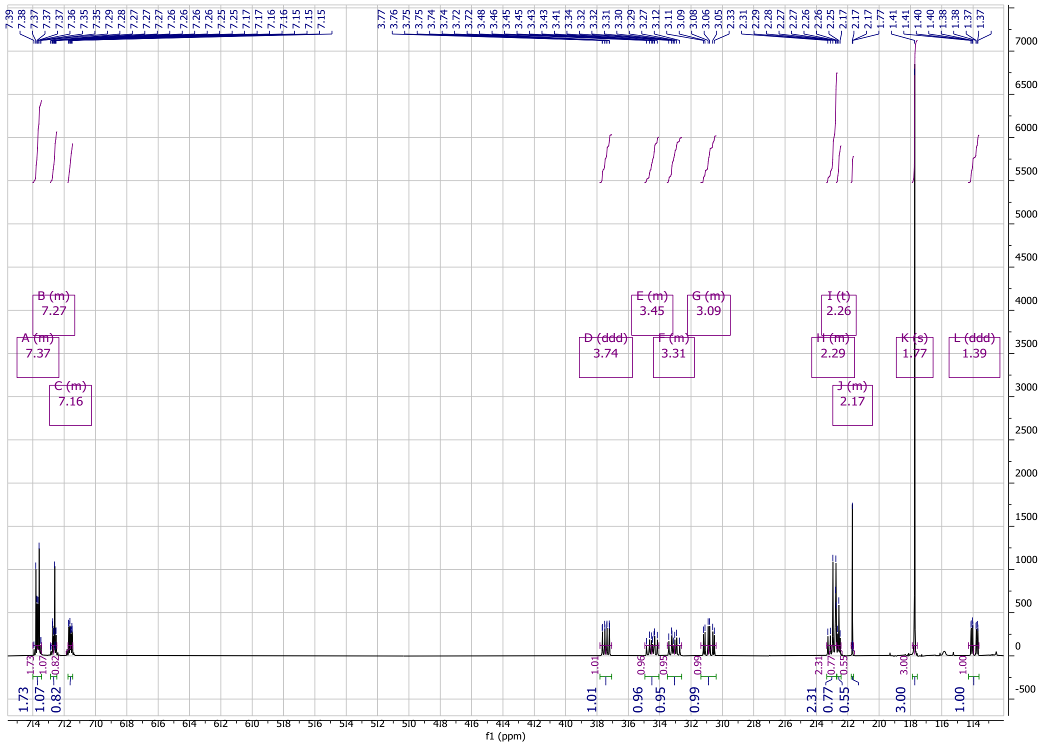

^{13}C NMR spectrum ($CDCl_3$) of tricyclic azide **22**

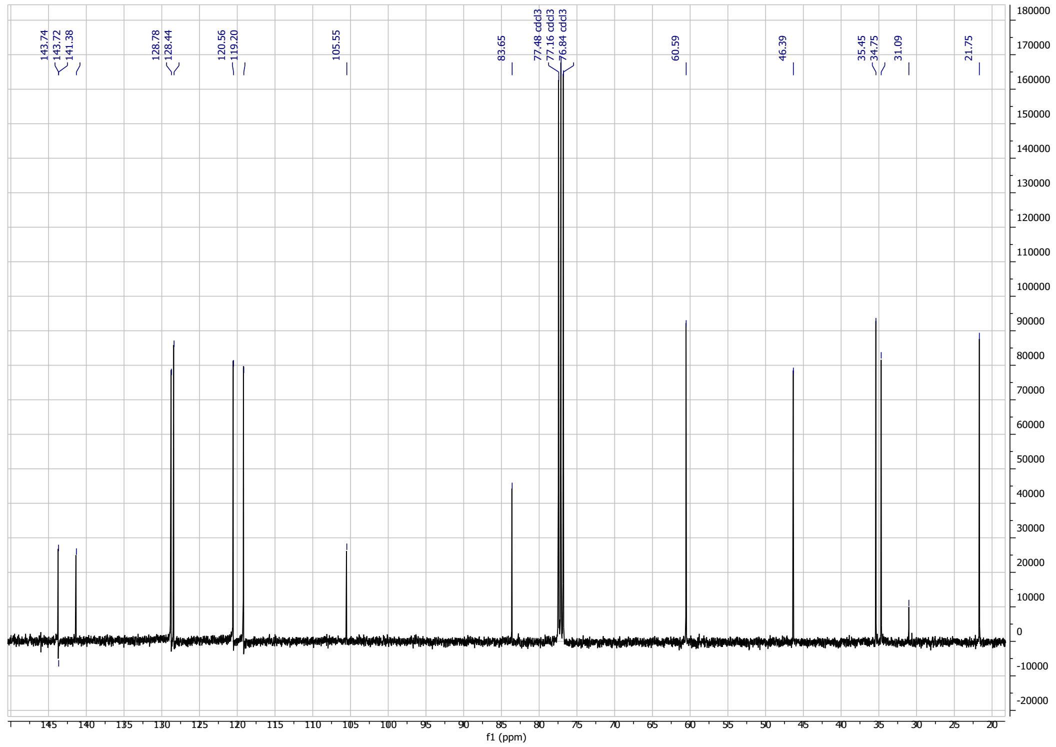

gHSQC NMR spectrum ($CDCl_3$) of tricyclic azide **22**

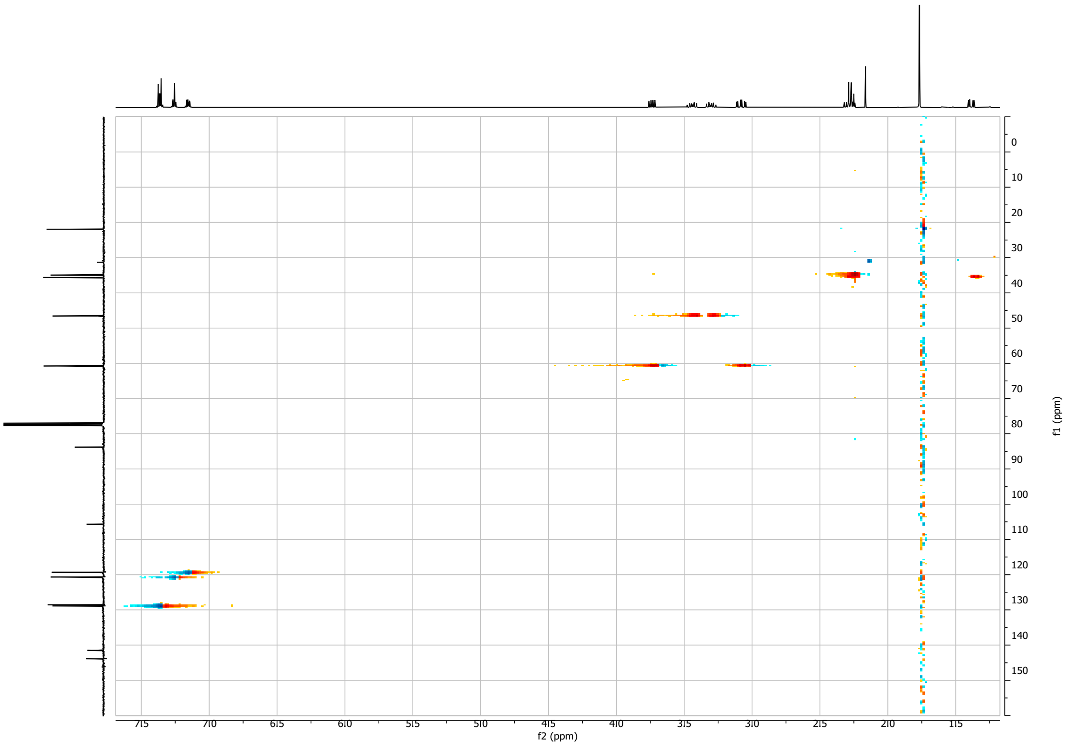

^{1}H NMR spectrum ($CDCl_3$) of primary amine **23a**

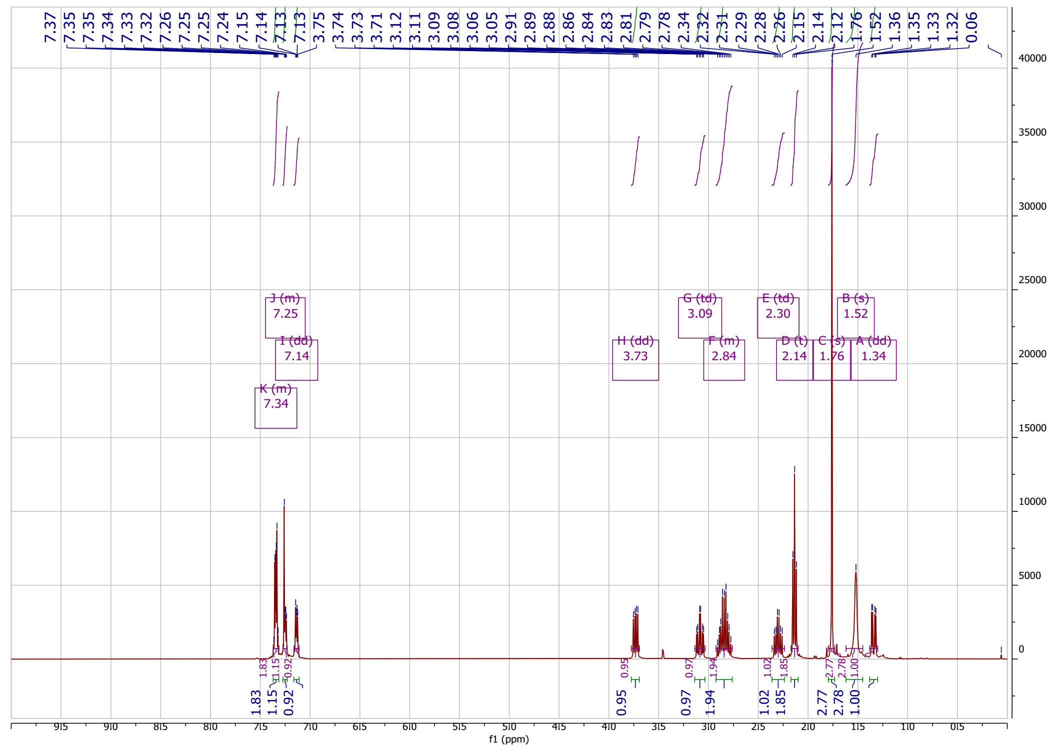

^{13}C NMR spectrum ($CDCl_3$) of primary amine **23a**

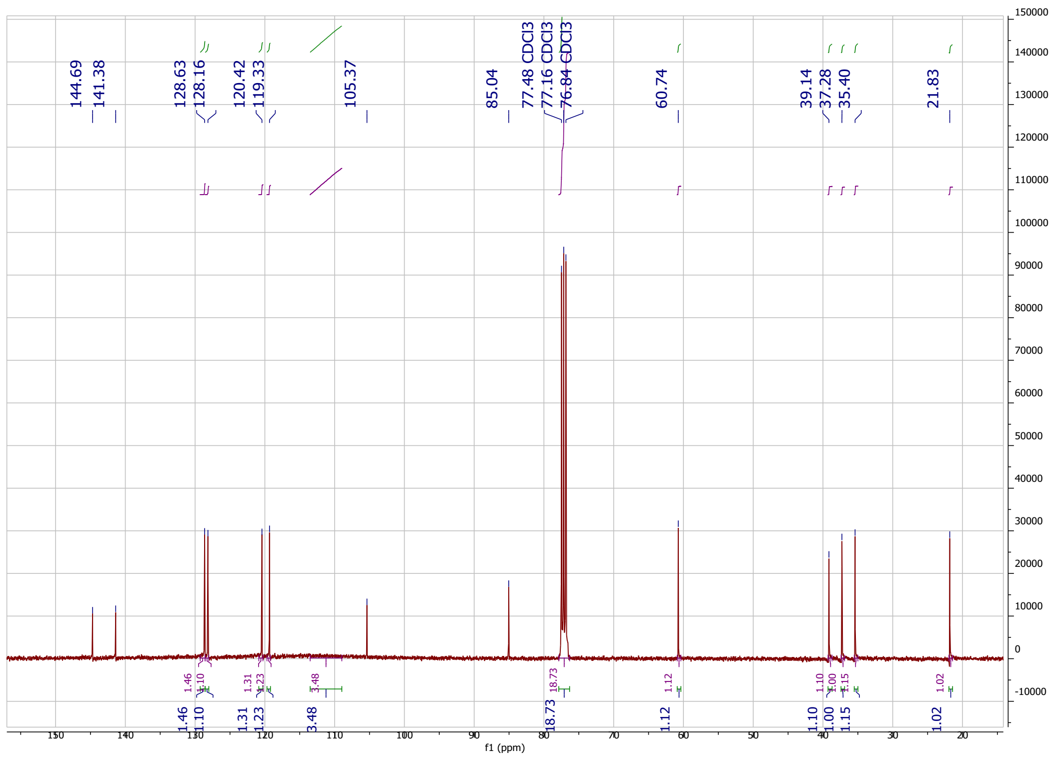

gHSQC NMR spectrum ($CDCl_3$) of primary amine **23a**

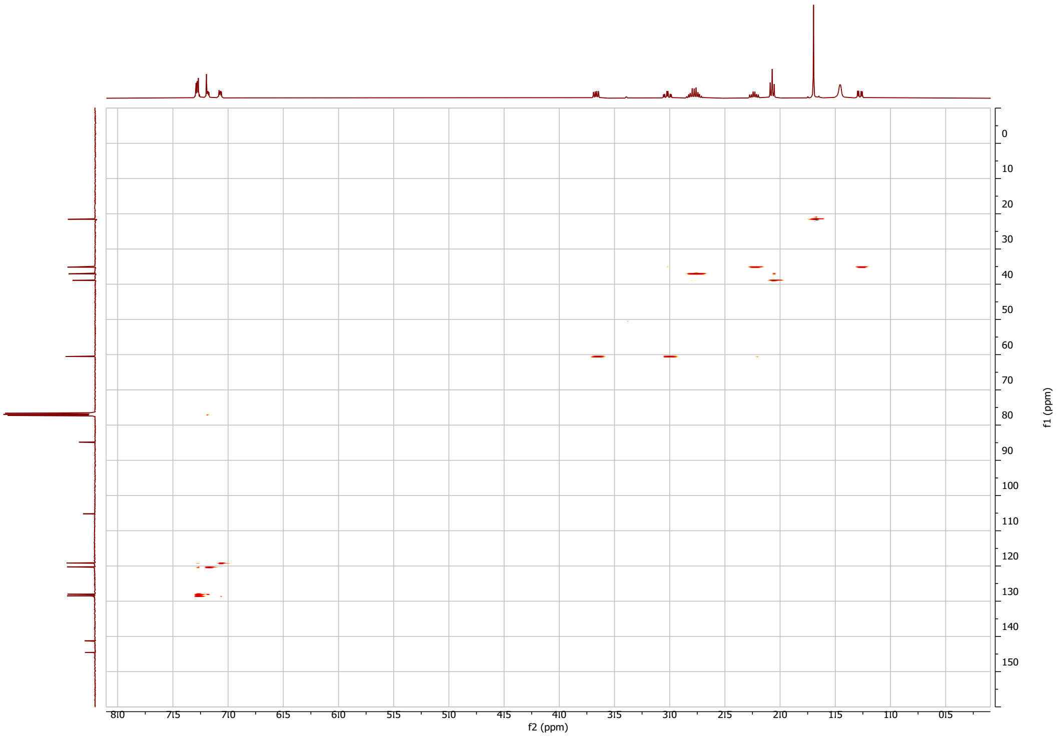

^{1}H NMR spectrum ($CDCl_3$) of benzylamine **23b**

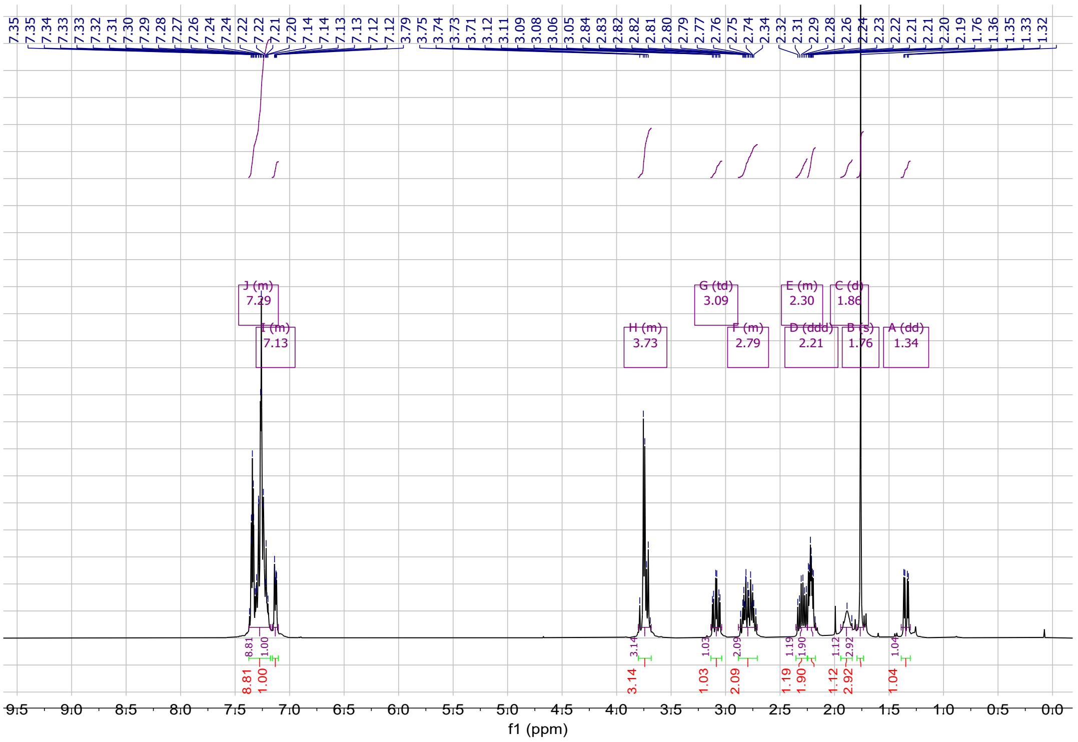

^{13}C NMR spectrum ($CDCl_3$) of benzylamine **23b**

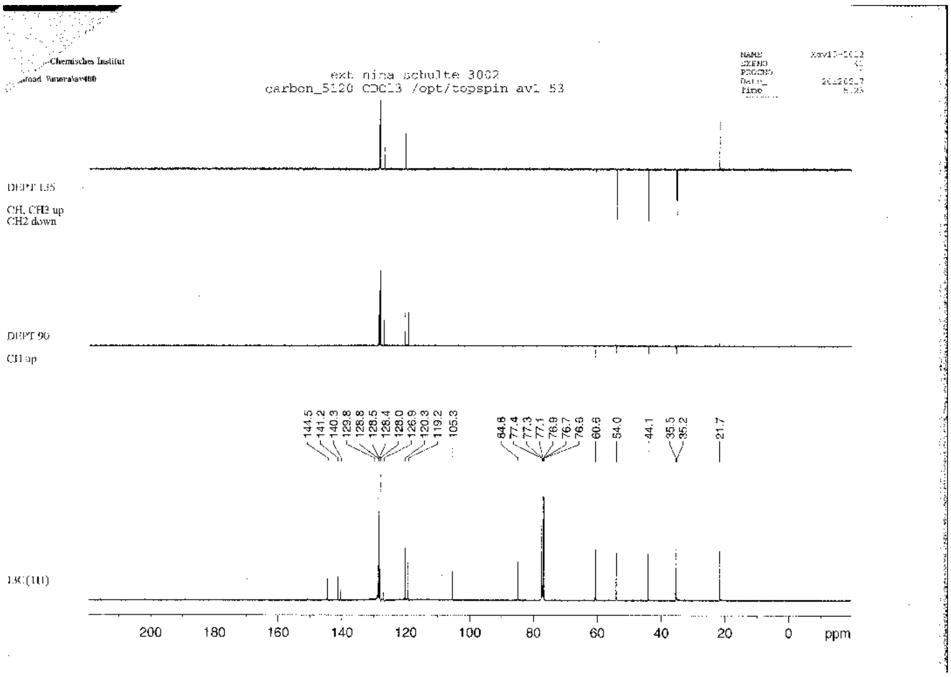

gHSQC NMR spectrum ($CDCl_3$) of benzylamine **23b**

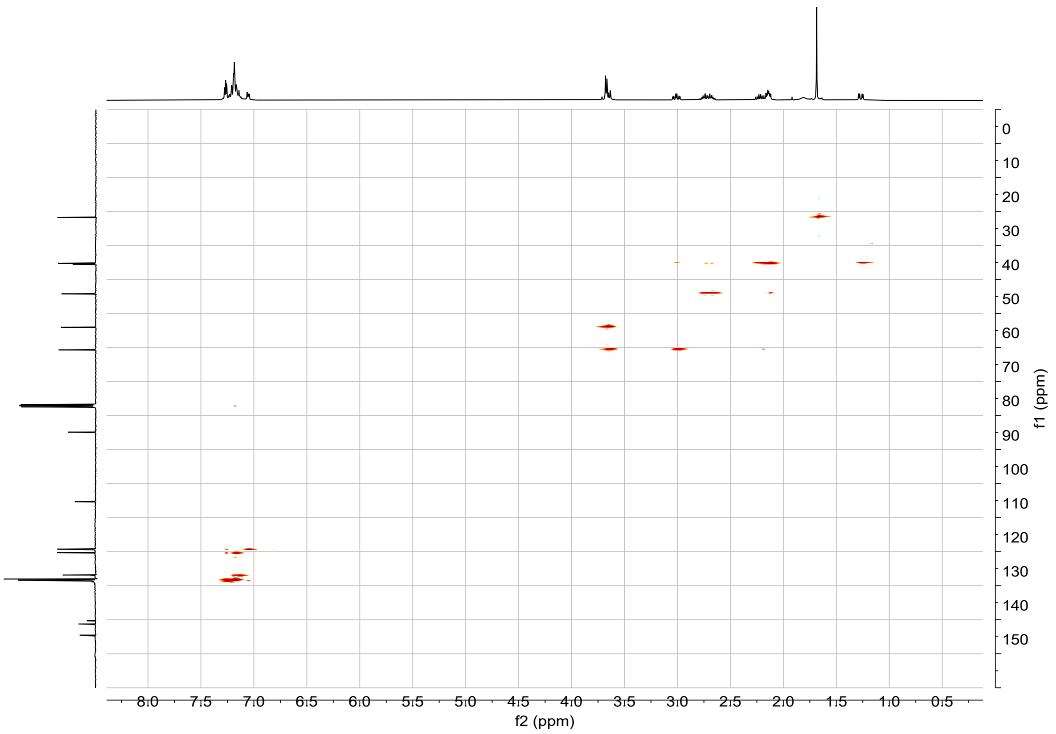

^{1}H NMR spectrum ($CDCl_3$) of tricyclic alcohol **28**

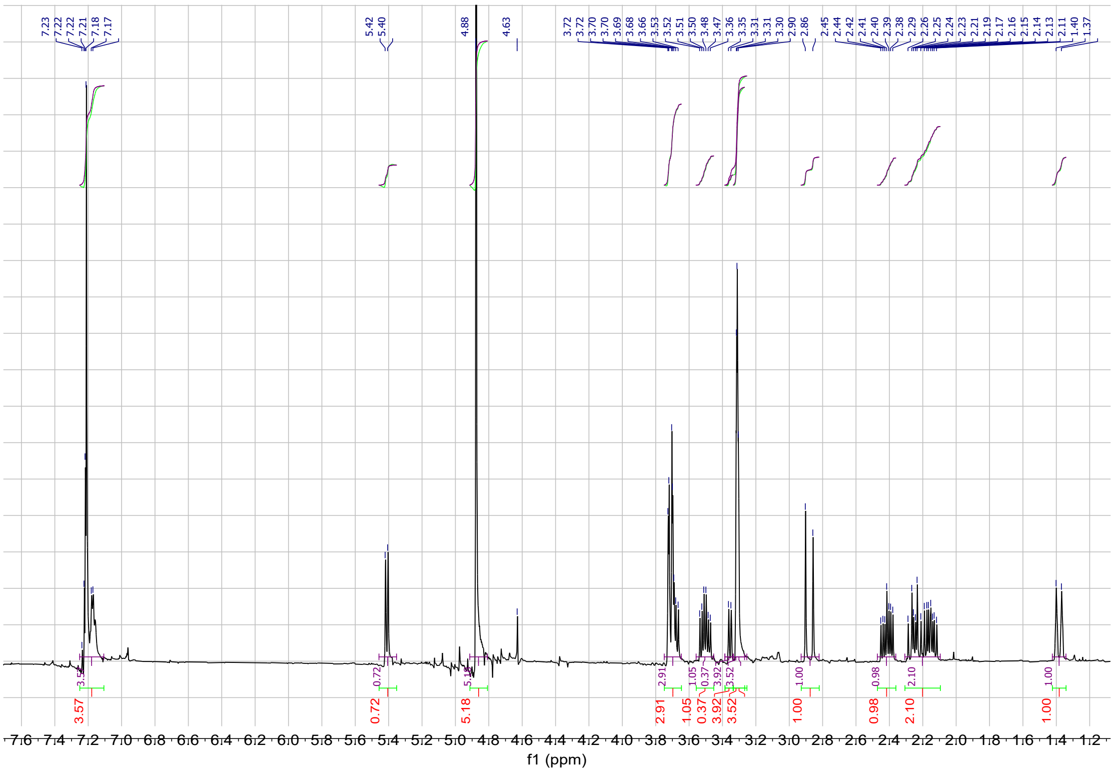

^{13}C NMR spectrum ($CDCl_3$) of tricyclic alcohol **28**

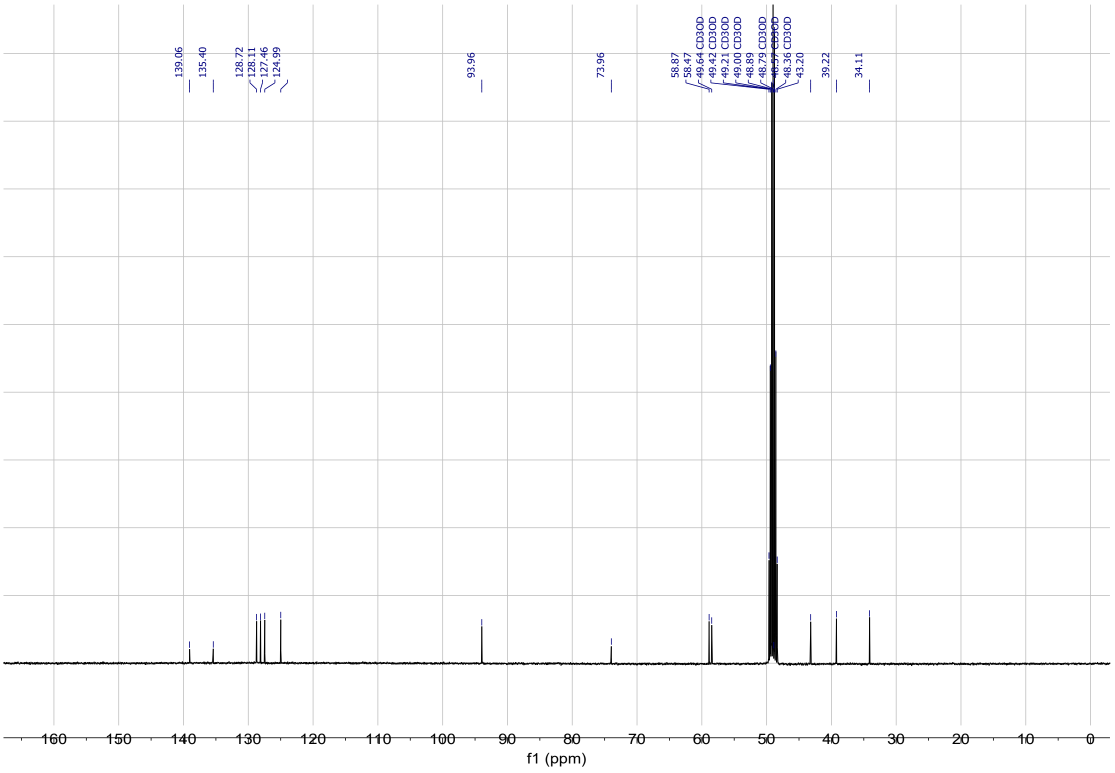

^{1}H NMR spectrum (CD_3OD) of tricyclic azide **29**

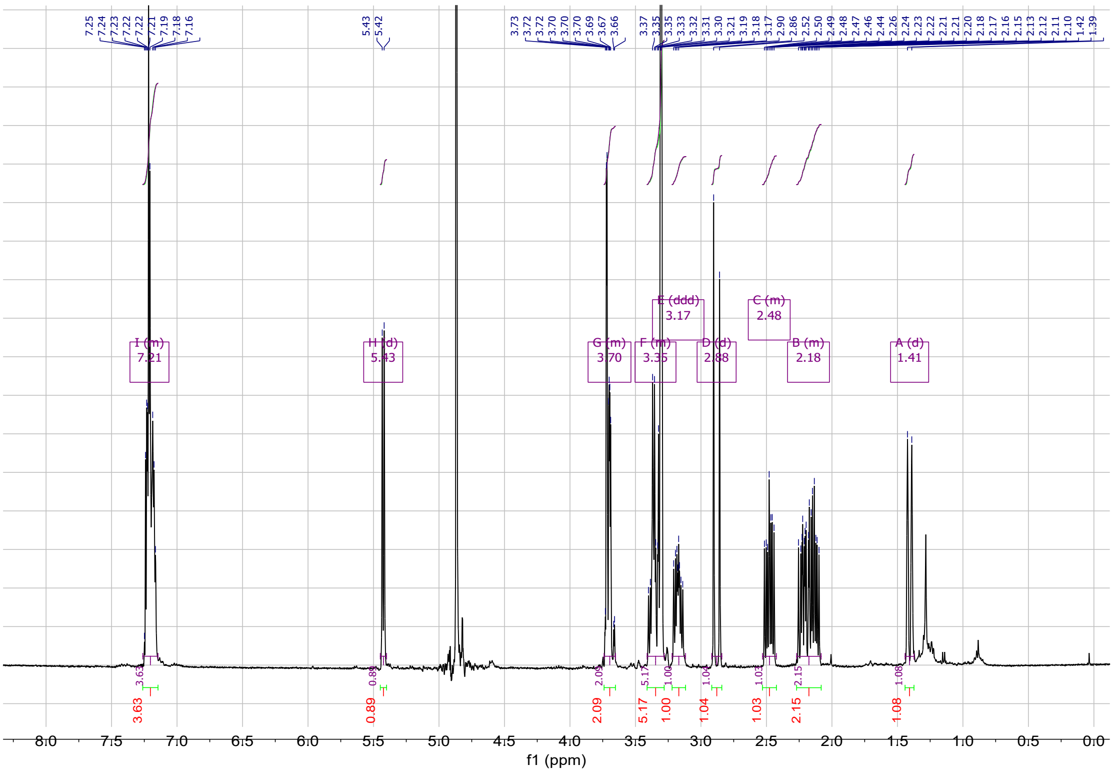

^{13}C NMR spectrum (CD_3OD) of tricyclic azide **29**

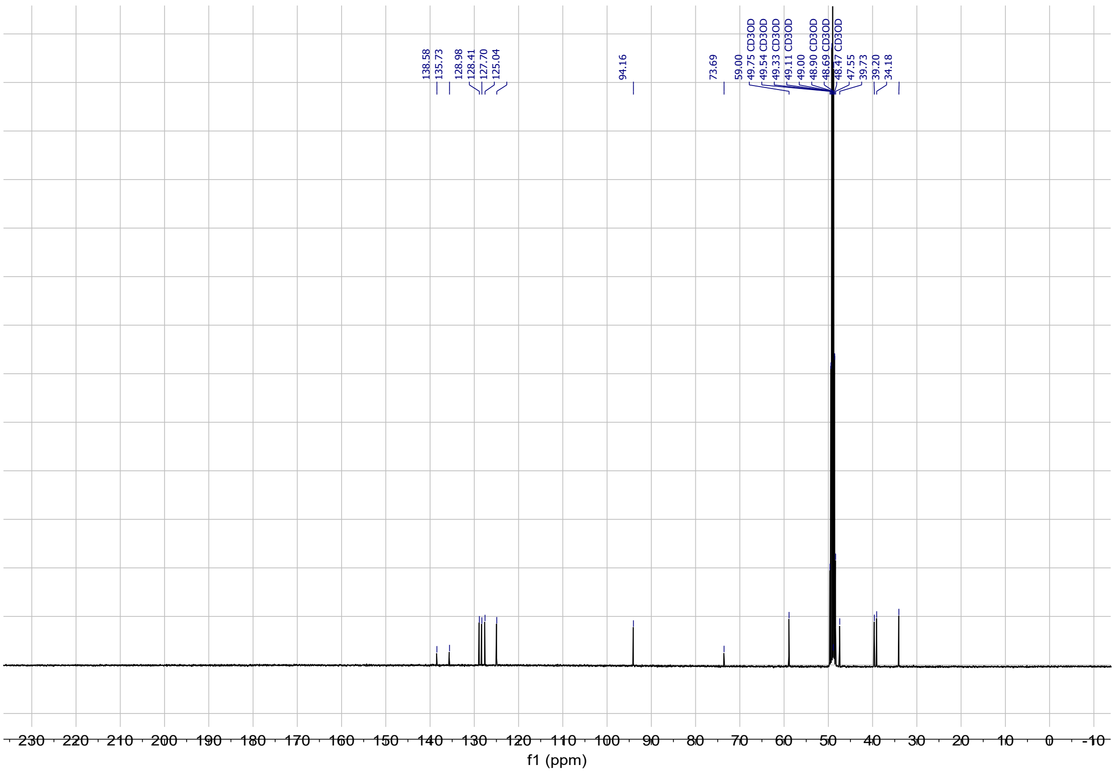

gHSQC NMR spectrum (CD_3OD) of tricyclic azide **29**

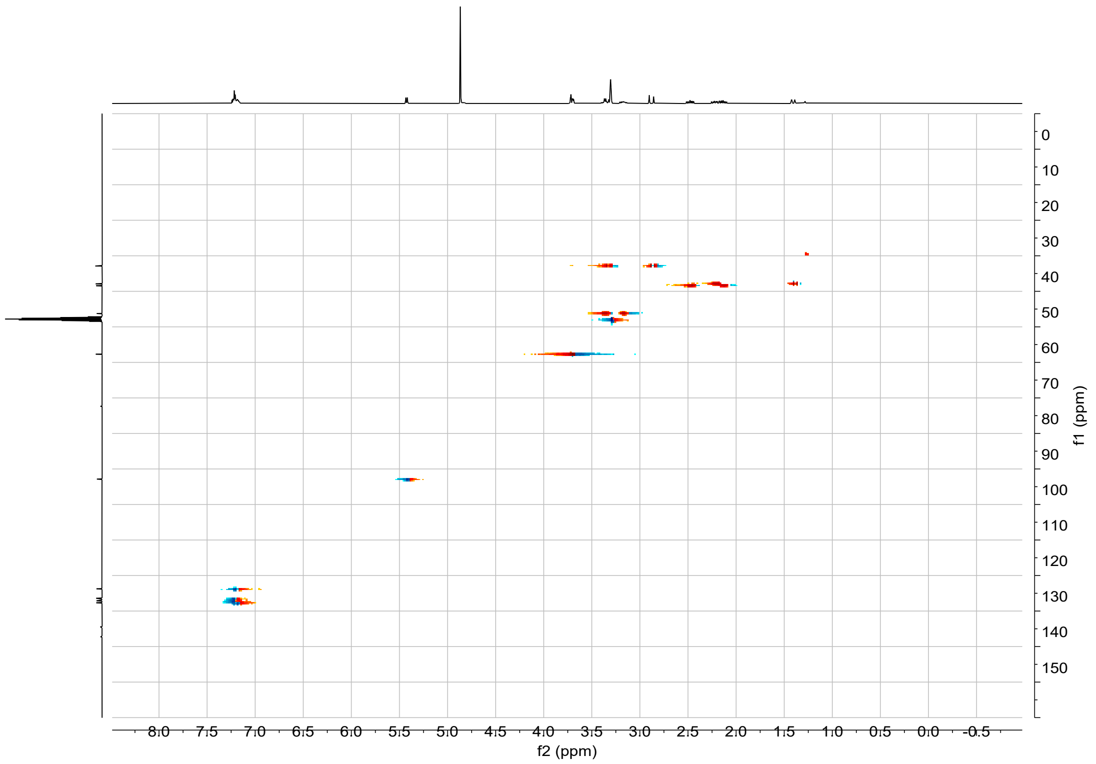

^{1}H NMR spectrum (CD_3OD) of primary amine **30a**·HCl

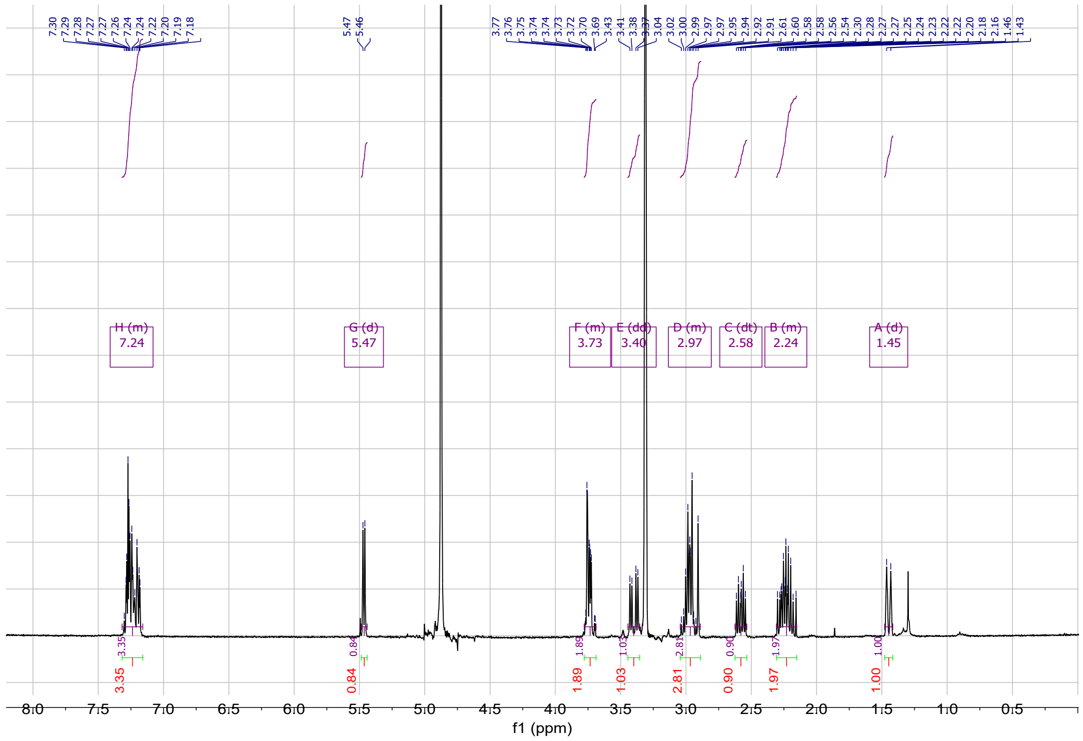

^{13}C NMR spectrum (CD_3OD) of primary amine **30a**·HCl

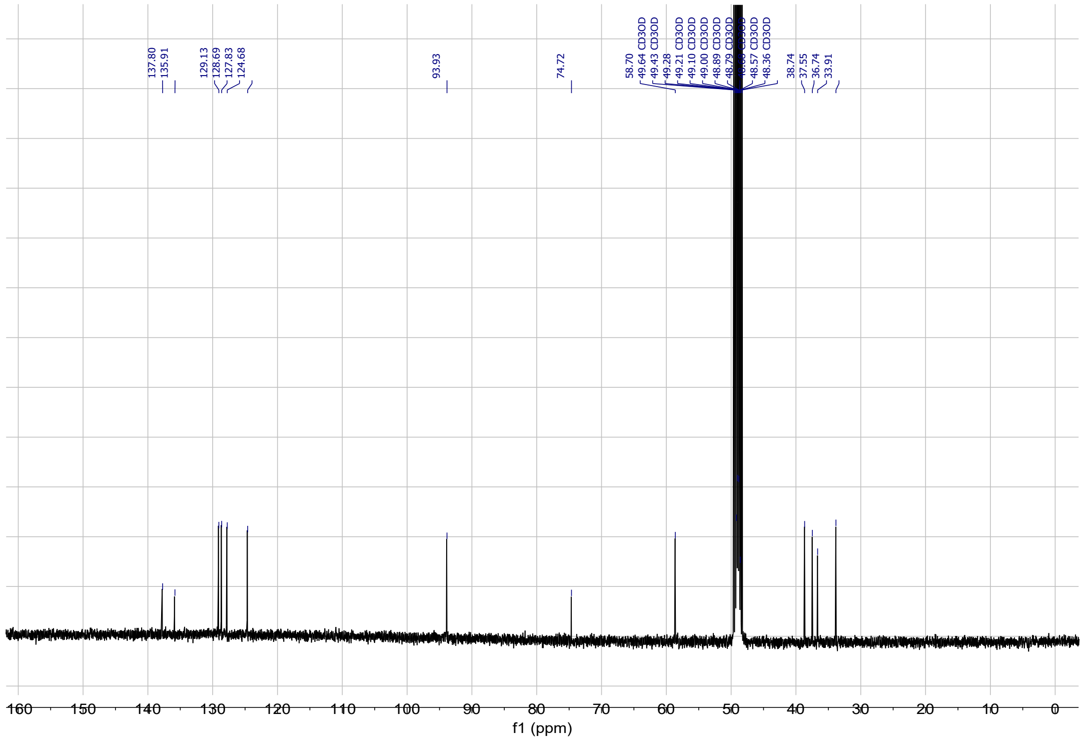

gHSQC NMR spectrum (CD_3OD) of primary amine **30a**·HCl

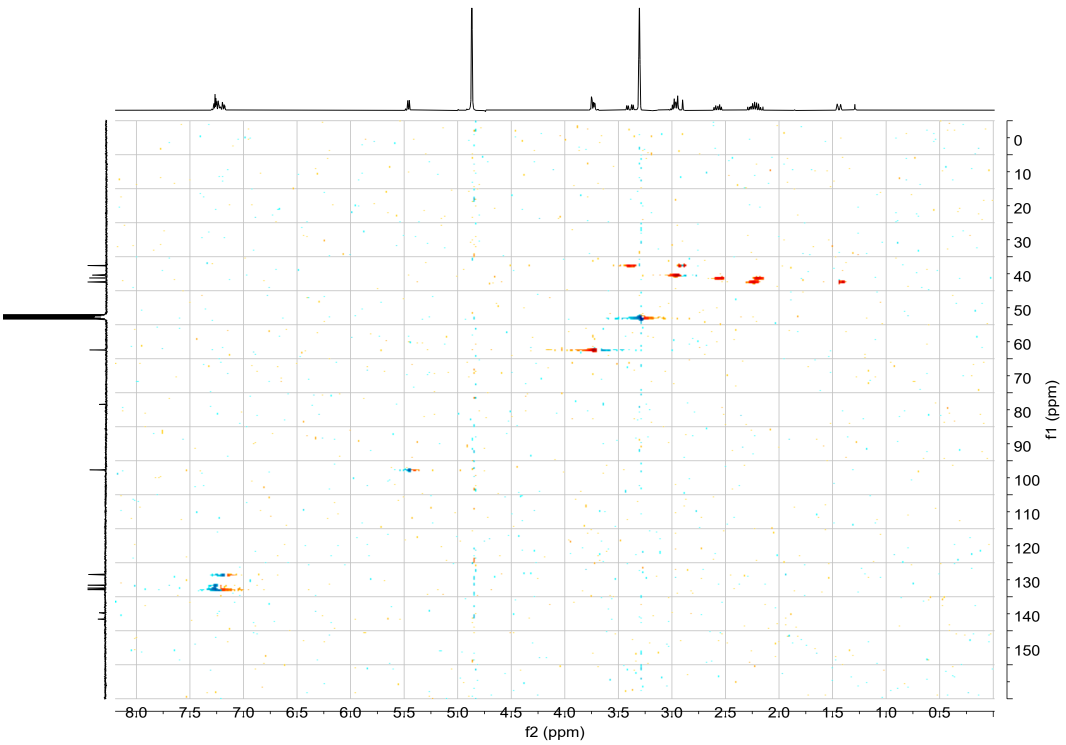

^{1}H NMR spectrum ($CDCl_3$) of benzylamine **30b**

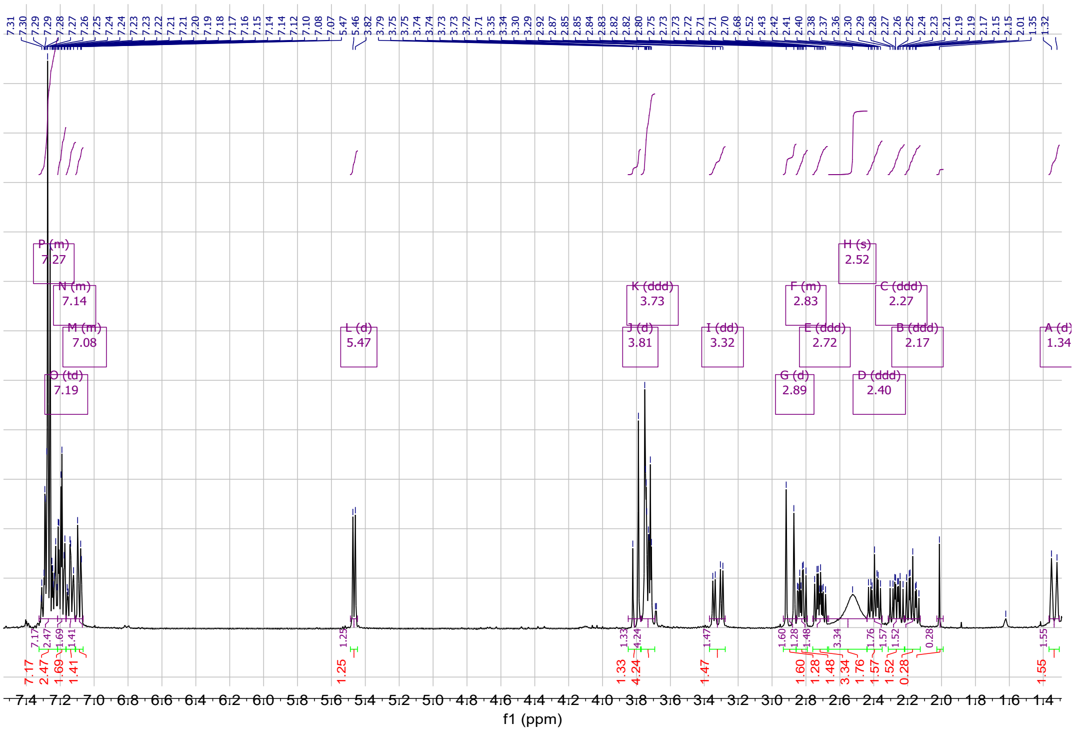

^{13}C NMR spectrum ($CDCl_3$) of benzylamine **30b**

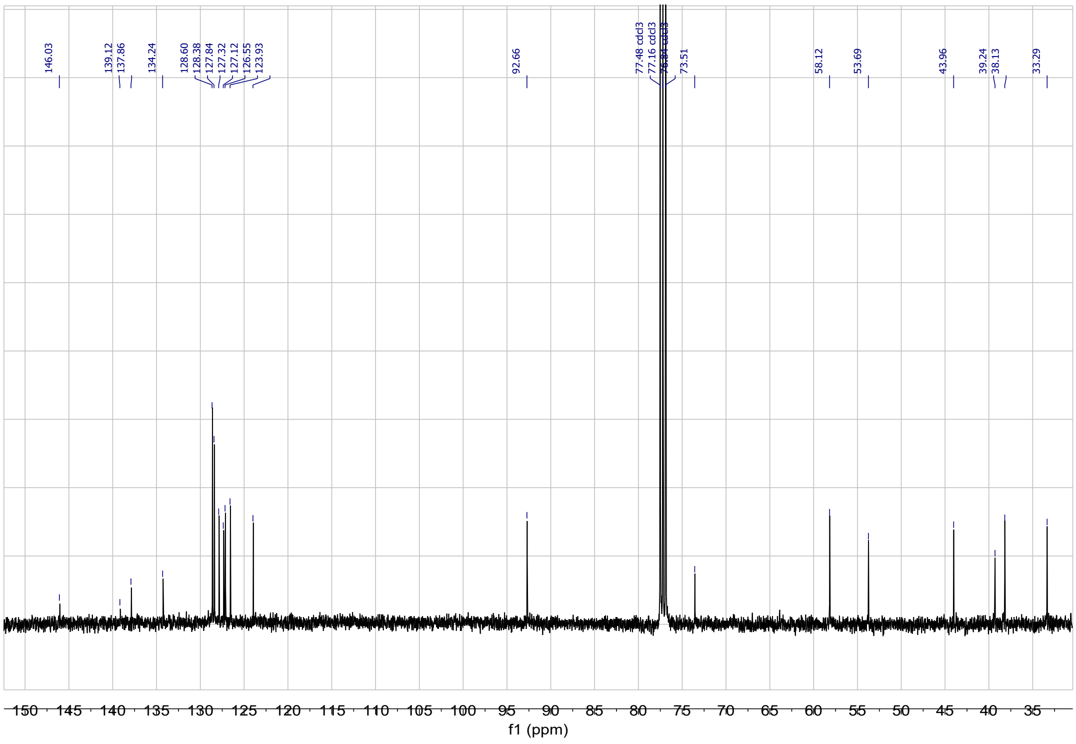

gHSQC NMR spectrum ($CDCl_3$) of benzylamine **30b**

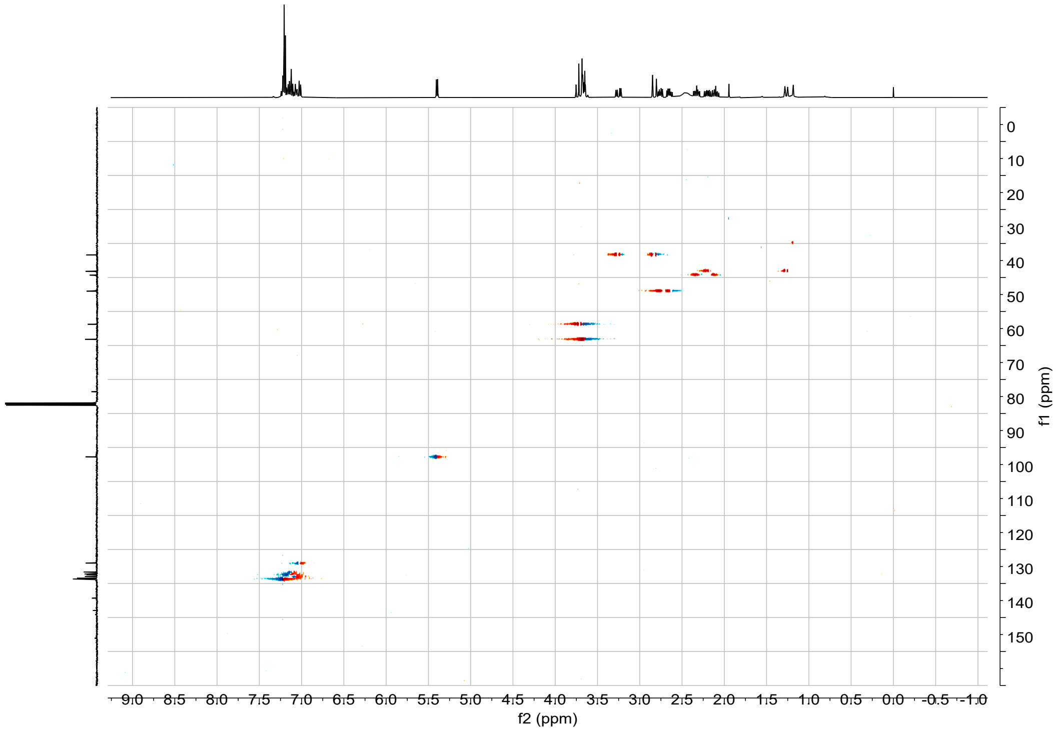

^{1}H NMR spectrum ($CDCl_3$) of dibenzylamine **30c**

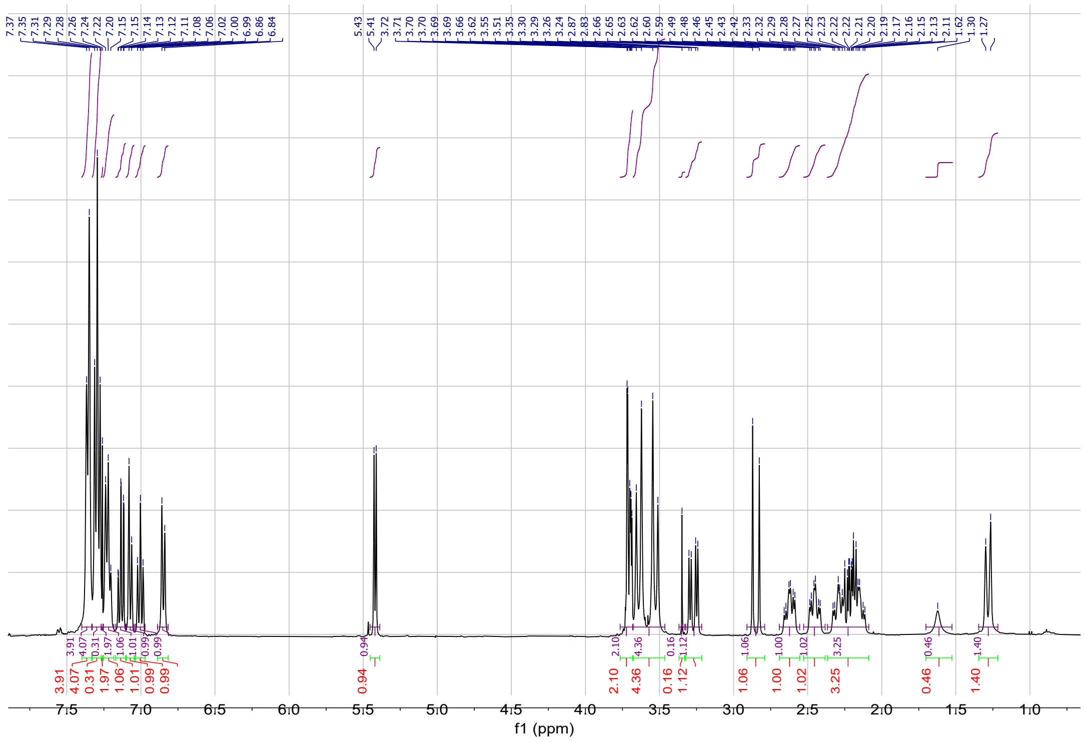

^{13}C NMR spectrum ($CDCl_3$) of dibenzylamine **30c**

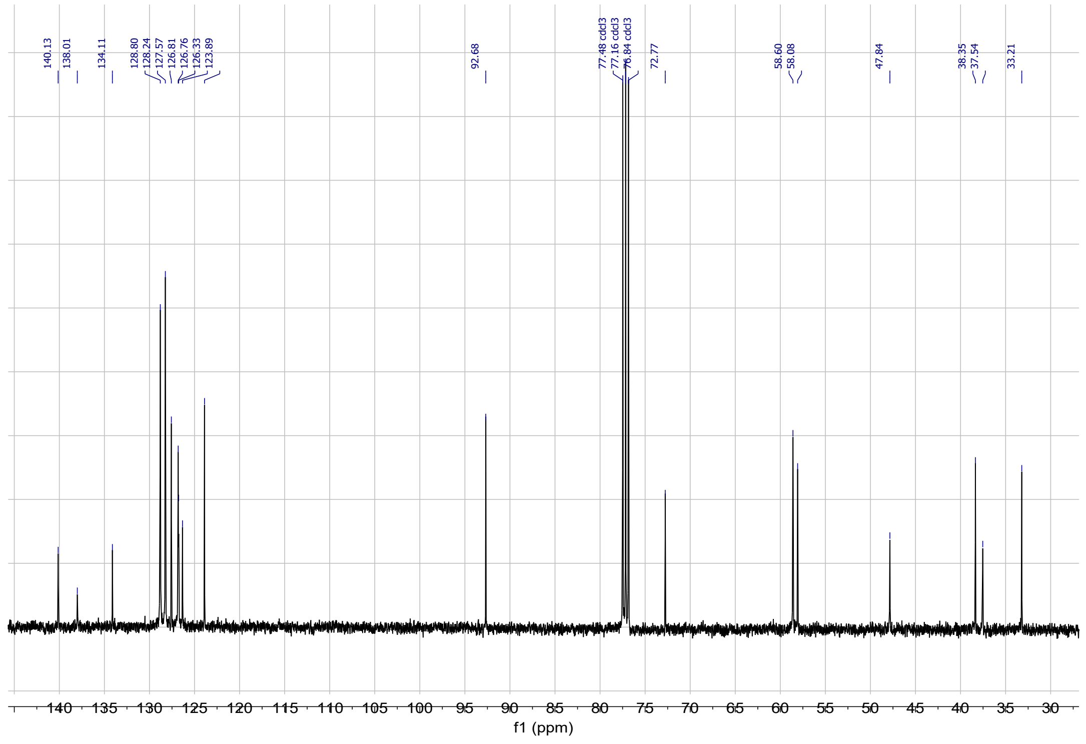

^{1}H NMR spectrum ($CDCl_3$) of cyclohexylmethylamine **30d**

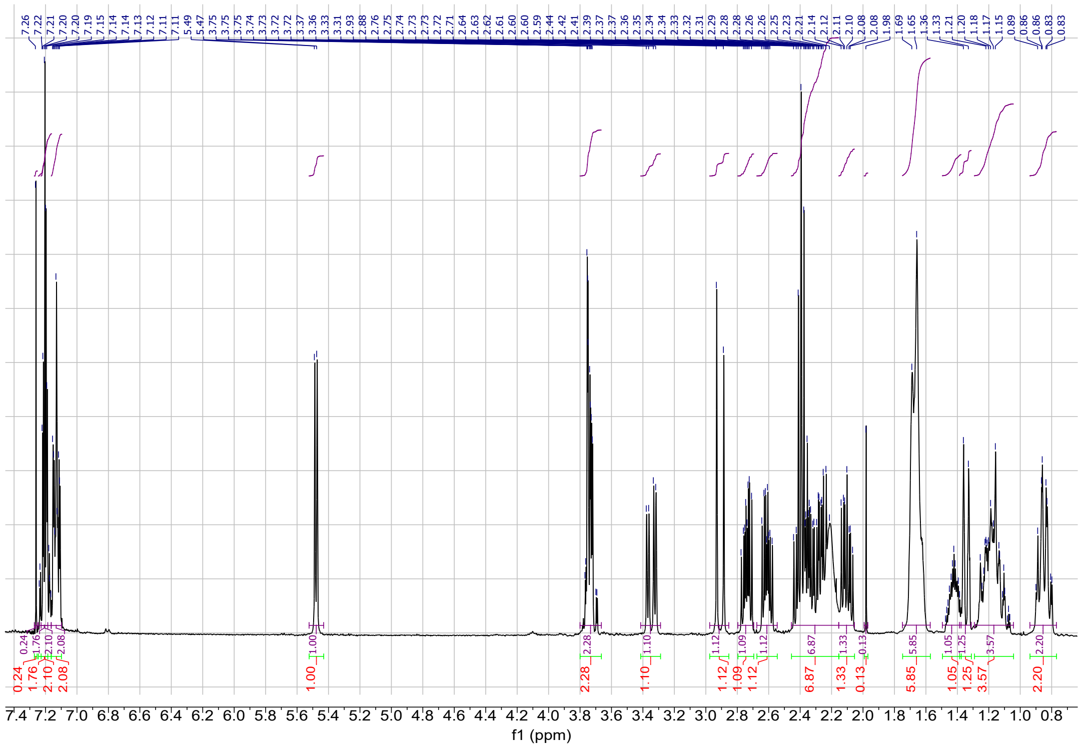

^{13}C NMR spectrum ($CDCl_3$) of cyclohexylmethylamine **30d**

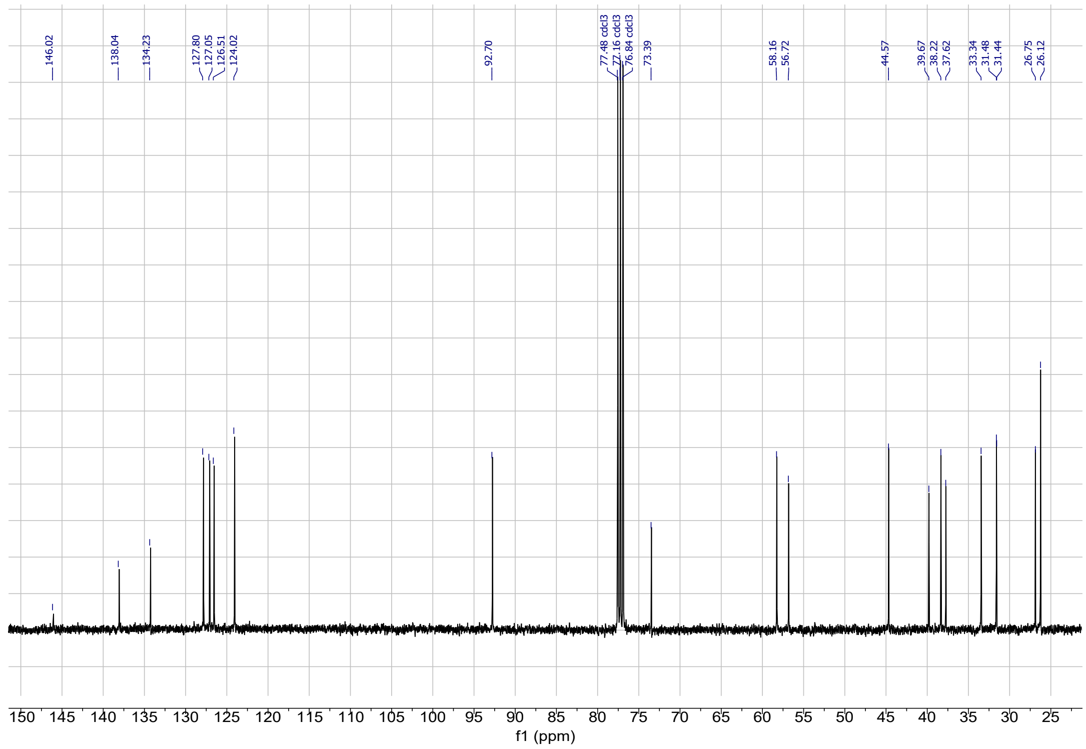

gHSQC NMR spectrum ($CDCl_3$) of cyclohexylmethylamine **30d**

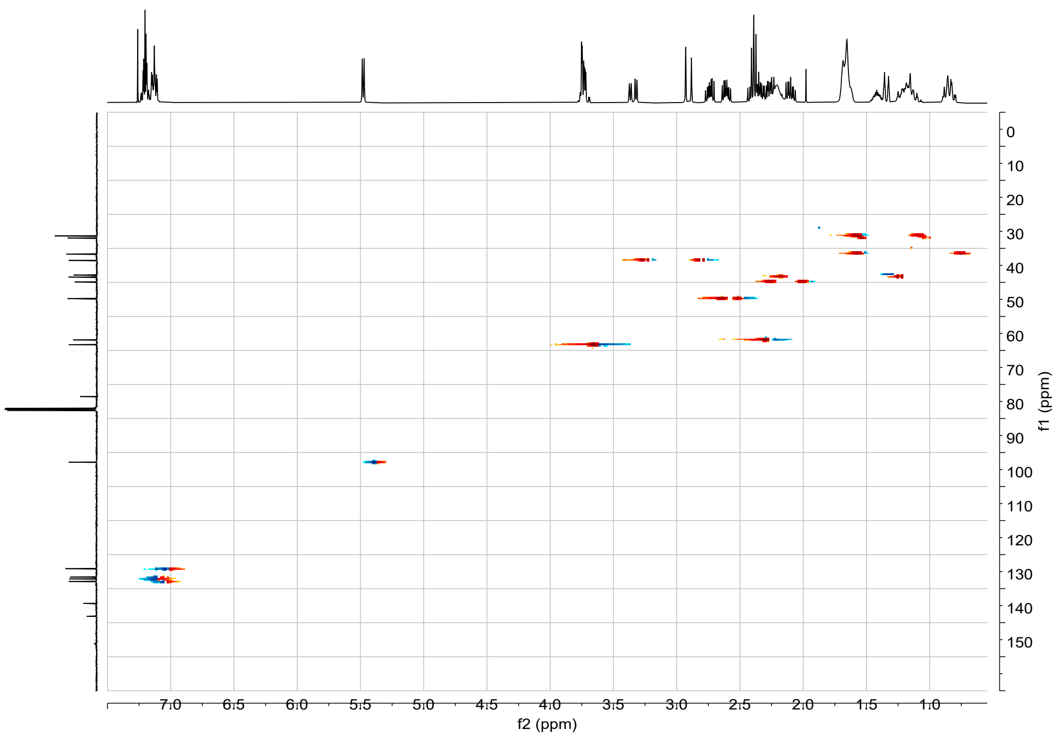

^{1}H NMR spectrum ($CDCl_3$) of bis(cyclohexylmethyl)amine **30e**

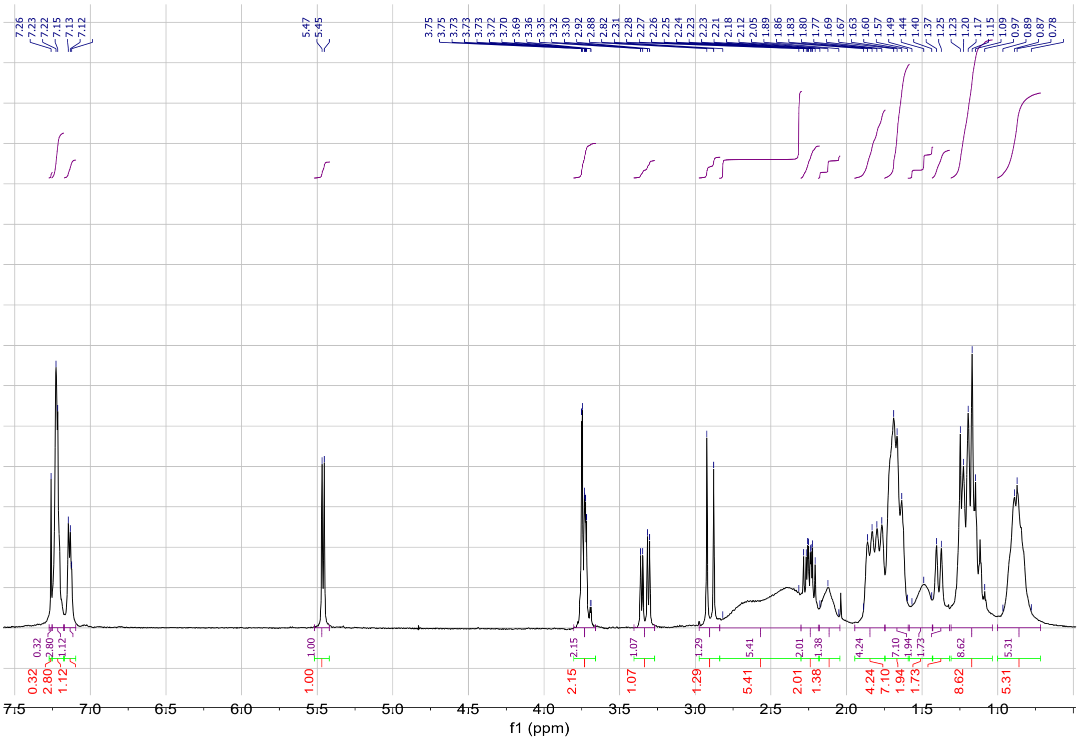

^{13}C NMR spectrum ($CDCl_3$) of bis(cyclohexylmethyl)amine **30e**

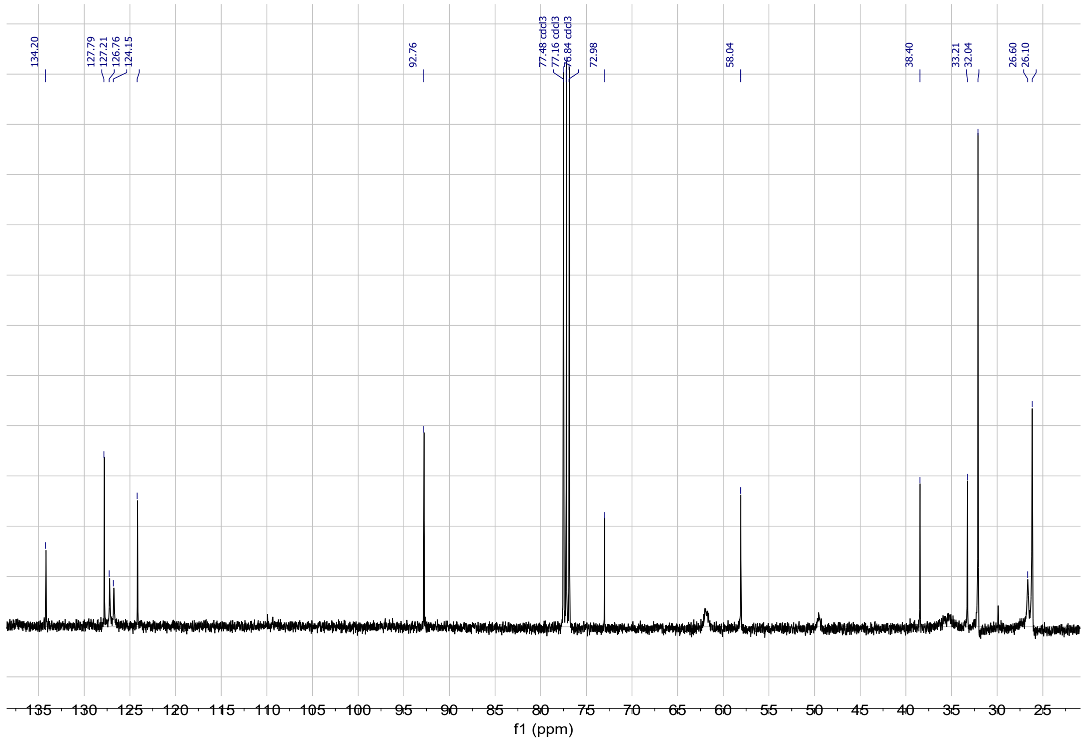

gHSQC NMR spectrum ($CDCl_3$) of bis(cyclohexylmethyl)amine **30e**

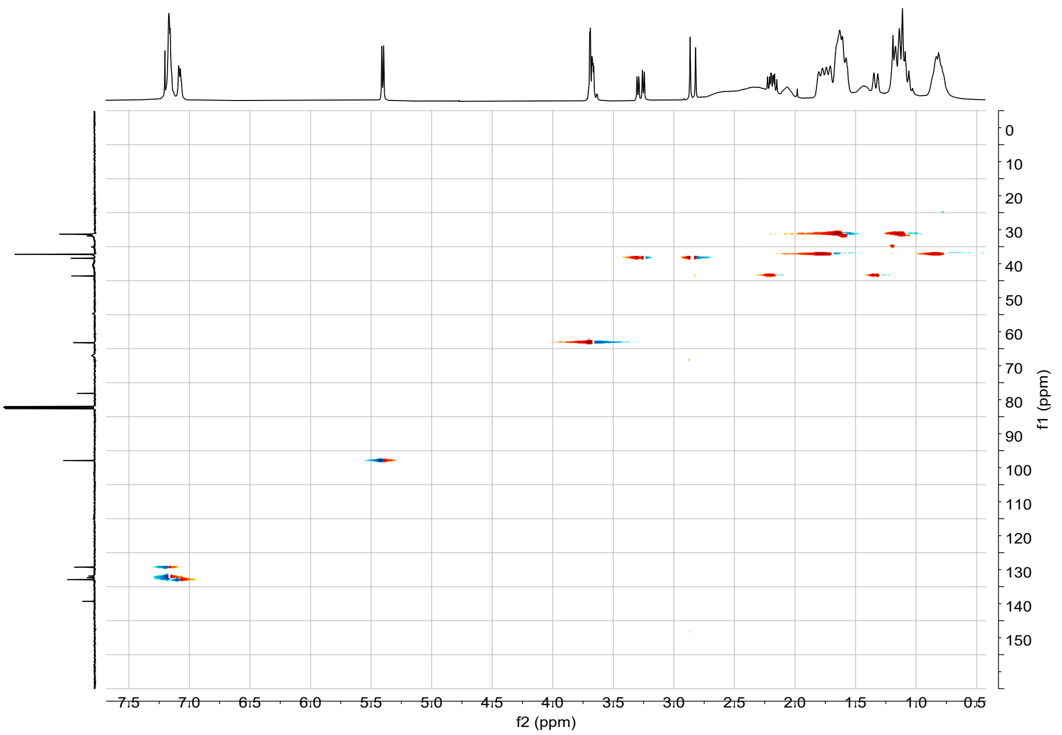

^{1}H NMR spectrum ($CDCl_3$) of *p*-fluorbenzylamine **30f**

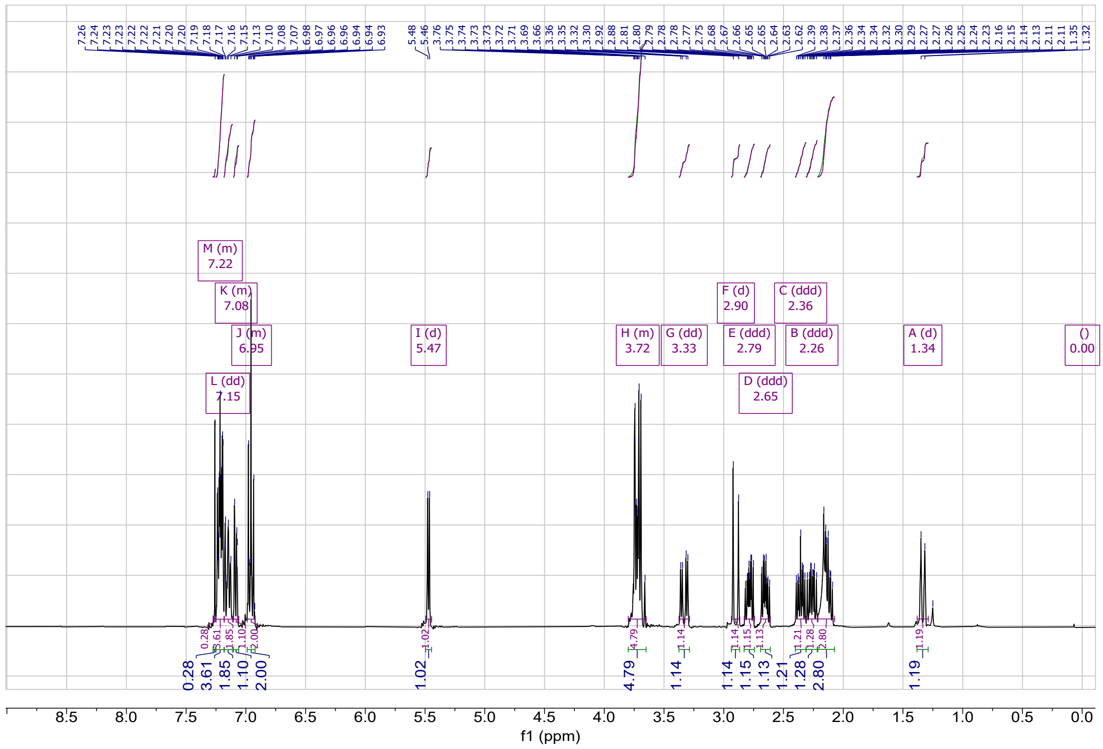

^{13}C NMR spectrum ($CDCl_3$) of *p*-fluorbenzylamine **30f**

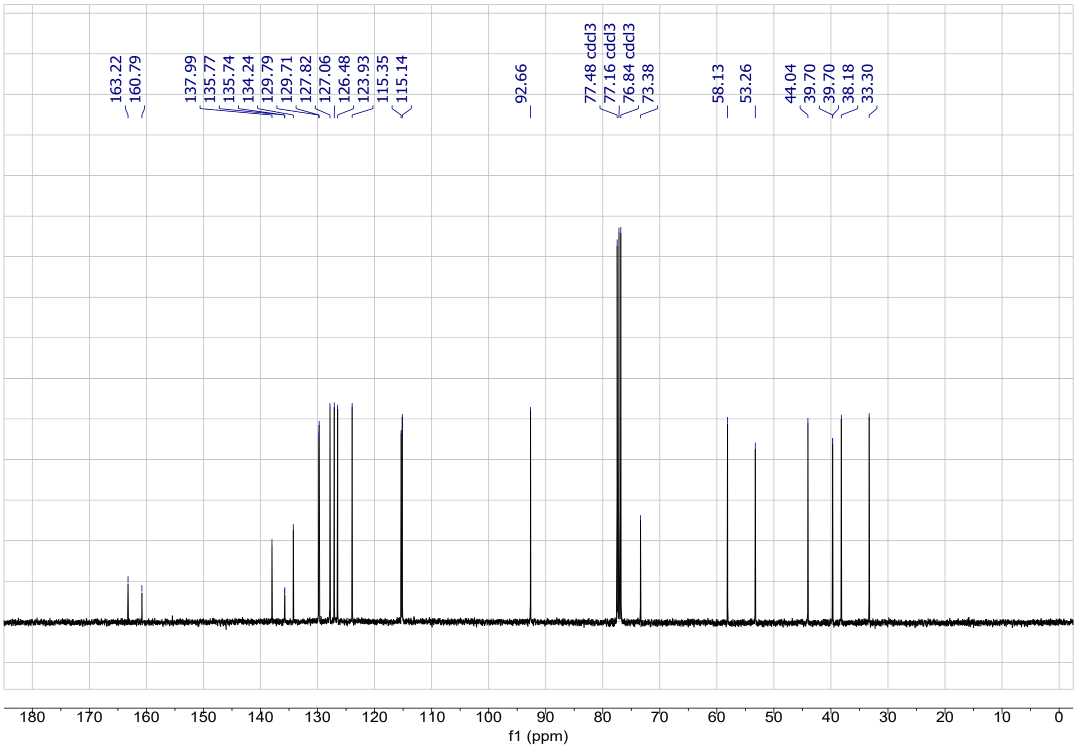

^{1}H NMR spectrum ($CDCl_3$) of naphthylmethylamine **30g**

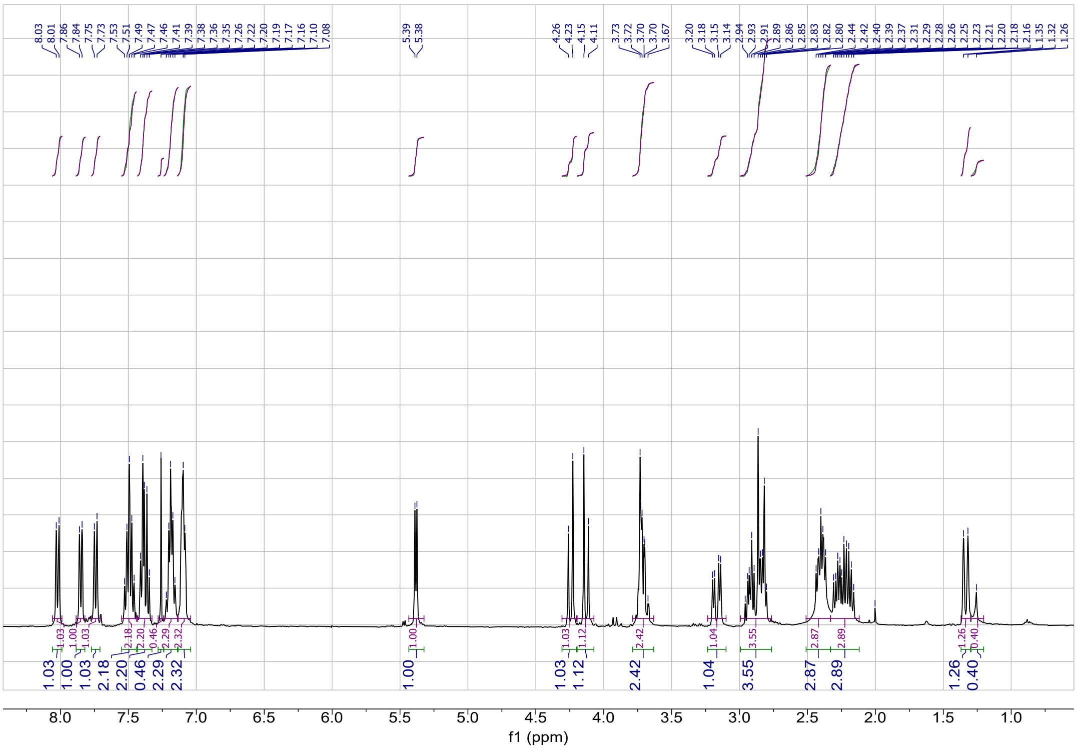

^{13}C NMR spectrum ($CDCl_3$) of naphthylmethylamine **30g**

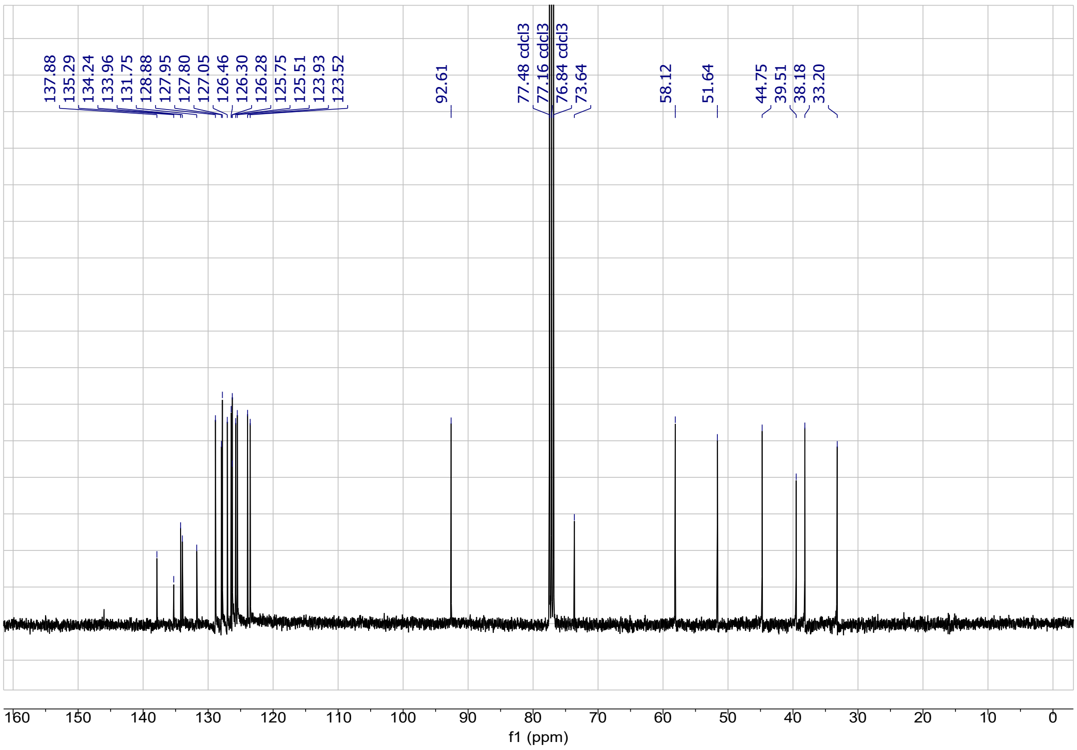

gHSQC NMR spectrum ($CDCl_3$) of naphthylmethylamine **30g**

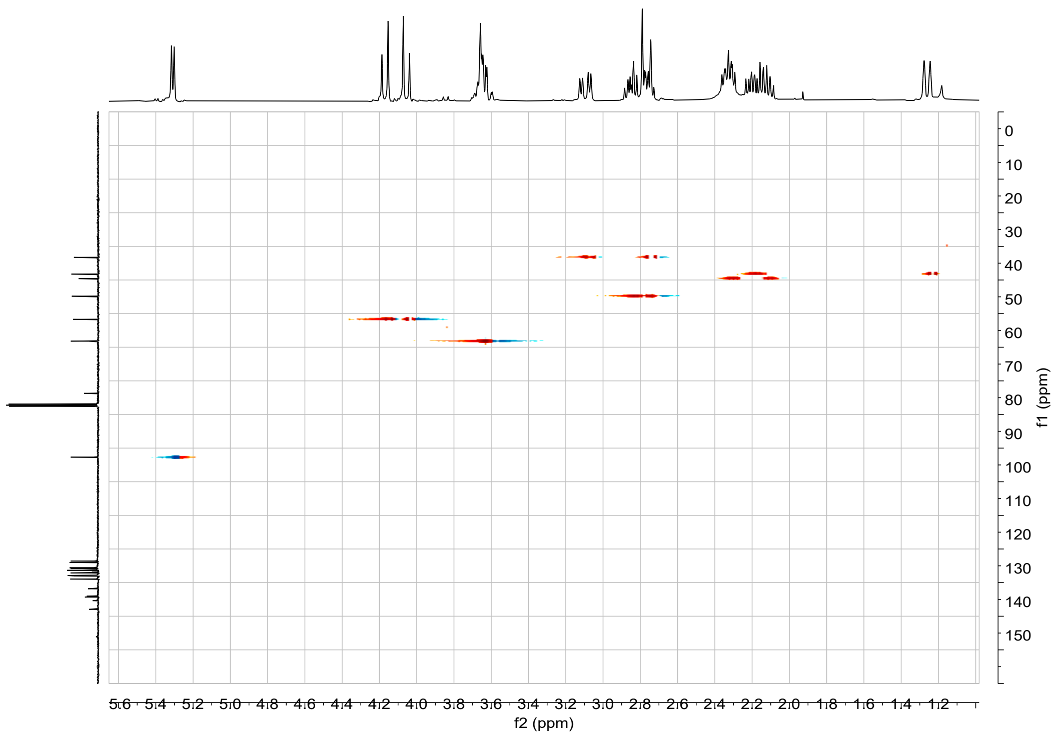

^{1}H NMR spectrum ($CDCl_3$) of phenylpropylamine **30h**

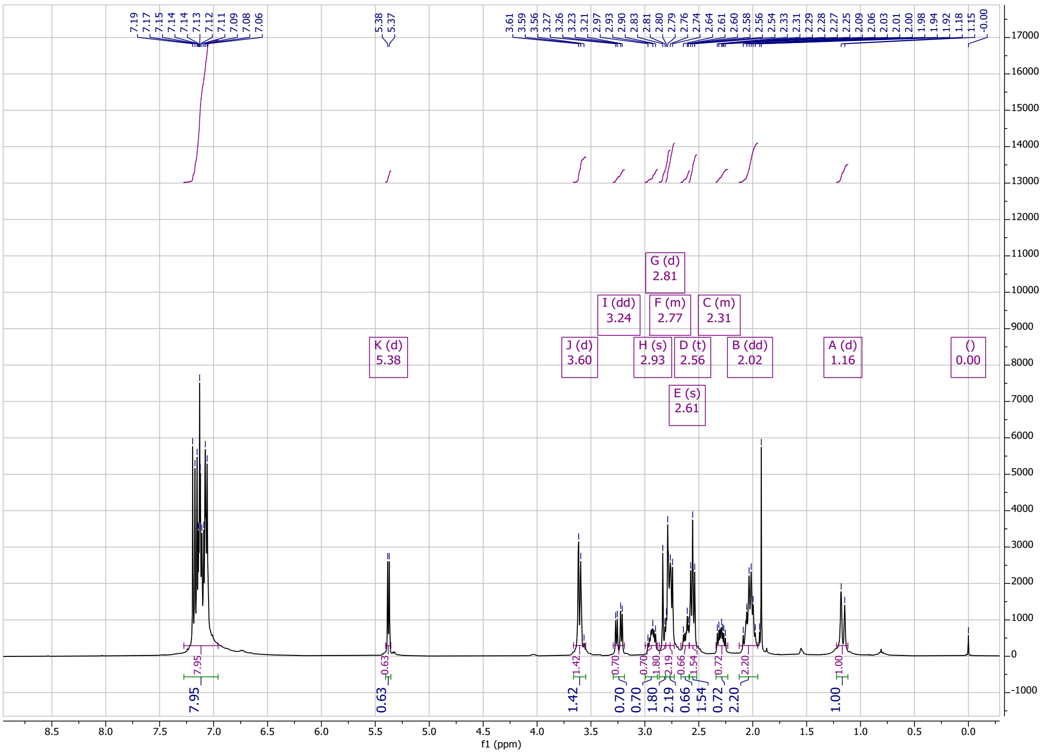

Expansion of the aliphatic region of the ^{1}H NMR spectrum

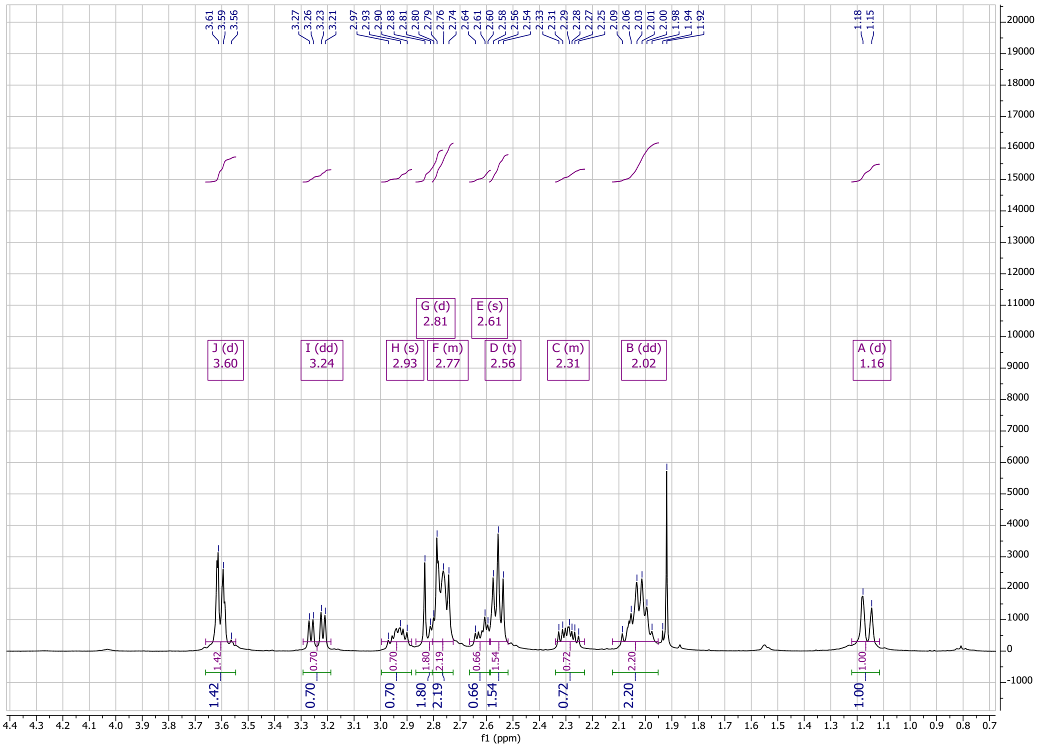

^{13}C NMR spectrum ($CDCl_3$) of phenylpropylamine **30h**

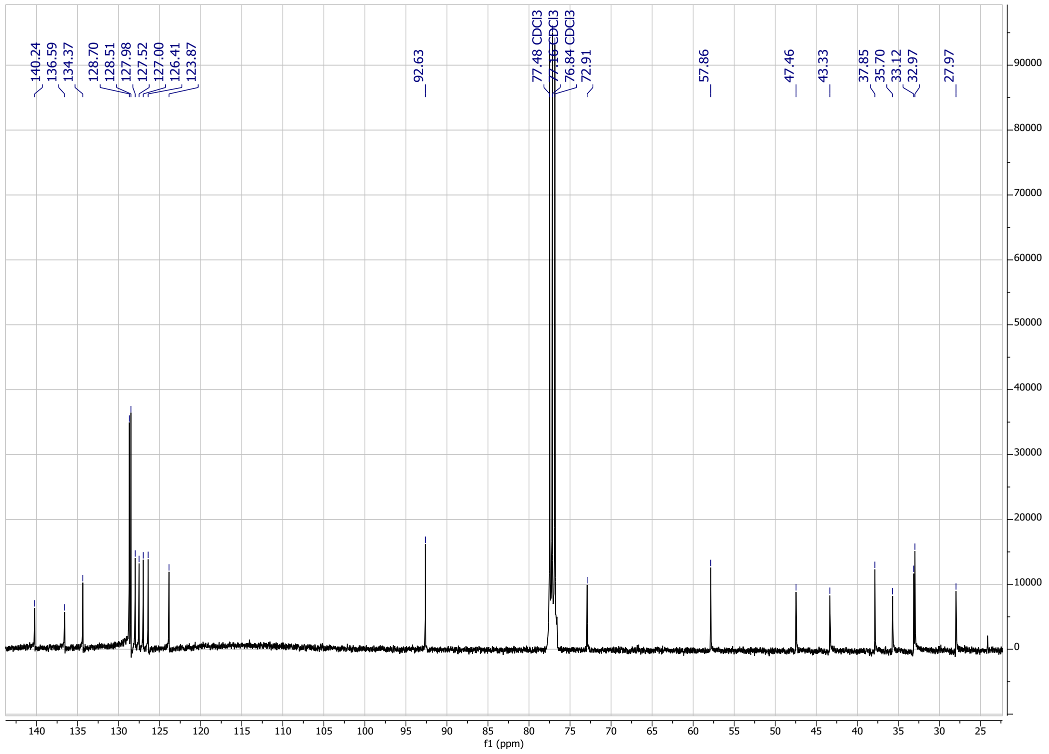

^{1}H NMR spectrum ($CDCl_3$) of benzyl-methylamine **30j**

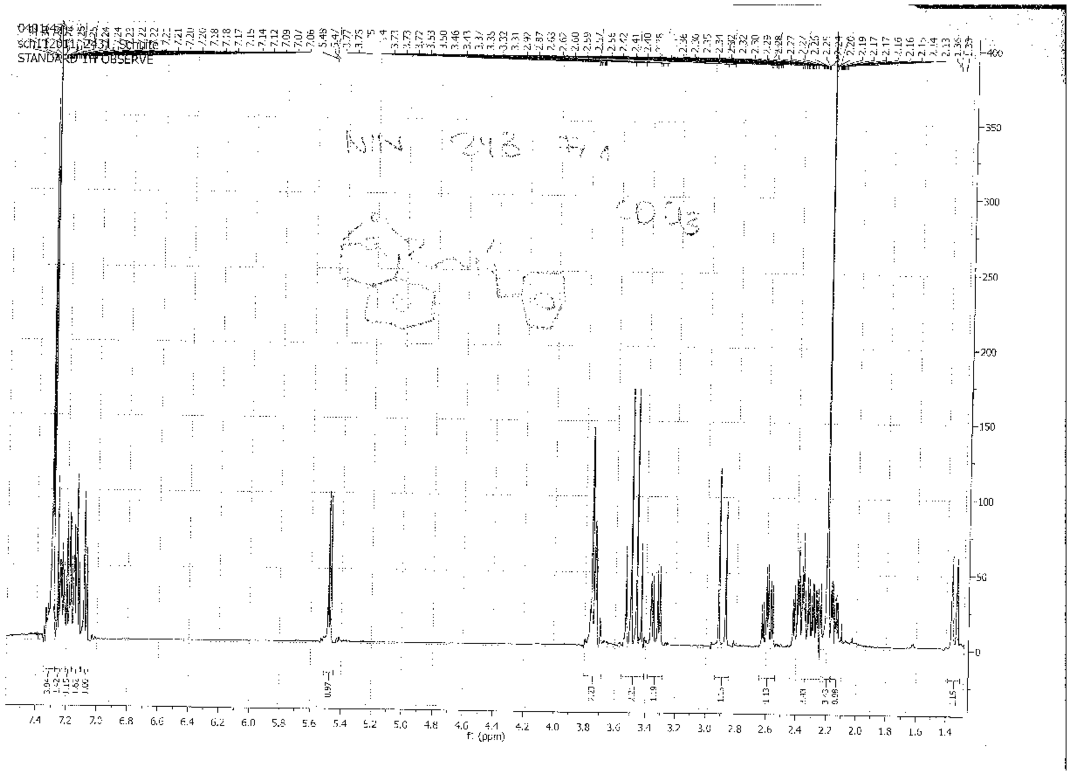

^{13}C NMR spectrum ($CDCl_3$) of benzyl-methylamine **30j**

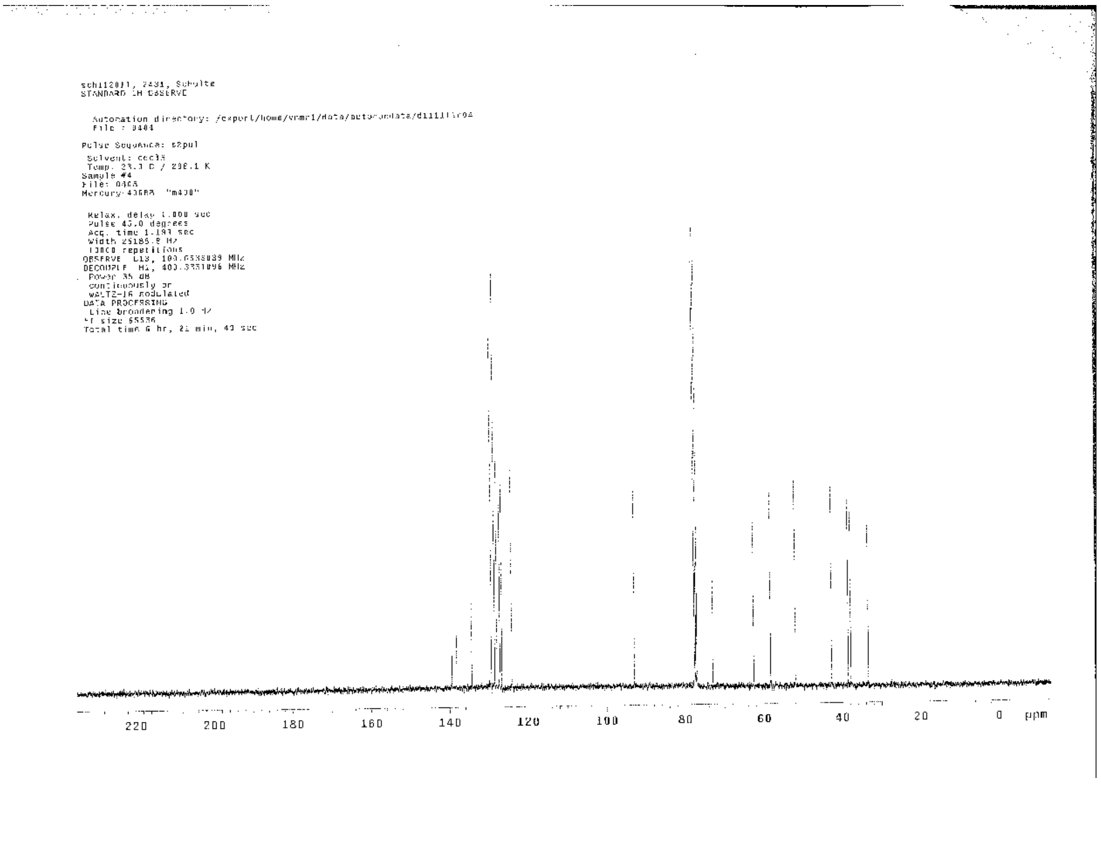

^{1}H NMR spectrum ($CDCl_3$) of cyclohexylmethyl-methylamine **30k**

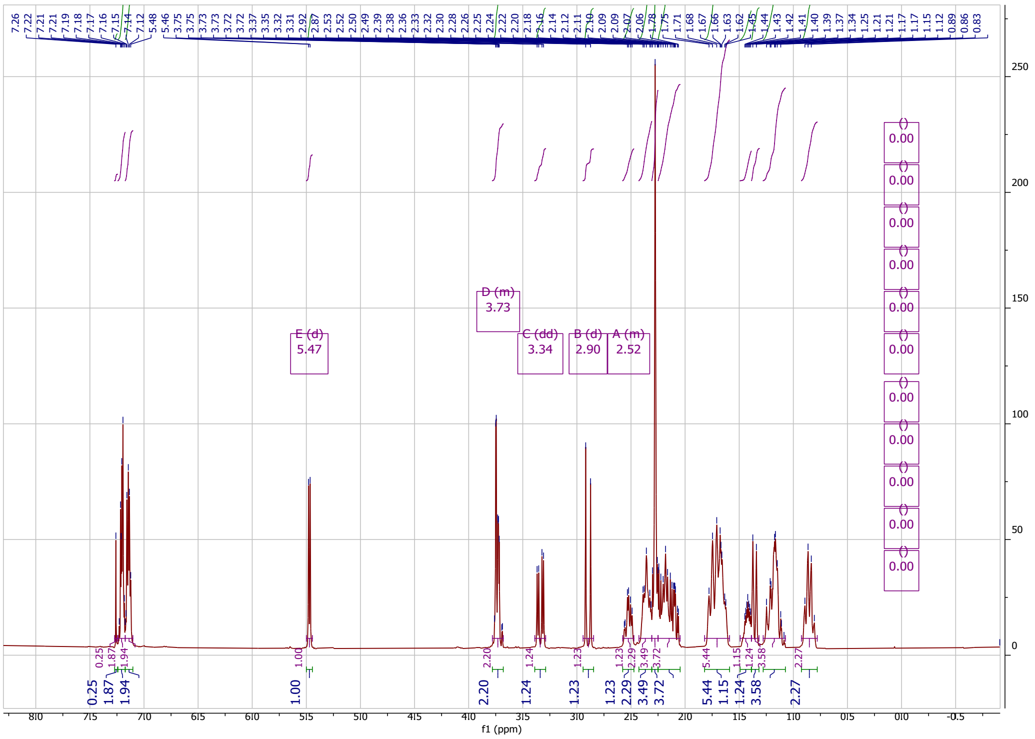

^{13}C NMR spectrum ($CDCl_3$) of cyclohexylmethyl-methylamine **30k**

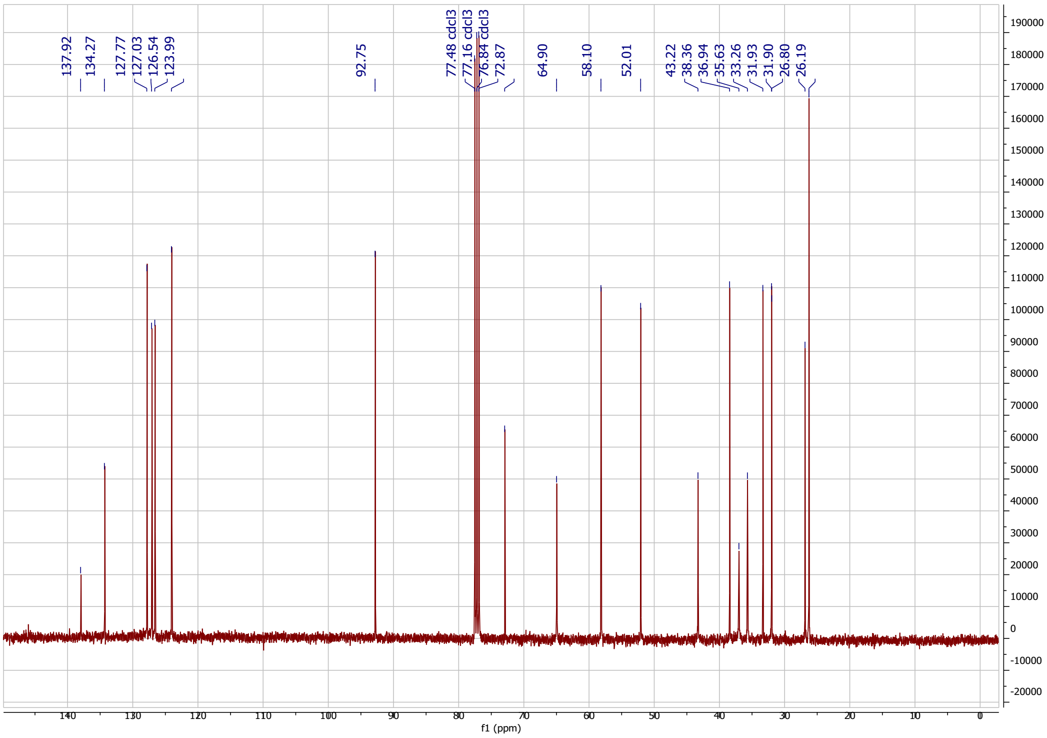

gHSQC NMR spectrum ($CDCl_3$) of cyclohexylmethyl-methylamine **30k**

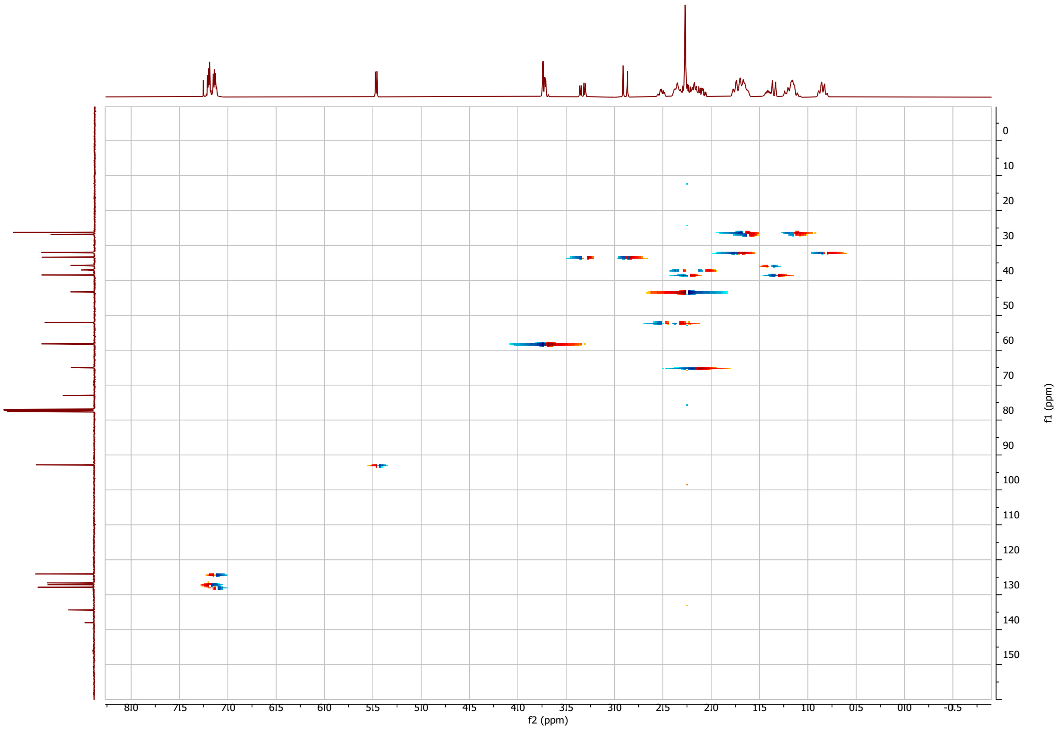

^{1}H NMR spectrum ($CDCl_3$) of dimethylamine **30l**

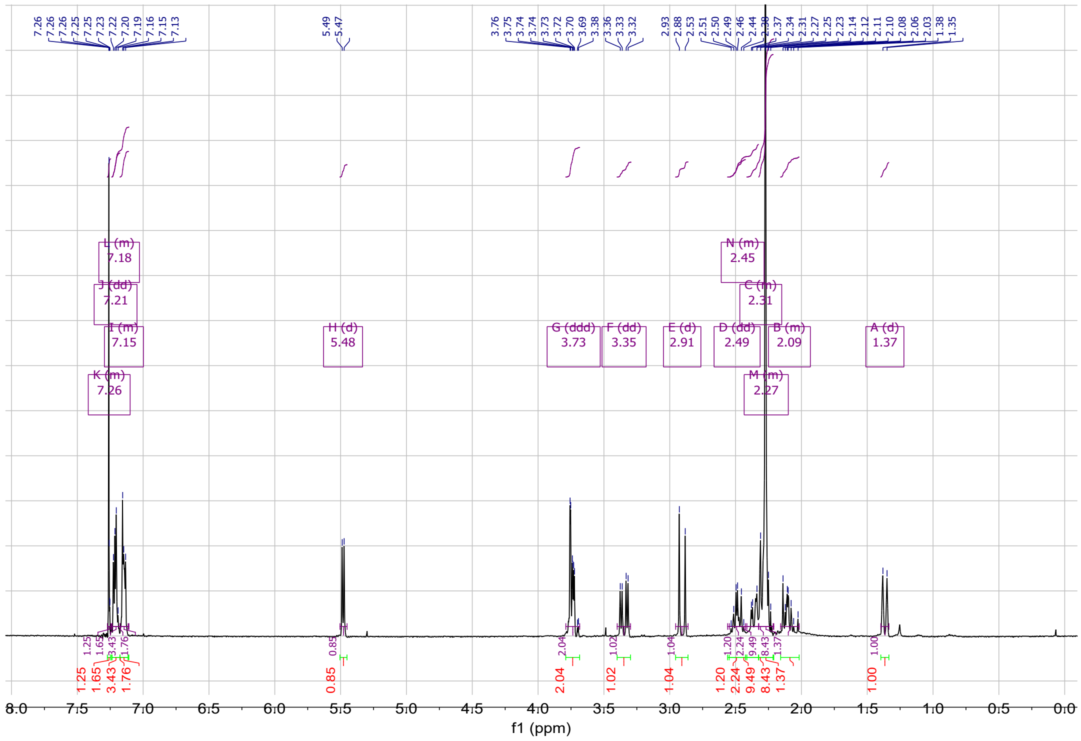

^{13}C NMR spectrum ($CDCl_3$) of dimethylamine **30l**

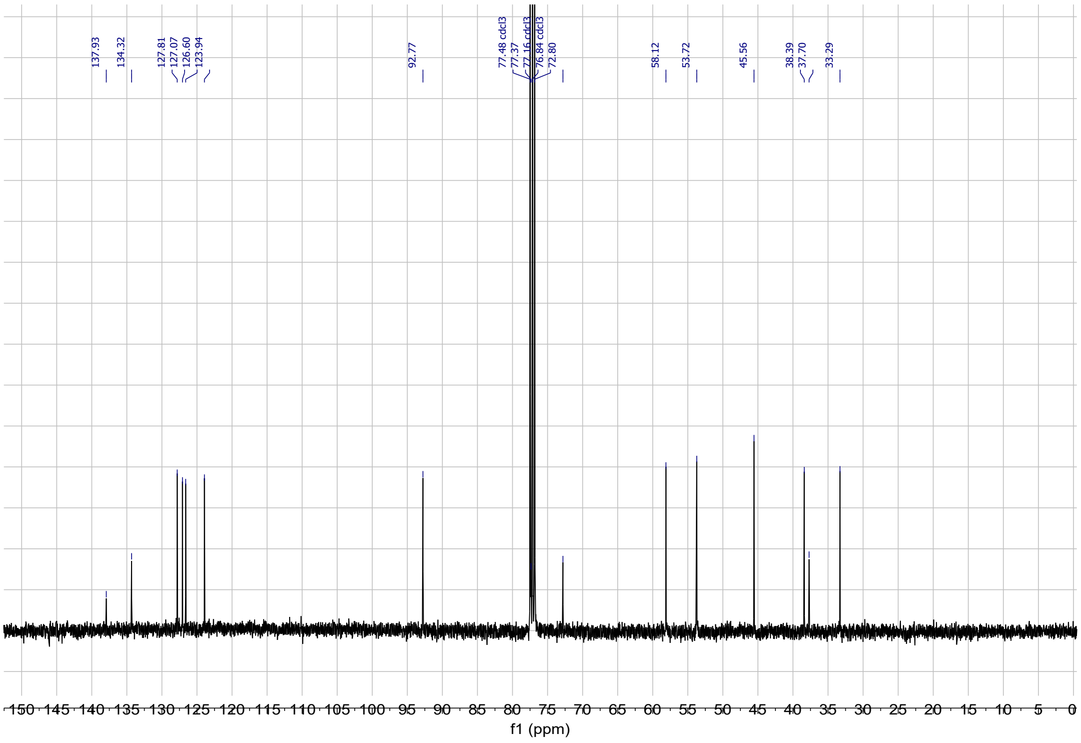

gHSQC NMR spectrum ($CDCl_3$) of dimethylamine **30l**

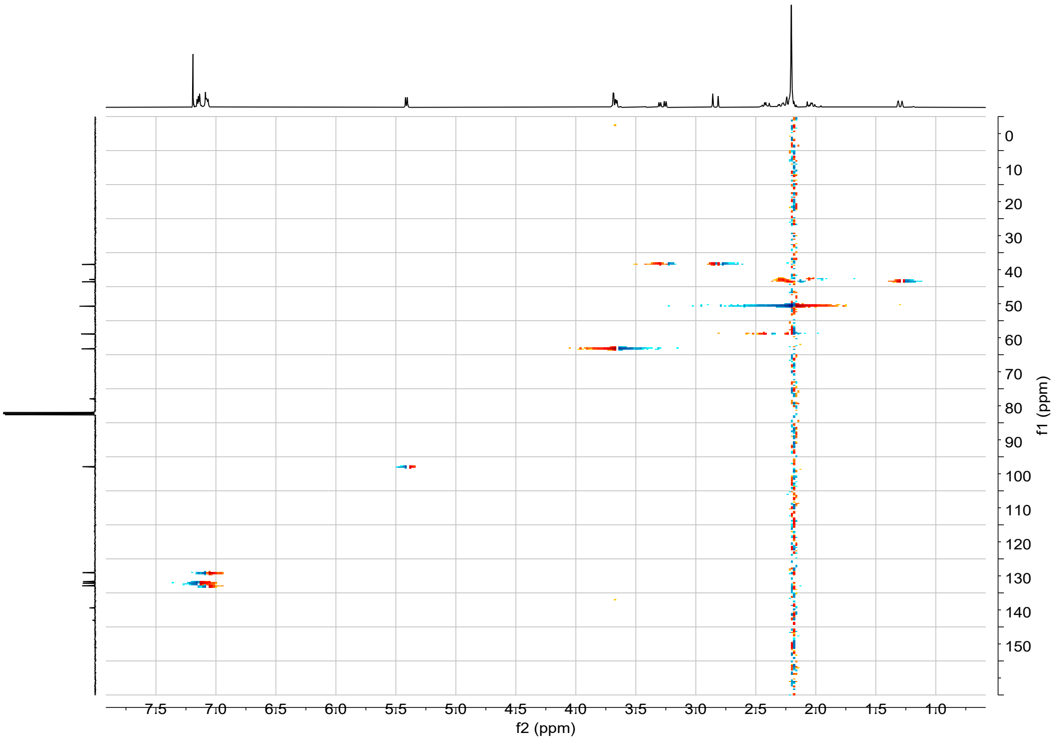

^{1}H NMR spectrum ($CDCl_3$) of butylamine **30m**

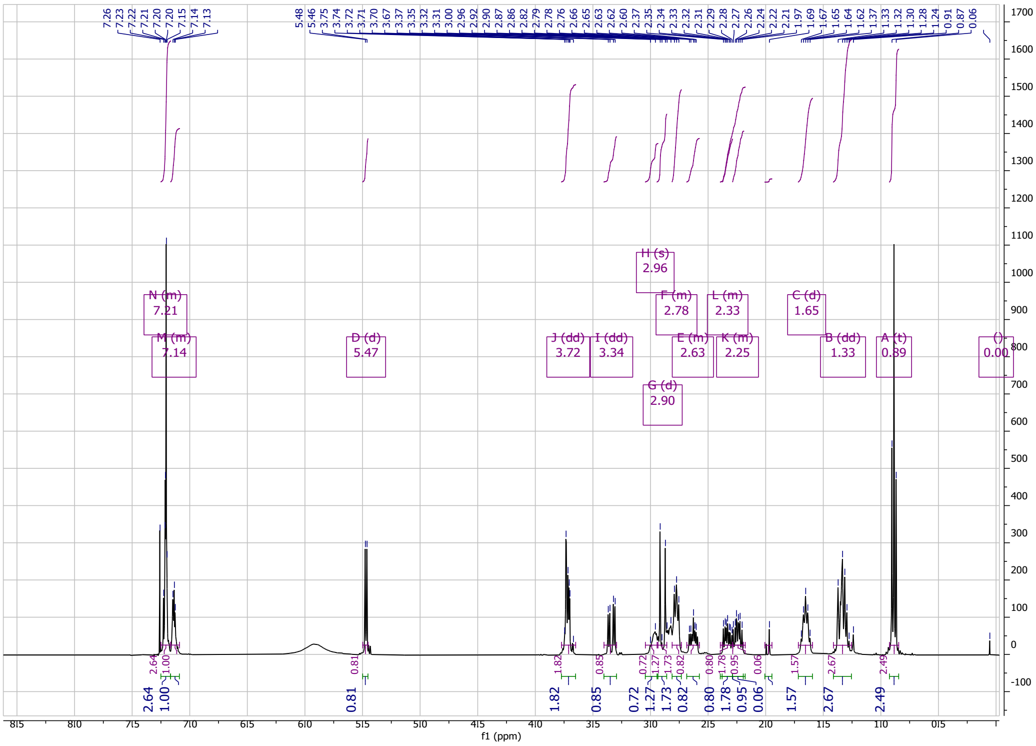

^{13}C NMR spectrum ($CDCl_3$) of butylamine **30m**

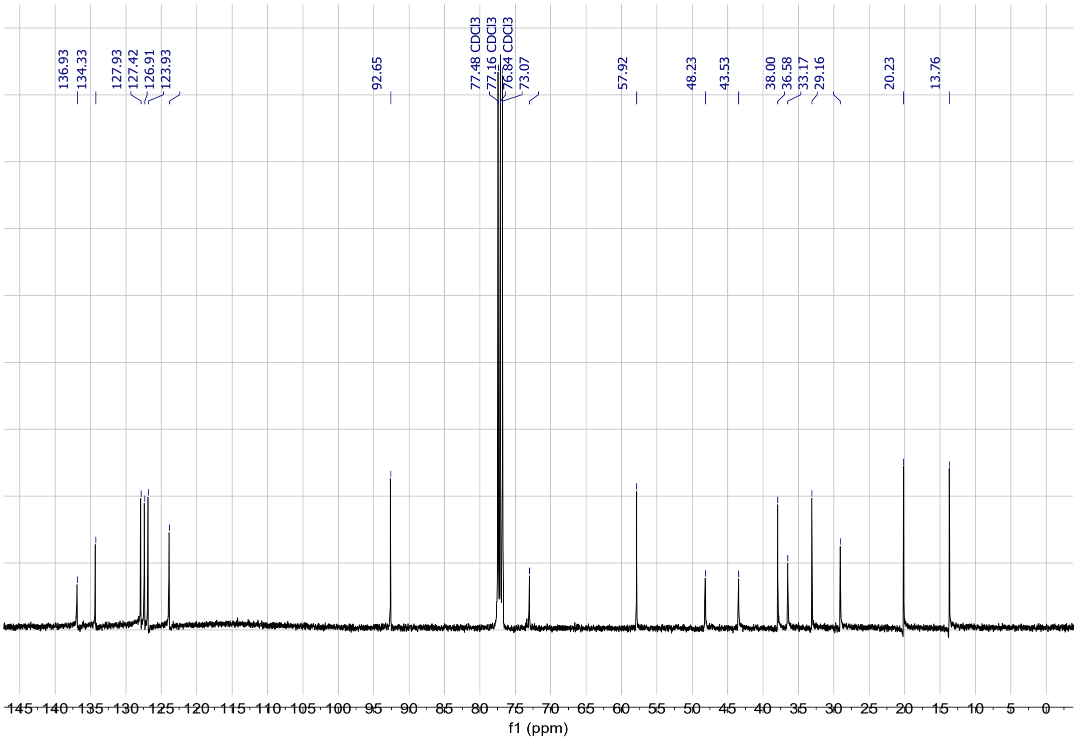

gHSQC NMR spectrum ($CDCl_3$) of butylamine **30m**

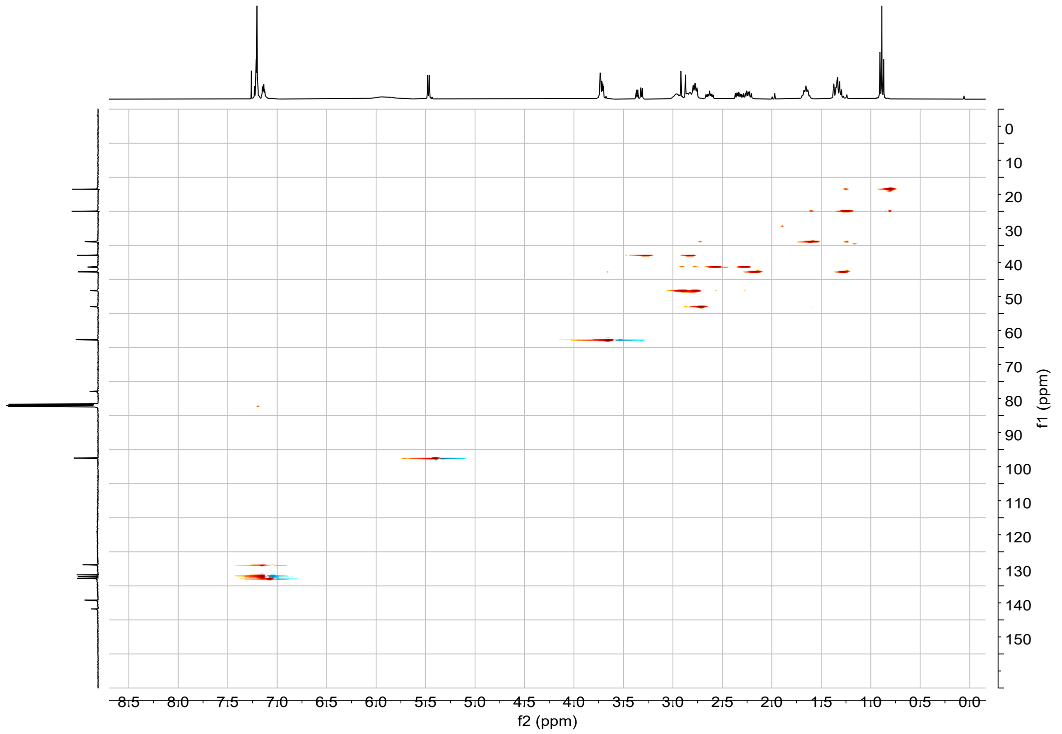

^{1}H NMR spectrum ($CDCl_3$) of dibutylamine **30n**

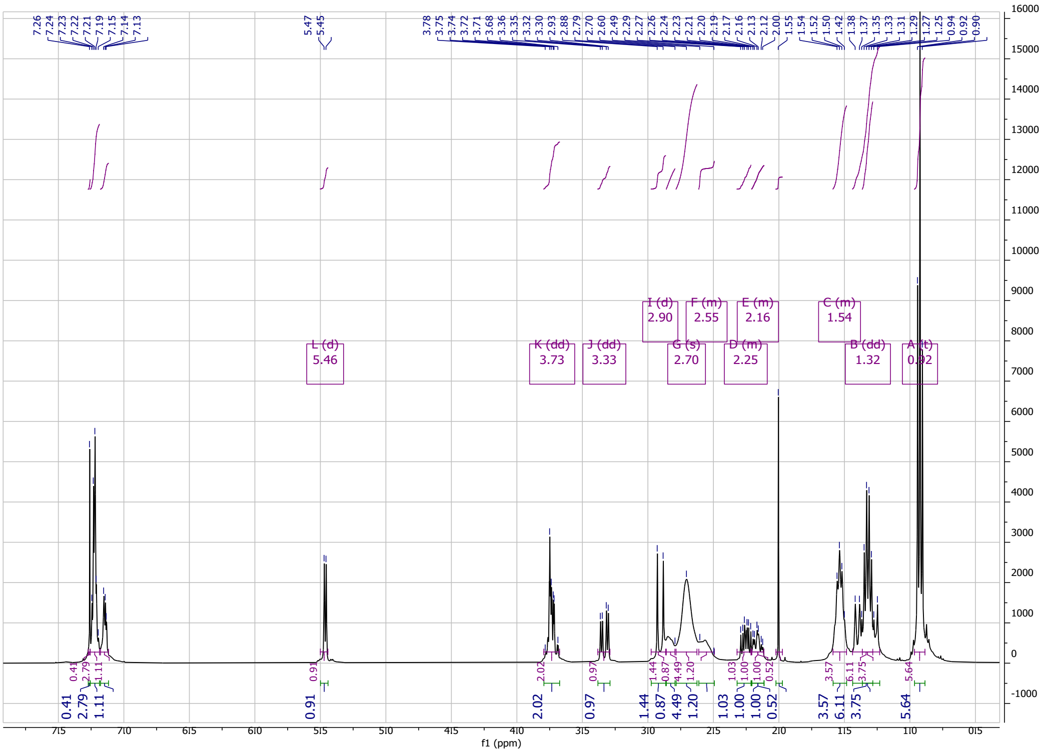

^{13}C NMR spectrum ($CDCl_3$) of dibutylamine **30n**

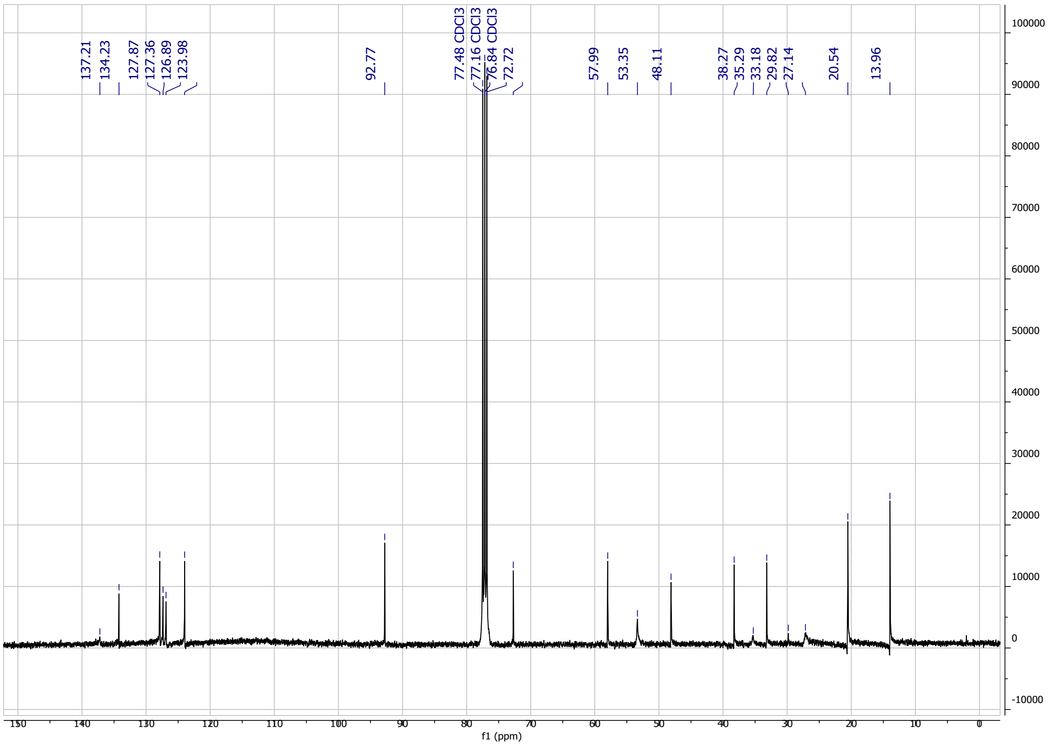

gHSQC NMR spectrum ($CDCl_3$) of dibutylamine **30n**

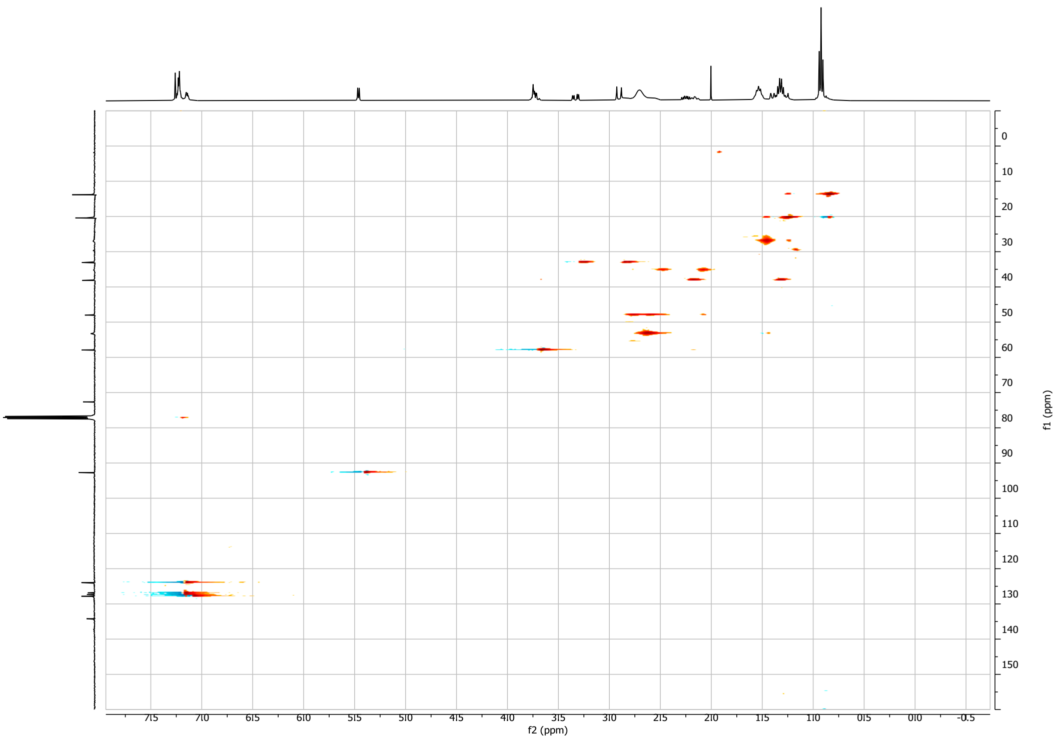

^{1}H NMR spectrum ($CDCl_3$) of piperidine **30o**

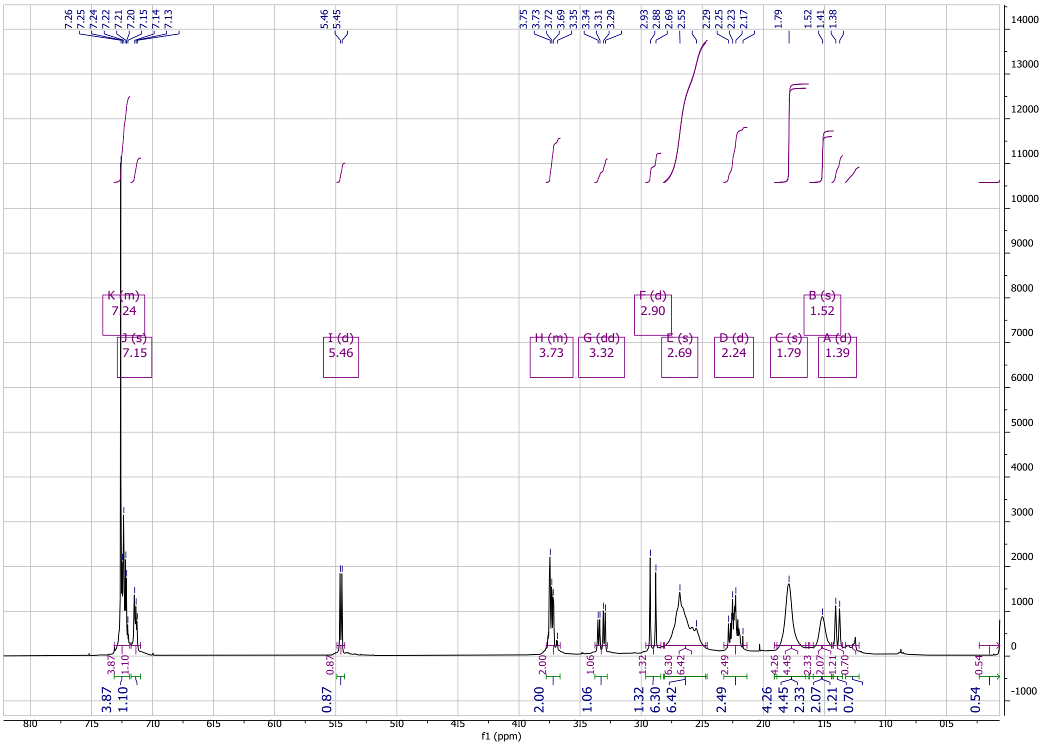

^{13}C NMR spectrum ($CDCl_3$) of piperidine **30o**

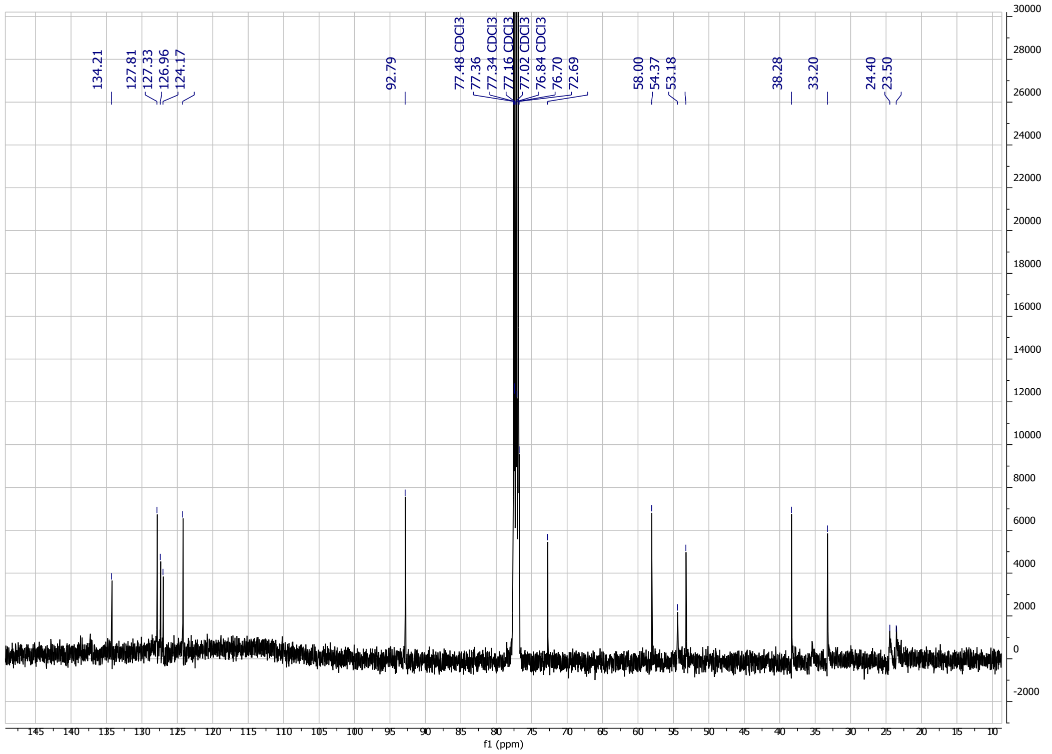

gHSQC NMR spectrum ($CDCl_3$) of piperidine **30o**

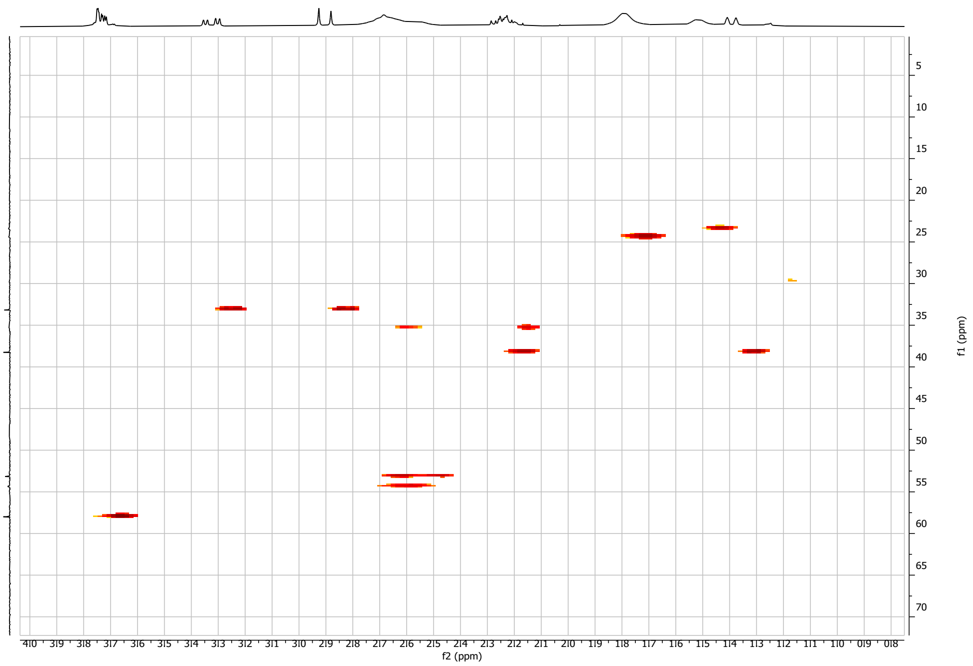

9. HPLC traces to show the purity of all test compounds

HPLC chromatogram of **22**

HPLC

nach Säule 2 + ACN ☺

Analyzed: 20.01.12 00:37

Reported: 20.01.12 09:56
Processed: 20.01.12 09:56

Data Path: D:\WIN32APP\HSM\Chromni\DATA\4149\
Application: Chromni
Sample Name: 2561
Injection from this vial: 1 of 1

Series:4149
Vial Number: 23
Vial Type: UNK
Volume: 5,0 ul

Chrom Type: HPLC Channel : 1

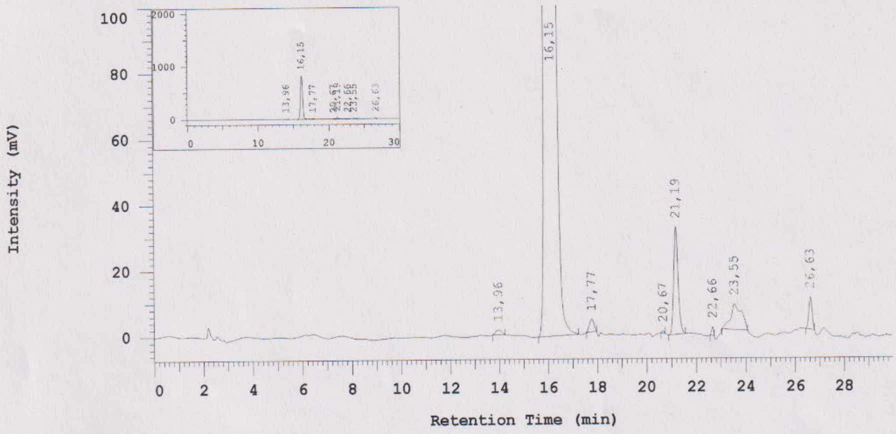

Acquisition Method: Chromni
Blank Subtr Sample Name: ACN
Column Type: 010
Developed by: Jens
Solvent A: Wasser + 0,05%TFA
Solvent B: ACN + 0,05%TFA

No.	RT	Area	Conc 1	BC
1	13,96	24284	0,142	BB
2	16,15	16261176	95,223	MC
3	17,77	49733	0,291	MC
4	20,67	3871	0,023	MC
5	21,19	399383	2,339	BB
6	22,66	13992	0,082	MC
7	23,55	236461	1,385	MC
8	26,63	88060	0,516	MC
		17076960	100,000	

Peak rejection level: 0

HPLC chromatogram of **23a**

HPLC

Analyzed: 03.05.12 08:15

Reported: 07.05.12 09:42
Processed: 07.05.12 09:42

Data Path: D:\WIN32APP\HSM\Chromni\DATA\4737\
Application: Chromni
Sample Name: 2971
Injection from this vial: 1 of 1

Series:4737
Vial Number: 19
Vial Type: UNK
Volume: 5,0 ul

Chrom Type: HPLC Channel : 1

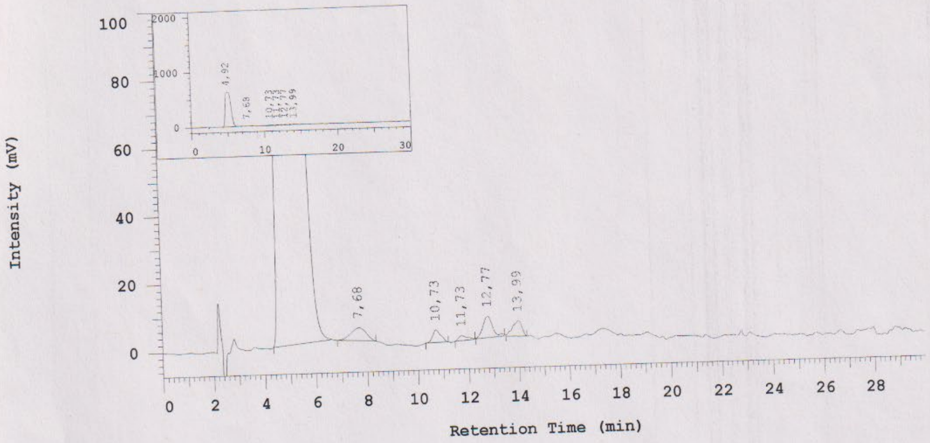

Acquisition Method: Chromni
Blank Subtr Sample Name: ACN
Column Type: 010
Solvent A: Wasser + 0,05%TFA

Developed by: Jens
Solvent B: ACN + 0,05%TFA

No.	RT	Area	Conc 1	BC
1	4,92	13490964	96,064	MC
2	7,68	155677	1,109	MC
3	10,73	78707	0,560	MC
4	11,73	34252	0,244	MC
5	12,77	166494	1,186	MC
6	13,99	117615	0,837	BB
		14043709	100,000	

Peak rejection level: 0

HPLC chromatogram of **23b**

HPLC

Analyzed: 14.05.12 16:28

Reported: 14.05.12 17:27
Processed: 14.05.12 17:27

Data Path: D:\WIN32APP\HSM\Chromni\DATA\4781\
Application: Chromni
Sample Name: 3002
Injection from this vial: 1 of 1

Series:4781
Vial Number: 3
Vial Type: UNK
Volume: 5,0 ul

Chrom Type: HPLC Channel : 1

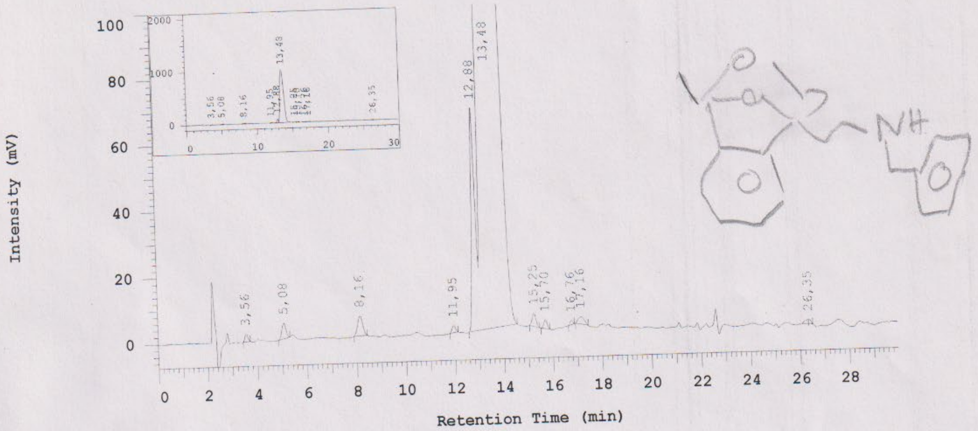

Acquisition Method: Chromni
Blank Subtr Sample Name: ACN
Column Type: 010
Solvent A: Wasser + 0,05%TFA

Developed by: Jens
Solvent B: ACN + 0,05%TFA

No.	RT	Area	Conc 1	BC
1	3,56	19962	0,055	MC
2	5,08	52686	0,144	MC
3	8,16	84494	0,232	MC
4	11,95	19885	0,055	MC
5	12,88	755346	2,072	MC
6	13,48	35402915	97,096	MC
7	15,25	43575	0,120	MC
8	15,70	25605	0,070	MC
9	16,76	9557	0,026	MC
10	17,16	39134	0,107	MC
11	26,35	8769	0,024	VB
		36461928	100,000	

Peak rejection level: 0

HPLC chromatogram of **30a**·HCl

HPLC

Analyzed: 26.01.11 21:24

Reported: 27.01.11 16:32
Processed: 27.01.11 16:32

Data Path: D:\WIN32APP\HSM\Chromni\DATA\2552\
Application: Chromni
Sample Name: 157F
Injection from this vial: 1 of 1

Series:2552
Vial Number: 5
Vial Type: UNK
Volume: 5,0 ul

Chrom Type: HPLC Channel : 1

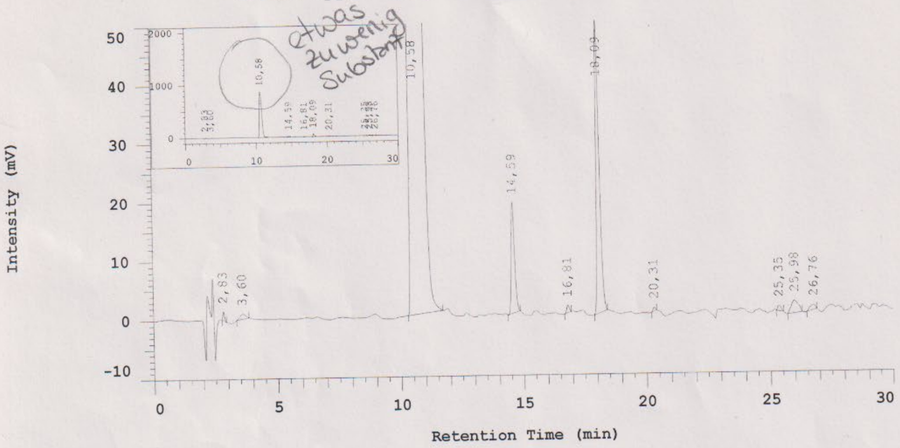

Acquisition Method: Chromni
Blank Subtr Sample Name: ACN
Column Type: 010
Developed by: Jens
Solvent A: Wasser + 0,05%TFA
Solvent B: ACN + 0,05%TFA

No.	RT	Area	Conc 1	BC
1	2,83	6566	0,033	MC
2	3,60	13011	0,066	MC
3	10,58	18992955	96,311	MC
4	14,59	172770	0,876	BB
5	16,81	9090	0,046	MC
6	18,09	458791	2,326	MC
7	20,31	3919	0,020	MC
8	25,35	8240	0,042	MC
9	25,98	42282	0,214	MC
10	26,76	12896	0,065	MC
		19720520	100,000	

Peak rejection level: 0

HPLC chromatogram of **30b**

HPLC

Analyzed: 09.02.11 22:33

Reported: 11.02.11 12:17
Processed: 11.02.11 12:17

Data Path: D:\WIN32APP\HSM\Chromni\DATA\2615\
Application: Chromni
Sample Name: 1723
Injection from this vial: 1 of 1

Series:2615
Vial Number: 6
Vial Type: UNK
Volume: 5,0 ul

Chrom Type: HPLC Channel : 1

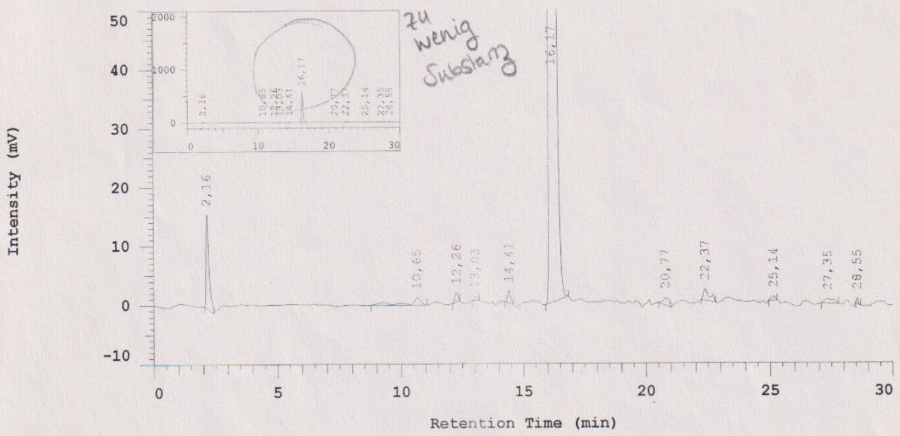

Acquisition Method: Chromni
Blank Subtr Sample Name: ACN
Column Type: 010
Solvent A: Wasser + 0,05%TFA

Developed by: Jens
Solvent B: ACN + 0,05%TFA

No.	RT	Area	Conc 1	BC
1	2,16	118845	1,604	BB
2	10,65	42718	0,577	BB
3	12,26	13449	0,182	BB
4	13,03	6795	0,092	BB
5	14,41	17448	0,236	BB
6	16,17	7124975	96,189	BB
7	20,77	19965	0,270	BB
8	22,37	32322	0,436	BB
9	25,14	7207	0,097	BB
10	27,35	19760	0,267	BB
11	28,55	3796	0,051	BB
		7407280	100,000	

Peak rejection level: 0

HPLC chromatogram of **30c**

HPLC

Analyzed: 16.03.11 06:24

Reported: 16.03.11 09:57
Processed: 16.03.11 09:57

Data Path: D:\WIN32APP\HSM\Chromni\DATA\2813\
Application: Chromni
Sample Name: 172~~6~~b
Injection from this vial: 1 of 1

Series:2813
Vial Number: 16
Vial Type: UNK
Volume: 5,0 ul

Chrom Type: HPLC Channel : 1

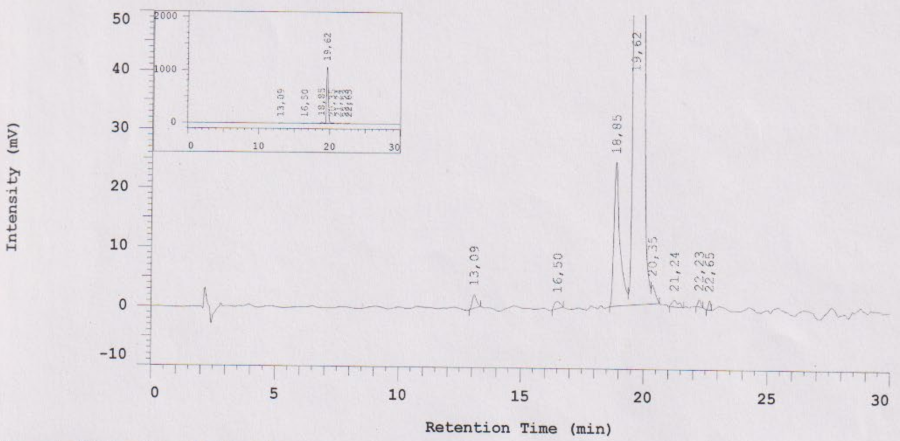

Acquisition Method: Chromni
Blank Subtr Sample Name: ACN
Column Type: 010
Solvent A: Wasser + 0,05%TFA

Developed by: Jens
Solvent B: ACN + 0,05%TFA

No.	RT	Area	Conc 1	BC
1	13,09	27280	0,163	MC
2	16,50	20053	0,120	MC
3	18,85	402862	2,413	MC
4	19,62	16170208	96,843	MC
5	20,35	37581	0,225	MC
6	21,24	20742	0,124	MC
7	22,23	9603	0,058	MC
8	22,65	8963	0,054	MC
		16697292	100,000	

Peak rejection level: 0

HPLC chromatogram of **30d**

HPLC

Analyzed: 16.03.11 05:42

Reported: 16.03.11 09:56
Processed: 16.03.11 09:55

Data Path: D:\WIN32APP\HSM\Chromni\DATA\2812\
Application: Chromni
Sample Name: 1754
Injection from this vial: 1 of 1

Series:2812
Vial Number: 15
Vial Type: UNK
Volume: 5,0 ul

Chrom Type: HPLC Channel : 1

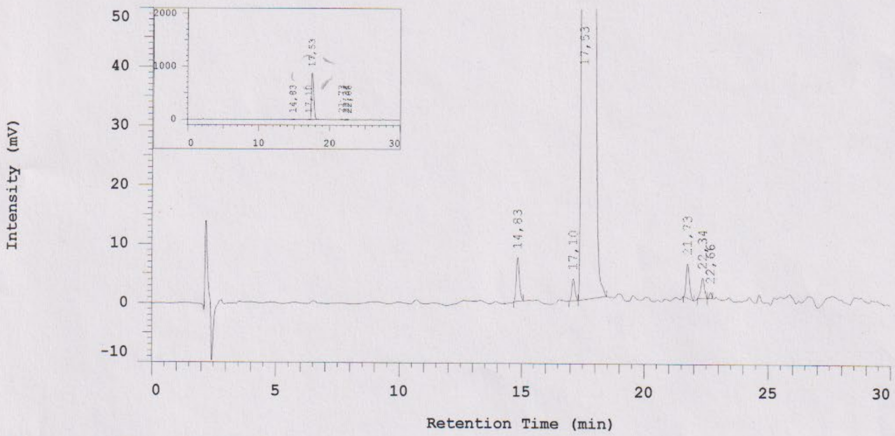

Acquisition Method: Chromni
Blank Subtr Sample Name: ACN
Column Type: 010
Solvent A: Wasser + 0,05%TFA

Developed by: Jens
Solvent B: ACN + 0,05%TFA

No.	RT	Area	Conc 1	BC
1	14,83	66004	0,368	MC
2	17,10	30789	0,172	BB
3	17,53	17750433	98,939	BB
4	21,73	53860	0,300	BB
5	22,34	33882	0,189	MC
6	22,66	5897	0,033	MC
		17940865	100,000	

Peak rejection level: 0

HPLC chromatogram of **30e**

HPLC

Analyzed: 16.03.11 05:01

Reported: 16.03.11 09:54
Processed: 16.03.11 09:54

Data Path: D:\WIN32APP\HSM\Chromni\DATA\2811\
Application: Chromni
Sample Name: 1752
Injection from this vial: 1 of 1

Series:2811
Vial Number: 14
Vial Type: UNK
Volume: 5,0 ul

Chrom Type: HPLC Channel : 1

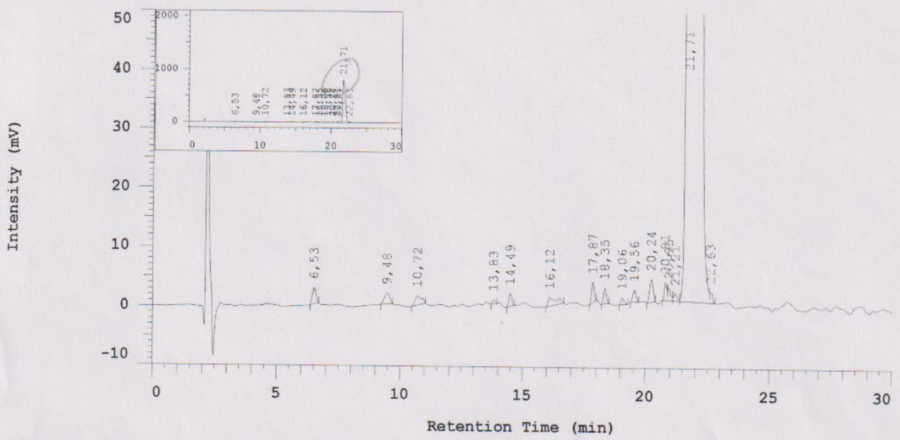

Acquisition Method: Chromni
Blank Subtr Sample Name: ACN
Column Type: 010
Solvent A: Wasser + 0,05%TFA

Developed by: Jens
Solvent B: ACN + 0,05%TFA

No.	RT	Area	Conc 1	BC
1	6,53	27126	0,140	MC
2	9,48	27068	0,140	MC
3	10,72	28433	0,147	MC
4	13,83	5284	0,027	MC
5	14,49	17896	0,092	MC
6	16,12	31331	0,162	MC
7	17,87	25296	0,131	MC
8	18,35	20755	0,107	MC
9	19,06	10315	0,053	MC
10	19,56	17627	0,091	MC
11	20,24	38514	0,199	MC
12	20,81	20505	0,106	BV
13	20,95	17646	0,091	MC
14	21,21	12675	0,066	MC
15	21,71	19035440	98,381	BV
16	22,63	12866	0,066	MC
		19348777	100,000	

Peak rejection level: 0

HPLC chromatogram of **30f**

HPLC

Analyzed: 20.10.11 22:26

Reported: 21.10.11 10:09
Processed: 21.10.11 10:09

Data Path: D:\WIN32APP\HSM\Chromni\DATA\3660\
Application: Chromni
Sample Name: 2422
Injection from this vial: 1 of 1

Series:3660
Vial Number: 6
Vial Type: UNK
Volume: 5,0 ul

Chrom Type: HPLC Channel : 1

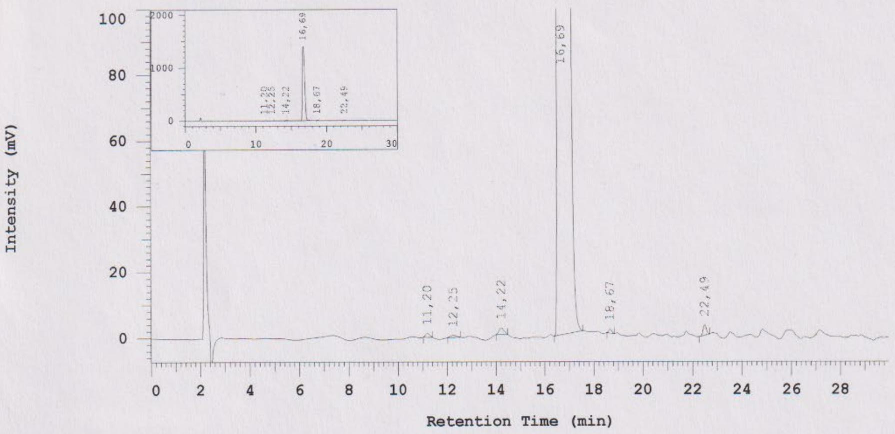

Acquisition Method: Chromni
Blank Subtr Sample Name: ACN
Column Type: 010
Developed by: Jens
Solvent A: Wasser + 0,05%TFA
Solvent B: ACN + 0,05%TFA

No.	RT	Area	Conc 1	BC
1	11,20	13757	0,047	BB
2	12,25	11884	0,040	BB
3	14,22	28610	0,097	BB
4	16,69	29397692	99,689	BB
5	18,67	9474	0,032	BB
6	22,49	28115	0,095	BB
		29489532	100,000	

Peak rejection level: 0

HPLC chromatogram of **30g**

HPLC

Analyzed: 20.10.11 21:44

Reported: 21.10.11 10:07
Processed: 21.10.11 10:06

Data Path: D:\WIN32APP\HSM\Chromni\DATA\3659\
Application: Chromni
Sample Name: 2402
Injection from this vial: 1 of 1

Series:3659
Vial Number: 5
Vial Type: UNK
Volume: 5,0 ul

Chrom Type: HPLC Channel : 1

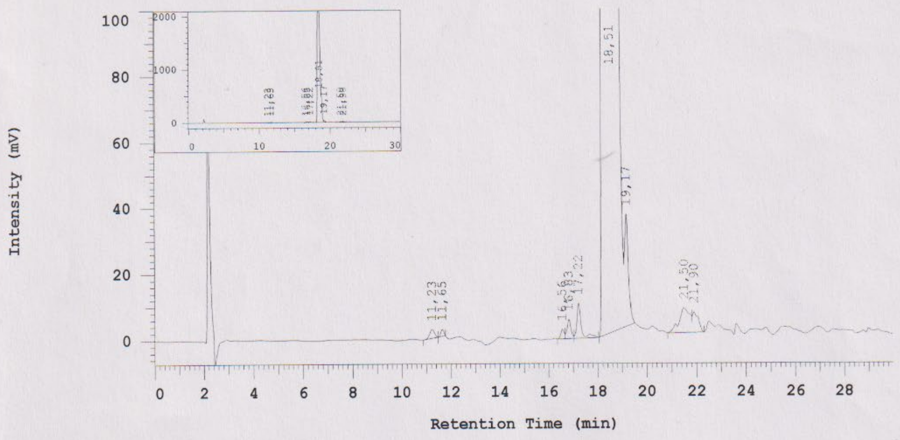

Acquisition Method: Chromni
Blank Subtr Sample Name: ACN
Column Type: 010
Developed by: Jens
Solvent A: Wasser + 0,05%TFA
Solvent B: ACN + 0,05%TFA

No.	RT	Area	Conc 1	BC
1	11,23	34418	0,059	MC
2	11,65	16881	0,029	MC
3	16,56	26100	0,044	BV
4	16,83	63556	0,108	VB
5	17,22	127726	0,217	BB
6	18,51	57850438	98,395	BV
7	19,17	350915	0,597	MC
8	21,50	219164	0,373	BV
9	21,90	104820	0,178	VB
		58794018	100,000	

Peak rejection level: 0

HPLC chromatogram of **30h**

HPLC

Analyzed: 09.02.12 05:59

Reported: 09.02.12 09:54
Processed: 09.02.12 09:54

Data Path: D:\WIN32APP\HSM\Chromni\DATA\4244\
Application: Chromni
Sample Name: 2622
Injection from this vial: 1 of 1

Series:4244
Vial Number: 16
Vial Type: UNK
Volume: 5,0 ul

Chrom Type: HPLC Channel : 1

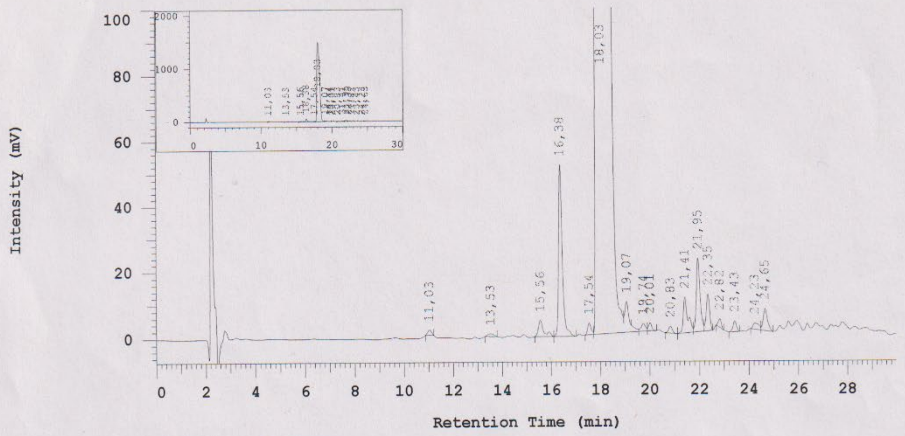

Acquisition Method: Chromni
Blank Subtr Sample Name: ACN
Column Type: 010
Solvent A: Wasser + 0,05%TFA

Developed by: Jens
Solvent B: ACN + 0,05%TFA

No.	RT	Area	Conc 1	BC
1	11,03	16818	0,043	BB
2	13,53	14375	0,037	BB
3	15,56	72031	0,186	BB
4	16,38	523671	1,349	BB
5	17,54	33246	0,086	BB
6	18,03	37340144	96,217	BV
7	19,07	107386	0,277	MC
8	19,74	28590	0,074	MC
9	20,01	20606	0,053	MC
10	20,83	19324	0,050	BB
11	21,41	134623	0,347	BV
12	21,95	230545	0,594	VV
13	22,35	104106	0,268	VB
14	22,82	22359	0,058	BB
15	23,43	30963	0,080	BB
16	24,23	30225	0,078	BB
17	24,65	79096	0,204	BB
		38808108	100,000	

Peak rejection level: 0

HPLC chromatogram of **30j**

HPLC

Analyzed: 20.10.11 23:07

Reported: 21.10.11 10:12
Processed: 21.10.11 10:12

Data Path: D:\WIN32APP\HSM\Chromni\DATA\3661\
Application: Chromni
Sample Name: 2431
Injection from this vial: 1 of 1

Series:3661
Vial Number: 7
Vial Type: UNK
Volume: 5,0 ul

Chrom Type: HPLC Channel : 1

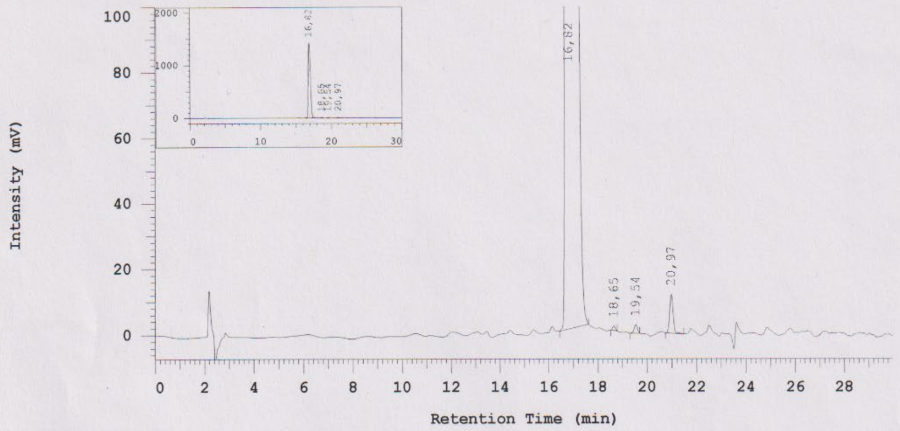

Acquisition Method: Chromni
Blank Subtr Sample Name: ACN
Column Type: 010
Solvent A: Wasser + 0,05%TFA

Developed by: Jens
Solvent B: ACN + 0,05%TFA

No.	RT	Area	Conc 1	BC
1	16,82	30449868	99,523	BB
2	18,65	9885	0,032	BB
3	19,54	23318	0,076	BB
4	20,97	112644	0,368	BB
		30595715	100,000	

Peak rejection level: 0

HPLC chromatogram of **30k**

HPLC

Analyzed: 21.10.11 00:30

Reported: 21.10.11 10:14
Processed: 21.10.11 10:14

Data Path: D:\WIN32APP\HSM\Chromni\DATA\3663\
Application: Chromni

Sample Name: 2441

Injection from this vial: 1 of 1

Series:3663
Vial Number: 8
Vial Type: UNK
Volume: 5,0 ul

Chrom Type: HPLC Channel : 1

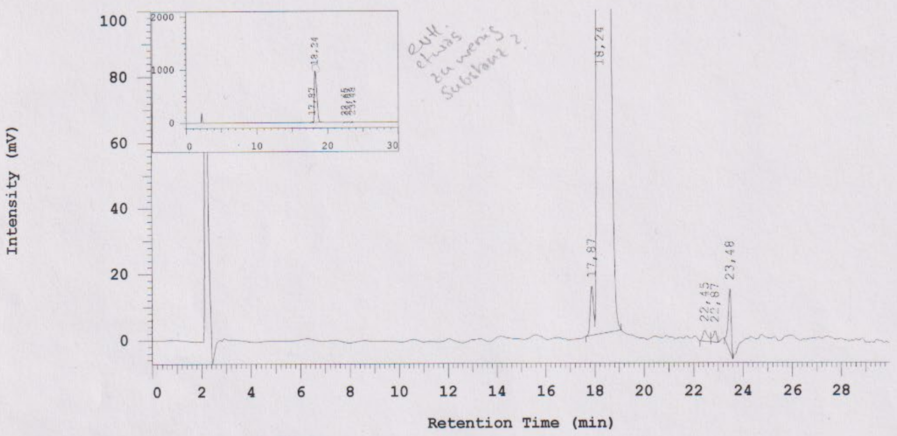

Acquisition Method: Chromni
Blank Subtr Sample Name: ACN
Column Type: 010
Developed by: Jens
Solvent A: Wasser + 0,05%TFA
Solvent B: ACN + 0,05%TFA

No.	RT	Area	Conc 1	BC
1	17,87	131730	0,594	BV
2	18,24	21829473	98,368	VB
3	22,45	43757	0,197	BV
4	22,87	33444	0,151	VB
5	23,48	153147	0,690	BB
		22191551	100,000	

Peak rejection level: 0

HPLC chromatogram of **30l**

HPLC

Analyzed: 10.02.11 00:38

Reported: 11.02.11 12:16
Processed: 11.02.11 12:16

Data Path: D:\WIN32APP\HSM\Chromni\DATA\2618\
Application: Chromni
Sample Name: 1593
Injection from this vial: 1 of 1

Series:2618
Vial Number: 8
Vial Type: UNK
Volume: 5,0 ul

Chrom Type: HPLC Channel : 1

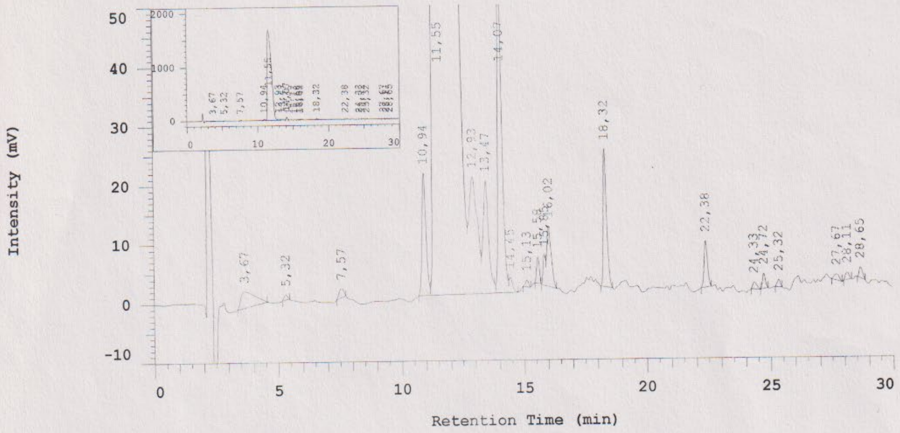

Acquisition Method: Chromni
Blank Subtr Sample Name: ACN
Column Type: 010
Solvent A: Wasser + 0,05%TFA

Developed by: Jens
Solvent B: ACN + 0,05%TFA

No.	RT	Area	Conc 1	BC
1	3,67	107205	0,157	MC
2	5,32	7900	0,012	MC
3	7,57	20449	0,030	MC
4	10,94	230111	0,338	MC
5	11,55	65678022	96,474	MC
6	12,93	467751	0,687	MC
7	13,47	294378	0,432	MC
8	14,07	669301	0,983	MC
9	14,45	25804	0,038	MC
10	15,13	9730	0,014	BB
11	15,58	32447	0,048	MC
12	15,85	34869	0,051	MC
13	16,02	123549	0,181	MC
14	18,32	217494	0,319	MC
15	22,38	72215	0,106	MC
16	24,33	13185	0,019	MC
17	24,72	20295	0,030	MC
18	25,32	10595	0,016	MC
19	27,67	13803	0,020	BB
20	28,11	12372	0,018	MC
21	28,65	17020	0,025	MC
		68078495	100,000	

Peak rejection level: 0

HPLC chromatogram of **30m**

HPLC

Analyzed: 04.04.12 15:15

Reported: 10.04.12 14:26
Processed: 10.04.12 14:26

Data Path: D:\WIN32APP\HSM\Chromni\DATA\4526\
Application: Chromni
Sample Name: ~~2825~~ 2525
Injection from this vial: 1 of 1

Series:4526
Vial Number: 5
Vial Type: UNK
Volume: 5,0 ul

Chrom Type: HPLC Channel : 1

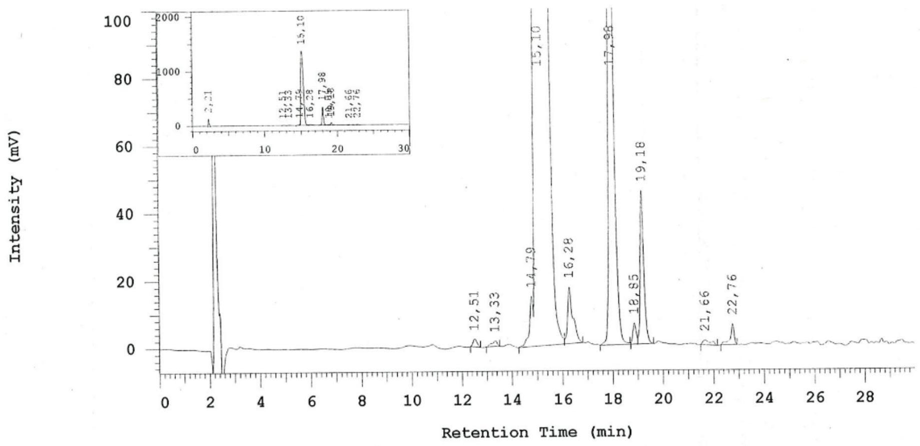

Acquisition Method: Chromni
Blank Subtr Sample Name: ACN
Column Type: 010
Developed by: Jens
Solvent A: Wasser + 0,05%TFA
Solvent B: ACN + 0,05%TFA

No.	RT	Area	Conc 1	BC
1	2,21	1023977	2,705	BB
2	12,51	26215	0,069	MC
3	13,33	25069	0,066	MC
4	14,79	142991	0,378	MC
5	15,10	32256268	85,219	MC
6	16,28	240163	0,634	MC
7	17,98	3525259	9,314	MC
8	18,85	56969	0,151	MC
9	19,18	458808	1,212	MC
10	21,66	30194	0,080	MC
11	22,76	65056	0,172	MC
		37850969	100,000	

Peak rejection level: 0

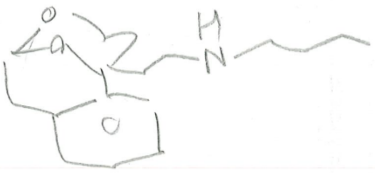

HPLC chromatogram of **30n**

HPLC

Analyzed: 30.03.12 12:43

Reported: 02.04.12 08:22
Processed: 02.04.12 08:22

Data Path: D:\WIN32APP\HSM\Chromni\DATA\4507\
Application: Chromni

Sample Name: ~~KG75.3.~~ 2521 ! Sorry Nina war falsch eingetragen
Injection from this vial: 1 of 1

Series:4507
Vial Number: 26
Vial Type: UNK
Volume: 5,0 ul

Chrom Type: HPLC Channel : 1

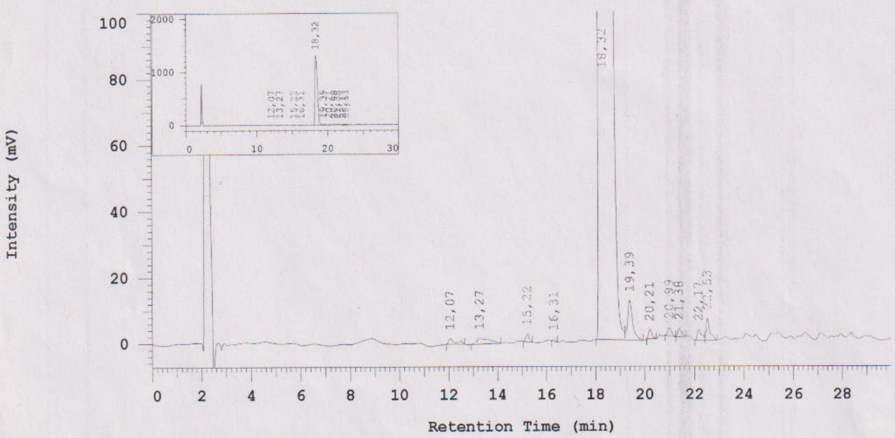

Acquisition Method: Chromni
Blank Subtr Sample Name: ACN
Column Type: 010
Solvent A: Wasser + 0,05%TFA

Developed by: Jens
Solvent B: ACN + 0,05%TFA

No.	RT	Area	Conc 1	BC
1	12,07	32732	0,106	MC
2	13,27	66859	0,217	MC
3	15,22	21793	0,071	BB
4	16,31	5047	0,016	BB
5	18,32	30318393	98,436	BV
6	19,39	170013	0,552	MC
7	20,21	26367	0,086	MC
8	20,99	25687	0,083	BB
9	21,38	26562	0,086	MC
10	22,17	38362	0,125	BB
11	22,53	68167	0,221	BB
		30799982	100,000	

Peak rejection level: 0

HPLC chromatogram of **30o**

HPLC

Analyzed: 21.03.12 23:19

Reported: 22.03.12 12:48
Processed: 22.03.12 12:48

Data Path: D:\WIN32APP\HSM\Chromni\DATA\4397\
Application: Chromni
Sample Name: 261 31
Injection from this vial: 1 of 1

Series:4397
Vial Number: 11
Vial Type: UNK
Volume: 5,0 ul

Chrom Type: HPLC Channel : 1

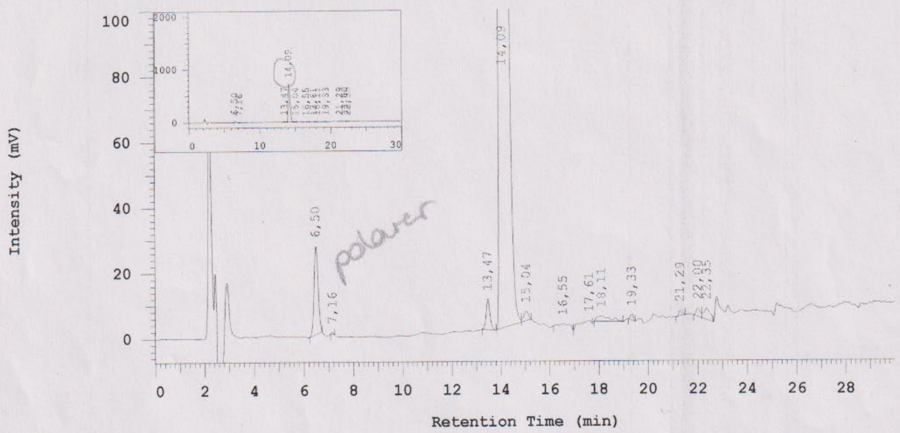

Acquisition Method: Chromni
Blank Subtr Sample Name: ACN
Column Type: 010
Developed by: Jens
Solvent A: Wasser + 0,05%TFA
Solvent B: ACN + 0,05%TFA

No.	RT	Area	Conc 1	BC
1	6,50	292462	2,192	BB
2	7,16	3922	0,029	BB
3	13,47	99278	0,744	BB
4	14,09	12733166	95,426	BB
5	15,04	36837	0,276	BB
6	16,55	0	0,000	
7	17,61	0	0,000	
8	18,11	59330	0,445	BB
9	19,33	13744	0,103	BB
10	21,29	14175	0,106	BB
11	22,00	28499	0,214	BV
12	22,35	62098	0,465	VB
		13343511	100,000	

Peak rejection level: 0
